# A Cu‐Catalysed Radical Cross‐Dehydrogenative Coupling Approach to Acridanes and Related Heterocycles

**DOI:** 10.1002/ejoc.201601336

**Published:** 2017-01-06

**Authors:** Timothy E. Hurst, Richard J. K. Taylor

**Affiliations:** ^1^ Department of ChemistryUniversity of YorkHeslingtonYO10 5DDYorkUK

**Keywords:** Acridanes, Copper, Homogeneous catalysis, Nitrogen heterocycles, Cross‐coupling, Dehydrogenation, One‐pot reaction

## Abstract

The synthesis of acridanes and related compounds through a Cu‐catalysed radical cross‐dehydrogenative coupling of simple 2‐[2‐(arylamino)aryl]malonates is reported. This method can be further streamlined to a one‐pot protocol involving the in situ fomation of the 2‐[2‐(arylamino)aryl]malonate by α‐arylation of diethyl malonate with 2‐bromodiarylamines under Pd catalysis, followed by Cu‐catalysed cyclisation.

## Introduction

In recent years C–H activation has emerged as a powerful and attractive method in organic synthesis since it enables the formation of C–C bonds without pre‐functionalisation of one or both of the coupling partners, which leads to more efficient and atom‐economical processes. Within the wider pantheon of C–H activation, cross‐dehydrogenative couplings (CDC) have proved to be versatile procedures for the selective formation of C–C bonds from two different C–H systems under oxidative conditions.^1^ Our contribution in this area involves the synthesis of diverse nitrogen heterocycles through the Cu‐catalysed oxidative coupling of C_sp2_–H and C_sp3_–H bonds;^2^ a radical variant of the CDC process.

**Figure 1:**
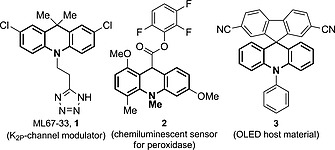
Examples demonstrating the utility of acridanes.

9,10‐Dihydroacridines (acridanes) have garnered much attention^3^ due to their potent biological activity, including inhibition of histone deacetylase (class IIa)^4^ and HIV reverse transcriptase,^5^ neuroleptic activity^6^ and as activators of K_2P_ potassium channels^7^ (e.g., **1**, Figure 1). Furthermore, acridanes have been employed as chemiluminescent sensors in immunoassays^8^ (e.g., **2**), as NADH analogues in hydride‐transfer reactions,^9^ as host materials in OLEDs^10^ (e.g., **3**), as photoswitches^11^ and as molecular motors.^12^ Moreover, acridanes are valuable building blocks that are readily oxidised to acridines or acridones,^13^ functionalised by C–H activation^14^ and easily converted into 5*H*‐dibenzo[*b*,*f*]azepines or 5,11‐dihydro‐10*H*‐dibenzo[*b*,*e*][1,4]diazepines by ring expansion.^15^

**Scheme 1:**
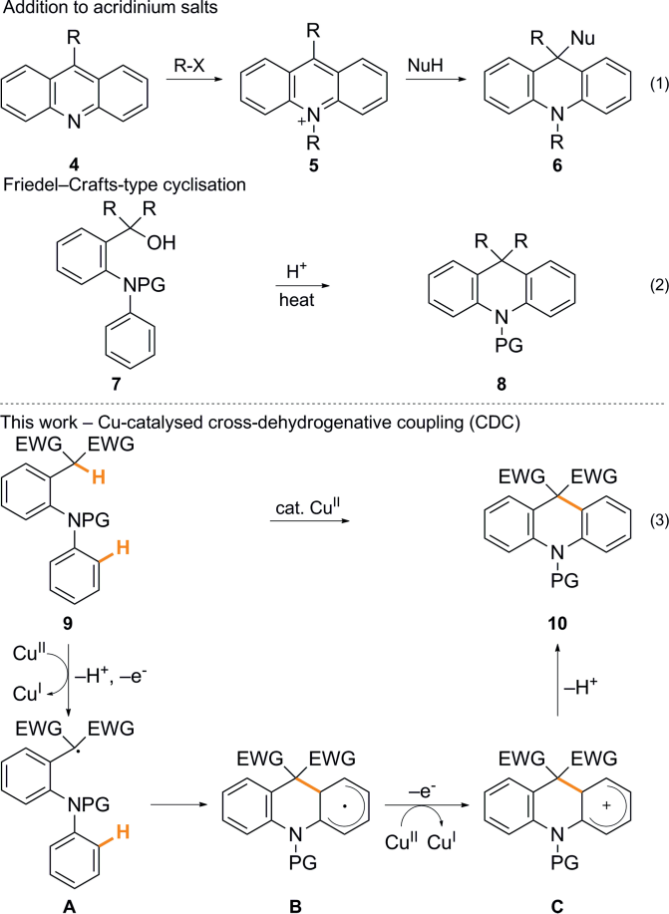
Approaches for the synthesis of acridanes.

Given the proven utility of acridanes, the sparse number of general methods available for their preparation is surprising.^3^ Traditionally, they have been prepared by nucleophilic addition to acridines **4** or acridinium salts **5** (Scheme 1, eq 1),^16^ or by a Friedel–Crafts‐type cyclisation of diarylamines **7** with strong acid (Scheme 1, eq 2).^17^ However, the need to rely either on the commercial availability or on a potentially lengthy synthesis of acridines **4**, in combination with the requirements for the use of strong acids and well‐known problems of site selectivity associated with Friedel–Crafts cyclisations, means that new approaches to highly substituted acridane derivatives are of considerable interest.

Thus, in continuation of our studies on the synthesis of diverse heterocyclic scaffolds by oxidative cyclisation of linear precursors with inexpensive Cu salts,^2^ our goal was to exploit this simple yet powerful methodology in the preparation of acridane derivatives **10** from 2‐[2‐(arylamino)aryl]malonates **9** (Scheme 1, eq 3).

The likely mechanism for this process, by analogy with earlier studies,[2d], [2f] is shown in Scheme 1. Initially, proton abstraction and single‐electron oxidation would give malonyl radical **A**, which would undergo intramolecular homolytic aromatic substitution to give **B**, followed by further oxidation to give the cyclohexadienyl cation **C**, which would finally aromatise to generate the desired product **10**. Evidence for a radical‐based mechanism in these Cu‐mediated oxidative coupling reactions includes radical‐clock experiments,[2d] as well as DFT calculations conducted by Kündig in related systems.[2f] Clearly, two equivalents of Cu salt are nominally required to effect both single‐electron oxidations involved in the process. However, in related studies we have demonstrated that a catalytic amount of the Cu salt may be used with air serving as the terminal oxidant.

## Results and Discussion

**Scheme 2:**
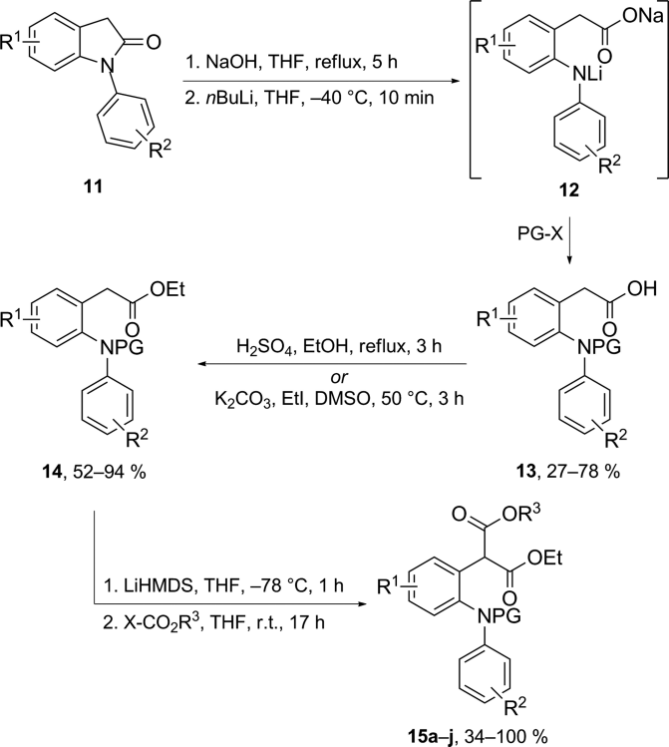
Modular synthesis of cyclisation precursors **15a**–**j**.

The initial task was to develop a flexible and modular approach for the synthesis of the cyclisation precursors **15** (Scheme 2), which would allow the facile introduction of substituents at various positions on the acridane skeleton.

In the event, *N*‐aryloxindoles **11** proved to be versatile starting materials for the preparation of cyclisation precursors **15** by a three‐step protocol (Scheme 2). First, ring‐opening of the lactam moiety was carried out by treatment with NaOH in refluxing THF, followed by in situ formation of the dianions **12** by addition of *n*BuLi at low temperature and subsequent trapping with the appropriate electrophile to give carboxylic acids **13**.^18^ Then, simple esterification, deprotonation with LiHMDS and trapping with the requisite cyano‐ or chloroformate delivered 2‐[2‐(arylamino)aryl]malonates **15a**–**j**. In this manner, control over the substitution pattern on the aromatic rings, as well as on the nitrogen atom and ester, functionality could be readily achieved (14–63 % unoptimised overall yields; see the Supporting Information for details).

**Scheme 3:**
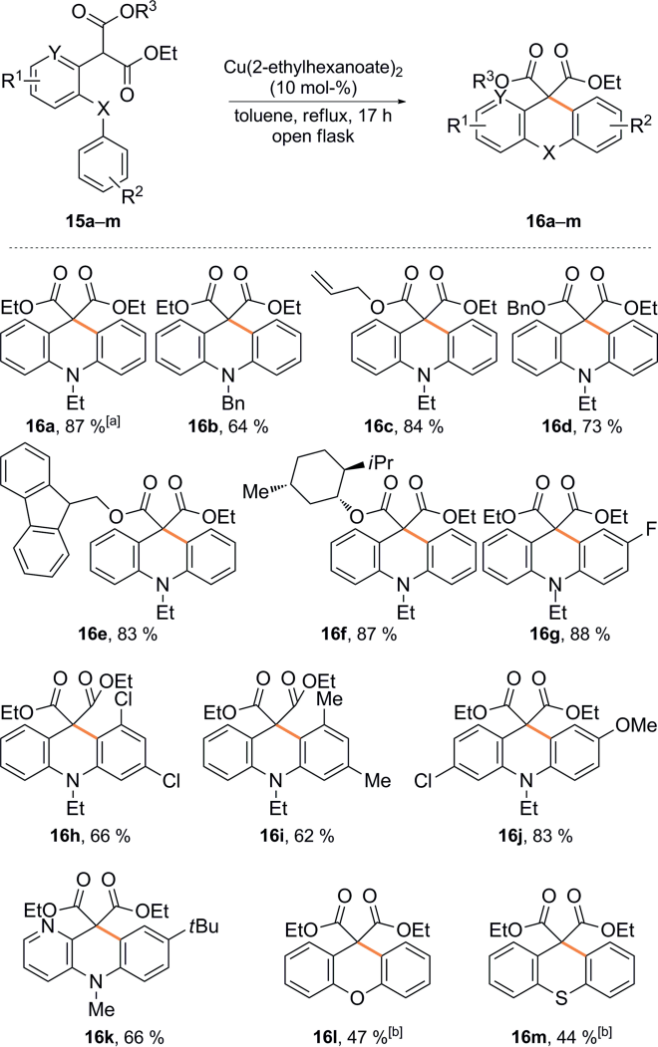
Scope of the Cu‐catalysed synthesis of acridanes. [a] **16a** was obtained in 83 and 84 % yield when Cu(2‐ethylhexanoate)_2_ (5 mol‐% and 2 mol‐%) were used, respectively. [b] Cu(2‐ethylhexanoate)_2_ (2.5 equiv.) and DIPEA (2.5 equiv.) were used under an atmosphere of argon.

With ready access to the required linear substrates in hand, the cyclisation of model substrate **15a** was then examined. Pleasingly, treatment of **15a** under the conditions previously reported[2b] in our synthesis of oxindoles [Cu(2‐ethylhexanoate)_2_ (10 mol‐%), toluene, reflux, open flask] delivered the desired acridane **16a** in 87 % yield without the need for further optimisation (Scheme 3). It is also noteworthy that the oxidative coupling could be performed in the presence of only 5 mol‐% or 2 mol‐% of the catalyst, with only a minor drop in yield (Scheme 3, footnote a).

By following this successful initial result, the generality of this new Cu‐catalysed approach to acridanes was explored (Scheme 3). First, cyclisation of **15b**, which bears a removable protecting group on the nitrogen atom, was carried out to give *N*‐benzylacridane **16b**, albeit in reduced yield in comparison to *N*‐ethyl derivative **16a**. Next, acridanes **16c**–**f** bearing differentially protected esters (e.g., allyl, Bn, Fmoc) were prepared, which opens the possibility for further manipulations of the cyclised products (vide infra). The Fmoc‐protected ester **16e** was crystalline, which allowed unambiguous confirmation of its structure through X‐ray crystallographic analysis (see the Supporting Information).

Next, the introduction of substituents at various positions on either aromatic ring of the acridane skeleton was explored, which gave **16g**–**j** in good to excellent yields. Incorporation of a nitrogen atom into one of the aromatic rings was also possible; isolation of the corresponding aza‐acridane **16k** was achieved in 66 % yield.

Having established the effectiveness of the Cu‐catalysed route to acridanes **16a**–**k**, we extended this method to the synthesis of other related heterocycles of interest, such as xanthenes and thioxanthenes (Scheme 3, **16l**–**m**). However, cyclisation of a linear diaryl ether in the presence of 10 mol‐% Cu(2‐ethylhexanoate)_2_ under the standard reaction conditions delivered xanthene **16l** in a disappointing yield of 33 %. Also isolated was an equal amount of a by‐product, identified as ethyl 2‐oxo‐2‐(2‐phenoxyphenyl)acetate, which arises from competing decarboxylation and aerial oxidation of the starting material. This problem was exacerbated in the case of thioxanthene **16m**, which was isolated in only 20 % yield along with 53 % of the undesired by‐product. Further optimisation of these reactions is clearly required but fortunately, in both cases, the yield of the oxidation by‐products could be minimised by performing the cyclisation under an atmosphere of argon, which leads to formation of xanthene **16l** and thioxanthene **16m** in 47 and 44 % yields, respectively. While formation of the desired products **16l**–**m** is enhanced under these conditions, performing the reaction under argon necessitates the use of 2.5 equiv. of Cu salt to allow the reaction to proceed to completion (see proposed mechanism, Scheme 1).

**Scheme 4:**
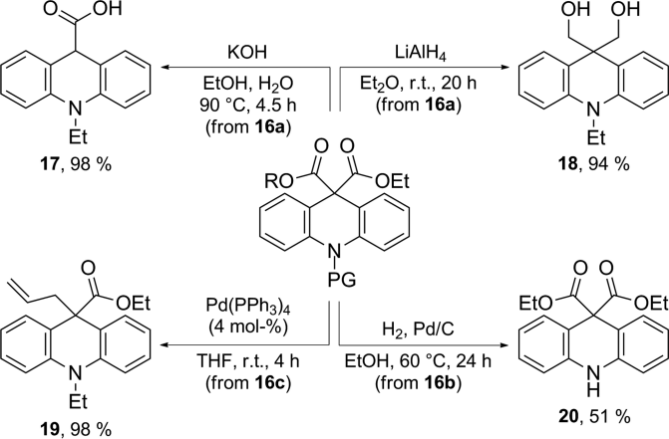
Further derivatisation of acridanes obtained from oxidative coupling.

In order to demonstrate the utility of the acridanes derived from the Cu‐catalysed cyclisation, a brief study on their further functionalisation was carried out (Scheme 4). For example, treatment of **16a** with an excess of KOH in EtOH/H_2_O resulted in saponification and decarboxylation to give acid **17** in excellent yield. This provides an alternative route, which may be useful in the synthesis of chemiluminescent sensors analogous to **2** (Figure 1). Reduction of the esters was also achieved in the presence of LiAlH_4_ to give diol **18** in 94 % yield. Furthermore, selective functionalisation of the allyl ester in **16c** was carried out by decarboxylative allylic alkylation in the presence of 4 mol‐% Pd(PPh_3_)_4_ to give **19** and thereby generate a new quaternary carbon centre, again in excellent yield. Finally, hydrogenolysis of the benzyl protecting group in **16b** delivered acridane **20** bearing a free N–H group in reasonable, un‐optimised yield.

**Scheme 5:**
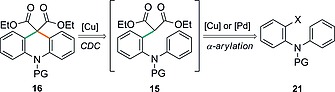
Retrosynthesis of acridanes **16** through a one‐pot α‐arylation–cyclisation process.

As a final aspect to this work, we explored the potential of an expedited, one‐pot approach to acridanes **16** based on the α‐arylation of 1,3‐dicarbonyl compounds with haloarenes **21** to generate intermediates **15** in situ, which would then undergo the oxidative coupling reaction (Scheme 5). Given the well‐known ability of Cu salts to catalyse both processes,^19^ a highly efficient transformation seemed attainable.

**Scheme 6:**
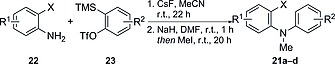
Synthesis of 2‐halodiarylamines **21a**–**d**.

The requisite 2‐halodiarylamines **21** were easily prepared by the addition of commercially available 2‐haloanilines **22** to benzynes, which were prepared in situ from 2‐(trimethylsilyl)phenyl triflates **23** in the presence of CsF, and subsequent *N*‐alkylation (Scheme 6; see the Supporting Information for more details).^20^

Next, the crucial transition‐metal‐catalysed α‐arylation reaction between diethyl malonate and 2‐halodiarylamines **21a**–**b** was examined; selected results are shown in Table 1. In the event, none of the desired α‐arylation products was observed on heating either 2‐iodo‐ or 2‐bromo‐*N*‐methyl‐*N*‐phenylaniline **21a**–**b** with diethyl malonate in the presence of CuI and 2‐picolinic acid[19a] (Table 1, entries 1–2). Similarly, other Cu‐based catalyst systems, which are reported for the coupling of dialkyl malonates with simple haloarenes, proved equally ineffective (results not shown). Thus, attention turned to the use of palladium catalysts to effect the initial transformation.

**Table 1:** Optimisation of the α‐arylation of **21a**–**b** with diethyl malonate

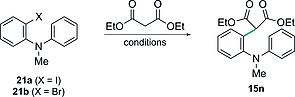			
Entry	X	Conditions	Yield [%]
1	I	CuI (10 mol‐%), 2‐picolinic acid (20 mol‐%)	–a
		Cs_2_CO_3_ (3 equiv.), dioxane, reflux, 17 h	
2	Br	CuI (10 mol‐%), 2‐picolinic acid (20 mol‐%)	–a
		Cs_2_CO_3_ (3 equiv.), dioxane, reflux, 17 h	
3	I	Pd(OAc)_2_ (2 mol‐%), *t*BuMePhos (4.4 mol‐%)	–a
		K_3_PO_4_ (2.4 equiv.), toluene, reflux, 18 h	
4	Br	Pd(OAc)_2_ (2 mol‐%), *t*BuMePhos (4.4 mol‐%)	–a
		K_3_PO_4_ (2.4 equiv.), toluene, reflux, 18 h	
5	I	Pd_2_dba_3_ **·**CHCl_3_ (1 mol‐%), *t*Bu_3_P**·**HBF_4_ (4 mol‐%)	32
		K_3_PO_4_ (3 equiv.), toluene, 70 °C, 15 h	
6	I	Pd_2_dba_3_ **·**CHCl_3_ (1 mol‐%), *t*Bu_3_P**·**HBF_4_ (4 mol‐%)	61
		K_3_PO_4_ (3 equiv.), toluene, reflux, 14 h	
**7**	**Br**	**Pd_2_dba_3_·CHCl_3_ (1 mol‐%), *t*Bu_3_P·HBF_4_ (4 mol‐%)**	**72**
		**K_3_PO_4_ (3 equiv.), toluene, reflux, 17 h**	

a Starting materials only were observed by ^1^H NMR analysis of the unpurified reaction mixture.

No α‐arylation was observed when using the Pd(OAc)_2_/*t*BuMePhos catalyst system developed by Buchwald (Table 1, entries 3–4),^21^ but an encouraging yield of 32 % was obtained for **15n** by switching to Pd_2_dba_3_ **·**CHCl_3_ (1 mol‐%) as the catalyst with the bench stable ligand *t*Bu_3_P**·**HBF_4_ (4 mol‐%) in toluene at 70 °C (Table 1, entry 5).^22^ Increasing the reaction temperature allowed us to isolate 2‐[2‐(arylamino)aryl]malonate **15n** in 61 % (from **21a**) and 72 % (from **21b**) yields, respectively.

**Scheme 7:**
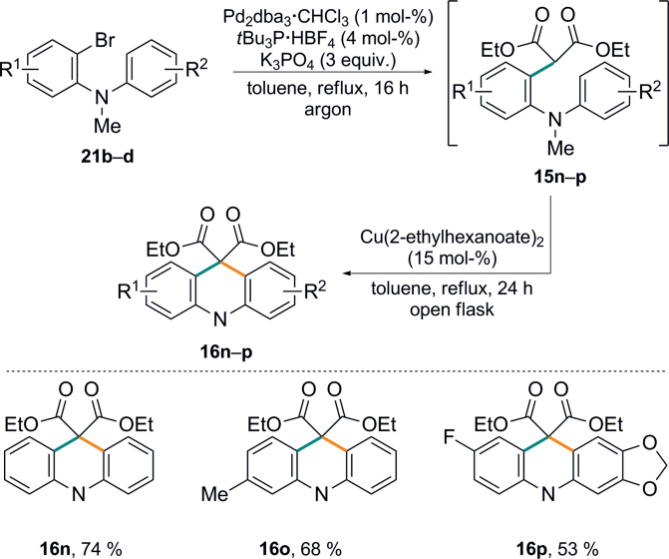
Scope of the one‐pot α‐arylation–cyclisation approach to acridanes.

With conditions for the malonate coupling in hand, our attention then turned to establishing the one‐pot α‐arylation–cyclisation protocol to prepare acridanes directly (Scheme 7). To this end, the Pd‐catalysed α‐arylation reaction between 2‐bromo‐*N*‐methyl‐*N*‐phenylaniline **21b** and diethyl malonate was first carried out by using the optimised conditions described above under an atmosphere of argon. Subsequently, the reaction flask was simply opened to the air, Cu(2‐ethylhexanoate)_2_ (15 mol‐%) was added and heating continued for a further 24 h to facilitate the cyclisation. In this manner, the target acridane **16n** was isolated in a pleasing yield of 74 % over the 2 steps. Furthermore, substituents could be readily introduced on one or both of the aromatic rings, which allows access to more highly substituted acridanes such as **16o** and **16p**.

## Conclusions

In summary, we report a Cu‐catalysed radical cross‐dehydrogenative‐coupling approach to acridanes and related heterocycles from readily available 2‐[2‐(arylamino)aryl]malonates. This highly atom‐economical method uses inexpensive Cu(2‐ethylhexanoate)_2_ as the catalyst under mild conditions and thus avoids many of the problems associated with classical strategies for the synthesis of acridanes. The diester moiety resulting from the oxidative coupling reaction serves as a useful handle for further functionalisation. In addition, we have established a streamlined protocol involving the in situ formation of the cyclisation precursor by the α‐arylation of diethyl malonate with a 2‐bromodiarylamine under Pd catalysis, followed by subsequent Cu‐catalysed cyclisation to give the acridanes in a one‐pot fashion. Further studies will be carried out to utilise this new method in target synthesis.

## Experimental Section

**Representative Procedure for the Cu‐catalysed Synthesis of Acridanes:** To a solution of the cyclisation precursor **15** (1.00 mmol) in toluene (10 mL) was added Cu(2‐ethylhexanoate)_2_ (35.0 mg, 0.100 mmol). The reaction mixture was heated to reflux (oil bath at 120 °C) for 17 h with the condenser left open to the air. After cooling to room temp., saturated NH_4_Cl soln (25 mL) was added, and the aqueous phase extracted with EtOAc (3 × 25 mL). The combined organics were washed with NH_4_OH soln (10 %, 25 mL), dried with MgSO_4_, filtered and concentrated in vacuo. Purification by flash column chromatography with EtOAc/hexane as eluent afforded the title compound **16** (see the Supporting Information for details).

CCDC 1498037 (for **16e**) contain the supplementary crystallographic data for this paper. These data can be obtained free of charge from The Cambridge Crystallographic Data Centre.

## Supporting information

Supporting Information
