# Supplementary material for: A Cu‐Catalysed Radical Cross‐Dehydrogenative Coupling Approach to Acridanes and Related Heterocycles
Source: European J Org Chem. 2017 Jan 6;2017(1):203–7. doi: 10.1002/ejoc.201601336 (PMC5396374; doi:10.1002/ejoc.201601336)
Supplement: Supplementary file 1 — Supporting Information [file EJOC-2017-203-s001.pdf]

Eur. J. Org. Chem. **2017** · ISSN 1099–0690

SUPPORTING INFORMATION

DOI: 10.1002/ejoc.201601336
Title: A Cu-Catalysed Radical Cross-Dehydrogenative Coupling Approach to Acridanes and Related Heterocycles
Author(s): Timothy E. Hurst*, Richard J. K. Taylor*

Table of Contents

1.0 General Information **S3**

2.0 Modular Synthesis of Cyclisation Precursors **S4**

General Procedure 1 – *N*-Arylation of 2-Oxindoles **S4**

General Procedure 2 – Ring-Opening/*N*-Alkylation of 2-Oxindoles and Esterification **S8**

General Procedure 3 – Acylation of Ethyl Phenylacetates **S15**

3.0 Copper(II)-Catalysed Synthesis of Acridanes **S26**

Optimisation of the Cyclisation Reaction to form Acridanes **S26**

General Procedure 4 – Copper(II)-Catalysed Cyclisations **S26**

4.0 Derivatisation of Acridanes **S35**

5.0 One-Pot α-Arylation/Cyclisation Approach to Acridanes **S38**

General Procedure 5 – Synthesis of 2-Bromo-*N*-phenylanilines **S38**

General Procedure 6 – Methylation of 2-Bromo-*N*-phenylanilines **S40**

General Procedure 7 – One-Pot α-Arylation/Cyclisation Approach to Acridanes **S43**

6.0 Copies of 1H/13C NMR, Mass Spec, and IR Spectra **S46**

1.0 General Information

Except where stated, all reagents were purchased from commercial sources and used without further purification. Except where stated, all experimental procedures were carried out under an atmosphere of argon. Anhydrous solvents (CH_2Cl_2, toluene) were obtained from an Innovative Technologies solvent purification system. Anhydrous THF was obtained by distillation over sodium benzophenone ketyl immediately before use. NMR spectra were recorded either on a JEOL spectrometer operating at 400 MHz (1H) and 100 MHz (13C), or a Bruker AV500 spectrometer operating at 500 MHz (1H) and 125 MHz (13C). All spectral data were acquired at 295 K. Chemical shifts (δ) are quoted in parts per million (ppm). The residual solvent peak, δ_H 7.26 ($CHCl_3$) and δ_C 77.0 ($CDCl_3$), δ_H 3.31 (MeOH) and δ_C 49.0 (MeOD-*d4*) and δ_H 2.50 (DMSO) and δ_C 39.5 (DMSO-*d6*) was used as a reference. Coupling constants (*J*) are reported in Hertz (Hz) to the nearest 0.1 Hz. The multiplicity abbreviations used are: s singlet, d doublet, t triplet, q quartet, m multiplet. Signal assignment was achieved by analysis of DEPT, COSY, NOESY, HMBC and HSQC experiments where required. Infrared (IR) spectra were recorded neat on a Perkin-Elmer Spectrum Two FTIR-ATR spectrometer. Mass-spectra (low and high-resolution) were obtained using electrospray ionisation (ESI) on a Micro-TOF spectrometer. Melting points were recorded in capillary tubes on a Gallenkamp apparatus. Thin layer chromatography was carried out on silica gel 60F254 pre-coated aluminium foil sheets and were visualised using UV light (254 nm) and stained with basic aqueous potassium permanganate. Flash column chromatography was carried out using slurry packed silica gel (SiO_2), 35–75 Dm particle size, 60 Å pore size, under a light positive pressure, eluting with the specified solvent system.

2.0 Modular Synthesis of Cyclisation Precursors

General Procedure 1 – *N*-Arylation of 2-Oxindoles[1,2]

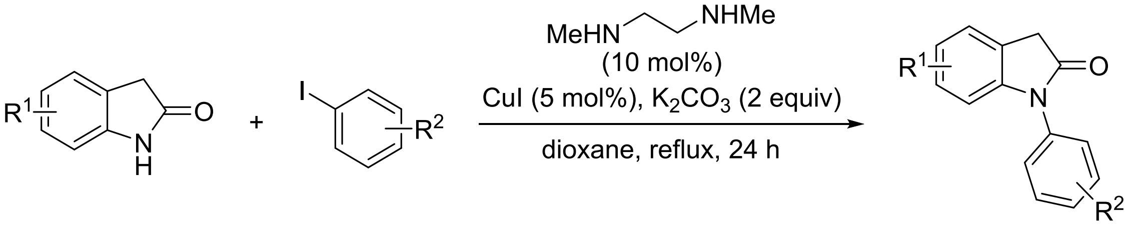

To a solution of the iodoarene (8.00 mmol), 2-oxindole (9.60 mmol), CuI (76.0 mg, 0.400 mmol) and K_2CO_3 (2.21 g, 16.0 mmol) in dioxane (8 mL) was added *N*,*N'*-dimethylethylenediamine (86.0 µL, 0.800 mmol). The reaction mixture was heated at reflux for 24 h, then cooled to rt and partitioned between 10% HCl (50 mL) and EtOAc (3 × 50 mL). The combined organics were dried over $MgSO_4$, filtered, and concentrated *in vacuo*. Purification by flash column chromatography, eluting with EtOAc/Hexane, afforded the title compound.

1-Phenylindolin-2-one 11a

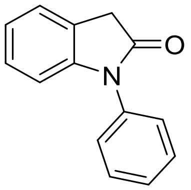

Iodobenzene (2.24 mL, 20.0 mmol), 2-oxindole (3.15 g, 24.0 mmol), CuI (0.190 g, 1.00 mmol), *N*,*N'*-dimethylethylenediamine (0.215 mL, 2.00 mmol) and K_2CO_3 (5.53 g, 40.0 mmol) in dioxane (20 mL) were subjected to General Procedure 1. Purification by flash column chromatography, eluting with EtOAc/Hexane (1:9 to 1:4), afforded the title compound as a colourless solid (3.48 g, 83%).

MP: 121-122 °C (lit.[3] 121-122 °C).

1H NMR (400 MHz; $CDCl_3$): δ 7.56-7.51 (m, 2 H), 7.43-7.40 (m, 3 H), 7.32 (d, *J* = 7.2 Hz, 1 H), 7.21 (td, *J* = 7.8 and 0.9 Hz, 1 H), 7.08 (td, *J* = 7.5 and 0.6 Hz, 1 H), 6.80 (d, *J* = 7.9 Hz, 1 H), 3.72 (s, 2 H).

13C NMR (100 MHz; $CDCl_3$): δ 174.4 (C), 145.2 (C), 134.4 (C), 129.6 (CH), 128.1 (CH), 127.7 (CH), 126.6 (CH), 124.6 (CH), 124.3 (C), 122.8 (CH), 109.4 (CH), 36.0 (CH_2).

Data is consistent with literature values.[1]

---

[1] X.-H. Xu, X. Wang, G.-k. Liu, E. Tokunaga and N. Shibata *Org. Lett.* **2012**, *14*, 2544-2547.
[2] R. A. Altman, A. M. Hyde, X. Huang and S. L. Buchwald *J. Am. Chem. Soc.* **2008**, *130*, 9613-9620.
[3] R. A. Franich, G. Lowe and J. Parker *J. Chem. Soc., Perkin Trans. 1* **1972**, 2034-2041.

1-(4-Fluorophenyl)indolin-2-one 11b

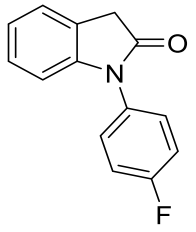

4-Fluoro-1-iodobenzene (0.92 mL, 8.00 mmol), 2-oxindole (1.26 g, 9.60 mmol), CuI (76.0 mg, 0.400 mmol), *N*,*N'*-dimethylethylenediamine (86.0 µL, 0.800 mmol) and K_2CO_3 (2.21 g, 16.0 mmol) in dioxane (8 mL) were subjected to General Procedure 1. Purification by flash column chromatography, eluting with EtOAc/Hexane (1:4), afforded the title compound as a colourless solid (1.70 g, 93%).

MP: 131-132 °C (lit.[2] 129-132 °C).

1H NMR (400 MHz; $CDCl_3$): δ 7.42-7.36 (m, 2 H), 7.32 (d, *J* = 7.2 Hz, 1 H), 7.25-7.19 (m, 3 H), 7.09 (td, *J* = 7.4 and 0.8 Hz, 1 H), 6.75 (d, *J* = 7.9 Hz, 1 H), 3.72 (s, 2 H).

13C NMR (100 MHz; $CDCl_3$): δ 174.5 (C), 161.9 (d, *J* = 247 Hz, C), 145.0 (C), 130.3 (d, *J* = 3.0 Hz, C), 128.5 (d, *J* = 8.3 Hz, CH), 127.8 (CH), 124.7 (CH), 124.2 (C), 122.9 (CH), 116.6 (d, *J* = 22.3 Hz, CH), 109.1 (CH), 35.9 (CH_2).

Data is consistent with literature values.[2]

1-(3,5-Dichlorophenyl)indolin-2-one 11c

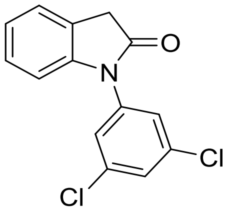

3,5-Dichloro-1-iodobenzene (2.18 g, 8.00 mmol), 2-oxindole (1.26 g, 9.60 mmol), CuI (76.0 mg, 0.400 mmol), *N*,*N'*-dimethylethylenediamine (86.0 µL, 0.800 mmol) and K_2CO_3 (2.21 g, 16.0 mmol) in dioxane (8 mL) were subjected to General Procedure 1. Purification by flash column chromatography, eluting with EtOAc/Hexane (1:4), afforded the title compound as a colourless solid (1.59 g, 71%).

MP: 145-146 °C.

1H NMR (400 MHz; $CDCl_3$): δ 7.41 (t, *J* = 1.9 Hz, 1 H), 7.37 (d, *J* = 1.9 Hz, 2 H), 7.32 (d, *J* = 7.3 Hz, 1 H), 7.25 (td, *J* = 7.7 and 0.9 Hz, 1 H), 7.12 (td, *J* = 7.7 and 0.9 Hz, 1 H), 6.84 d, *J* = 7.9 Hz, 1 H), 3.72 (s, 2 H).

13C NMR (100 MHz; $CDCl_3$): δ 174.0 (C), 143.9 (C), 136.3 (C), 135.8 (C), 128.2 (CH), 128.0 (CH), 125.1 (CH), 124.9 (CH), 124.1 (C), 123.4 (CH), 109.3 (CH), 35.9 (CH_2).

IR (ATR, cm^{-1}): 3085, 2915, 1727, 1614, 1583, 1484, 1465, 1443, 1396, 1359, 1318, 1245, 1229, 1199, 1166, 1093.

HRMS (ESI): Found MNa$^+$, 299.9948. $C_{14}H_9ClNNaO^+$ requires 299.9953. Δ = 1.9 ppm.

1-(3,5-Dimethylphenyl)indolin-2-one 11d

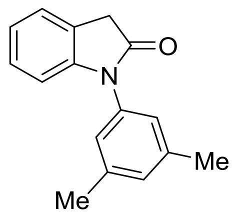

1-Iodo-3,5-dimethylbenzene (1.86 g, 8.00 mmol), 2-oxindole (1.26 g, 9.60 mmol), CuI (76.0 mg, 0.400 mmol), *N,N'*-dimethylethylenediamine (86.0 µL, 0.800 mmol) and K_2CO_3 (2.21 g, 16.0 mmol) in dioxane (8 mL) were subjected to General Procedure 1. Purification by flash column chromatography, eluting with EtOAc/Hexane (1:4), afforded the title compound as a colourless solid (1.44 g, 76%).

MP: 88-89 °C. (lit.[2] 92-94 °C).

1H NMR (400 MHz; $CDCl_3$): δ 7.31 (d, *J* = 7.3 Hz, 1 H), 7.20 (t, *J* = 7.5 Hz, 1 H), 7.08-7.05 (m, 2 H), 7.01-6.99 (m, 2 H), 6.75 (d, *J* = 7.9 Hz, 1 H), 3.71 (s, 2 H), 2.37 (s, 6 H).

13C NMR (100 MHz; $CDCl_3$): δ 174.6 (C), 145.5 (C), 139.4 (C), 134.2 (C), 130.0 (CH), 127.7 (CH), 124.5 (CH), 124.32 (CH), 124.26 (C), 122.6 (CH), 109.5 (CH), 36.0 (CH_2), 21.3 (Me).

Data is consistent with literature values.[2]

6-Chloro-1-(4-methoxyphenyl)indolin-2-one 11e

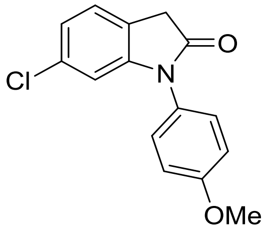

4-Iodoanisole (1.87 g, 8.00 mmol), 6-chloro-2-oxindole (1.61 g, 10.0 mmol), CuI (76.0 mg, 0.400 mmol), (*rac*)-*trans*-*N,N'*-dimethyl-1,2-cyclohexanediamine (0.126 mL, 0.800 mmol) and K_2CO_3 (2.21 g, 16.0 mmol) in dioxane (8 mL) were subjected to General Procedure 1. Purification by flash column chromatography, eluting with EtOAc/Hexane (1:9 to 1:4), afforded the title compound as a colourless solid (1.68 g, 77%).

MP: 109-111 °C.

1H NMR (400 MHz; $CDCl_3$): δ 7.29-7.25 (m, 2 H), 7.19 (dd, *J* = 7.9 and 0.8 Hz, 1 H), 7.05-7.00 (m, 3 H), 6.69 (d, *J* = 1.7 Hz, 1 H), 3.85 (s, 3 H), 3.65 (s, 2 H).

13C NMR (100 MHz; $CDCl_3$): δ 174.6 (C), 159.4 (C), 146.8 (C), 133.6 (C), 127.9 (CH), 126.4 (C), 125.4 (CH), 122.5 (CH), 122.4 (C), 115.1 (CH), 109.8 (CH), 55.5 (Me), 35.5 (CH_2).

IR (ATR, cm^{-1}): 3059, 3019, 2966, 2847, 1711, 1611, 1514, 1486, 1430, 1391, 1368, 1318, 1298, 1252, 1236, 1204, 1191, 1170, 1109, 1067, 1028, 997.

HRMS (ESI): Found MH^+, 274.0618. $C_{15}H_{13}ClNO_2^+$ requires 274.0629. Δ = 4.1 ppm.

General Procedure 2 – Ring-Opening/*N*-Alkylation of 2-Oxindoles and Esterification[4]

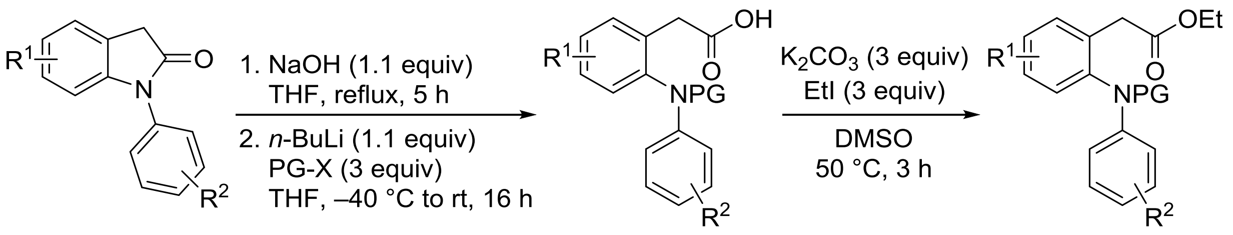

Step A: To a solution of the *N*-aryl-2-oxindole (5.00 mmol) in THF (2.5 mL) was added NaOH (0.220 g, 5.50 mmol). The reaction mixture was heated at reflux for 5 h before cooling to rt. Further THF (5 mL) was added and the solution cooled to –40 °C. *n*-BuLi (2.45 M in Hexane, 2.24 mL, 5.50 mmol) was then added dropwise over 5 min. Stirring was continued at –40 °C for 10 min before addition of the electrophile (15.0 mmol). The reaction mixture was then stirred at –40 °C for 30 min and at rt for 17 h before addition of 10% HCl (50 mL). The aqueous phase was extracted with EtOAc (3 × 50 mL) and the combined organics were dried over $MgSO_4$, filtered, and concentrated *in vacuo*. Purification by flash column chromatography, eluting with EtOAc/Hexane, afforded the carboxylic acid, which was used directly in the next step.

Step B: To a solution of the carboxylic acid (3.14 mmol) in DMSO (12 mL) was added K_2CO_3 (1.30 g, 9.42 mmol) and EtI (0.76 mL, 9.42 mmol). The reaction mixture was heated at 50 °C for 3 h, then cooled to rt and partitioned between H_2O (35 mL) and EtOAc (3 × 35 mL). The combined organics were dried over $MgSO_4$, filtered, and concentrated *in vacuo*. Purification by flash column chromatography, eluting with EtOAc/Hexane, afforded the title compound.

Ethyl 2-(2-[ethyl(phenyl)amino]phenyl)acetate 14a

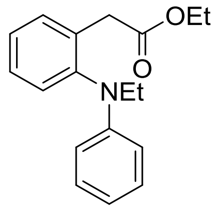

1-Phenylindolin-2-one **11a** (3.48 g, 16.6 mmol), NaOH (0.730 g, 18.3 mmol), *n*-BuLi (2.45 M in Hexane, 7.47 mL, 18.3 mmol) and EtI (4.00 mL, 49.8 mmol) in THF (8.3 mL + 16.6 mL) were subjected to General Procedure 2 (Step A). Purification by flash column chromatography, eluting with EtOAc/Hexane (1:9 to 1:4), afforded the carboxylic acid as a pale orange oil (2.11 g, 50%).

To a solution of the carboxylic acid (2.11 g, 8.30 mmol) in EtOH (33 mL) was added conc. H_2SO_4 (0.8 mL). The reaction mixture was heated at reflux for 3 h, then cooled to rt and partitioned between H_2O (100 mL) and Et_2O (3 × 100 mL). The combined organics were washed with saturated $NaHCO_3$ (100 mL), dried over $MgSO_4$, filtered, and concentrated *in vacuo*. Purification by flash column chromatography, eluting with EtOAc/Hexane (1:19 to 1:9), afforded the title compound as a colourless oil (1.93 g, 82%).

[4] P. C. Fuenfschilling, W. Zaugg, U. Beutler, D. Kaufmann, O. Lohse, J.-P. Mutz, U. Onken, J.-L. Reber and D. Shenton *Org. Process Res. Dev.* **2005**, *9*, 272-277.

1H NMR (400 MHz; $CDCl_3$): δ 7.43 (dd, *J* = 7.6 and 1.5 Hz, 1 H), 7.36 (td, *J* = 7.6 and 1.5 Hz, 1 H), 7.31 (dd, *J* = 7.6 and 1.5 Hz, 1 H), 7.19 (dd, *J* = 7.6 and 1.5 Hz, 1 H), 7.17-7.13 (m, 2 H), 6.70 (t, *J* = 7.6 Hz, 1 H), 6.52 (d, *J* = 7.6 Hz, 2 H), 4.03 (q, *J* = 6.9 Hz, 2 H), 3.65 (q, *J* = 6.9 Hz, 2 H), 3.55 (s, 2 H), 1.24 (t, *J* = 6.9 Hz, 3 H), 1.17 (t, *J* = 6.9 Hz, 3 H).

13C NMR (100 MHz; $CDCl_3$): δ 171.7 (C), 148.3 (C), 145.5 (C), 134.3 (C), 131.6 (CH), 130.0 (CH), 129.1 (CH), 129.0 (CH), 127.0 (CH), 117.1 (CH), 113.4 (CH), 60.8 (CH_2), 45.9 (CH_2), 37.0 (CH_2), 14.2 (Me), 12.5 (Me).

IR (ATR, cm^{-1}): 2977, 2939, 1732, 1594, 1576, 1493, 1448, 1368, 1333, 1299, 1266, 1244, 1206, 1153, 1116, 1090, 1030.

HRMS (ESI): Found MH^+, 284.1635. $C_{18}H_{22}NO_2^+$ requires 284.1645. Δ = 3.5 ppm.

Ethyl 2-(2-[benzyl(phenyl)amino]phenyl)acetate 14b

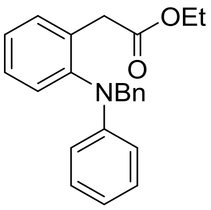

1-Phenylindolin-2-one **11a** (0.837 g, 4.00 mmol), NaOH (0.176 g, 4.40 mmol), *n*-BuLi (2.45 M in Hexane, 1.80 mL, 4.40 mmol) and BnBr (1.43 mL, 12.0 mmol) in THF (2 mL + 4 mL) were subjected to General Procedure 2 (Step A). Purification by flash column chromatography, eluting with EtOAc/Hexane (1:9), afforded the carboxylic acid as a colourless semi-solid (0.996 g, 78%).

The carboxylic acid (0.996 g, 3.14 mmol), K_2CO_3 (1.30 g, 9.42 mmol) and EtI (0.76 mL, 9.42 mmol) in DMSO (12 mL) were subjected to General Procedure 2 (Step B). Purification by flash column chromatography, eluting with EtOAc/Hexane (1:19 to 1:9), afforded the title compound as a colourless oil (1.93 g, 82%).

1H NMR (400 MHz; $CDCl_3$): δ 7.40 (dd, *J* = 7.3 and 2.1 Hz, 1 H), 7.35-7.21 (m, 8 H), 7.12 (d, *J* = 7.4 Hz, 1 H), 7.10 (d, *J* = 7.4 Hz, 1 H), 6.71 (t, *J* = 7.2 Hz, 1 H), 6.53 (d, *J* = 8.3 Hz, 2 H), 4.84 (s, 2 H), 3.99 (q, *J* = 7.2 Hz, 2 H), 3.55 (s, 2 H), 1.15 (t, *J* = 7.2 Hz, 3 H).

13C NMR (100 MHz; $CDCl_3$): δ 171.4 (C), 148.4 (C), 146.4 (C), 138.7 (C), 133.5 (C), 131.7 (CH), 129.4 (CH), 129.0 (CH), 128.9 (CH), 128.4 (CH), 126.92 (CH), 126.91 (CH), 126.7 (CH), 117.6 (CH), 114.0 (CH), 60.7 (CH_2), 56.2 (CH_2), 37.1 (CH_2), 14.0 (Me).

IR (ATR, cm^{-1}): 3061, 3027, 2980, 1731, 1594, 1576, 1493, 1452, 1367, 1350, 1331, 1297, 1249, 1206, 1153, 1115, 1063, 1028, 1004.

HRMS (ESI): Found MH^+, 346.1801. $C_{23}H_{24}NO_2^+$ requires 346.1802. Δ = 0.3 ppm.

Ethyl 2-(2-[ethyl(4-fluorophenyl)amino]phenyl)acetate 14c

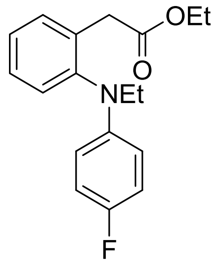

1-(4-Fluorophenyl)indolin-2-one **11b** (1.36 g, 6.00 mmol), NaOH (0.264 g, 6.60 mmol), *n*-BuLi (2.45 M in Hexane, 2.69 mL, 6.60 mmol) and EtI (1.45 mL, 18.0 mmol) in THF (3 mL + 6 mL) were subjected to General Procedure 2 (Step A). Purification by flash column chromatography, eluting with EtOAc/Hexane (1:9), afforded the carboxylic acid as a brown oil (0.934 g, 57%).

The carboxylic acid (0.934 g, 3.42 mmol), K_2CO_3 (1.42 g, 10.3 mmol) and EtI (0.83 mL, 10.3 mmol) in DMSO (12 mL) were subjected to General Procedure 2 (Step B). Purification by flash column chromatography, eluting with EtOAc/Hexane (1:19), afforded the title compound as a colourless oil (0.894 g, 87%).

1H NMR (400 MHz; $CDCl_3$): δ 7.41 (dd, *J* = 7.4 and 1.6 Hz, 1 H), 7.34 (td, *J* = 7.5 and 1.7 Hz, 1 H), 7.28 (td, *J* = 7.4 and 1.4 Hz, 1 H), 7.15 (dd, *J* = 7.7 and 1.3 Hz, 1 H), 6.88-6.82 (m, 2 H), 6.49-6.43 (m, 2 H), 4.02 (q, *J* = 7.3 Hz, 2 H), 3.61 (q, *J* = 7.0 Hz, 2 H), 3.54 (s, 2 H), 1.21 (t, *J* = 7.3 Hz, 3 H), 1.17 (t, *J* = 7.0 Hz, 3 H).

13C NMR (100 MHz; $CDCl_3$): δ 171.5 (C), 155.6 (d, *J* = 235 Hz, C), 145.7 (C), 144.7 (C), 133.9 (C), 131.6 (CH), 129.4 (CH), 128.9 (CH), 126.7 (CH), 115.3 (d, *J* = 22.5 Hz, CH), 114.7 (d, *J* = 7.0 Hz, CH), 60.7 (CH_2), 46.2 (CH_2), 36.9 (CH_2), 14.0 (Me), 12.3 (Me).

IR (ATR, cm^{-1}): 2978, 2935, 1732, 1505, 1493, 1451, 1368, 1332, 1223, 1155, 1115, 1029.

HRMS (ESI): Found MH^+, 302.1542. $C_{18}H_{21}FNO_2^+$ requires 302.1551. Δ = 2.8 ppm.

Ethyl 2-(2-[ethyl(3,5-dichlorophenyl)amino]phenyl)acetate 14d

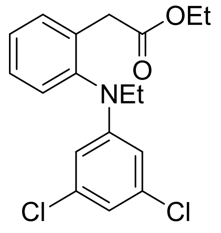

1-(3,5-Dichlorophenyl)indolin-2-one **11c** (1.39 g, 5.00 mmol), NaOH (0.220 g, 5.50 mmol), *n*-BuLi (2.45 M in Hexane, 2.24 mL, 5.50 mmol) and EtI (1.21 mL, 15.0 mmol) in THF (2.5 mL + 5 mL) were subjected to General Procedure 2 (Step A). Purification by flash column chromatography, eluting with EtOAc/Hexane (1:9), afforded the carboxylic acid as a colourless solid (0.953 g, 59%).

The carboxylic acid (0.953 g, 2.94 mmol), K_2CO_3 (1.22 g, 8.82 mmol) and EtI (0.71 mL, 8.82 mmol) in DMSO (12 mL) were subjected to General Procedure 2 (Step B). Purification by flash column

chromatography, eluting with EtOAc/Hexane (1:19), afforded the title compound as a colourless oil (0.977 g, 94%).

1H NMR (400 MHz; $CDCl_3$): δ 7.45 (dd, *J* = 6.9 and 2.4 Hz, 1 H), 7.38 (td, *J* = 7.3 and 2.0 Hz, 1 H), 7.35 (dd, *J* = 7.4 and 1.8 Hz, 1 H), 7.13 (dd, *J* = 7.2 and 2.0 Hz, 1 H), 6.66 (t, *J* = 1.8 Hz, 1 H), 6.32 (t, *J* = 1.8 Hz, 2 H), 4.03 (q, *J* = 7.0 Hz, 2 H), 3.58 (br q, *J* = 6.8 Hz, 2 H), 3.49 (s, 2 H), 1.21 (t, *J* = 7.0 Hz, 3 H), 1.17 (t, *J* = 6.8 Hz, 3 H).

13C NMR (100 MHz; $CDCl_3$): δ 171.0 (C), 149.6 (C), 143.5 (C), 135.3 (C), 133.7 (C), 132.0 (CH), 129.7 (CH), 129.2 (CH), 127.8 (CH), 116.5 (CH), 111.1 (CH), 60.9 (CH_2), 45.9 (CH_2), 36.8 (CH_2), 14.0 (Me), 12.0 (Me).

IR (ATR, cm^{-1}): 2978, 2935, 1732, 1585, 1576, 1554, 1494, 1452, 1367, 1332, 1297, 1275, 1234, 1208, 1154, 1120, 1095, 1085, 1066, 1029, 982.

HRMS (ESI): Found MH^+, 352.0863. $C_{18}H_{20}Cl_2NO_2^+$ requires 352.0866. Δ = 0.9 ppm.

Ethyl 2-(2-[ethyl(3,5-dimethylphenyl)amino]phenyl)acetate 14e

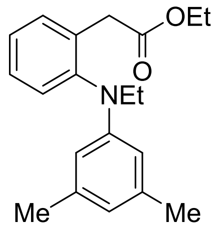

1-(3,5-Dimethylphenyl)indolin-2-one **11d** (1.19 g, 5.00 mmol), NaOH (0.220 g, 5.50 mmol), *n*-BuLi (2.45 M in Hexane, 2.24 mL, 5.50 mmol) and EtI (1.21 mL, 15.0 mmol) in THF (2.5 mL + 5 mL) were subjected to General Procedure 2 (Step A). Purification by flash column chromatography, eluting with EtOAc/Hexane (1:9), afforded the carboxylic acid as an orange oil (0.692 g, 49%).

The carboxylic acid (0.692 g, 2.44 mmol), K_2CO_3 (1.01 g, 7.32 mmol) and EtI (0.59 mL, 7.32 mmol) in DMSO (10 mL) were subjected to General Procedure 2 (Step B). Purification by flash column chromatography, eluting with EtOAc/Hexane (1:19), afforded the title compound as an orange oil (0.396 g, 52%).

1H NMR (400 MHz; $CDCl_3$): δ 7.41 (dd, *J* = 7.6 and 1.8 Hz, 1 H), 7.34 (td, *J* = 7.4 and 1.9 Hz, 1 H), 7.28 (td, *J* = 7.4 and 1.4 Hz, 1 H), 7.16 (dd, *J* = 7.6 and 1.4 Hz, 1 H), 6.36 (s, 1 H), 6.13 (s, 2 H), 4.03 (q, *J* = 7.0 Hz, 2 H), 3.61 (q, *J* = 7.0 Hz, 2 H), 3.53 (s, 2 H), 2.19 (s, 6 H), 1.21 (t, *J* = 7.0 Hz, 3 H), 1.17 (t, *J* = 7.0 Hz, 3 H).

13C NMR (100 MHz; $CDCl_3$): δ 171.7 (C), 148.2 (C), 145.5 (C), 138.5 (C), 134.0 (C), 131.3 (CH), 129.8 (CH), 128.8 (CH), 126.6 (CH), 119.0 (CH), 111.2 (CH), 60.6 (CH_2), 45.9 (CH_2), 36.8 (CH_2), 21.7 (Me), 14.1 (Me), 12.5 (Me).

IR (ATR, cm^{-1}): 2976, 2919, 1733, 1594, 1492, 1474, 1450, 1368, 1332, 1303, 1246, 1203, 1153, 1089, 1030.

HRMS (ESI): Found MH^+, 312.1960. $C_{20}H_{26}NO_2^+$ requires 312.1958. Δ = –0.6 ppm.

Ethyl 2-(4-chloro-2-[ethyl(4-methoxyphenyl)amino]phenyl)acetate 14f

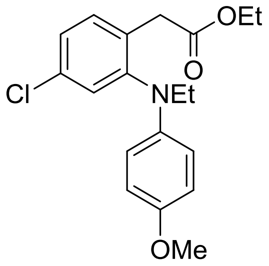

6-Chloro-1-(4-methoxyphenyl)indolin-2-one **11e** (1.37 g, 5.00 mmol), NaOH (0.220 g, 5.50 mmol), *n*-BuLi (2.45 M in Hexane, 2.24 mL, 5.50 mmol) and EtI (1.21 mL, 15.0 mmol) in THF (2.5 mL + 5 mL) were subjected to General Procedure 2 (Step A). Purification by flash column chromatography, eluting with EtOAc/Hexane (1:9), afforded the carboxylic acid as a colourless oil (0.432 g, 27%).

The carboxylic acid (0.432 g, 1.35 mmol), K_2CO_3 (0.560 g, 4.05 mmol) and EtI (0.33 mL, 4.05 mmol) in DMSO 5.4 mL) were subjected to General Procedure 2 (Step B). Purification by flash column chromatography, eluting with EtOAc/Hexane (1:9), afforded the title compound as a colourless oil (0.404 g, 86%).

1H NMR (400 MHz; $CDCl_3$): δ 7.28 (d, *J* = 8.2 Hz, 1 H), 7.19 (dd, *J* = 8.2 and 2.2 Hz, 1 H), 7.15 (d, *J* = 2.2 Hz, 1 H), 6.78-6.74 (m, 2 H), 6.58-6.54 (m, 2 H), 4.03 (q, *J* = 7.2 Hz, 2 H), 3.74 (s, 3 H), 3.57 (q, *J* = 7.1 Hz, 2 H), 3.48 (s, 2 H), 1.19 (t, *J* = 7.1 Hz, 3 H), 1.17 (t, *J* = 7.2 Hz, 3 H).

13C NMR (100 MHz; $CDCl_3$): δ 171.3 (C), 152.7 (C), 148.0 (C), 142.2 (C), 133.6 (C), 132.5 (CH), 131.8 (C), 128.3 (CH), 125.9 (CH), 117.1 (CH), 114.5 (CH), 60.8 (CH_2), 55.6 (Me), 46.9 (CH_2), 36.6 (CH_2), 14.1 (Me), 12.8 (Me).

IR (ATR, cm^{-1}): 2978, 2934, 2832, 1732, 1591, 1568, 1507, 1488, 1465, 1443, 1367, 1331, 1289, 1239, 1207, 1154, 1113, 1094, 1031.

HRMS (ESI): Found MH^+, 348.1364. $C_{19}H_{23}ClNO_3^+$ requires 348.1361. Δ = –1.0 ppm.

Ethyl 2-(2-phenoxyphenyl)acetate S1

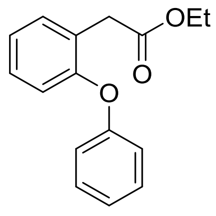

A solution of 2-(2-iodophenyl)acetate (0.928 g, 3.20 mmol), phenol (0.361 g, 3.84 mmol), CuCl (0.127 g, 1.28 mmol), *N*,*N*-dimethylglycine (66.0 mg, 0.640 mmol), and Cs_2CO_3 (2.09 g, 6.40 mmol) in dioxane (13 mL) was heated at reflux for 4 d. After cooling to rt, 10% HCl (30 mL) was added and the aqueous phase extracted with EtOAc (3 × 30 mL). The combined organics were dried over $MgSO_4$, filtered, and concentrated *in vacuo*. Purification by flash column chromatography, eluting with EtOAc/Hexane (1:19), afforded the title compound as a colourless oil (0.345 g, 42%).

1H NMR (400 MHz; CDCl$_3$): δ 7.34-7.29 (m, 3 H), 7.25 (td, *J* = 7.7 and 1.7 Hz, 1 H), 7.13-7.06 (m, 2 H), 6.99-6.95 (m, 2 H), 6.89 (dd, *J* = 8.2 and 1.0 Hz, 1 H), 4.09 (q, *J* = 7.3 Hz, 2 H), 3.69 (s, 2 H), 1.19 (t, *J* = 7.3 Hz, 3 H).

13C NMR (100 MHz; CDCl$_3$): δ 171.5 (C), 157.5 (C), 155.2 (C), 131.6 (CH), 129.7 (CH), 128.7 (CH), 126.2 (C), 123.8 (CH), 123.1 (CH), 119.0 (CH), 118.4 (CH), 60.9 (CH$_2$), 36.0 (CH$_2$), 14.2 (Me).

IR (ATR, cm^{-1}): 2982, 1733, 1583, 1486, 1454, 1368, 1335, 1234, 1214, 1153, 1097, 1028.

Data is consistent with literature values.[5]

2-[2-(Phenylsulfanyl)phenyl]acetic acid S2

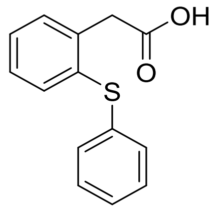

To a suspension of 2-(2-iodophenyl)acetic acid (2.62 g, 10.0 mmol), benzenethiol (1.08 mL, 10.5 mmol) and copper powder (0.190 g, 3.00 mmol) in H_2O (20 mL) was added KOH (1.68 g, 30.0 mmol). The reaction mixture was heated at reflux for 19 h. After cooling to rt, 2 M NaOH (50 mL) was added and the mixture filtered through Celite, which was washed with further 2 M NaOH (2 × 25 mL). The aqueous solution was acidified with 10% HCl and extracted with EtOAc (3 × 50 mL). The combined organics were dried over $MgSO_4$, filtered, and concentrated *in vacuo* to afford the title compound as a colourless solid (2.35 g, 96%).

MP: 105-107 °C.

1H NMR (400 MHz; CDCl$_3$): δ 10.95 (s, 1 H), 7.45 (dd, *J* = 7.1 and 1.2 Hz, 1 H), 7.37-7.17 (m, 8 H), 3.90 (s, 2 H).

13C NMR (100 MHz; CDCl$_3$): δ 177.9 (C), 136.5 (C), 136.2 (C), 134.7 (CH), 131.4 (CH), 129.6 (CH), 129.3 (CH), 128.72 (CH), 128.67 (CH), 126.7 (CH), 39.6 (CH$_2$).

IR (ATR, cm^{-1}): 3019, 2919, 2736, 2631, 2560, 1698, 1580, 1478, 1439, 1406, 1337, 1279, 1235, 1059, 1026, 933.

Data is consistent with literature values.[6]

---

[5] S. Mashayekh, N. Rahmanipour, B. Mahmoodi, F. Ahmadi, D. Motaharian, S. Shahhosseini, H. Shafaroodi, H. R. Banafshe, A. Shafiee and L. Navidpour *Bioorg. Med. Chem.* **2014**, *22*, 1929-1937.
[6] M. Botta, E. Castiglioni, R. Di Fabio, R. Spinosa and A. Togninelli WO2009/016085, **2009**.

Ethyl 2-[2-(phenylsulfanyl)phenyl]acetate S3

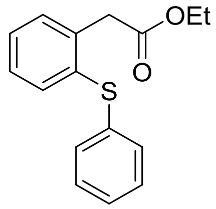

To a solution of 2-[2-(phenylsulfanyl)phenyl]acetic acid **S2** (2.35 g, 9.62 mmol) in EtOH (30 mL) was added conc. HCl (20 mL). The reaction mixture was heated at reflux for 2.5 h, then cooled to rt and partitioned between H_2O (50 mL) and EtOAc (3 × 100 mL). The combined organics were washed with saturated $NaHCO_3$ (2 × 50 mL) and saturated brine (50 mL), dried over $MgSO_4$, filtered, and concentrated *in vacuo*. Purification by flash column chromatography, eluting with EtOAc/Hexane (1:9), afforded the title compound as a colourless oil (2.01 g, 77%).

1H NMR (400 MHz; $CDCl_3$): δ 7.41 (d, *J* = 7.6 Hz, 1 H), 7.35-7.28 (m, 2 H), 7.26-7.23 (m, 3 H), 7.19-7.15 (m, 3 H), 4.09 (q, *J* = 7.0 Hz, 2 H), 3.83 (s, 2 H), 1.21 (t, *J* = 7.0 Hz, 3 H).

13C NMR (100 MHz; $CDCl_3$): δ 171.1 (C), 136.9 (C), 136.6 (C), 134.5 (CH), 134.2 (C), 131.0 (CH), 129.2 (CH), 129.0 (CH), 128.4 (CH), 128.2 (CH), 126.2 (CH), 60.8 (CH_2), 39.7 (CH_2), 14.1 (Me).

IR (ATR, cm^{-1}): 3059, 2981, 1731, 1582, 1475, 1439, 1367, 1332, 1245, 1212, 1155, 1095, 1059, 1024.

HRMS (ESI): Found MNa^+, 295.0767. $C_{16}H_{16}NaO_2S^+$ requires 295.0763. Δ = –1.2 ppm.

General Procedure 3 – Acylation of Ethyl Phenylacetates

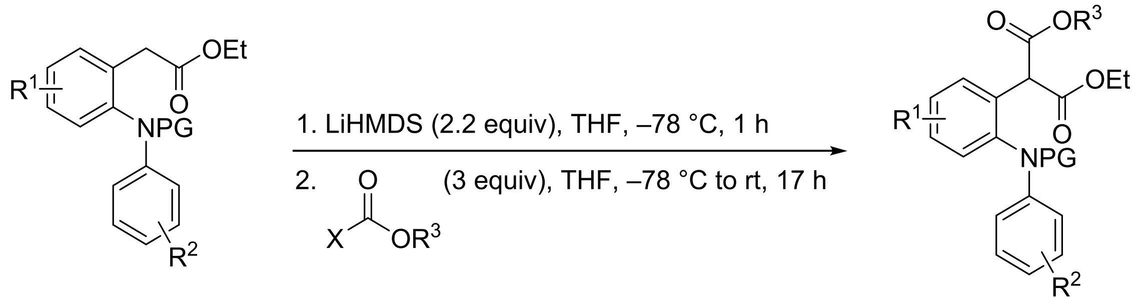

To a solution of the ethyl phenylacetate (2.00 mmol) in THF (20 mL) at –78 °C was added LiHMDS (1.00 M in THF, 4.40 mL, 4.40 mmol) dropwise over 10 min. Stirring was continued at –78 °C for 1 h, then the electrophile (6.00 mmol) added dropwise over 5 min. The reaction mixture was stirred at –78 °C for a further 30 min, then at rt for 17 h. Saturated NH_4Cl (35 mL) was added and the aqueous phase extracted with EtOAc (3 × 35 mL). The combined organics were dried over $MgSO_4$, filtered, and concentrated *in vacuo*. Purification by flash column chromatography, eluting with EtOAc/Hexane, afforded the title compound.

1,2-Diethyl 2-(2-[ethyl(phenyl)amino]phenyl)propanedioate 15a

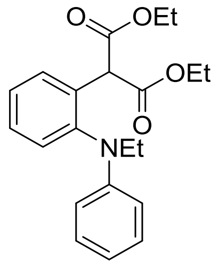

Ethyl 2-(2-[ethyl(phenyl)amino]phenyl)acetate **14a** (0.567 g, 2.00 mmol), LiHMDS (1.00 M in THF, 4.40 mL, 4.40 mmol) and ethyl cyanoformate (0.59 mL, 6.00 mmol) in THF (20 mL) were subjected to General Procedure 3. Purification by flash column chromatography, eluting with EtOAc/Hexane (1:19), afforded the title compound as a colourless oil (0.711 g, quant.).

[The reaction may also be conducted in the same manner except using ethyl chloroformate as the electrophile. In this case the yield is lowered to 79%]

1H NMR (400 MHz; $CDCl_3$): δ 7.67 (dd, J = 7.6 and 1.5 Hz, 1 H), 7.42 (td, J = 7.6 and 2.3 Hz, 1 H), 7.37 (td, J = 6.9 and 1.5 Hz, 1 H), 7.20 (dd, J = 7.6 and 1.5 Hz, 1 H), 7.15 (dd, J = 8.4 and 6.9 Hz, 2 H), 6.72 (t, J = 6.9 Hz, 1 H), 6.54 (d, J = 9.2 Hz, 2 H), 5.05 (s, 1 H), 4.21-4.08 (m, 4 H), 3.67 (q, J = 6.9 Hz, 2 H), 1.25 (t, J = 6.9 Hz, 3 H), 1.20 (t, J = 6.9 Hz, 6 H).

13C NMR (100 MHz; $CDCl_3$): δ 168.4 (C), 148.5 (C), 145.5 (C), 133.0 (C), 130.6 (CH), 129.9 (CH), 129.6 (CH), 128.9 (CH), 127.0 (CH), 117.3 (CH), 113.7 (CH), 61.6 (CH_2), 51.9 (CH), 46.3 (CH_2), 13.9 (Me), 12.4 (Me).

IR (ATR, cm^{-1}): 2979, 2937, 1750, 1731, 1594, 1576, 1495, 1447, 1368, 1349, 1301, 1246, 1214, 1188, 1144, 1096, 1029.

HRMS (ESI): Found MH^+, 356.1853. $C_{21}H_{26}NO_4^+$ requires 356.1856. Δ = 0.9 ppm.

1,2-Diethyl 2-(2-[benzyl(phenyl)amino]phenyl)propanedioate 15b

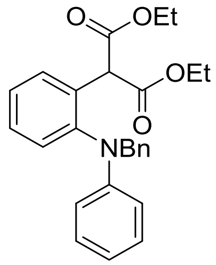

Ethyl 2-(2-[benzyl(phenyl)amino]phenyl)acetate **14b** (0.691 g, 2.00 mmol), LiHMDS (1.00 M in THF, 4.40 mL, 4.40 mmol) and ethyl cyanoformate (0.59 mL, 6.00 mmol) in THF (20 mL) were subjected to General Procedure 3. Purification by flash column chromatography, eluting with EtOAc/Hexane (1:19), afforded the title compound as a colourless oil (0.821 g, 98%).

1H NMR (400 MHz; $CDCl_3$): δ 7.67-7.63 (m, 1 H), 7.40-7.23 (m, 8 H), 7.15-7.10 (m, 2 H), 6.75 (t, *J* = 7.3 Hz, 1 H), 6.58 (d, *J* = 7.7 Hz, 2 H), 5.06 (s, 1 H), 4.86 (s, 2 H), 4.18-4.03 (m, 4 H), 1.18 (t, *J* = 7.2 Hz, 3 H), 1.17 (t, *J* = 7.0 Hz, 3 H).

13C NMR (100 MHz; $CDCl_3$): δ 168.4 (C), 149.1 (C), 146.7 (C), 138.6 (C), 132.5 (C), 131.0 (CH), 130.1 (CH), 129.3 (CH), 129.1 (CH), 128.6 (CH), 127.3 (CH), 127.2 (CH), 127.0 (CH), 118.3 (CH), 114.8 (CH), 61.8 (CH_2), 56.9 (CH_2), 52.3 (CH), 14.0 (Me).

IR (ATR, cm^{-1}): 3062, 2983, 1745, 1724, 1592, 1488, 1452, 1365, 1312, 1265, 1249, 1212, 1146, 1112, 1080, 1027.

HRMS (ESI): Found MH^+, 418.2002. $C_{26}H_{28}NO_4^+$ requires 418.2013. Δ = 2.7 ppm.

1-Benzyl-3-ethyl 2-(2-[ethyl(phenyl)amino]phenyl)propanedioate 15c

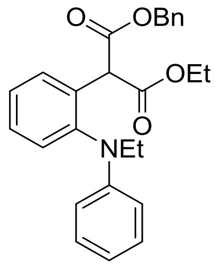

Ethyl 2-(2-[ethyl(phenyl)amino]phenyl)acetate **14a** (0.283 g, 1.00 mmol), LiHMDS (1.00 M in THF, 2.20 mL, 2.20 mmol) and benzyl chloroformate (0.43 mL, 3.00 mmol) in THF (10 mL) were subjected to General Procedure 3. Purification by flash column chromatography, eluting with EtOAc/Hexane (1:19), afforded the title compound as a colourless oil (0.311 g, 74%).

1H NMR (400 MHz; $CDCl_3$): δ 7.65 (dd, *J* = 7.6 and 1.0 Hz, 1 H), 7.44-7.30 (m, 5 H), 7.24-7.22 (m, 2 H), 7.18 (dd, *J* = 7.8 and 1.1 Hz, 1 H), 7.13-7.09 (m, 2 H), 6.70 (td, *J* = 7.3 and 0.7 Hz, 1 H), 6.51 (d, *J* = 8.6 Hz,

2 H), 5.12 (s, 1 H), 5.10 (s, 2 H), 4.18-4.05 (m, 2 H), 3.62 (q, *J* = 7.2 Hz, 2 H), 1.19 (t, *J* = 7.3 Hz, 3 H), 1.15 (t, *J* = 7.2 Hz, 3 H).

13C NMR (100 MHz; $CDCl_3$): δ 168.3 (C), 148.5 (C), 145.5 (C), 135.3 (C), 132.9 (C), 130.6 (CH), 129.9 (CH), 129.7 (CH), 128.9 (CH), 128.4 (CH), 128.2 (CH), 128.1 (CH), 127.0 (CH), 117.4 (CH), 113.7 (CH), 67.2 (CH_2), 61.7 (CH_2), 51.9 (CH), 46.2 (CH_2), 13.8 (Me), 12.4 (Me).

IR (ATR, cm^{-1}): 3034, 2977, 1750, 1731, 1594, 1576, 1495, 1448, 1370, 1350, 1300, 1249, 1212, 1141, 1082, 1028.

HRMS (ESI): Found MH^+, 418.2014. $C_{26}H_{28}NO_4^+$ requires 418.2013. Δ = –0.2 ppm.

1-Allyl-3-ethyl 2-(2-[ethyl(phenyl)amino]phenyl)propanedioate 15d

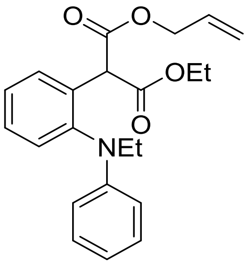

Ethyl 2-(2-[ethyl(phenyl)amino]phenyl)acetate **14a** (0.283 g, 1.00 mmol), LiHMDS (1.00 M in THF, 2.20 mL, 2.20 mmol) and allyl chloroformate (0.32 mL, 3.00 mmol) in THF (10 mL) were subjected to General Procedure 3. Purification by flash column chromatography, eluting with EtOAc/Hexane (1:19), afforded the title compound as a colourless oil (0.324 g, 88%).

1H NMR (400 MHz; $CDCl_3$): δ 7.64 (dd, *J* = 7.6 and 1.8 Hz, 1 H), 7.41 (td, *J* = 7.4 and 1.9 Hz, 1 H), 7.36 (td, *J* = 7.4 and 1.5 Hz, 1 H), 7.18 (dd, *J* = 7.6 and 1.5 Hz, 1 H), 7.15-7.10 (m, 2 H), 6.70 (tt, *J* = 7.3 and 1.0 Hz, 1 H), 6.53-6.50 (m, 2 H), 5.85-5.75 (m, 1 H), 5.25-5.16 (m, 2 H), 5.06 (s, 1 H), 4.61-4.50 (m, 2 H), 4.19-4.05 (m, 2 H), 3.65 (q, *J* = 7.1 Hz, 2 H), 1.22 (t, J = 7.1 Hz, 3 H), 1.18 (t, *J* = 7.1 Hz, 3 H).

13C NMR (100 MHz; $CDCl_3$): δ 168.3 (C), 168.1 (C), 148.5 (C), 145.5 (C), 132.9 (C), 131.4 (CH), 130.6 (CH), 129.9(CH), 129.7 (CH), 128.9 (CH), 127.0 (CH), 118.4 (CH_2), 117.4 (CH), 113.7 (CH), 66.1 (CH_2), 61.7 (CH_2), 51.9 (CH), 46.3 (CH_2), 13.9 (Me), 12.4 (Me).

IR (ATR, cm^{-1}): 2979, 1751, 1732, 1594, 1576, 1494, 1448, 1368, 1350, 1302, 1245, 1211, 1188, 1141, 1062, 1029, 990.

HRMS (ESI): Found MH^+, 368.1841. $C_{22}H_{26}NO_4^+$ requires 368.1856. Δ = 4.1 ppm.

1-Ethyl-3-(9*H*-fluoren-9-ylmethyl) 2-(2-[ethyl(phenyl)amino]phenyl)propanedioate 15e

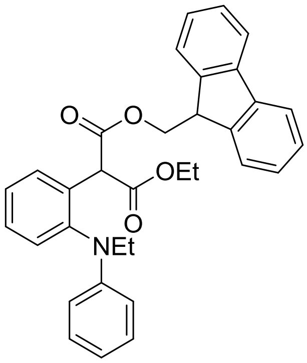

Ethyl 2-(2-[ethyl(phenyl)amino]phenyl)acetate **14a** (0.283 g, 1.00 mmol), LiHMDS (1.00 M in THF, 2.20 mL, 2.20 mmol) and Fmoc-Cl (0.776 g, 3.00 mmol) in THF (10 mL + 3 mL) were subjected to General Procedure 3. Purification by flash column chromatography, eluting with EtOAc/Hexane (1:19), afforded the title compound as a colourless oil (0.174 g, 34%).

1H NMR (400 MHz; $CDCl_3$): δ 7.73 (d, *J* = 8.0 Hz, 2 H), 7.56 (dd, *J* = 7.8 and 1.5 Hz, 1 H), 7.43 (td, *J* = 7.5 and 1.7 Hz, 1 H), 7.40-7.32 (m, 5 H), 7.26-7.19 (m, 3 H), 7.13-7.08 (m, 2 H), 6.68 (t, *J* = 7.0 Hz, 1 H), 6.53 (d, *J* = 7.9 Hz, 2 H), 5.11 (s, 1 H), 4.43 (dd, *J* = 10.7 and 6.8 Hz, 1 H), 4.32 (dd, *J* = 10.7 and 7.3 Hz, 1 H), 4.18-4.05 (m, 3 H), 3.68-3.55 (m, 2 H), 1.21 (t, *J* = 7.0 Hz, 3 H), 1.17 (t, *J* = 7.0 Hz, 3 H).

13C NMR (100 MHz; $CDCl_3$): δ 168.4 (C), 168.2 (C), 148.5 (C), 145.6 (C), 143.44 (C), 143.37 (C), 141.21(C), 141.18 (C), 132.8 (C), 130.7 (CH), 129.9 (CH), 129.7 (CH), 129.0 (CH), 127.72 (CH), 127.71 (CH), 127.2 (CH), 127.1 (CH), 127.0 (CH), 125.0 (CH), 124.9 (CH), 119.93 (CH), 119.92 (CH), 117.5 (CH), 113.8 (CH), 67.5 (CH_2), 61.8 (CH_2), 52.1 (CH), 46.5 (CH), 46.3 (CH_2), 13.9 (Me), 12.4 (Me).

IR (ATR, cm^{-1}): 2977, 1731, 1594, 1576, 1494, 1449, 1370, 1349, 1300, 1247, 1212, 1189, 1142, 1029.

HRMS (ESI): Found MH^+, 506.2336. $C_{33}H_{32}NO_4^+$ requires 506.2326. Δ = –2.1 ppm.

1-Ethyl-3-(*L*-menthyl) 2-(2-[ethyl(phenyl)amino]phenyl)propanedioate 15f

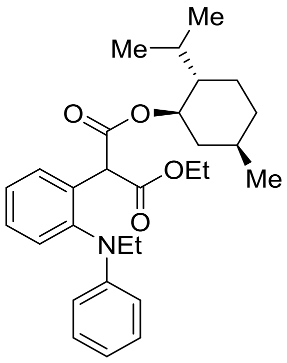

Ethyl 2-(2-[ethyl(phenyl)amino]phenyl)acetate **14a** (0.283 g, 1.00 mmol), LiHMDS (1.00 M in THF, 2.20 mL, 2.20 mmol) and *L*-menthyl chloroformate (0.64 mL, 3.00 mmol) in THF (10 mL) were subjected to General Procedure 3. Purification by flash column chromatography, eluting with EtOAc/Hexane (1:19),

afforded the title compound as a colourless oil (0.294 g, 63%) as an inseparable 1:1 mixture of diastereomers.

1H NMR (400 MHz; $CDCl_3$): δ 7.65 (2 × t, *J* = 7.5 Hz, 1 H), 7.41-7.32 (m, 2 H), 7.17-7.10 (m, 3 H), 6.71-6.67 (m, 1 H), 6.53-6.50 (m, 2 H), 5.01 (s, 1 H), 4.71-4.63 (m, 1 H), 4.20-4.03 (m, 2 H), 3.63 (2 × q, *J* = 6.9 Hz, 2 H), 2.05-1.99 (m, 0.5 H), 1.88-1.80 (m, 1 H), 1.68-1.59 (m, 2 H), 1.56-1.37 (m, 1.5 H), 1.33-1.26 (m, 1 H), 1.21 (2 × t, *J* = 7.0 Hz, 3 H), 1.18 (2 × t, *J* = 7.0 Hz, 3 H), 1.06-0.93 (m, 1.5 H), 0.89 (d, *J* = 6.4 Hz, 1.5 H), 0.86-0.78 (m, 2 H), 0.85 (dd, *J* = 6.7 and 6.5 Hz, 3 H), 0.74 (dd, *J* = 7.9 and 7.1 Hz, 3 H), 0.62 (d, *J* = 7.1 Hz, 1.5 H).

13C NMR (100 MHz; $CDCl_3$): δ 168.43 and 168.40 (C), 168.1 and 168.0 (C), 148.6 and 148.5 (C), 145.51 and 145.48 (C), 133.3 and 133.1 (C), 130.8 and 130.6 (CH), 129.8 (CH), 129.64 and 129.61 (CH), 128.93 and 128.92 (CH), 127.0 and 126.9 (CH), 117.3 (CH), 113.6 (CH), 75.7 and 75.6 (CH), 61.58 and 61.56 (CH_2), 52.1 and 52.0 (CH), 46.9 and 46.8 (CH), 46.23 and 46.21 (CH_2), 40.4 and 40.3 (CH_2), 34.1 (CH_2), 31.3 and 31.2 (CH), 25.7 and 25.5 (CH), 23.0 and 22.9 (CH_2), 21.93 and 21.92 (Me), 20.69 and 20.67 (Me), 15.9 and 15.8 (Me), 13.9 (Me), 12.45 and 12.40 (Me).

IR (ATR, cm^{-1}): 2955, 2870, 1749, 1730, 1594, 1577, 1496, 1449, 1369, 1299, 1257, 1215, 1187, 1144, 1096, 1031.

HRMS (ESI): Found MH^+, 466.2954. $C_{29}H_{40}NO_4^+$ requires 466.2952. Δ = –0.4 ppm.

1,2-Diethyl 2-(2-[ethyl(4-fluorophenyl)amino]phenyl)propanedioate 15g

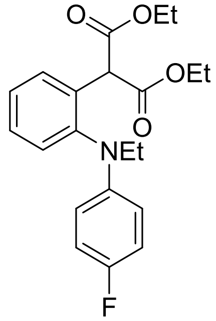

Ethyl 2-(2-[ethyl(4-fluorophenyl)amino]phenyl)acetate **14c** (0.567 g, 1.88 mmol), LiHMDS (1.00 M in THF, 4.40 mL, 4.40 mmol) and ethyl cyanoformate (0.59 mL, 6.00 mmol) in THF (20 mL) were subjected to General Procedure 3. Purification by flash column chromatography, eluting with EtOAc/Hexane (1:19), afforded the title compound as a colourless oil (0.697 g, 99%).

1H NMR (400 MHz; $CDCl_3$): δ 7.63 (dd, *J* = 7.8 and 1.8 Hz, 1 H), 7.40 (td, *J* = 7.3 and 1.8 Hz, 1 H), 7.35 (td, *J* = 7.3 and 1.4 Hz, 1 H), 7.15 (dd, *J* = 7.8 and 1.8 Hz, 1 H), 6.87-6.81 (m, 2 H), 6.49-6.44 (m, 2 H), 5.04 (s, 1 H), 4.20-4.07 (m, 4 H), 3.61 (q, *J* = 7.1 Hz, 2 H), 1.21 (t, *J* = 7.1 Hz, 3 H), 1.19 (t, *J* = 6.9 Hz, 6 H).

13C NMR (100 MHz; $CDCl_3$): δ 168.4 (C), 155.8 (d, *J* = 236 Hz, C), 145.8 (C), 145.2 (d, *J* = 1.9 Hz, C), 132.9 (C), 130.6 (CH), 129.9 (CH), 129.2 (CH), 127.0 (CH), 115.3 (d, *J* = 22.1 Hz, CH), 115.0 (d, *J* = 11.5 Hz, CH), 61.7 (CH_2), 51.9 (CH), 46.7 (CH_2), 13.9 (Me), 12.5 (Me).

IR (ATR, cm^{-1}): 2980, 1750, 1731, 1505, 1450, 1368, 1304, 1219, 1188, 1145, 1115, 1096, 1029.

HRMS (ESI): Found MH^+, 374.1750. $C_{21}H_{25}FNO_4^+$ requires 374.1762. Δ = 3.1 ppm.

1,2-Diethyl 2-(2-[ethyl(3,5-dichlorophenyl)amino]phenyl)propanedioate 15h

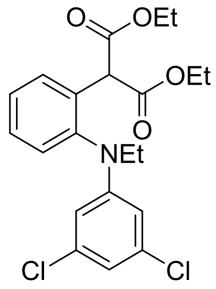

Ethyl 2-(2-[ethyl(3,5-dichlorophenyl)amino]phenyl)acetate **14d** (0.705 g, 2.00 mmol), LiHMDS (1.00 M in THF, 4.40 mL, 4.40 mmol) and ethyl cyanoformate (0.59 mL, 6.00 mmol) in THF (15 mL) were subjected to General Procedure 3. Purification by flash column chromatography, eluting with EtOAc/Hexane (1:19), afforded the title compound as a colourless oil (0.798 g, 94%).

1H NMR (400 MHz; $CDCl_3$): δ 7.68 (dd, *J* = 6.9 and 2.8 Hz, 1 H), 7.44 (td, *J* = 7.3 and 2.3 Hz, 1 H), 7.41 (td, *J* = 7.3 and 1.8 Hz, 1 H), 7.13 (dd, *J* = 6.9 and 2.3 Hz, 1 H), 6.67 (t, *J* = 3.7 Hz, 1 H), 6.33 (d, *J* = 1.4 Hz, 2H), 4.82 (s, 1 H), 4.20-4.10 (m, 4 H), 3.70-3.50 (m, 2 H), 1.21 (t, *J* = 7.3 Hz, 6 H), 1.19 (t, *J* = 7.2 Hz, 3 H).

13C NMR (100 MHz; $CDCl_3$): δ 168.1 (C), 150.0 (C), 143.6 (C), 135.4 (C), 132.7 (C), 131.0 (CH), 130.3 (CH), 129.5 (CH), 128.0 (CH), 117.0 (CH), 111.5 (CH), 61.9 (CH_2), 51.9 (CH), 46.5 (CH_2), 13.9 (Me), 12.1 (Me).

IR (ATR, cm^{-1}): 2980, 1750, 1732, 1585, 1577, 1556, 1491, 1454, 1367, 1348, 1297, 1255, 1215, 1188, 1120, 1095, 1029, 983.

HRMS (ESI): Found MH^+, 424.1071. $C_{21}H_{24}Cl_2NO_4^+$ requires 424.1077. Δ = 1.4 ppm.

1,2-Diethyl 2-(2-[ethyl(3,5-dimethylphenyl)amino]phenyl)propanedioate 15i

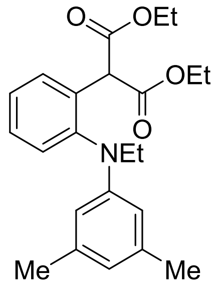

Ethyl 2-(2-[ethyl(3,5-dimethylphenyl)amino]phenyl)acetate **14e** (0.311 g, 1.00 mmol), LiHMDS (1.00 M in THF, 2.20 mL, 2.20 mmol) and ethyl cyanoformate (0.30 mL, 3.00 mmol) in THF (10 mL) were subjected to General Procedure 3. Purification by flash column chromatography, eluting with EtOAc/Hexane (1:19), afforded the title compound as a colourless oil (0.307 g, 80%).

1H NMR (400 MHz; $CDCl_3$): δ 7.64 (dd, *J* = 7.6 and 1.8 Hz, 1 H), 7.41 (td, *J* = 7.3 and 1.8 Hz, 1 H), 7.36 (td, *J* = 7.3 and 1.4 Hz, 1 H), 7.18 (dd, *J* = 7.6 and 1.6 Hz, 1 H), 6.39 (s, 1 H), 6.17 (s, 2 H), 5.02 (s, 1 H), 4.20-4.07 (m, 4 H), 3.65 (q, *J* = 7.1 Hz, 2 H), 2.20 (s, 6 H), 1.23 (t, *J* = 7.3 Hz, 3 H), 1.20 (t, *J* = 7.0 Hz, 6 H).

13C NMR (100 MHz; $CDCl_3$): δ 168.5 (C), 148.5 (C), 145.7 (C), 138.5 (C), 132.9 (C), 130.5 (CH), 129.8 (CH), 129.5 (CH), 126.8 (CH), 119.4 (CH), 111.6 (CH), 61.5 (CH_2), 51.9 (CH), 46.3 (CH_2), 21.6 (Me), 13.9 (Me), 12.6 (Me).

IR (ATR, cm^{-1}): 2979, 1751, 1732, 1594, 1490, 1474, 1448, 1367, 1349, 1302, 1247, 1205, 1144, 1095, 1029.

HRMS (ESI): Found MH^+, 384.2157. $C_{23}H_{30}NO_4^+$ requires 384.2169. Δ = 3.3 ppm.

1,2-Diethyl 2-(4-chloro-2-[ethyl(4-methoxyphenyl)amino]phenyl)propanedioate 15j

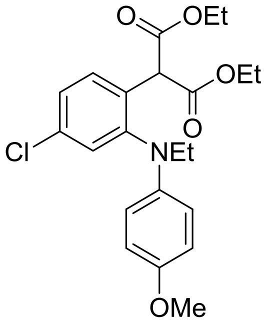

Ethyl 2-(4-chloro-2-[ethyl(4-methoxyphenyl)amino]phenyl)acetate **14f** (0.348 g, 1.00 mmol), LiHMDS (1.00 M in THF, 2.20 mL, 2.20 mmol) and ethyl cyanoformate (0.30 mL, 3.00 mmol) in THF (10 mL) were subjected to General Procedure 3. Purification by flash column chromatography, eluting with EtOAc/Hexane (1:19), afforded the title compound as a colourless oil (0.388 g, 92%).

1H NMR (400 MHz; $CDCl_3$): δ 7.52 (d, *J* = 8.7 Hz, 1 H), 7.25 (dd, *J* = 8.2 and 2.3 Hz, 1 H), 7.14 (d, *J* = 2.3 Hz, 1 H), 6.76-6.71 (m, 2 H), 6.57-6.52 (m, 2 H), 5.06 (s, 1 H), 4.17-4.04 (m, 4 H), 3.72 (s, 3 H), 3.57 (q, *J* = 6.9 Hz, 2 H), 1.18 (t, *J* = 6.9 Hz, 3 H), 1.17 (t, *J* = 6.9 Hz, 6 H).

13C NMR (100 MHz; $CDCl_3$): δ 168.2 (C), 152.8 (C), 148.0 (C), 142.6 (C), 134.8 (C), 131.6 (CH), 131.0 (C), 128.4 (CH), 126.5 (CH), 117.0 (CH), 114.5 (CH), 61.7 (CH_2), 55.5 (Me), 51.5 (CH), 47.2 (CH_2), 13.9 (Me), 12.9 (Me).

IR (ATR, cm^{-1}): 2980, 2834, 1749, 1731, 1590, 1507, 1488, 1466, 1444, 1367, 1298, 1240, 1179, 1146, 1112, 1094, 1063, 1031.

HRMS (ESI): Found MNa^+, 442.1372. $C_{22}H_{26}ClNNaO_5^+$ requires 442.1392. Δ = 4.5 ppm.

N-(4-*tert*-Butylphenyl)-2-iodopyridin-3-amine S4[7]

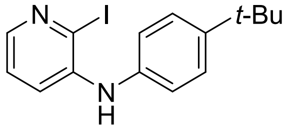

To a solution of 4-*tert*-butylcyclohex-2-en-1-one[8] (2.80 g, 18.2 mmol) and 2-iodopyridin-3-amine[9] (3.34 g, 15.2 mmol) in DMSO (36 mL) was added *p*-TsOH·H_2O (0.346 g, 1.82 mmol) and iodine (2.31 g, 9.1 mmol). The reaction mixture was heated at 90 °C for 16 h. After cooling to rt, the reaction was quenched by the addition of 10% $Na_2S_2O_3$ (100 mL), and the aqueous phase extracted with CH_2Cl_2 (3 × 100 mL). The combined organics were washed with 10% $Na_2S_2O_3$ (100 mL), 2M NaOH (2 × 100 mL) and saturated brine (100 mL), dried over $MgSO_4$, filtered, and concentrated *in vacuo*. Purification by flash column chromatography, eluting with EtOAc/Hexane (0:1 to 1:19), afforded the title compound as a purple solid (2.59 g, 48%).

MP: 72-74 °C (EtOAc/Hexane).

1H NMR (400 MHz; $CDCl_3$): δ 7.84 (dd, *J* = 4.6 and 1.8 Hz, 1 H), 7.39-7.36 (m, 2 H), 7.26 (dd, *J* = 8.2 and 1.8 Hz, 1 H), 7.11-7.07 (m, 2 H), 7.03 (dd, *J* = 8.2 and 4.6 Hz, 1 H), 5.96 (br s, 1 H), 1.33 (s, 9 H).

13C NMR (100 MHz; $CDCl_3$): δ 147.1 (C), 142.4 (C), 141.1 (CH), 137.5 (C), 126.5 (CH), 123.3 (CH), 121.1 (CH), 119.8 (CH), 112.5 (C), 34.4 (C), 31.4 (Me).

IR (ATR, cm^{-1}): 3363, 2959, 2863, 1608, 1581, 1567, 1552, 1513, 1480, 1444, 1412, 1357, 1267, 1187, 1112, 1031.

HRMS (ESI): Found MH^+, 353.0494. $C_{15}H_{18}IN_2^+$ requires 353.509. Δ = 4.2 ppm.

N-(4-*tert*-Butylphenyl)-2-iodo-*N*-methylpyridin-3-amine S5

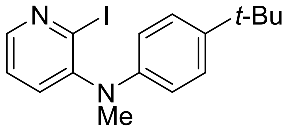

To a solution of NaH (60% in mineral oil, 0.250 g, 6.24 mmol) in THF (6.2 mL) at 0 °C was added *N*-(4-*tert*-butylphenyl)-2-iodopyridin-3-amine **S4** (1.10 g, 3.12 mmol) in THF (9.3 mL) dropwise over 5 min. Stirring was continued at 0 °C for 1.5 h, then iodomethane (0.388 mL, 6.24 mmol) was added. The reaction mixture was stirred at rt for 20 h, then partitioned between saturated NH_4Cl (100 mL) and EtOAc (3 × 100 mL). The combined organics were dried over $MgSO_4$, filtered, and concentrated *in vacuo*. Purification by flash column chromatography, eluting with EtOAc/Hexane (1:19), afforded the title compound as a brown solid (0.888 g, 78%).

MP: 62-64 °C (EtOAc/Hexane).

[7] M. T. Barros, S. S. Dey, C. D. Maycock and Paula Rodrigues *Chem. Commun.* **2012**, *48*, 10901-10903.
[8] Y. Shvo and A. H. I. Arisha *J. Org. Chem.* **1998**, *63,* 5640-5642.
[9] H.-Y. Lee, N. Yerkes and S. E. O'Connor *Chem. Biol.* **2009**, *16*, 1225-1229.

1H NMR (400 MHz; $CDCl_3$): δ 8.27 (dd, *J* = 4.6 and 1.8 Hz, 1 H), 7.46 (dd, *J* = 7.8 and 1.8 Hz, 1 H), 7.27 (dd, *J* = 7.8 and 4.6 Hz, 1 H), 7.25-7.21 (m, 2 H), 6.56-6.52 (m, 2 H), 3.21 (s, 3 H), 1.27 (s, 9 H).

13C NMR (100 MHz; $CDCl_3$): δ 148.8 (C), 148.0 (CH), 145.7 (C), 141.5 (C), 136.7 (CH), 125.9 (CH), 125.7 (C), 123.9 (CH), 113.9 (CH), 39.5 (Me), 33.9 (C), 31.4 (Me).

IR (ATR, cm^{-1}): 2955, 2867, 1610, 1563, 1548, 1512, 1471, 1444, 1389, 1367, 1333, 1313, 1286, 1267, 1231, 1201, 1143, 1126, 1109, 1083, 1039.

HRMS (ESI): Found MH^+, 367.0667. $C_{16}H_{20}IN_2^+$ requires 367.0666. Δ = –0.2 ppm.

1,3-Diethyl 2-{3-[(4-*tert*-butylphenyl)(methyl)amino]pyridine-2-yl}propanedioate 15k

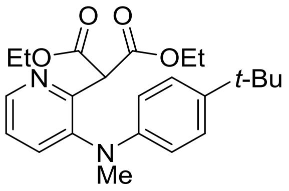

To a solution of *N*-(4-*tert*-butylphenyl)-2-iodo-*N*-methylpyridin-3-amine **S5** (0.659 g, 1.80 mmol), CuI (34.0 mg, 0.180 mmol), 2-phenylphenol (46.0 mg, 0.270 mmol) and Cs_2CO_3 (1.17 g, 3.60 mmol) in THF (3.6 mL) was added diethyl malonate (0.547 mL, 3.60 mmol). The reaction mixture was heated at reflux for 15 h. After cooling to rt, H_2O (50 mL) was added and the aqueous phase extracted with EtOAc (3 × 50 mL). The combined organics were dried over $MgSO_4$, filtered, and concentrated *in vacuo*. Purification by flash column chromatography, eluting with EtOAc/Hexane (1:9 to 1:4), afforded the title compound as a colourless oil (81.4 mg, 11%).

1H NMR (400 MHz; $CDCl_3$): δ 8.56 (d, *J* = 3.9 Hz, 1 H), 7.52 (dd, *J* = 8.0 and 1.2 Hz, 1 H), 7.32 (dd, *J* = 8.0 and 4.7 Hz, 1 H), 7.23-7.19 (m, 2 H), 6.56-6.52 (m, 2 H), 5.11 (s, 1 H), 4.22-4.09 (m, 4 H), 3.15 (s, 3 H), 1.27 (s, 9 H), 1.20 (t, *J* = 7.2 Hz, 6 H).

13C NMR (100 MHz; $CDCl_3$): δ 167.4 (C), 153.4 (C), 147.4 (CH), 146.3 (C), 144.1 (C), 141.6 (C), 136.3 (CH), 125.8 (CH), 124.4 (CH), 114.2 (CH), 61.6 (CH_2), 55.9 (CH), 39.8 (Me), 33.8 (C), 31.4 (Me), 14.0 (Me).

IR (ATR, cm^{-1}): 2961, 2904, 2869, 1733, 1612, 1576, 1515, 1474, 1456, 1439, 1392, 1366, 1311, 1268, 1201, 1173, 1142, 1095, 1032.

HRMS (ESI): Found MNa^+, 421.2099. $C_{23}H_{30}N_2NaO_4^+$ requires 421.2098. Δ = –0.2 ppm.

1,3-Diethyl 2-(2-phenoxyphenyl)propanedioate 15l

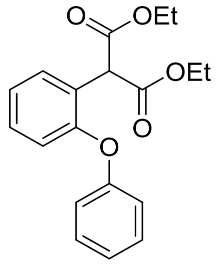

Ethyl 2-(2-phenoxyphenyl)acetate **S1** (0.300 g, 1.17 mmol), LiHMDS (1.00 M in THF, 2.58 mL, 2.58 mmol) and ethyl cyanoformate (0.35 mL, 3.51 mmol) in THF (12 mL) were subjected to General Procedure 3. Purification by flash column chromatography, eluting with EtOAc/Hexane (1:19), afforded the title compound as a colourless oil (0.316 g, 82%).

1H NMR (400 MHz; $CDCl_3$): δ 7.50 (dd, *J* = 7.7 and 1.2 Hz, 1 H), 7.33-7.25 (m, 3 H), 7.16-7.12 (m, 1 H), 7.10-7.06 (m, 1 H), 6.98-6.95 (m, 2 H), 6.89 (d, *J* = 8.0 Hz, 1 H), 5.14 (s, 1 H), 4.25-4.13 (m, 4 H), 1.22 (t, *J* = 7.0 Hz, 6 H).

13C NMR (100 MHz; $CDCl_3$): δ 168.3 (C), 157.3 (C), 154.9 (C), 130.3 (CH), 129.8 (CH), 129.6 (CH), 124.9 (C), 123.9 (CH), 123.4 (CH), 119.0 (CH), 118.6 (CH), 61.8 (CH_2), 51.6 (CH), 14.1 (Me).

IR (ATR, cm^{-1}): 2983, 1732, 1583, 1487, 1454, 1368, 1304, 1230, 1202, 1145, 1094, 1025.

HRMS (ESI): Found MNa^+, 351.1204. $C_{19}H_{20}NaO_5^+$ requires 351.1203. Δ = –0.3 ppm.

1,3-Diethyl 2-[2-(phenylsulfanyl)phenyl]propanedioate 15m

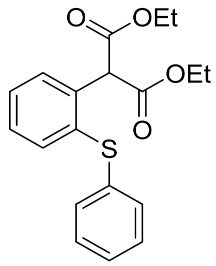

Ethyl 2-[2-(phenylsulfanyl)phenyl]acetate **S3** (0.545 g, 2.00 mmol), LiHMDS (1.00 M in THF, 4.40 mL, 4.40 mmol) and ethyl cyanoformate (0.59 mL, 6.00 mmol) in THF (20 mL) were subjected to General Procedure 3. Purification by flash column chromatography, eluting with EtOAc/Hexane (1:19), afforded the title compound as a colourless oil (0.682 g, 99%).

1H NMR (400 MHz; $CDCl_3$): δ 7.59 (dd, *J* = 7.8 and 1.4 Hz, 1 H), 7.53 (dd, *J* = 7.8 and 1.4 Hz, 1 H), 7.41 (td, *J* = 7.3 and 1.8 Hz, 1 H), 7.32 (td, *J* = 7.8 and 1.4 Hz, 1 H), 7.25-7.20 (m, 2 H), 7.17-7.11 (m, 3 H), 5.53 (s, 1 H), 4.21-4.06 (m, 4 H), 1.20 (t, *J* = 6.9 Hz, 6 H).

13C NMR (100 MHz; $CDCl_3$): δ 168.1 (C), 136.8 (C), 136.3 (CH), 135.5 (C), 133.6 (C), 130.1 (CH), 129.04 (CH), 128.99 (CH), 128.9 (CH), 128.6 (CH), 126.1 (CH), 61.7 (CH_2), 55.0 (CH), 13.9 (Me).

IR (ATR, cm^{-1}): 3060, 2982, 1749, 1731, 1582, 1472, 1440, 1367, 1301, 1258, 1216, 1144, 1095, 1024.

HRMS (ESI): Found MNa^+, 367.0975. $C_{19}H_{20}NaO_4S^+$ requires 367.0975. Δ = –0.1 ppm.

3.0 Cu-Catalysed Synthesis of Acridanes

Optimisation of the Cyclisation Reaction to form Acridanes

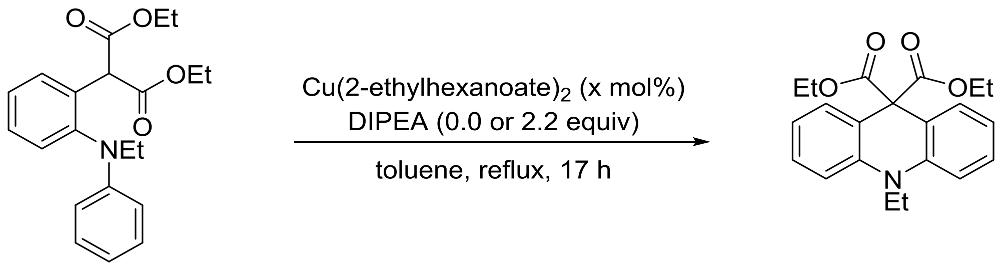

entry	catalyst loading (x mol%)	DIPEA (equiv)	isolated yield
**1**	**10 mol%**	**–**	**87%**
2	10 mol%	2.2	91%
3	5 mol%	–	83%
4	5 mol%	2.2	83%
5	2 mol%	–	84%
6	2 mol%	2.2	83%

General Procedure 4 – Copper(II)-Catalysed Cyclisations

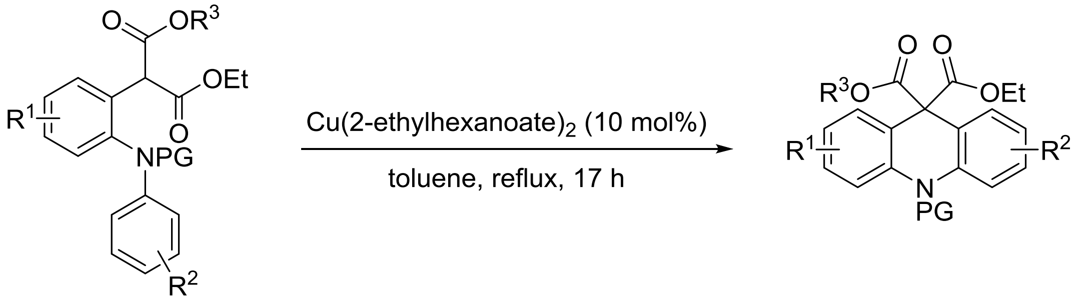

To a solution of the cyclisation precursor (1.00 mmol) in toluene (10 mL) was added copper(II) 2-ethylhexanoate (35.0 mg, 0.100 mmol). The reaction mixture was heated at reflux (oil bath at 120 °C) for 17 h with the condenser left open to the air. After cooling to rt, saturated NH_4Cl (25 mL) was added and the aqueous phase extracted with EtOAc (3 × 25 mL). The combined organics were washed with 10% NH_4OH (25 mL), dried over $MgSO_4$, filtered, and concentrated *in vacuo*. Purification by flash column chromatography, eluting with EtOAc/Hexane, afforded the title compound.

9,9-Diethyl 10-ethyl-9,10-dihydroacridine-9,9-dicarboxylate 16a

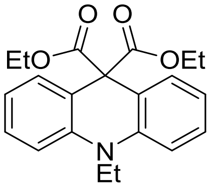

1,2-Diethyl 2-(2-[ethyl(phenyl)amino]phenyl)propanedioate **15a** (0.140 g, 0.394 mmol) and copper(II) 2-ethylhexanoate (14.0 mg, 0.039 mmol) in toluene (3.9 mL) were subjected to General Procedure 4. Purification by flash column chromatography, eluting with EtOAc/Hexane (1:19 to 1:9), afforded the title compound as a colourless solid (0.121 g, 87%).

MP: 79-81 °C.

1H NMR (400 MHz; $CDCl_3$): δ 7.33 (ddd, *J* = 8.6, 7.3 and 1.5 Hz, 2 H), 7.29 (dd, *J* = 7.8 and 1.3 Hz, 2 H), 7.04 (d, *J* = 8.2 Hz, 2 H), 6.99 (td, *J* = 7.8 and 0.8 Hz, 2 H), 4.25 (q, *J* = 7.1 Hz, 4 H), 4.00 (q, *J* = 7.1 Hz, 2 H), 1.41 (t, *J* = 7.1 Hz, 3 H), 1.24 (t, *J* = 7.1 Hz, 6 H).

13C NMR (100 MHz; $CDCl_3$): δ 170.0 (C), 139.4 (C), 129.7 (CH), 128.7 (CH), 119.9 (CH), 119.7 (C), 112.6 (CH), 62.1 (C), 62.0 (CH_2), 40.6 (CH_2), 13.9 (Me), 11.6 (Me).

IR (ATR, cm^{-1}): 2980, 1738, 1725, 1593, 1481, 1461, 1377, 1302, 1258, 1221, 1209, 1169, 1085, 1040.

HRMS (ESI): Found MNa^+, 376.1515. $C_{21}H_{23}NNaO_4^+$ requires 376.1519. Δ = 1.1 ppm.

9,9-Diethyl 10-benzyl-9,10-dihydroacridine-9,9-dicarboxylate 16b

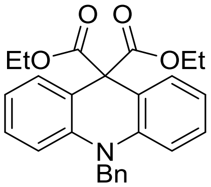

1,2-Diethyl 2-(2-[benzyl(phenyl)amino]phenyl)propanedioate **15b** (0.418 g, 1.00 mmol) and copper(II) 2-ethylhexanoate (35.0 mg, 0.100 mmol) in toluene (10 mL) were subjected to General Procedure 4. Purification by flash column chromatography, eluting with EtOAc/Hexane (1:19 to 1:9), afforded the title compound as a colourless solid (0.266 g, 64%).

MP: 143-145 °C.

1H NMR (400 MHz; $CDCl_3$): δ 7.36-7.28 (m, 5 H), 7.24-7.21 (m, 2 H), 7.21-7.16 (ddd, *J* = 8.8, 7.4 and 1.5 Hz, 2 H), 6.99 (td, *J* = 7.0 and 1.2 Hz, 2 H), 6.79 (dd, *J* = 6.8 and 0.8 Hz, 2 H), 5.16 (s, 2 H), 4.27 (q, *J* = 7.0 Hz, 4 H), 1.26 (t, *J* = 7.0 Hz, 6 H).

13C NMR (100 MHz; $CDCl_3$): δ 170.4 (C), 140.0 (C), 136.5 (C), 129.5 (CH), 128.8 (2 × CH), 127.0 (CH), 126.0 (CH), 120.4 (CH), 120.1 (C), 113.7 (CH), 62.11 (CH_2), 62.09 (C), 51.1 (CH_2), 13.9 (Me).

IR (ATR, cm^{-1}): 2980, 1738, 1592, 1482, 1459, 1376, 1212, 1042.

HRMS (ESI): Found MNa^+, 438.1659. $C_{26}H_{25}NNaO_4^+$ requires 438.1676. Δ = 3.7 ppm.

9-Benzyl-9-ethyl 10-ethyl-9,10-dihydroacridine-9,9-dicarboxylate 16c

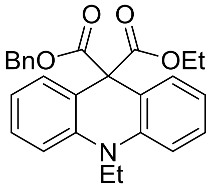

1-Benzyl-3-ethyl 2-(2-[ethyl(phenyl)amino]phenyl)propanedioate **15c** (0.209 g, 0.500 mmol) and copper(II) 2-ethylhexanoate (17.5 mg, 0.050 mmol) in toluene (5 mL) were subjected to General Procedure 4. Purification by flash column chromatography, eluting with EtOAc/Hexane (0:1 to 1:19), afforded the title compound as a colourless solid (0.152 g, 73%).

MP: 73-74 °C.

1H NMR (400 MHz; $CDCl_3$): δ 7.36 (td, *J* = 8.2 and 0.8 Hz, 2 H), 7.32-7.30 (m, 5 H), 7.21-7.19 (m, 2 H), 7.06 (d, *J* = 8.3 Hz, 2 H), 7.01 (t, *J* = 7.3 Hz, 2 H), 5.23 (s, 2 H), 4.28 (q, *J* = 7.2 Hz, 2 H), 4.00 (q, *J* = 6.9 Hz, 2 H), 1.40 (t, *J* = 6.9 Hz, 3 H), 1.23 (t, *J* = 7.2 Hz, 3 H).

13C NMR (100 MHz; $CDCl_3$): δ 170.3 (C), 170.1 (C), 139.3 (C), 135.4 (C), 129.7 (CH), 128.7 (CH), 128.2 (CH), 127.8 (CH), 127.6 (CH), 119.9 (CH), 119.6 (C), 112.6 (CH), 67.2 (CH_2), 62.1 (C), 62.0 (CH_2), 40.4 (CH_2), 13.8 (Me), 11.4 (Me).

IR (ATR, cm^{-1}): 3032, 2982, 1727, 1594, 1479, 1459, 1376, 1321, 1297, 1254, 1217, 1201, 1176, 1091, 1031.

HRMS (ESI): Found MH^+, 416.1849. $C_{26}H_{26}NO_4^+$ requires 416.1856. Δ = 1.9 ppm.

9-Allyl-9-ethyl 10-ethyl-9,10-dihydroacridine-9,9-dicarboxylate 16d

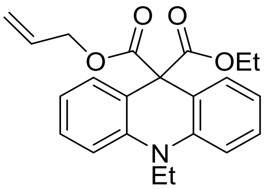

1-Allyl-3-ethyl 2-(2-[ethyl(phenyl)amino]phenyl)propanedioate **15d** (0.110 g, 0.300 mmol) and copper(II) 2-ethylhexanoate (10.5 mg, 0.030 mmol) in toluene (3 mL) were subjected to General Procedure 4. Purification by flash column chromatography, eluting with EtOAc/Hexane (0:1 to 1:19), afforded the title compound as a colourless solid (0.092 g, 84%).

MP: 65-66 °C.

1H NMR (400 MHz; $CDCl_3$): δ 7.37-7.31 (m, 4 H), 7.06 (d, *J* = 8.2 Hz, 2 H), 7.01 (td, *J* = 7.9 and 0.9 Hz, 2 H), 5.91-5.81 (m, 1 H), 5.21-5.15 (m, 2 H), 4.67 (dt, *J* = 5.4 and 1.5 Hz, 2 H), 4.29 (q, *J* = 7.2 Hz, 2 H), 4.02 (q, *J* = 6.9 Hz, 2 H), 1.42 (t, *J* = 7.2 Hz, 3 H), 1.26 (t, *J* = 6.9 Hz, 3 H).

13C NMR (100 MHz; $CDCl_3$): δ 170.4 (C), 170.1 (C), 139.3 (C), 131.4 (CH), 129.7 (CH), 128.8 (CH), 119.9 (CH), 119.5 (C), 117.9 (CH_2), 112.6 (CH), 66.2 (CH_2), 62.1 (C), 62.0 (CH_2), 40.5 (CH_2), 13.8 (Me), 11.5 (Me).

IR (ATR, cm^{-1}): 2981, 1738, 1724, 1593, 1480, 1460, 1376, 1257, 1209, 1169, 1085, 1037, 1001.

HRMS (ESI): Found MH^+, 366.1698. $C_{22}H_{24}NO_4^+$ requires 366.1700. Δ = 0.4 ppm.

9-Ethyl-9-(9*H*-fluoren-9-ylmethyl) 10-ethyl-9,10-dihydroacridine-9,9-dicarboxylate 16e

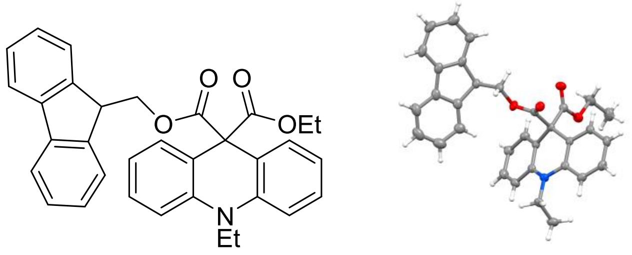

1-Ethyl-3-(9*H*-fluoren-9-ylmethyl) 2-(2-[ethyl(phenyl)amino]phenyl)propanedioate **15e** (0.126 g, 0.250 mmol) and copper(II) 2-ethylhexanoate (8.7 mg, 0.025 mmol) in toluene (2.5 mL) were subjected to General Procedure 4. Purification by flash column chromatography, eluting with EtOAc/Hexane (1:19), afforded the title compound as a colourless solid (0.104 g, 83%).

MP: 83-85 °C.

1H NMR (400 MHz; $CDCl_3$): δ 7.73 (d, *J* = 7.6 Hz, 2 H), 7.43-7.34 (m, 6 H), 7.21 (td, *J* = 7.4 and 0.8 Hz, 2 H), 7.14 (d, *J* = 7.9 Hz, 2 H), 7.11 (d, *J* = 8.7 Hz, 2 H), 7.05 (t, *J* = 7.7 Hz, 2 H), 4.41 (d, *J* = 7.3 Hz, 2 H), 4.35 (q, *J* = 7.1 Hz, 2 H), 4.11 (t, *J* = 7.3 Hz, 1 H), 4.03 (q, *J* = 7.2 Hz, 2 H), 1.43 (t, *J* = 7.2 Hz, 3 H), 1.30 (t, *J* = 7.1 Hz, 3 H).

13C NMR (100 MHz; $CDCl_3$): δ 170.3 (C), 170.1 (C), 143.2 (C), 140.8 (C), 139.2 (C), 129.5 (CH), 128.6 (CH), 127.3 (CH), 126.6 (CH), 124.8 (CH), 119.9 (CH), 119.5 (CH), 119.4 (C), 112.5 (CH), 67.8 (CH_2), 62.0 (C+CH_2), 46.2 (CH), 40.3 (CH_2), 13.6 (Me), 11.3 (Me).

IR (ATR, cm^{-1}): 2980, 1737, 1720, 1593, 1479, 1459, 1375, 1233, 1208, 1178, 1087, 1040, 990.

HRMS (ESI): Found MH^+, 504.2160. $C_{33}H_{30}NO_4^+$ requires 504.2169. Δ = 1.8 ppm.

XRAY: CCDC number 1498037.

9-Ethyl-9-(*L*-menthyl) 10-ethyl-9,10-dihydroacridine-9,9-dicarboxylate 16f

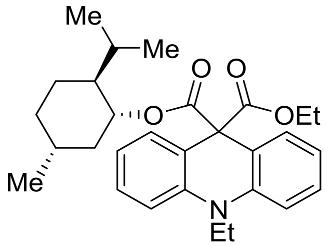

1-Ethyl-3-(*L*-menthyl) 2-(2-[ethyl(phenyl)amino]phenyl)propanedioate **15f** (0.140 g, 0.300 mmol) and copper(II) 2-ethylhexanoate (10.5 mg, 0.030 mmol) in toluene (3 mL) were subjected to General Procedure 4. Purification by flash column chromatography, eluting with EtOAc/Hexane (1:19), afforded the title compound as a colourless solid (0.121 g, 87%).

MP: 120-122 °C.

1H NMR (400 MHz; $CDCl_3$): δ 7.38-7.30 (m, 4 H), 7.08 (dd, *J* = 8.2 and 5.8 Hz, 2 H), 7.03 (dd, *J* = 7.7 and 7.3 Hz, 2 H), 4.70 (td, *J* = 10.9 and 4.3 Hz, 1 H), 4.39-4.26 (m, 2 H), 4.04 (q, *J* = 7.0 Hz, 2 H), 2.19-2.16 (m, 1 H), 1.71-1.62 (m, 2 H), 1.56-1.42 (m, 2 H), 1.45 (t, *J* = 7.0 Hz, 3 H), 1.33-1.25 (m, 1 H), 1.31 (t, *J* = 7.2 Hz, 3 H), 1.09-0.97 (m, 2 H), 0.96 (d, *J* = 6.8 Hz, 3 H), 0.92-0.82 (m, 1 H), 0.77 (d, *J* = 6.9 Hz, 3 H), 0.67 (d, *J* = 6.9 Hz, 3 H).

13C NMR (100 MHz; $CDCl_3$): δ 170.5 (C), 169.8 (C), 139.4 (C), 139.3 (C), 129.9 (CH), 129.3 (CH), 128.6 (CH), 128.4 (CH), 120.3 (C), 120.0 (C), 119.74 (CH), 119.69 (CH), 112.5 (CH), 112.4 (CH), 76.2 (CH), 62.3 (C), 61.9 (CH_2), 46.6 (CH), 40.4 (CH_2), 39.9 (CH_2), 34.0 (CH_2), 31.2 (CH), 25.1 (CH), 22.8 (CH_2), 21.9 (Me), 20.6 (Me), 15.6 (Me), 13.8 (Me), 11.6 (Me).

IR (ATR, cm^{-1}): 2956, 2867, 1723, 1591, 1484, 1456, 1376, 1298, 1258, 1224, 1178, 1152, 1135, 1097, 1085, 1061, 1042.

HRMS (ESI): Found MH^+, 486.2599. $C_{29}H_{37}NNaO_4^+$ requires 486.2615. Δ = 3.2 ppm.

9,9-Diethyl 10-ethyl-2-fluoro-9,10-dihydroacridine-9,9-dicarboxylate 16g

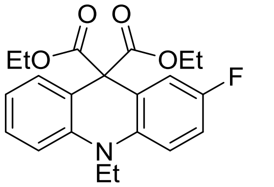

1,2-Diethyl 2-(2-[ethyl(4-fluorophenyl)amino]phenyl)propanedioate **15g** (0.373 g, 1.00 mmol) and copper(II) 2-ethylhexanoate (35.0 mg, 0.100 mmol) in toluene (10 mL) were subjected to General Procedure 4. Purification by flash column chromatography, eluting with EtOAc/Hexane (1:19), afforded the title compound as a colourless solid (0.325 g, 88%).

MP: 85-86 °C.

1H NMR (400 MHz; $CDCl_3$): δ 7.33 (ddd, *J* = 8.7, 7.3 and 1.6 Hz, 1 H), 7.29 (dd, *J* = 7.8 and 1.4 Hz, 1 H), 7.08-6.95 (m, 5 H), 4.26 (q, *J* = 7.1 Hz, 4 H), 3.97 (q, *J* = 7.1 Hz, 2 H), 1.38 (t, *J* = 7.1 Hz, 3 H), 1.25 (t, *J* = 7.1 Hz, 6 H).

13C NMR (100 MHz; $CDCl_3$): δ 169.6 (C), 156.4 (d, *J* = 237 Hz, C), 139.0, 135.6 (d, *J* = 1.6 Hz, C), 129.3 (CH), 128.6 (CH), 120.7 (d, *J* = 7.1 Hz, C), 119.6 (CH), 118.6, 115.7 (d, *J* = 24.1 Hz, CH), 115.2 (d, *J* = 22.5 Hz, CH), 113.2 (d, *J* = 8.0 Hz, CH), 112.3 (CH), 61.8 (CH_2), 61.7 (C), 40.4 (CH_2), 13.5 (Me), 11.0 (Me).

IR (ATR, cm^{-1}): 2980, 1733, 1717, 1596, 1514, 1482, 1455, 1432, 1374, 1286, 1261, 1219, 1171, 1136, 1087, 1037.

HRMS (ESI): Found MNa^+, 394.1422. $C_{21}H_{22}FNNaO_4^+$ requires 394.1425. Δ = 0.7 ppm.

9,9-Diethyl 1,3-dichloro-10-ethyl-9,10-dihydroacridine-9,9-dicarboxylate 16h

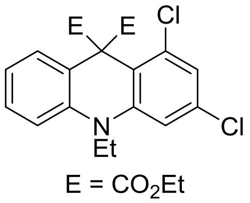

1,2-Diethyl 2-(2-[ethyl(3,5-dichlorophenyl)amino]phenyl)propanedioate **15h** (0.424 g, 1.00 mmol) and copper(II) 2-ethylhexanoate (35.0 mg, 0.100 mmol) in toluene (10 mL) were subjected to General Procedure 4. Purification by flash column chromatography, eluting with EtOAc/Hexane (1:19), afforded the title compound as a colourless solid (0.279 g, 66%).

MP: 152-153 °C.

1H NMR (400 MHz; $CDCl_3$): δ 7.52 (d, *J* = 7.9 Hz, 1 H), 7.30-7.25 (m, 1 H), 6.98-6.92 (m, 3 H), 6.89 (s, 1 H), 4.24-4.08 (m, 4 H), 3.90 (q, *J* = 7.1 Hz, 2 H), 1.38 (t, *J* = 7.1 Hz, 3 H), 1.16 (t, *J* = 6.9 Hz, 3 H), 1.15 t, *J* = 6.9 Hz, 3 H).

13C NMR (100 MHz; $CDCl_3$): δ 168.4 (C), 141.7 (C), 137.5 (C), 136.4 (C), 134.3 (C), 129.3 (CH), 128.8 (CH), 121.1 (CH), 120.8 (CH), 118.8 (C), 117.8 (C), 113.0 (CH), 111.6 (CH), 62.1 (CH_2), 58.9 (C), 41.6 (CH_2), 13.6 (Me), 11.5 (Me).

IR (ATR, cm^{-1}): 2982, 1728, 1577, 1556, 1494, 1458, 1423, 1388, 1358, 1306, 1283, 1232, 1217, 1143, 1106, 1084, 1048, 1024.

HRMS (ESI): Found MNa^+, 444.0722. $C_{21}H_{21}Cl_2NNaO_4^+$ requires 444.0740. Δ = 4.0 ppm.

9,9-Diethyl 10-ethyl-1,3-dimethyl-9,10-dihydroacridine-9,9-dicarboxylate 16i

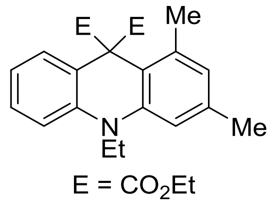

1,2-Diethyl 2-(2-[ethyl(3,5-dimethylphenyl)amino]phenyl)propanedioate **15i** (0.192 g, 0.500 mmol) and copper(II) 2-ethylhexanoate (17.5 mg, 0.050 mmol) in toluene (5 mL) were subjected to General Procedure 4. Purification by flash column chromatography, eluting with EtOAc/Hexane (1:19), afforded the title compound as a colourless solid (0.119 g, 62%).

MP: 150-152 °C.

1H NMR (400 MHz; $CDCl_3$): δ 7.33-7.29 (m, 2 H), 7.01 (dd, *J* = 8.3 and 1.1 Hz, 1 H), 6.94 (ddd, *J* = 8.5, 7.1 and 0.9 Hz, 1 H), 6.76 (s, 1 H), 6.69 (s, 1 H), 4.29-4.17 (m, 4 H), 3.99 (q, *J* = 7.0 Hz, 2 H), 2.38 (s, 3 H), 2.37 (s, 3 H), 1.44 (t, *J* = 7.0 Hz, 3 H), 1.22 (t, *J* = 7.0 Hz, 6 H).

13C NMR (100 MHz; $CDCl_3$): δ 170.5 (C), 139.9 (C), 139.1 (C), 138.5 (C), 137.6 (C), 128.6 (2×CH), 125.0 (CH), 120.3 (C), 119.4 (CH), 116.8 (C), 112.6 (CH), 111.9 (CH), 61.8 (CH_2), 60.0 (C), 41.3 (CH_2), 21.4 (Me), 21.0 (Me), 13.7 (Me), 11.9 (Me).

IR (ATR, cm^{-1}): 2981, 1725, 1590, 1570, 1478, 1460, 1371, 1288, 1214, 1149, 1094, 1047, 1024.

HRMS (ESI): Found MNa^+, 404.1833. $C_{23}H_{27}NNaO_4^+$ requires 404.1832. Δ = –0.2 ppm.

9,9-Diethyl 6-chloro-10-ethyl-2-methoxy-9,10-dihydroacridine-9,9-dicarboxylate 16j

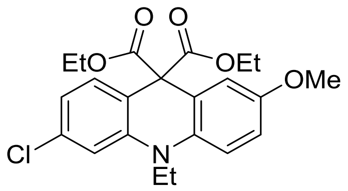

1,2-Diethyl 2-(4-chloro-2-[ethyl(4-methoxyphenyl)amino]phenyl)propanedioate **15j** (0.210 g, 0.500 mmol) and copper(II) 2-ethylhexanoate (17.5 mg, 0.050 mmol) in toluene (5 mL) were subjected to General Procedure 4. Purification by flash column chromatography, eluting with EtOAc/Hexane (1:19), afforded the title compound as a colourless solid (0.174 g, 83%).

MP: 87-89 °C.

1H NMR (400 MHz; $CDCl_3$): δ 7.16 (d, *J* = 8.2 Hz, 1 H), 6.96 (d, *J* = 8.7 Hz, 1 H), 6.93 (dd, *J* = 8.2 and 2.8 Hz, 1 H), 6.89 (d, *J* = 8.2 and 2.3 Hz, 1 H), 6.84 (d, *J* = 2.8 Hz, 1 H), 4.23 (q, *J* = 6.9 Hz, 4 H), 3.92 (q, *J* = 7.3 Hz, 2 H), 3.78 (s, 3 H), 1.37 (t, *J* = 7.3 Hz, 3 H), 1.22 (t, *J* = 6.9 Hz, 6 H).

13C NMR (100 MHz; CDCl$_3$): δ 170.0 (C), 153.7 (C), 140.7 (C), 134.7 (C), 133.1 (C), 131.1 (CH), 120.5 (C), 119.3 (CH), 117.4 (C), 115.0 (CH), 114.8 (CH), 113.8 (CH), 112.4 (CH), 62.2 (CH$_2$), 61.8 (C), 55.6 (Me), 40.8 (CH$_2$), 13.9 (Me), 11.4 (Me).

IR (ATR, cm^{-1}): 2981, 1725, 1621, 1592, 1562, 1514, 1485, 1437, 1366, 1297, 1220, 1159, 1111, 1098, 1085, 1042, 1017.

HRMS (ESI): Found MNa$^+$, 440.1224. $C_{22}H_{24}ClNNaO_5^+$ requires 440.1235. Δ = 2.5 ppm.

10,10-Diethyl 8-*tert*-butyl-5-methyl-5*H*,10*H*-benzo[*b*]1,5-naphthyridine-10,10-dicarboxylate 16k

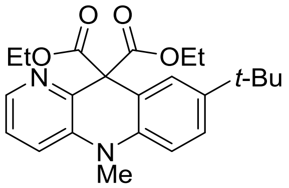

1,3-Diethyl 2-{3-[(4-*tert*-butylphenyl)(methyl)amino]pyridine-2-yl}propanedioate **15k** (80.0 mg, 0.201 mmol) and copper(II) 2-ethylhexanoate (7.0 mg, 0.020 mmol) in toluene (2 mL) were subjected to General Procedure 4. Purification by flash column chromatography, eluting with EtOAc/Hexane (1:9 to 1:2), afforded the title compound as a colourless solid (52.7 mg, 66%).

MP: 92-94 °C.

1H NMR (400 MHz; CDCl$_3$): δ 8.24 (dd, *J* = 3.6 and 1.8 Hz, 1 H), 7.39-7.35 (m, 2 H), 7.25-7.19 (m, 2 H), 6.92 (d, *J* = 8.3 Hz, 1 H), 4.35-4.16 (m, 4 H), 3.35 (s, 3 H), 1.33 (s, 9 H), 1.22 (t, *J* = 6.9 Hz, 6 H).

13C NMR (100 MHz; CDCl$_3$): δ 169.5 (C), 143.5 (C), 141.0 (CH), 140.7 (C), 137.6 (C), 136.8 (C), 127.5 (CH), 125.6 (CH), 123.1 (CH), 120.3 (C), 118.9 (CH), 112.0 (CH), 64.9 (C), 61.9 (CH$_2$), 34.0 (C), 32.5 (Me), 31.3 (Me), 13.9 (Me).

IR (ATR, cm^{-1}): 2959, 2901, 2868, 1739, 1575, 1509, 1500, 1472, 1461, 1444, 1366, 1352, 1282, 1208, 1163, 1140, 1107, 1096, 1056, 1046, 1029.

HRMS (ESI): Found MNa$^+$, 419.1941. $C_{23}H_{28}N_2NaO_4^+$ requires 419.1941. Δ = 0.1 ppm.

9,9-Diethyl 9*H*-xanthene-9,9-dicarboxylate 16l

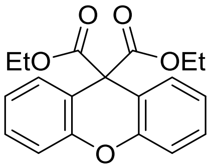

To a solution of 1,3-diethyl 2-(2-phenoxyphenyl)propanedioate **15l** (60.0 mg, 0.183 mmol) in toluene (1.8 mL) was added copper(II) 2-ethylhexanoate (160 mg, 0.458 mmol) and DIPEA (80.0 µL, 0.458 mmol). The reaction mixture was heated at reflux (oil bath at 120 °C) for 19 h under an atmosphere of argon. After cooling to rt, saturated NH_4Cl (20 mL) was added and the aqueous phase extracted with

EtOAc (3 × 20 mL). The combined organics were washed with 10% NH_4OH (20 mL) and saturated brine (20 mL), dried over $MgSO_4$, filtered, and concentrated *in vacuo*. Purification by flash column chromatography, eluting with EtOAc/Hexane (1:19), afforded the title compound as a colourless solid (28.0 mg, 47%).

MP: 69-71 °C.

1H NMR (400 MHz; $CDCl_3$): δ 7.48 (d, *J* = 8.0 Hz, 2 H), 7.36-7.32 (m, 2 H), 7.15-7.09 (m, 4 H), 4.23 (q, *J* = 7.0 Hz, 4 H), 1.22 (t, *J* = 7.0 Hz, 6 H).

13C NMR (100 MHz; $CDCl_3$): δ 169.6 (C), 150.4 (C), 130.0 (CH), 129.8 (CH), 123.0 (CH), 117.5 (C), 116.9 (CH), 62.5 (CH_2), 58.4 (C), 14.0 (Me).

IR (ATR, cm^{-1}): 3007, 2984, 2968, 2939, 1746, 1713, 1682, 1601, 1571, 1480, 1456, 1440, 1388, 1367, 1311, 1293, 1213, 1127, 1098, 1037.

HRMS (ESI): Found MNa^+, 349.1045. $C_{19}H_{18}NaO_5^+$ requires 349.1046. Δ = 0.5 ppm.

9,9-Diethyl 9*H*-thioxanthene-9,9-dicarboxylate 16m

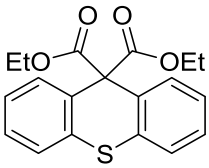

To a solution of 1,3-diethyl 2-[2-(phenylsulfanyl)phenyl]propanedioate **15m** (103 mg, 0.300 mmol) in toluene (3 mL) was added copper(II) 2-ethylhexanoate (262 mg, 0.750 mmol) and DIPEA (131 µL, 0.750 mmol). The reaction mixture was heated at reflux (oil bath at 120 °C) for 20 h under an atmosphere of argon. After cooling to rt, saturated NH_4Cl (20 mL) was added and the aqueous phase extracted with EtOAc (3 × 20 mL). The combined organics were washed with 10% NH_4OH (20 mL) and saturated brine (20 mL), dried over $MgSO_4$, filtered, and concentrated *in vacuo*. Purification by flash column chromatography, eluting with EtOAc/Hexane (1:19), afforded the title compound as a colourless solid (45.3 mg, 44%).

MP: 95-96 °C.

1H NMR (400 MHz; $CDCl_3$): δ 7.45-7.43 (m, 2 H), 7.35-7.31 (m, 2 H), 7.30-7.27 (m, 4 H), 4.29 (q, *J* = 7.1 Hz, 4 H), 1.23 (t, *J* = 7.1 Hz, 6 H).

13C NMR (100 MHz; $CDCl_3$): δ 169.5 (C), 133.8 (C), 132.2 (C), 129.2 (CH), 127.9 (CH), 126.8 (CH), 126.5 (CH), 67.6 (C), 62.5 (CH_2), 14.1 (Me).

IR (ATR, cm^{-1}): 2981, 2935, 1740, 1726, 1465, 1440, 1366, 1294, 1200, 1139, 1094, 1032, 1015.

HRMS (ESI): Found MNa^+, 365.0811. $C_{19}H_{18}NaO_4S^+$ requires 365.0818. Δ = 2.0 ppm.

4.0 Derivatisation of Acridanes

10-Ethyl-9,10-dihydroacridine-9-carboxylic acid 17

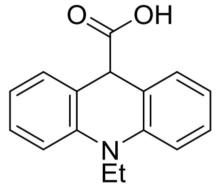

To a solution of 9,9-diethyl 10-ethyl-9,10-dihydroacridine-9,9-dicarboxylate **16a** (88.0 mg, 0.250 mmol) in EtOH (2.5 mL) was added KOH (0.140 g, 2.50 mmol) and H_2O (0.5 mL). The reaction mixture was heated at 90 °C for 4.5 h. After cooling to rt, the reaction was diluted with H_2O (5 mL), washed with Et_2O (10 mL), and the ethereal extract discarded. The aqueous phase was acidified with 10% HCl, then extracted with EtOAc (3 × 10 mL). The combined organics were dried over $MgSO_4$, filtered, and concentrated *in vacuo*. Purification by flash column chromatography, eluting with EtOAc/Hexane (1:1), afforded the title compound as a pale brown solid (61.8 mg, 98%).

MP: 108-110 °C.

1H NMR (400 MHz; DMSO-*d*$_6$): δ 7.27-7.21 (m, 4 H), 7.08 (d, *J* = 8.2 Hz, 2 H), 6.90 (td, *J* = 7.3 and 0.6 Hz, 2 H), 4.90 (s, 1 H), 3.98 (q, *J* = 7.0 Hz, 2 H), 1.24 (t, *J* = 7.0 Hz, 3 H).

13C NMR (100 MHz; DMSO-*d*$_6$): δ 173.2 (C), 140.0 (C), 129.3 (CH), 128.1 (CH), 120.4 (C), 119.9 (CH), 112.7 (CH), 48.2 (CH), 39.4 (CH_2), 11.0 (Me).

IR (ATR, cm^{-1}): 2973, 2914, 1701, 1684, 1633, 1593, 1478, 1460, 1416, 1370, 1289, 1256, 1225, 1211, 1189, 1170, 1133, 1100, 1084, 1052.

HRMS (ESI): Found MNa^+, 276.1001. $C_{16}H_{15}NNaO_2^+$ requires 276.0995. Δ = –2.3 ppm.

10-Ethyl-9,9-di(hydroxymethyl)-9,10-dihydroacridine 18

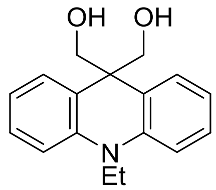

To a suspension of $LiAlH_4$ (29.0 mg, 0.750 mmol) in Et_2O (1 mL) was added 9,9-diethyl 10-ethyl-9,10-dihydroacridine-9,9-dicarboxylate **16a** (88.0 mg, 0.250 mmol) in Et_2O (1.5 mL). The reaction mixture was stirred at rt for 18 h, then quenched by the slow addition of H_2O (2 mL). The aqueous phase was extracted with Et_2O (3 × 10 mL), and the combined organics were dried over $MgSO_4$, filtered, and concentrated *in vacuo*. Purification by flash column chromatography, eluting with EtOAc/Hexane (1:9 to 1:4), afforded the title compound as a colourless solid (63.3 mg, 94%).

1H NMR (400 MHz; CDCl$_3$): δ 7.43 (dd, *J* = 7.7 and 1.4 Hz, 2 H), 7.25 (ddd, *J* = 8.6, 7.3 and 1.6 Hz, 2 H), 7.02-6.97 (m, 4 H), 3.98 (q, *J* = 7.0 Hz, 2 H), 3.91 (s, 4 H), 1.41 (t, *J* = 7.0 Hz, 3 H).

13C NMR (100 MHz; CDCl$_3$): δ 141.2 (C), 127.9 (CH), 126.4 (CH), 122.4 (C), 120.3 (CH), 112.7 (CH), 64.7 (CH$_2$), 48.6 (C), 40.3 (CH$_2$), 11.4 (Me).

IR (ATR, cm^{-1}): 3369, 2981, 2931, 1590, 1475, 1459, 1375, 1314, 1258, 1168, 1135, 1103, 1048.

HRMS (ESI): Found MNa$^+$, 292.1302. $C_{17}H_{19}NNaO_2^+$ requires 292.1308. Δ = 2.2 ppm.

Ethyl 10-ethyl-9-(prop-2-en-1-yl)-9,10-dihydroacridine-9-carboxylate 19

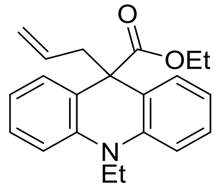

A solution of 9-allyl-9-ethyl 10-ethyl-9,10-dihydroacridine-9,9-dicarboxylate **16c** (55.0 mg, 0.151 mmol) and Pd(PPh$_3$)$_4$ (7.0 mg, 0.006 mmol) in THF was stirred at rt for 2 h, then concentrated in vacuo. The crude product was purified by flash column chromatography, eluting with EtOAc/Hexane (1:49 to 1:19), afforded the title compound as a colourless oil (47.6 mg, 98%).

1H NMR (400 MHz; CDCl$_3$): δ 7.22 (ddd, *J* = 8.5, 7.1 and 1.5 Hz, 2 H), 7.02 (dd, *J* = 7.7 and 1.6 Hz, 2 H), 6.95 (d, *J* = 8.2 Hz, 2 H), 6.89 (ddd, *J* = 8.2, 7.5 and 0.9 Hz, 2 H), 5.43 (dddd, *J* = 17.4, 10.3, 7.2 and 7.2 Hz, 1 H), 4.78-4.75 (m, 1 H), 4.68-4.63 (m, 1 H), 4.23 (q, *J* = 7.0 Hz, 2 H), 3.94 (q, *J* = 7.2 Hz, 2 H), 2.77-2.74 (m, 2 H), 1.40 (t, *J* = 7.0 Hz, 3 H), 1.17 (t, *J* = 7.2 Hz, 3 H).

13C NMR (100 MHz; CDCl$_3$): δ 174.4 (C), 139.7 (C), 133.4 (CH), 127.8 (CH), 127.5 (CH), 123.6 (C), 119.8 (CH), 117.9 (CH$_2$), 112.2 (CH), 61.2 (CH$_2$), 53.6 (C), 47.8 (CH$_2$), 40.5 (CH$_2$), 13.9 (Me), 11.7 (Me).

IR (ATR, cm^{-1}): 2980, 2933, 1723, 1592, 1479, 1456, 1375, 1320, 1292, 1259, 1221, 1170, 1153, 1136, 1087, 1054.

HRMS (ESI): Found MNa$^+$, 344.1617. $C_{21}H_{23}NNaO_2^+$ requires 344.1621. Δ = 1.2 ppm.

9,9-Diethyl 9,10-dihydroacridine-9,9-dicarboxylate 20

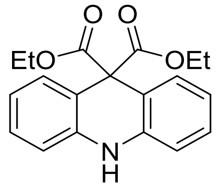

A solution of 9,9-diethyl 10-benzyl-9,10-dihydroacridine-9,9-dicarboxylate **16b** (51.0 mg, 0.123 mmol) and 10% Pd/C (10.0 mg) in EtOH (3 mL) was evacuated and backfilled with H_2 4 times. The reaction mixture was heated at 50 °C for 4 h under an atmosphere of H_2 (balloon). After flushing with argon, the mixture was then filtered through Celite, which was washed with EtOH (2 × 10 mL). The combined organics were concentrated in vacuo. Purification by flash column chromatography, eluting with EtOAc/Hexane (1:49 to 1:9), afforded the title compound as a colourless solid (27.1 mg, 68%).

MP: 130-131 °C.

1H NMR (400 MHz; $CDCl_3$): δ 7.33 (dd, *J* = 7.9 and 0.8 Hz, 2 H), 7.18 (ddd, *J* = 8.2, 8.2 and 1.3 Hz, 2 H), 6.91 (ddd, *J* = 8.4, 8.4 and 1.1 Hz, 2 H), 6.69 (dd, *J* = 8.2 and 1.1 Hz, 2 H), 6.38 (br s, 1 H), 4.23 (q, *J* = 7.1 Hz, 4 H), 1.22 (t, *J* = 7.1 Hz, 6 H).

13C NMR (100 MHz; $CDCl_3$): δ 170.3 (C), 137.8 (C), 129.9 (CH), 128.8 (CH), 120.3 (CH), 116.6 (C), 114.2 (CH), 62.1 (CH_2), 61.3 (C), 13.9 (Me).

IR (ATR, cm^{-1}): 3388, 3379, 2972, 2939, 1731, 1698, 1611, 1582, 1520, 1507, 1484, 1463, 1386, 1366, 1330, 1290, 1216, 1160, 1142, 1091, 1042, 1013.

HRMS (ESI): Found MNa^+, 348.1207. $C_{19}H_{19}NNaO_4^+$ requires 348.1206. Δ = –0.2 ppm.

5.0 One-Pot α-Arylation/Cyclisation Approach to Acridanes

General Procedure 5 – Synthesis of 2-Bromo-*N*-phenylanilines

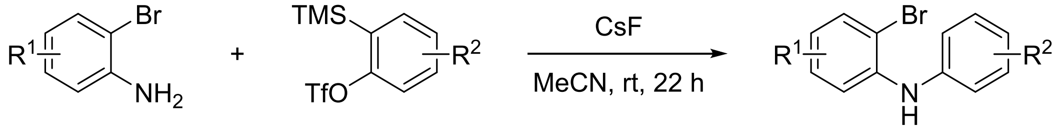

To a solution of the 2-bromoaniline (16.6 mmol) and aryne precursor (13.8 mmol) in MeCN (138 mL) was added CsF (6.29 g, 41.4 mmol). The reaction mixture was stirred at rt for 22 h under at atmosphere of air. H_2O (100 mL) was then added and the aqueous phase extracted with EtOAc (3 × 75 mL). The combined organics were dried over $MgSO_4$, filtered, and concentrated *in vacuo*. Purification by flash column chromatography, eluting with EtOAc/Hexane, afforded the title compound.

2-Bromo-*N*-phenylaniline S6

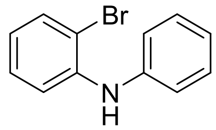

2-Bromoaniline (2.86 g, 16.6 mmol), 2-(trimethylsilyl)phenyl trifluoromethanesulfonate (4.12 g, 13.8 mmol) and CsF (6.29 g, 41.4 mmol) in MeCN (138 mL) were subjected to General Procedure 5. Purification by flash column chromatography, eluting with EtOAc/Hexane (0:1 to 1:19), afforded the title compound as a colourless oil (2.75 g, 80%).

1H NMR (400 MHz; $CDCl_3$): δ 7.56 (dd, *J* = 8.0 and 1.5 Hz, 1 H), 7.38-7.33 (m, 2 H), 7.29 (dd, *J* = 8.3 and 1.5 Hz, 1 H), 7.21-7.17 (m, 3 H), 7.08 (tt, *J* = 7.3 and 1.1 Hz, 1 H), 6.77 (ddd, *J* = 8.5, 7.5 and 1.7 Hz, 1 H), 6.12 (br s, 1 H).

13C NMR (100 MHz; $CDCl_3$): δ 141.5 (C), 141.4 (C), 133.0 (CH), 129.4 (CH), 128.1 (CH), 122.7 (CH), 120.9 (CH), 120.2 (CH), 115.7 (CH), 112.1 (C).

IR (ATR, cm^{-1}): 3395, 2981, 1583, 1506, 1496, 1464, 1450, 1414, 1309, 1288, 1240, 1218, 1175, 1157, 1077, 1020.

Data is consistent with literature values.[10]

[10] M. E. Budén, V. A. Vaillard, S. E. Martin and R. A. Rossi *J. Org. Chem.* **2009**, *74*, 4490-4498.

2-Bromo-5-methyl-*N*-phenylaniline S7

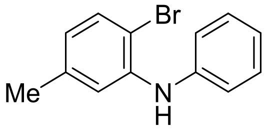

2-Bromo-5-methylaniline (0.572 g, 3.07 mmol), 2-(trimethylsilyl)phenyl trifluoromethanesulfonate (0.765 g, 2.56 mmol) and CsF (1.17 g, 7.68 mmol) in MeCN (26 mL) were subjected to General Procedure 5. Purification by flash column chromatography, eluting with EtOAc/Hexane (0:1 to 1:49), afforded the title compound as a colourless oil (0.562 g, 84%).

1H NMR (400 MHz; $CDCl_3$): δ 7.40 (dd, *J* = 8.2 and 0.9 Hz, 1 H), 7.37-7.31 (m, 2 H), 7.18-7.14 (m, 2 H), 7.09 (s, 1 H), 7.07-7.03 (m, 1 H), 6.58 (d, *J* = 7.9 Hz, 1 H), 6.04 (br s, 1 H), 2.24 (s, 3 H).

13C NMR (100 MHz; $CDCl_3$): δ 141.7 (C), 141.0 (C), 138.2 (C), 132.5 (CH), 129.4 (CH), 122.5 (CH), 121.9 (CH), 120.2 (CH), 116.4 (CH), 109.0 (C), 21.3 (Me).

IR (ATR, cm^{-1}): 3396, 3043, 2920, 2856, 1590, 1572, 1507, 1495, 1467, 1427, 1407, 1304, 1227, 1172, 1155, 1131, 1077, 1021.

HRMS (ESI): Found MH^+, 262.0228. $C_{13}H_{13}{}^{79}BrN^+$ requires 262.0226. Δ = –0.7 ppm.

***N*-(2-Bromo-4-fluorophenyl)-2*H*-1,3-benzodioxol-5-amine S8**

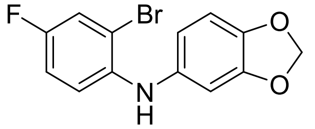

2-Bromo-4-fluoroaniline (0.939 g, 4.94 mmol), 6-(trimethylsilyl)-2*H*-1,3-benzodioxol-5-yl trifluoromethanesulfonate (1.41 g, 4.12 mmol) and CsF (1.88 g, 12.4 mmol) in MeCN (41 mL) were subjected to General Procedure 5. Purification by flash column chromatography, eluting with EtOAc/Hexane (0:1 to 1:19), afforded the title compound as a colourless oil (0.976 g, 76%).

1H NMR (400 MHz; $CDCl_3$): δ 7.27 (dd, *J* = 7.9 and 2.8 Hz, 1 H), 6.97 (dd, *J* = 9.0 and 5.3 Hz, 1 H), 6.88 (ddd, *J* = 8.7, 7.7 and 2.8 Hz, 1 H), 6.77 (d, *J* = 8.2 Hz, 1 H), 6.67 (d, *J* = 2.1 Hz, 1 H), 6.58 (dd, *J* = 8.2 and 2.1 Hz, 1 H), 5.97 (s, 2 H), 5.72 (br s, 1 H).

13C NMR (100 MHz; $CDCl_3$): δ 155.8 (d, *J* = 242 Hz, C), 148.4 (C), 143.9 (C), 139.4 (d, *J* = 2.6 Hz, C), 136.1 (C), 119.6 (d, *J* = 24.8 Hz, CH), 115.7 (d, *J* = 7.6 Hz, CH), 115.0 (d, *J* = 21.7 Hz, CH), 114.9 (CH), 110.8 (d, *J* = 10.7 Hz, C), 108.6 (CH), 104.1 (CH), 101.3 (CH_2).

IR (ATR, cm^{-1}): 3386, 2981, 2906, 1615, 1601, 1587, 1523, 1503, 1488, 1470, 1422, 1394, 1354, 1308, 1266, 1230, 1191, 1182, 1140, 1096, 1032.

HRMS (ESI): Found MH^+, 309.9877. $C_{13}H_{10}{}^{79}BrFNO_2{}^+$ requires 309.9873. Δ = –1.3 ppm.

General Procedure 6 – Methylation of 2-Bromo-*N*-phenylanilines

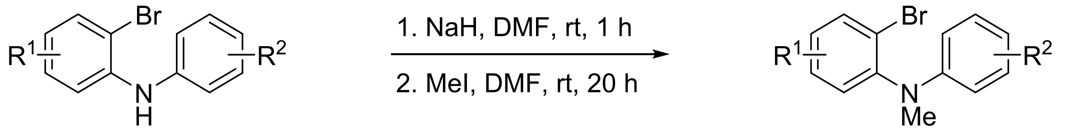

To a solution of the 2-bromo-*N*-phenylaniline (2.00 mmol) in DMF (10 mL) was added NaH (60% in mineral oil, 0.160 g, 4.00 mmol). The reaction mixture was stirred at rt for 1 h, then iodomethane (0.25 mL, 4.00 mmol) was added. Stirring was continued at rt for 20 h, then H_2O (50 mL) was added and the aqueous phase extracted with Et_2O (3 × 50 mL). The combined organics were washed with H_2O (2 × 50 mL), dried over $MgSO_4$, filtered, and concentrated *in vacuo*. Purification by flash column chromatography, eluting with EtOAc/Hexane, afforded the title compound.

2-Bromo-*N*-methyl-*N*-phenylaniline 21b

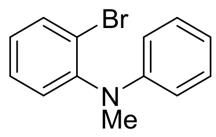

2-Bromo-*N*-phenylaniline **S6** (0.496 g, 2.00 mmol), NaH (0.160 g, 4.00 mmol) and iodomethane (0.25 mL, 4.00 mmol) in DMF (10 mL) were subjected to General Procedure 6. Purification by flash column chromatography, eluting with EtOAc/Hexane (0:1 to 1:19), afforded the title compound as a colourless oil (0.476 g, 91%).

1H NMR (400 MHz; $CDCl_3$): δ 7.71 (dd, *J* = 8.2 and 1.2 Hz, 1 H), 7.37 (td, *J* = 7.6 and 1.4 Hz, 1 H), 7.30 (dd, *J* = 7.9 and 1.6 Hz, 1 H), 7.26-7.20 (m, 2 H), 7.17 (td, *J* = 7.5 and 1.8 Hz, 1 H), 6.80 (td, *J* = 7.4 and 0.9 Hz, 1 H), 6.64-6.60 (m, 2 H), 3.27 (s, 3 H).

13C NMR (100 MHz; $CDCl_3$): δ 148.5 (C), 146.9 (C), 134.1 (CH), 130.4 (CH), 128.9 (2 × CH), 127.7 (CH), 124.2 (C), 117.7 (CH), 113.3 (CH), 38.9 (Me).

IR (ATR, cm^{-1}): 2981, 2884, 1600, 1579, 1497, 1480, 1466, 1452, 1437, 1344, 1300, 1272, 1261, 1242, 1187, 1156, 1118, 1091, 1071, 1039, 1027.

Data is consistent with literature values.[11]

[11] T. L. Andrew and T. M. Swager *J. Org. Chem.* **2011**, *76*, 2976-2993.

2-Bromo-*N*,5-dimethyl-*N*-phenylaniline 21c

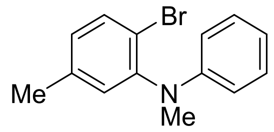

2-Bromo-5-methyl-*N*-phenylaniline **S7** (0.472 g, 1.80 mmol), NaH (0.144 g, 3.60 mmol) and iodomethane (0.22 mL, 3.60 mmol) in DMF (9 mL) were subjected to General Procedure 6. Purification by flash column chromatography, eluting with EtOAc/Hexane (0:1 to 1:19), afforded the title compound as a colourless oil (0.488 g, 98%).

1H NMR (400 MHz; $CDCl_3$): δ 7.54 (d, *J* = 8.2 Hz, 1 H), 7.21 (d, *J* = 7.2 Hz, 1 H), 7.19 (d, *J* = 7.3 Hz, 1 H), 7.09 (d, *J* = 1.9 Hz, 1 H), 6.96 (dd, *J* = 8.1 and 2.0 Hz, 1 H), 6.78-6.74 (m, 1 H), 6.60-6.57 (m, 2 H), 3.23 (s, 3 H), 2.30 (s, 3 H).

13C NMR (100 MHz; $CDCl_3$): δ 148.6 (C), 146.5 (C), 139.2 (C), 133.7 (CH), 131.0 (CH), 128.9 (CH), 128.7 (CH), 120.7 (C), 117.5 (CH), 113.3 (CH), 38.9 (Me), 20.8 (Me).

IR (ATR, cm^{-1}): 3028, 2922, 2811, 1600, 1589, 1568, 1497, 1479, 1468, 1452, 1399, 1339, 1300, 1278, 1244, 1189, 1156, 1129, 1111, 1094, 1073, 1029.

HRMS (ESI): Found MH^+, 276.0383. $C_{14}H_{15}{}^{79}BrN^+$ requires 276.0382. Δ = 0.0 ppm.

***N*-(2-Bromo-4-fluorophenyl)-*N*-methyl-2*H*-1,3-benzodioxol-5-amine 21d**

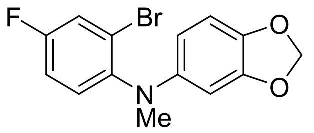

N-(2-Bromo-4-fluorophenyl)-2*H*-1,3-benzodioxol-5-amine **S8** (0.620 g, 2.00 mmol), NaH (0.160 g, 4.00 mmol) and iodomethane (0.25 mL, 4.00 mmol) in DMF (10 mL) were subjected to General Procedure 6. Purification by flash column chromatography, eluting with EtOAc/Hexane (0:1 to 1:19), afforded the title compound as a colourless oil (0.603 g, 93%).

1H NMR (400 MHz; $CDCl_3$): δ 7.41 (dd, *J* = 8.1 and 3.0 Hz, 1 H), 7.21 (dd, *J* = 8.8 and 5.7 Hz, 1 H), 7.06 (td, *J* = 7.7 and 2.8 Hz, 1 H), 6.69 (d, *J* = 8.4 Hz, 1 H), 6.18-6.16 (m, 1 H), 6.04 (dd, *J* = 8.6 and 2.0 Hz, 1 H), 5.87 (s, 2 H), 3.16 (s, 3 H).

13C NMR (100 MHz; $CDCl_3$): δ 160.2 (d, *J* = 250 Hz, C), 148.2 (C), 144.6 (C), 143.8 (d, *J* = 3.8 Hz, C), 140.0 (C), 130.7 (d, *J* = 8.6 Hz, CH), 124.4 (d, *J* = 10.2, C), 121.0 (d, *J* = 24.7 Hz, CH), 115.9 (d, *J* = 22.1 Hz, CH), 108.2 (CH), 105.6 (CH), 100.7 (CH_2), 97.0 (CH), 39.7 (Me).

IR (ATR, cm^{-1}): 2981, 2884, 1632, 1610, 1595, 1581, 1503, 1481, 1453, 1391, 1245, 1213, 1197, 1157, 1114, 1036.

HRMS (ESI): Found MH^+, 324.0037. $C_{14}H_{12}{}^{79}BrFNO_2^+$ requires 324.0030. Δ = –2.3 ppm.

1,2-Diethyl 2-(2-[ethyl(phenyl)amino]phenyl)propanedioate 15n

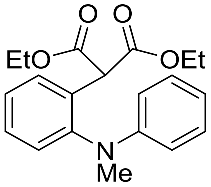

To a solution of 2-bromo-*N*-methyl-*N*-phenylaniline **21b** (52.0 mg, 0.200 mmol), $Pd_2dba_3{\cdot}CHCl_3$ (2.1 mg, 0.002 mmol), *t*-$Bu_3P{\cdot}HBF_4$ (2.3 mg, 0.008 mmol) and K_3PO_4 (0.127 g, 0.600 mmol) in toluene (1 mL) was added diethyl malonate (36.0 µL, 0.240 mmol). The reaction mixture was heated at reflux for 17 h. After cooling to rt, H_2O (10 mL) was added and the aqueous phase extracted with EtOAc (3 × 10 mL). The combined organics were dried over $MgSO_4$, filtered, and concentrated *in vacuo*. Purification by flash column chromatography, eluting with EtOAc/Hexane (0:1 to 1:19), afforded the title compound as a colourless oil (49.2 mg, 72%).

[The reaction may also be conducted in the same manner except using 2-iodo-*N*-methyl-*N*-phenylaniline **21a** as the starting material. In this case the yield is lowered to 61%]

1H NMR (400 MHz; $CDCl_3$): δ 7.62 (dd, *J* = 7.6 and 1.7 Hz, 1 H), 7.40 (td, *J* = 7.4 and 1.8 Hz, 1 H), 7.35 (td, *J* = 7.4 and 1.4 Hz, 1 H), 7.20-7.14 (m, 3 H), 6.75 (t, *J* = 7.3 Hz, 1 H), 6.56 (d, *J* = 8.5 Hz, 2 H), 5.04 (s, 1 H), 4.21-4.08 (m, 4 H), 3.19 (s, 3 H), 1.21 (t, *J* = 7.1 Hz, 6 H).

13C NMR (100 MHz; $CDCl_3$): δ 168.4 (C), 149.5 (C), 147.6 (C), 132.6 (C), 130.3 (CH), 130.1 (CH), 128.9 (CH), 128.3 (CH), 127.1 (CH), 117.8 (CH), 113.8 (CH), 61.6 (CH_2), 52.1 (CH), 39.8 (Me), 13.9 (Me).

IR (ATR, cm^{-1}): 2981, 2903, 1749, 1730, 1595, 1577, 1494, 1448, 1390, 1367, 1300, 1252, 1214, 1140, 1113, 1095, 1063, 1028.

HRMS (ESI): Found MNa^+, 364.1516. $C_{20}H_{23}NNaO_4^+$ requires 364.1519. Δ = 1.0 ppm.

General Procedure 7 – One-Pot α-Arylation/Cyclisation Approach to Acridanes

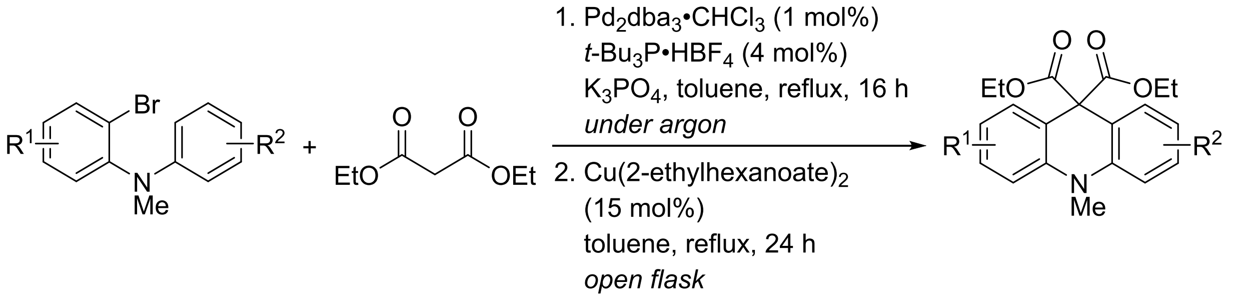

To a solution of the 2-bromo-*N*-methyl-*N*-arylaniline (0.500 mmol), $Pd_2dba_3 \cdot CHCl_3$ (5.2 mg, 0.005 mmol), *t*-$Bu_3P \cdot HBF_4$ (5.8 mg, 0.020 mmol) and K_3PO_4 (0.318 g, 1.50 mmol) in toluene (2.5 mL) was added diethyl malonate (91.0 µL, 0.600 mmol). The reaction mixture was heated at reflux for 16 h under argon. After cooling to rt, the argon balloon was removed and Cu(2-ethylhexanoate)$_2$ (26.2 mg, 0.075 mmol) was added, followed by further toluene (2.5 mL). The reaction mixture was heated at reflux for a further 24 h under an atmosphere of air. After cooling to rt, H_2O (35 mL) was added and the aqueous phase extracted with EtOAc (3 × 35 mL). The combined organics were washed with 10% NH_4OH (35 mL) and saturated brine (35 mL), dried over $MgSO_4$, filtered, and concentrated *in vacuo*. Purification by flash column chromatography, eluting with EtOAc/Hexane, afforded the title compound.

9,9-Diethyl 10-methyl-9,10-dihydroacridine-9,9-dicarboxylate 16n

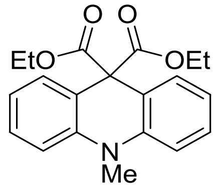

2-Bromo-*N*-methyl-*N*-phenylaniline **21b** (0.135 g, 0.515 mmol), diethyl malonate (94.0 µL, 0.618 mmol), $Pd_2dba_3 \cdot CHCl_3$ (5.3 mg, 0.0052 mmol), *t*-$Bu_3P \cdot HBF_4$ (6.0 mg, 0.021 mmol) and K_3PO_4 (0.328 g, 1.55 mmol) in toluene (2.5 mL), followed by Cu(2-ethylhexanoate)$_2$ (27.0 mg, 0.077 mmol) in further toluene (2.5 mL) were subjected to General Procedure 7. Purification by flash column chromatography, eluting with EtOAc/Hexane (0:1 to 1:19), afforded the title compound as a colourless solid (0.130 g, 74%).

MP: 114-116 °C.

1H NMR (400 MHz; CDCl$_3$): δ 7.35 (ddd, *J* = 8.6, 7.4 and 1.5 Hz, 2 H), 7.31 (dd, *J* = 7.8 and 1.4 Hz, 2 H), 7.05 (td, *J* = 7.3 and 0.8 Hz, 2 H), 7.00 (d, *J* = 8.2 Hz, 2 H), 4.28 (q, *J* = 7.0 Hz, 4 H), 3.40 (s, 3 H), 1.26 (t, *J* = 7.0 Hz, 6 H).

13C NMR (100 MHz; CDCl$_3$): δ 170.2 (C), 140.9 (C), 129.0 (CH), 128.5 (CH), 120.7 (C), 120.1 (CH), 112.5 (CH), 62.4 (C), 61.9 (CH_2), 33.2 (Me), 13.8 (Me).

IR (ATR, cm^{-1}): 2981, 2905, 1731, 1591, 1471, 1454, 1387, 1367, 1320, 1305, 1290, 1270, 1219, 1202, 1173, 1154, 1133, 1094, 1068, 1050, 1039, 1014.

HRMS (ESI): Found MNa^+, 362.1368. $C_{20}H_{21}NNaO_4^+$ requires 362.1363. Δ = –1.4 ppm.

9,9-Diethyl 3,10-dimethyl-9,10-dihydroacridine-9,9-dicarboxylate 16o

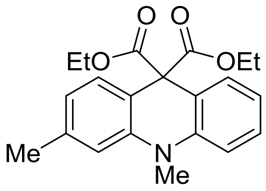

2-Bromo-*N*,5-dimethyl-*N*-phenylaniline **21c** (0.138 g, 0.515 mmol), diethyl malonate (91.0 µL, 0.600 mmol), $Pd_2dba_3 \cdot CHCl_3$ (5.2 mg, 0.005 mmol), *t*-$Bu_3P \cdot HBF_4$ (5.8 mg, 0.020 mmol) and K_3PO_4 (0.318 g, 1.50 mmol) in toluene (2.5 mL), followed by Cu(2-ethylhexanoate)$_2$ (26.2 mg, 0.075 mmol) in further toluene (2.5 mL) were subjected to General Procedure 7. Purification by flash column chromatography, eluting with EtOAc/Hexane (0:1 to 1:19), afforded the title compound as a colourless solid (0.120 g, 68%).

MP: 118-120 °C.

1H NMR (400 MHz; $CDCl_3$): δ 7.33 (ddd, *J* = 8.7, 7.3 and 1.8 Hz, 1 H), 7.28 (dd, *J* = 7.8 and 1.4 Hz, 1 H), 7.17 (d, *J* = 7.8 Hz, 1 H), 7.01 (td, *J* = 7.3 and 1.4 Hz, 1 H), 6.98 (d, *J* = 8.2 Hz, 1 H), 6.86 (d, *J* = 7.8 Hz, 1 H), 6.81 (br s, 1 H), 4.26 (q, *J* = 6.9 Hz, 4 H), 3.39 (s, 3 H), 2.41 (s, 3 H), 1.25 (t, *J* = 6.9 Hz, 6 H).

13C NMR (100 MHz; $CDCl_3$): δ 170.4 (C), 141.1 (C), 140.8 (C), 138.4 (C), 129.1 (CH), 128.9 (CH), 128.5 (CH), 121.1 (CH), 120.0 (CH), 117.9 (C), 113.3 (CH), 112.5 (CH), 62.1 (C), 61.9 (CH_2), 33.3 (Me), 21.6 (Me), 13.8 (Me).

IR (ATR, cm^{-1}): 2981, 2822, 1725, 1593, 1487, 1469, 1418, 1367, 1325, 1280, 1225, 1213, 1169, 1137, 1109, 1067, 1041, 1021.

HRMS (ESI): Found MNa^+, 376.1508. $C_{21}H_{23}NNaO_4^+$ requires 376.1519. Δ = 2.9 ppm.

10,10-Diethyl 8-fluoro-2*H*,5*H*,10*H*-[1,3]dioxolo[4,5-*b*]acridine-10,10-dicarboxylate 16p

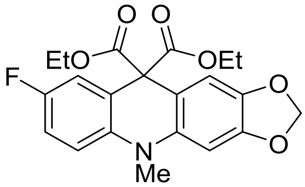

N-(2-Bromo-4-fluorophenyl)-*N*-methyl-2*H*-1,3-benzodioxol-5-amine **21d** (0.162 g, 0.500 mmol), diethyl malonate (91.0 µL, 0.600 mmol), $Pd_2dba_3 \cdot CHCl_3$ (5.2 mg, 0.005 mmol), *t*-$Bu_3P \cdot HBF_4$ (5.8 mg, 0.020 mmol) and K_3PO_4 (0.318 g, 1.50 mmol) in toluene (2.5 mL), followed by Cu(2-ethylhexanoate)$_2$ (26.2 mg, 0.075 mmol) in further toluene (2.5 mL) were subjected to General Procedure 7. Purification by flash column chromatography, eluting with EtOAc/Hexane (0:1 to 1:19), afforded the title compound as a colourless solid (0.106 g, 53%).

MP: 158-160 °C.

1H NMR (400 MHz; $CDCl_3$): δ 7.01 (ddd, *J* = 8.7, 8.0 and 2.8 Hz, 1 H), 6.96 (dd, *J* = 9.3 and 2.8 Hz, 1 H), 6.85 (dd, *J* = 8.8 and 4.5 Hz, 1 H), 6.72 (s, 1 H), 6.53 (s, 1 H), 5.94 (s, 2 H), 4.26 (q, *J* = 7.1 Hz, 4 H), 3.31 (s, 3 H), 1.25 (t, *J* = 7.1 Hz, 6 H).

13C NMR (100 MHz; $CDCl_3$): δ 169.8 (C), 157.1 (d, *J* = 238 Hz, C), 148.2 (C), 141.6 (C), 137.8 (d, *J* = 1.6 Hz, C), 136.9 (C), 121.7 (d, *J* = 7.2 Hz, C), 115.4 (d, *J* = 23.7 Hz, CH), 115.2 (d, *J* = 22.8 Hz, CH), 113.3 (d, *J* = 7.9 Hz, CH), 112.0 (C), 108.6 (CH), 101.2 (CH_2), 94.7 (CH), 62.4 (C), 62.2 (CH_2), 34.1 (Me), 13.9 (Me).

IR (ATR, cm^{-1}): 2981, 2891, 1726, 1630, 1503, 1485, 1469, 1443, 1432, 1382, 1365, 1212, 1166, 1133, 1119, 1093, 1033.

HRMS (ESI): Found MNa^+, 424.1163. $C_{21}H_{20}FNNaO_6^+$ requires 424.1167. Δ = 0.8 ppm.

6.0 Copies of 1H/13C NMR, Mass Spec, and IR Spectra

1-Phenylindolin-2-one 11a

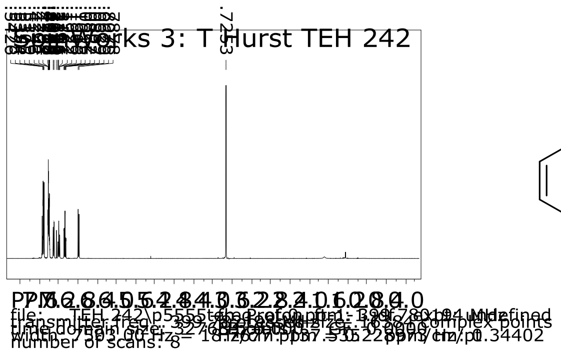

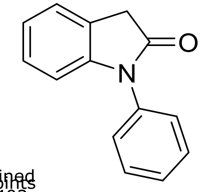

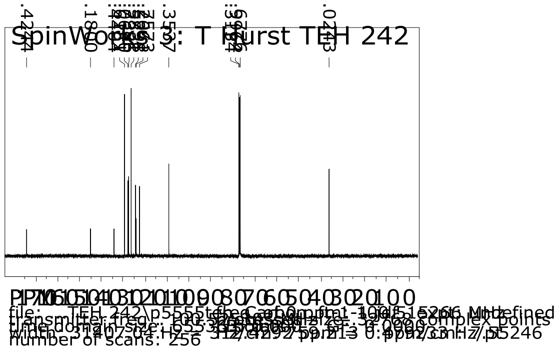
number of scans: 256

1-(4-Fluorophenyl)indolin-2-one 11b

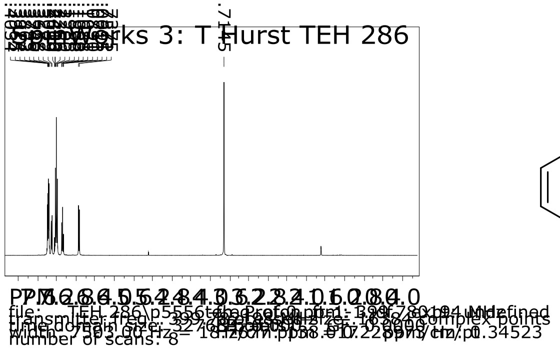

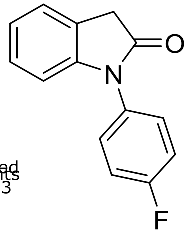

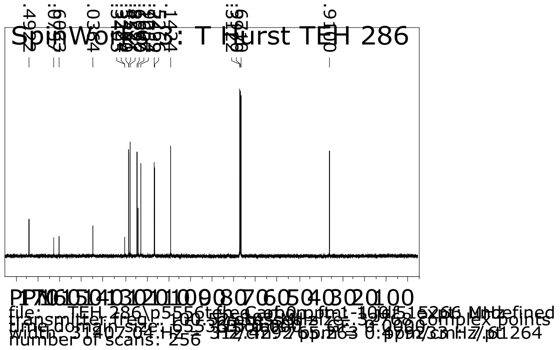
number of scans: 256

1-(3,5-Dichlorophenyl)indolin-2-one 11c

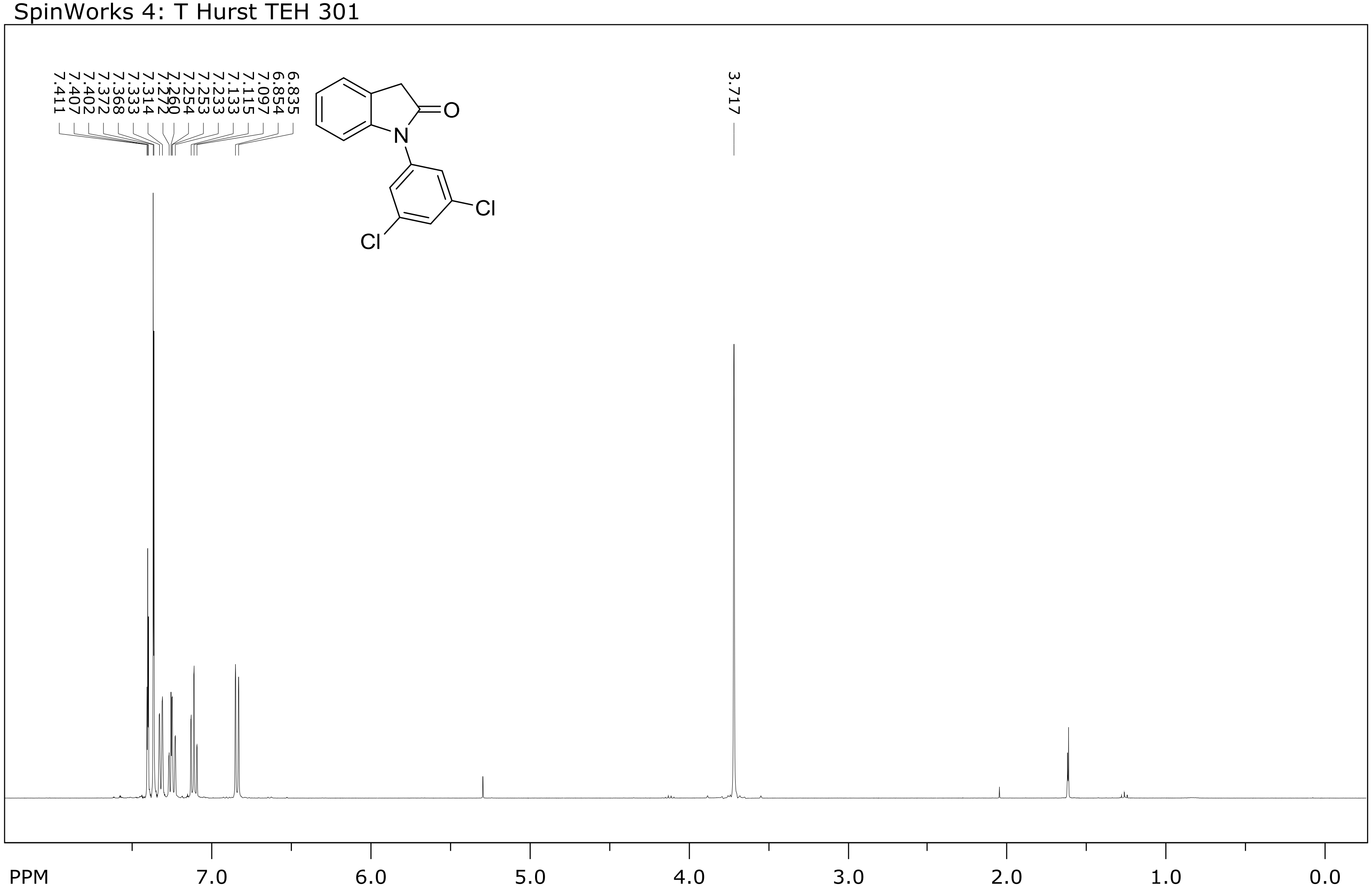

file: ...TEH 301\p5557teh_Proton_ft-1-1.jdf expt: undefined
transmitter freq.: 399.782198 MHz
time domain size: 32768 points
width: 7503.00 Hz = 18.7677 ppm = 0.228973 Hz/pt
number of scans: 8

freq. of 0 ppm: 399.780194 MHz
processed size: 16384 complex points
LB: 0.000 GF: 0.0000

SpinWorks 4: T Hurst TEH 301

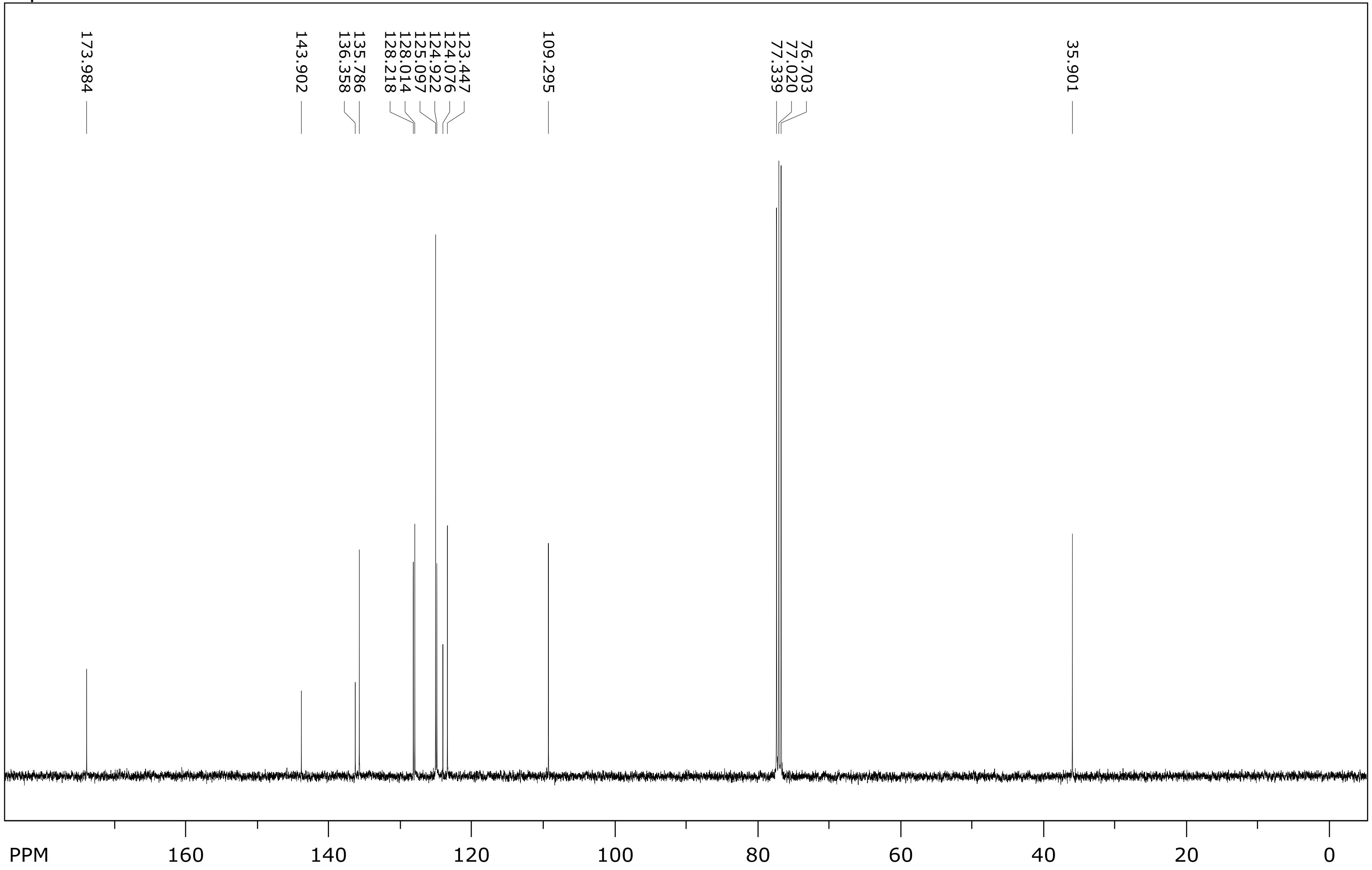

file: ...TEH 301\p5557teh_Carbon_ft-1-1.jdf   expt: undefined
transmitter freq.: 100.525303 MHz
time domain size: 65536 points
width: 31407.04 Hz = 312.4292 ppm = 0.479233 Hz/pt
number of scans: 256

freq. of 0 ppm: 100.515261 MHz
processed size: 32768 complex points
LB: 0.000   GF: 0.0000

teh301

York - Chemistry - Mass Spectrometry Service Report

Analysis Information

Acquisition Date 08/07/2015 09:49:41

Analysis Filename	rjkt53373th_P1-D-7_01_59657.d
Method	400p_meoh1260_2c1s.m
Submission Name	rjkt53373th
Instrument	micrOTOF
ESI	Positive

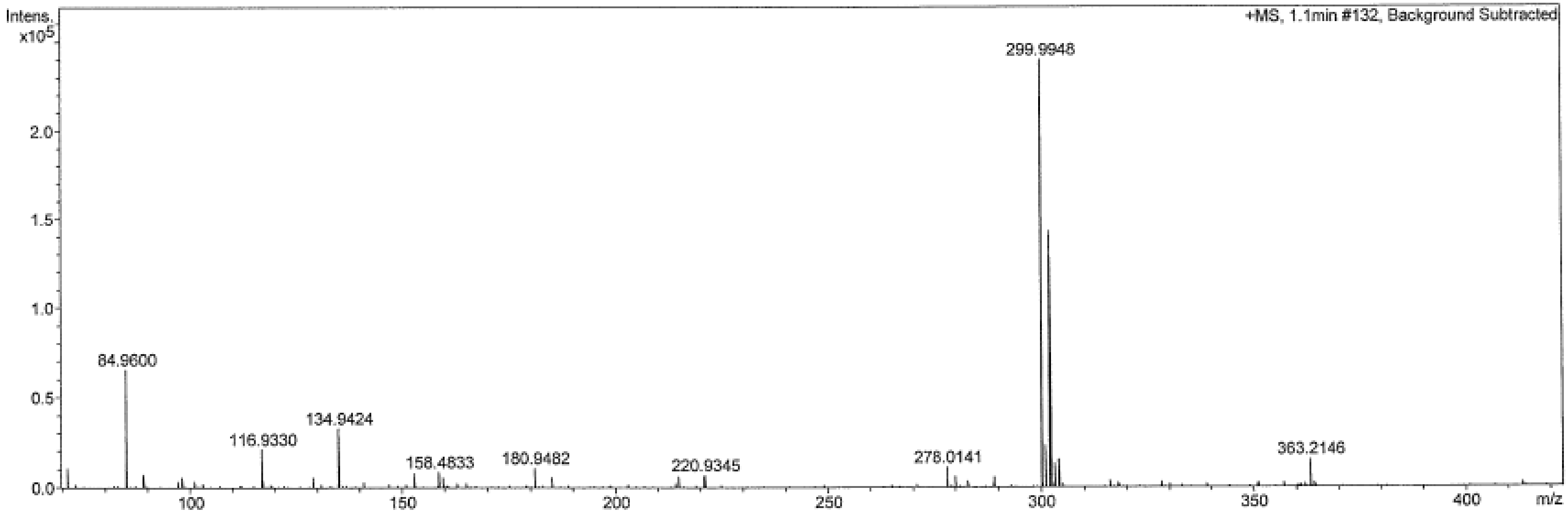

Meas. m/z	#	Formula	m/z	err [ppm]	err [mDa]	mSigma	Mean err [ppm]
299.9948	1	C 14 H 9 Cl 2 N Na O	299.9953	1.9	0.6	41.7	1.8

PerkinElmer Spectrum Version 10.03.07
01 July 2015 16:46

Analyst Administrator
Date 01 July 2015 16:46

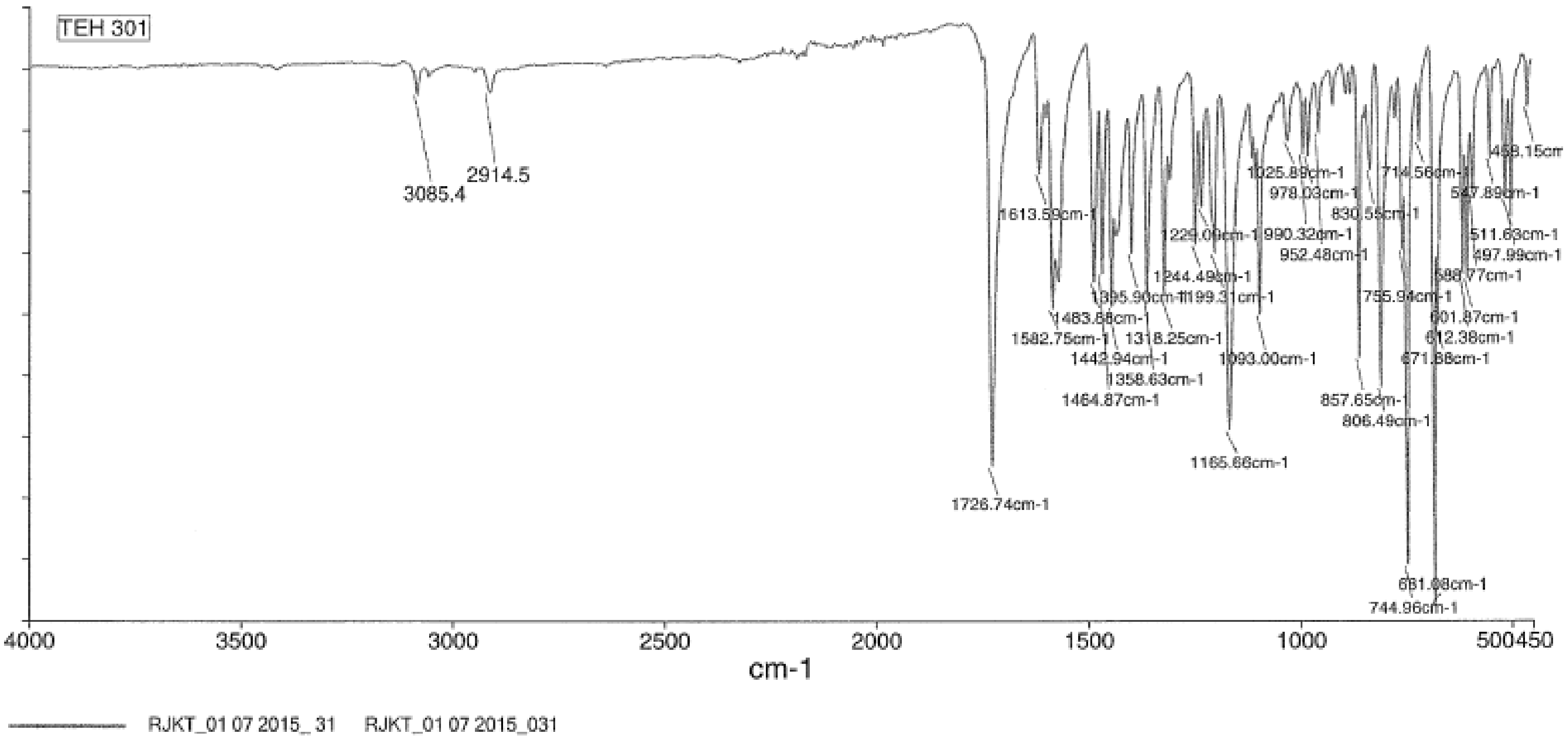

1-(3,5-Dimethylphenyl)indolin-2-one 11d

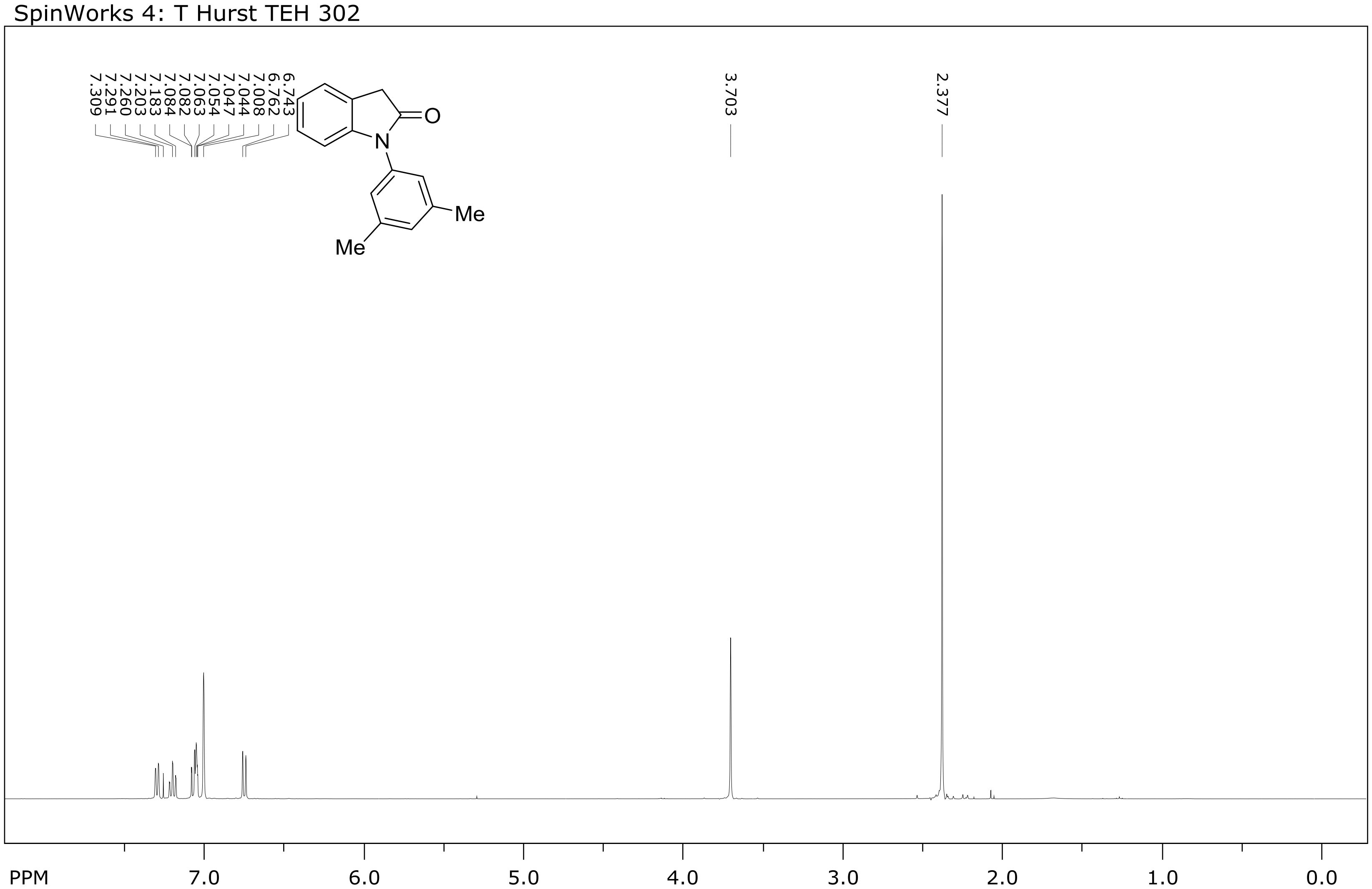

file: ...TEH 302\p5558teh_Proton_ft-1-1.jdf expt: undefined
transmitter freq.: 399.782198 MHz
time domain size: 32768 points
width: 7503.00 Hz = 18.7677 ppm = 0.228973 Hz/pt
number of scans: 8

freq. of 0 ppm: 399.780194 MHz
processed size: 16384 complex points
LB: 0.000 GF: 0.0000

SpinWorks 4: T Hurst TEH 302

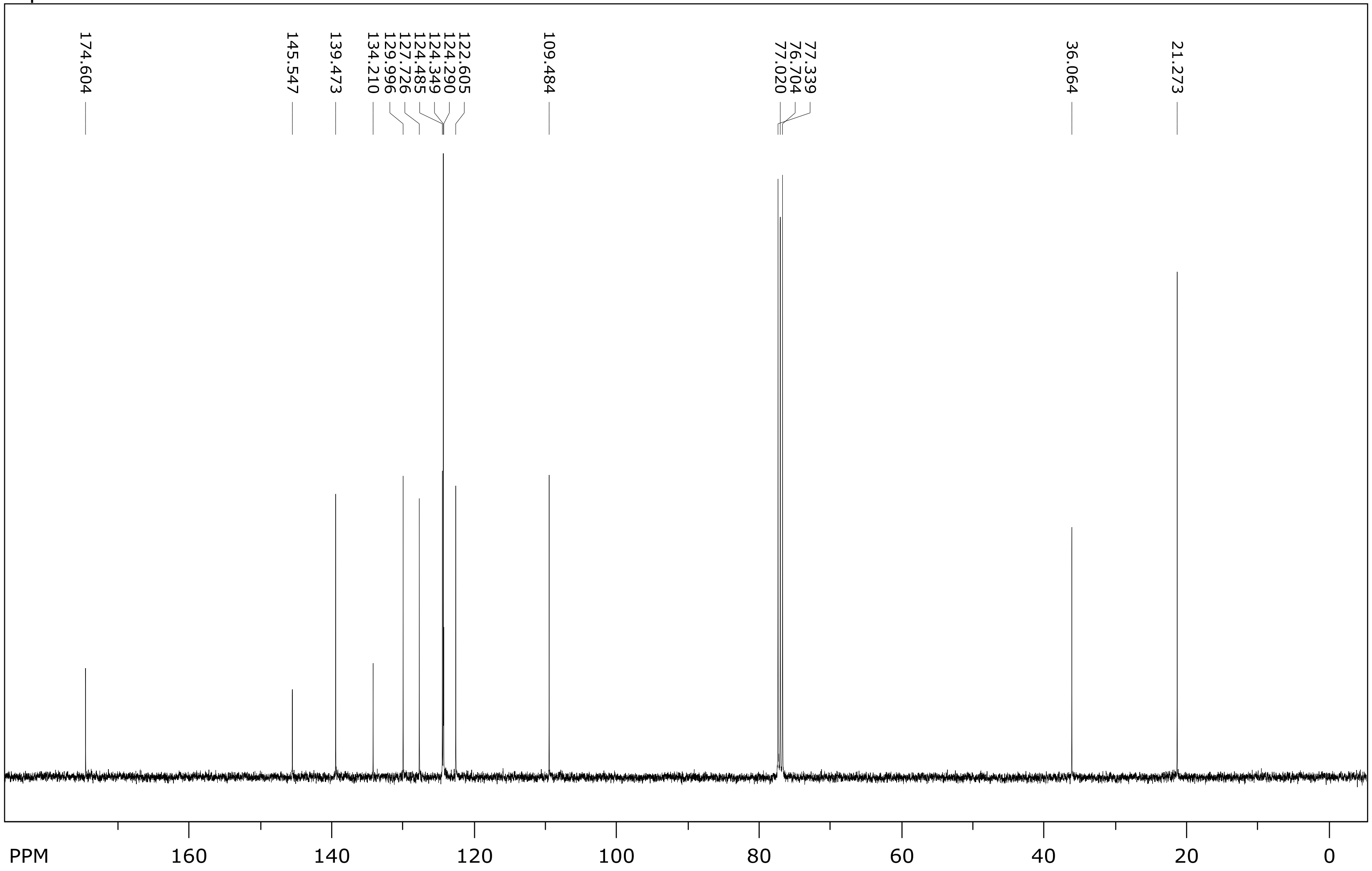

file: ...TEH 302\p5558teh_Carbon_ft-1-1.jdf expt: undefined
transmitter freq.: 100.525303 MHz
time domain size: 65536 points
width: 31407.04 Hz = 312.4292 ppm = 0.479233 Hz/pt
number of scans: 256

freq. of 0 ppm: 100.515263 MHz
processed size: 32768 complex points
LB: 0.000 GF: 0.0000

6-Chloro-1-(4-methoxyphenyl)indolin-2-one 11e

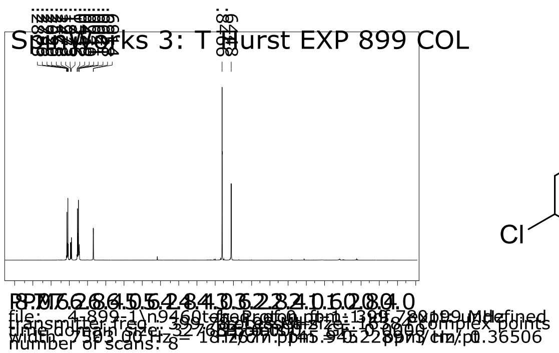

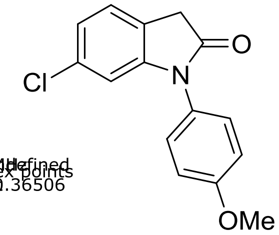

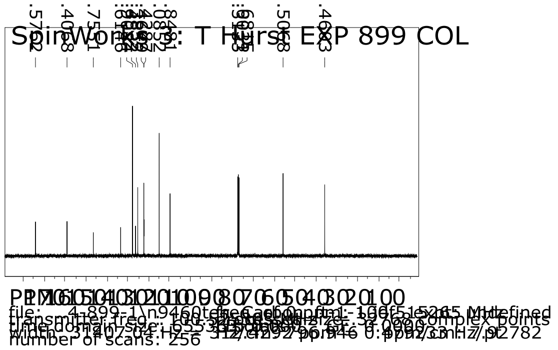
SpinWorks 3: T Hurst EXP 899 COL
number of scans: 256

teh334

York - Chemistry - Mass Spectrometry Service Report

Analysis Information

Acquisition Date 08/07/2015 09:52:36

Analysis Filename	rjkt53374th_P1-D-8_01_59658.d
Method	400p_meoh1260_2c1s.m
Submission Name	rjkt53374th
Instrument	micrOTOF
ESI	Positive

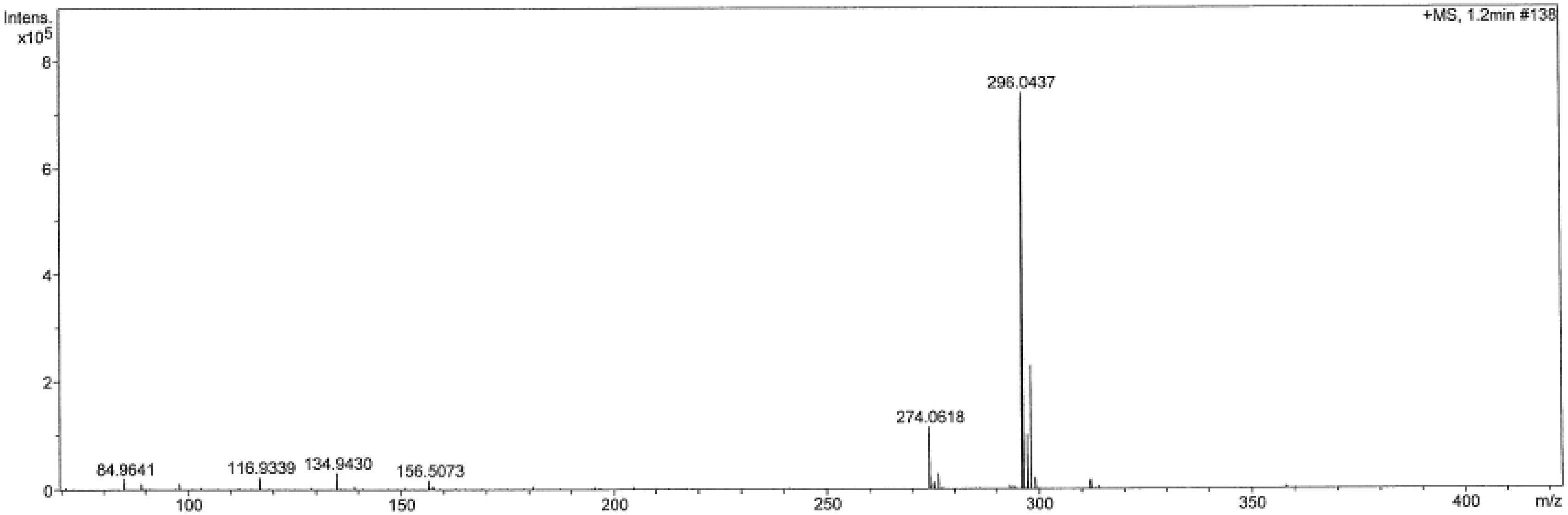

Meas. m/z	#	Formula	m/z	err [ppm]	err [mDa]	mSigma	Mean err [ppm]
274.0618	1	C 15 H 13 Cl N O 2	274.0629	4.1	1.1	46.7	3.6
296.0437	1	C 15 H 12 Cl N Na O 2	296.0449	4.1	1.2	21.5	3.7

PerkinElmer Spectrum Version 10.03.07
01 July 2015 16:50

Analyst	Administrator
Date	01 July 2015 16:50

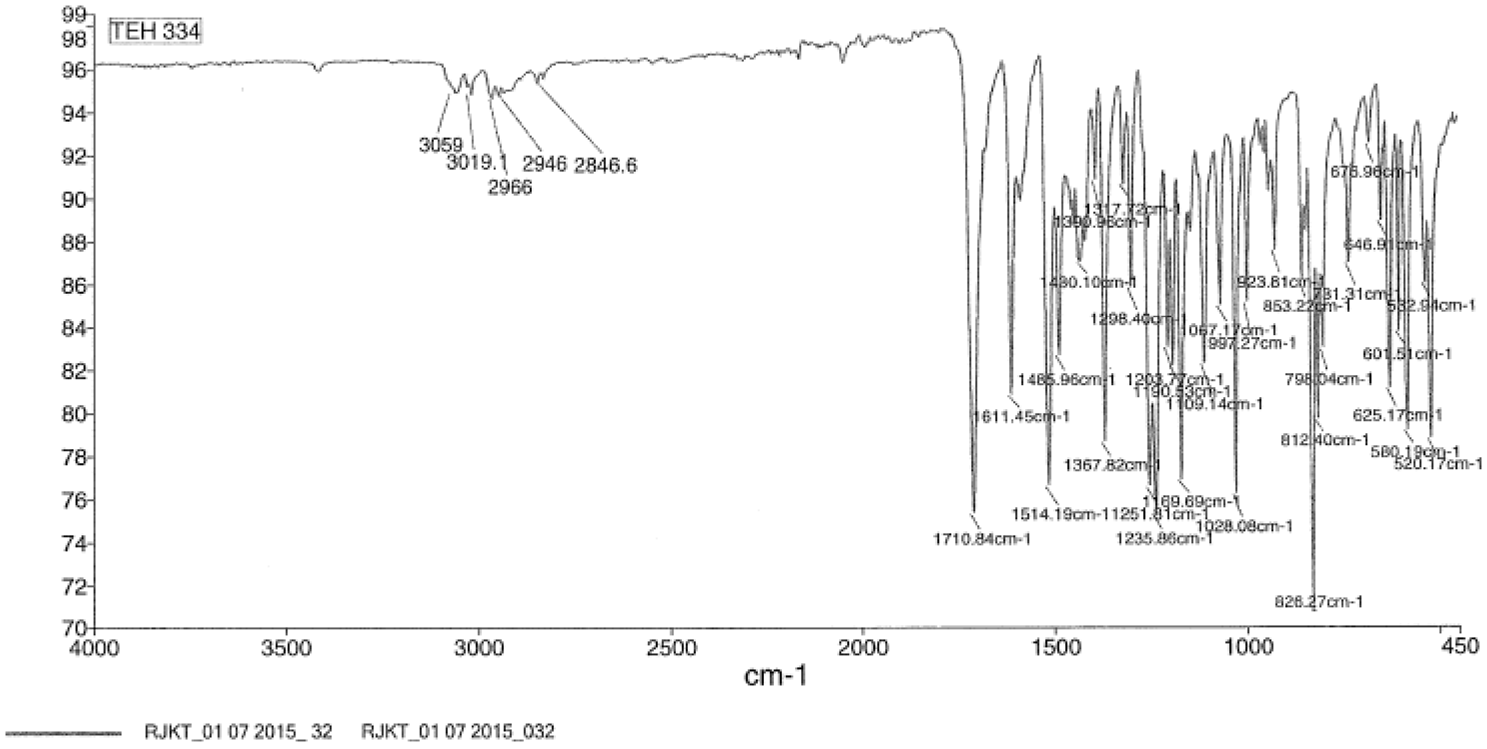

Ethyl 2-(2-[ethyl(phenyl)amino]phenyl)acetate 14a

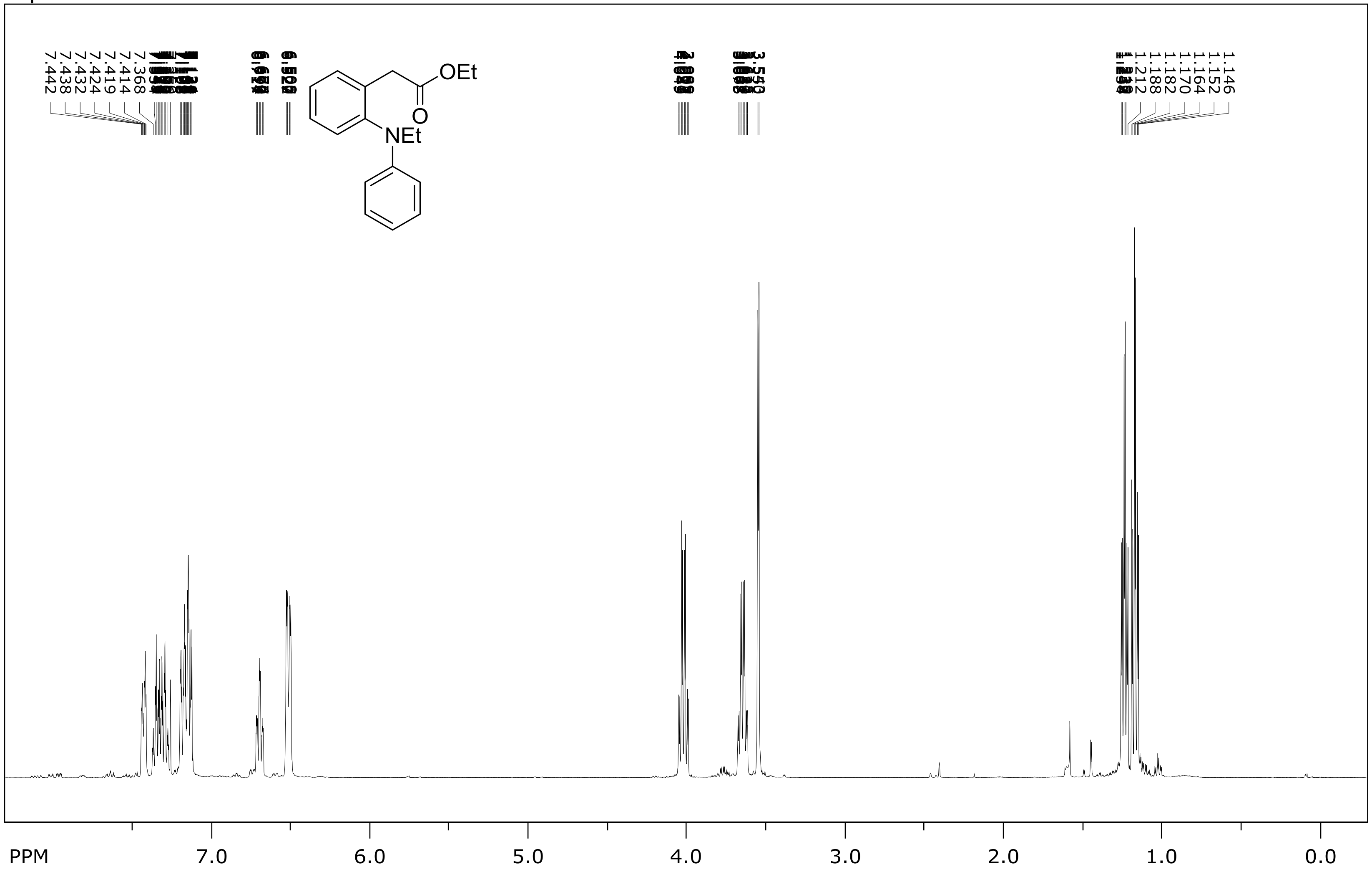

file: ...TEH 246\p6322teh_Proton_ft-1-1.jdf expt: undefined
transmitter freq.: 399.782198 MHz
time domain size: 32768 points
width: 7503.00 Hz = 18.7677 ppm = 0.228973 Hz/pt
number of scans: 8

freq. of 0 ppm: 399.780194 MHz
processed size: 16384 complex points
LB: 0.000 GF: 0.0000

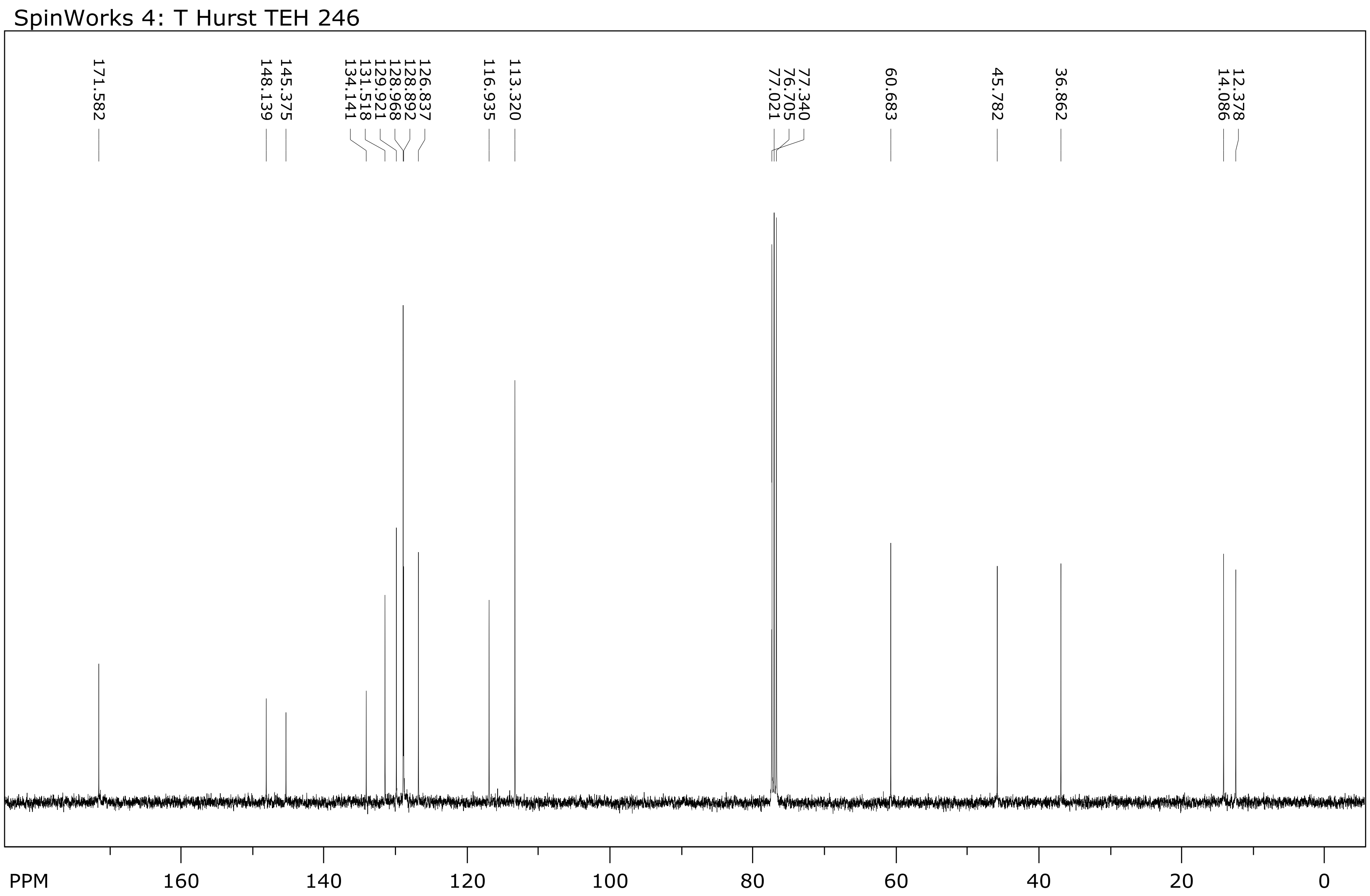

file: ...TEH 246\p6322teh_Carbon_ft-1-1.jdf expt: undefined
transmitter freq.: 100.525303 MHz
time domain size: 65536 points
width: 31407.04 Hz = 312.4292 ppm = 0.479233 Hz/pt
number of scans: 256

freq. of 0 ppm: 100.515261 MHz
processed size: 32768 complex points
LB: 0.000 GF: 0.0000

teh246

York - Chemistry - Mass Spectrometry Service Report

Analysis Information

Acquisition Date 08/07/2015 09:55:33

Analysis Filename	rjkt53375th_P1-D-9_01_59659.d
Method	400p_meoh1260_2c1s.m
Submission Name	rjkt53375th
Instrument	micrOTOF
ESI	Positive

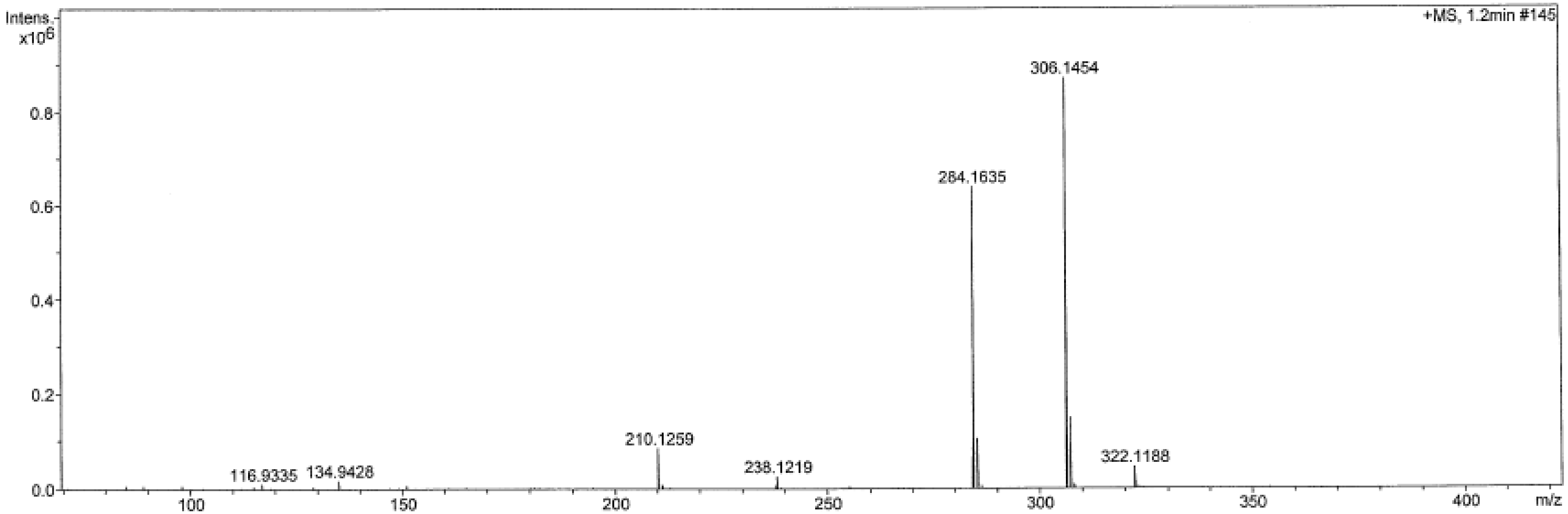

Meas. m/z	#	Formula	m/z	err [ppm]	err [mDa]	mSigma	Mean err [ppm]
284.1635	1	C 18 H 22 N O 2	284.1645	3.5	1.0	20.6	3.4
306.1454	1	C 18 H 21 N Na O 2	306.1465	3.4	1.0	16.8	3.2

PerkinElmer Spectrum Version 10.03.07
01 July 2015 16:53

Analyst Administrator
Date 01 July 2015 16:53

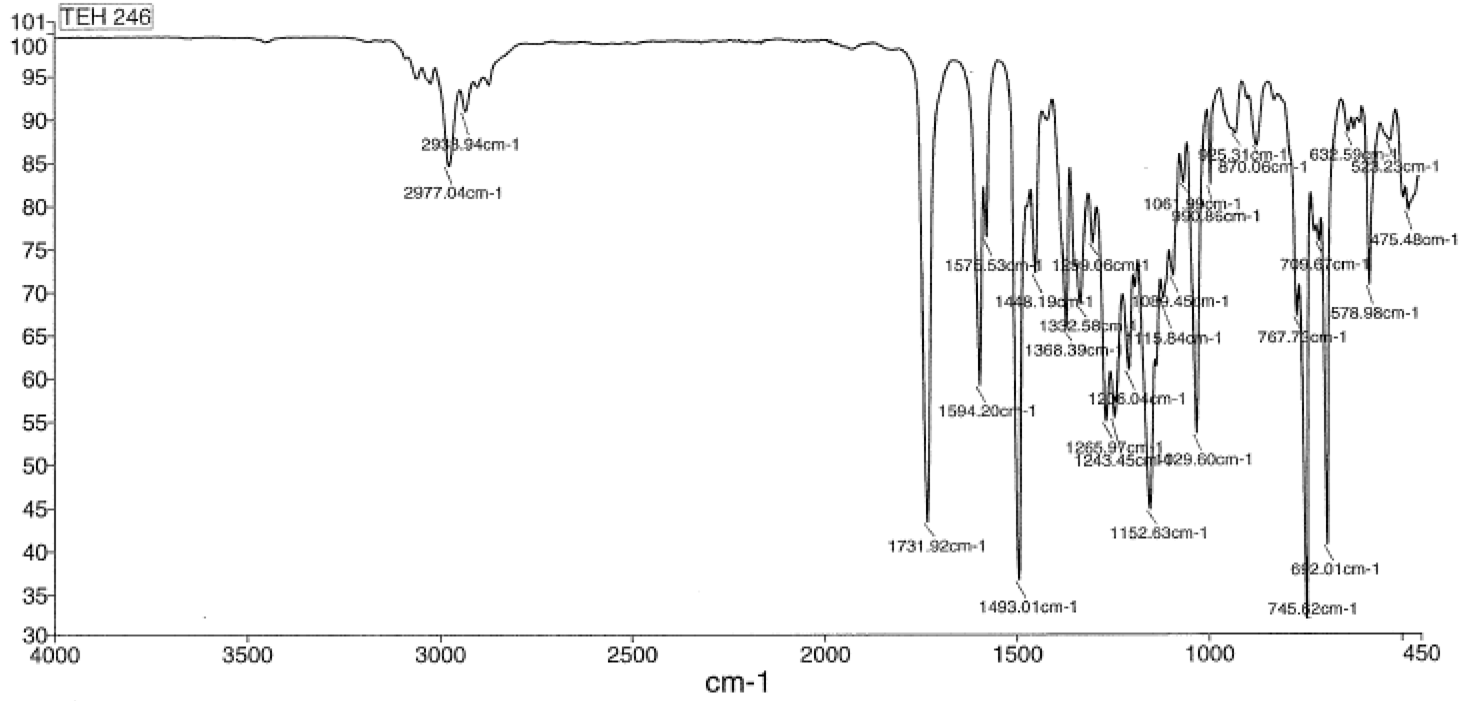

RJKT_01 07 2015_ 33 RJKT_01 07 2015_033

Ethyl 2-(2-[benzyl(phenyl)amino]phenyl)acetate 14b

SpinWorks 4: T Hurst TEH 283

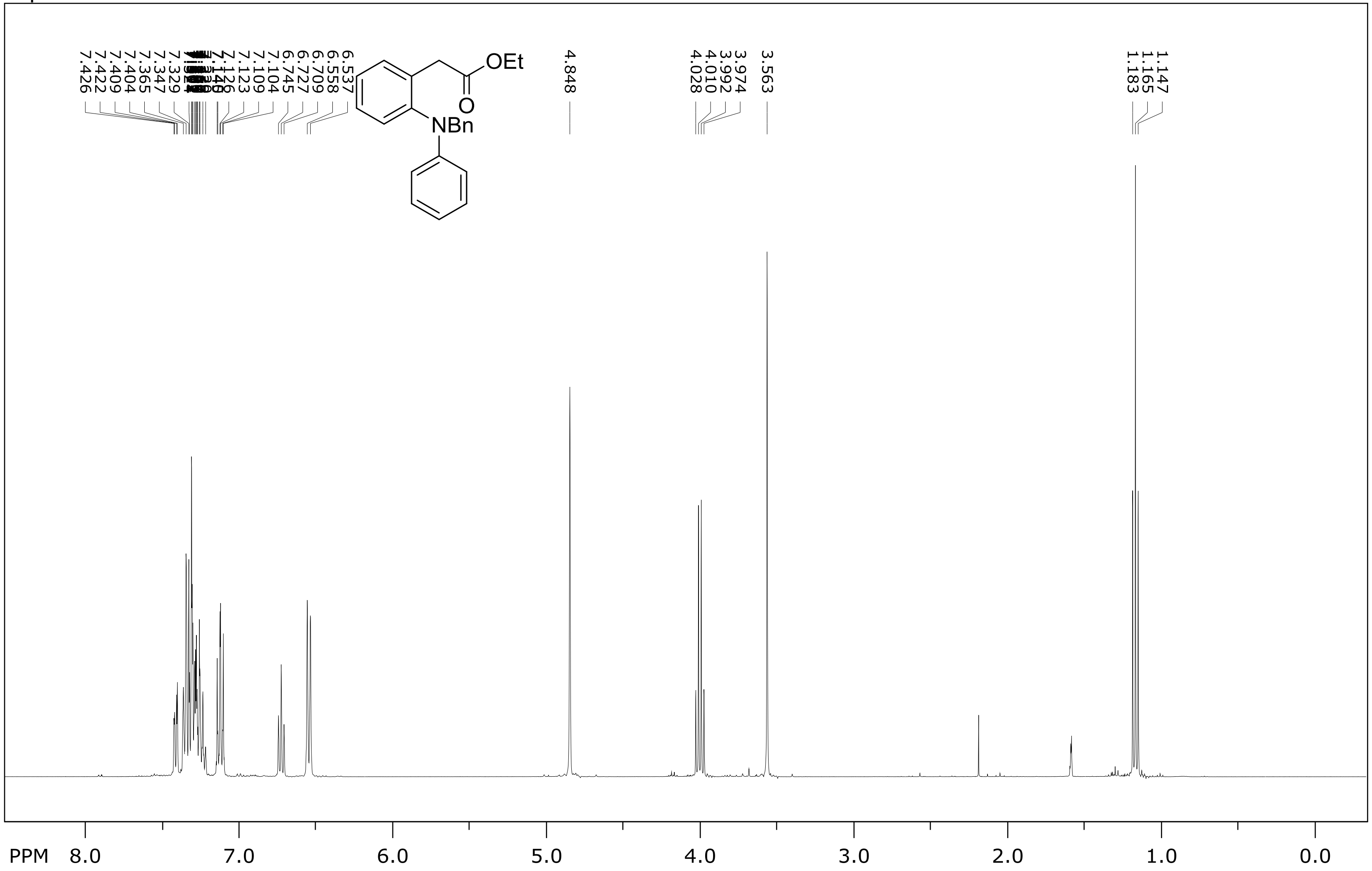

file: ...TEH 283\p5560teh_Proton_ft-1-1.jdf expt: undefined
transmitter freq.: 399.782198 MHz
time domain size: 32768 points
width: 7503.00 Hz = 18.7677 ppm = 0.228973 Hz/pt
number of scans: 8

freq. of 0 ppm: 399.780194 MHz
processed size: 16384 complex points
LB: 0.000 GF: 0.0000

SpinWorks 4: T Hurst TEH 283

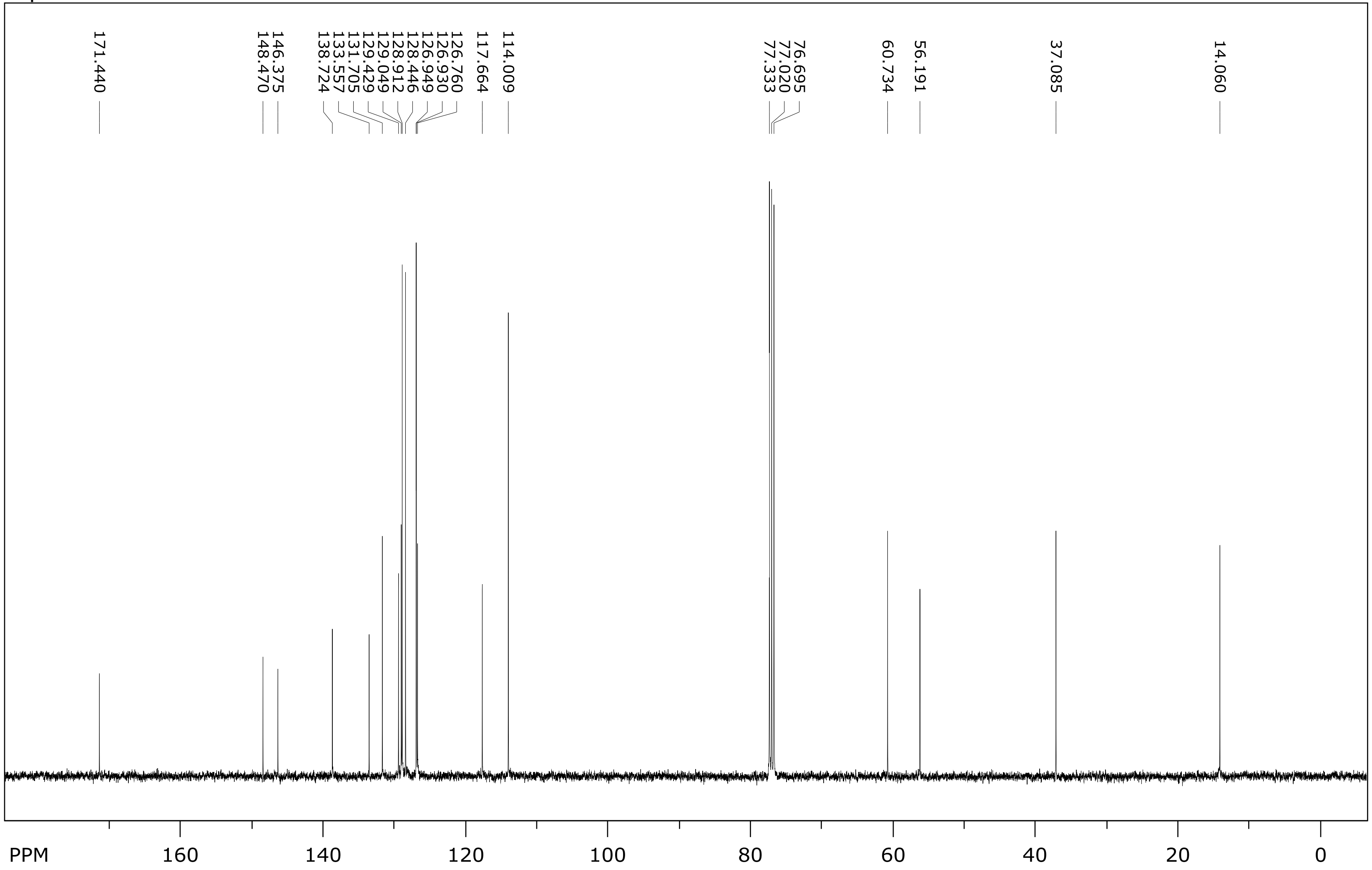

file: ...TEH 283\p5560teh_Carbon_ft-1-1.jdf expt: undefined
transmitter freq.: 100.525303 MHz
time domain size: 65536 points
width: 31407.04 Hz = 312.4292 ppm = 0.479233 Hz/pt
number of scans: 256

freq. of 0 ppm: 100.515263 MHz
processed size: 32768 complex points
LB: 0.000 GF: 0.0000

teh 283

York - Chemistry - Mass Spectrometry Service Report

Analysis Information

Acquisition Date 08/07/2015 09:58:30

Analysis Filename	rjkt53376th_P1-E-1_01_59660.d
Method	400p_meoh1260_2c1s.m
Submission Name	rjkt53376th
Instrument	micrOTOF
ESI	Positive

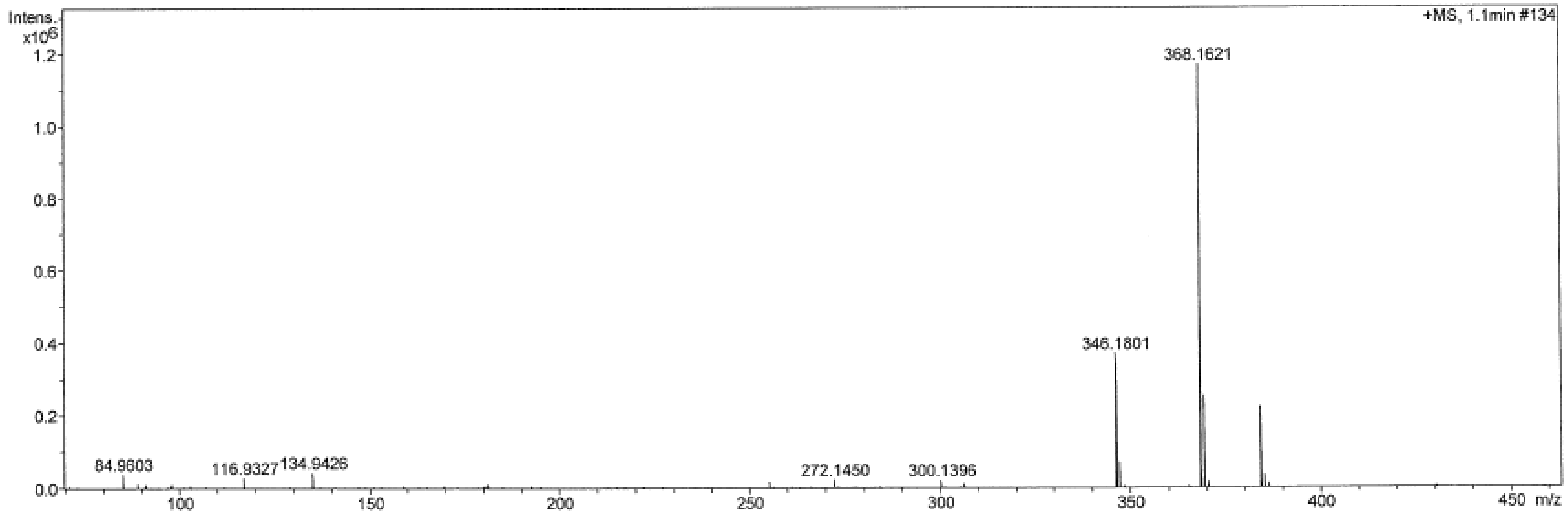

Meas. m/z	#	Formula	m/z	err [ppm]	err [mDa]	mSigma	Mean err [ppm]
346.1801	1	C 23 H 24 N O 2	346.1802	0.3	0.1	37.4	-0.1
368.1621	1	C 23 H 23 N Na O 2	368.1621	-0.1	-0.0	23.3	0.5

PerkinElmer Spectrum Version 10.03.07
01 July 2015 16:55

Analyst	Administrator
Date	01 July 2015 16:55

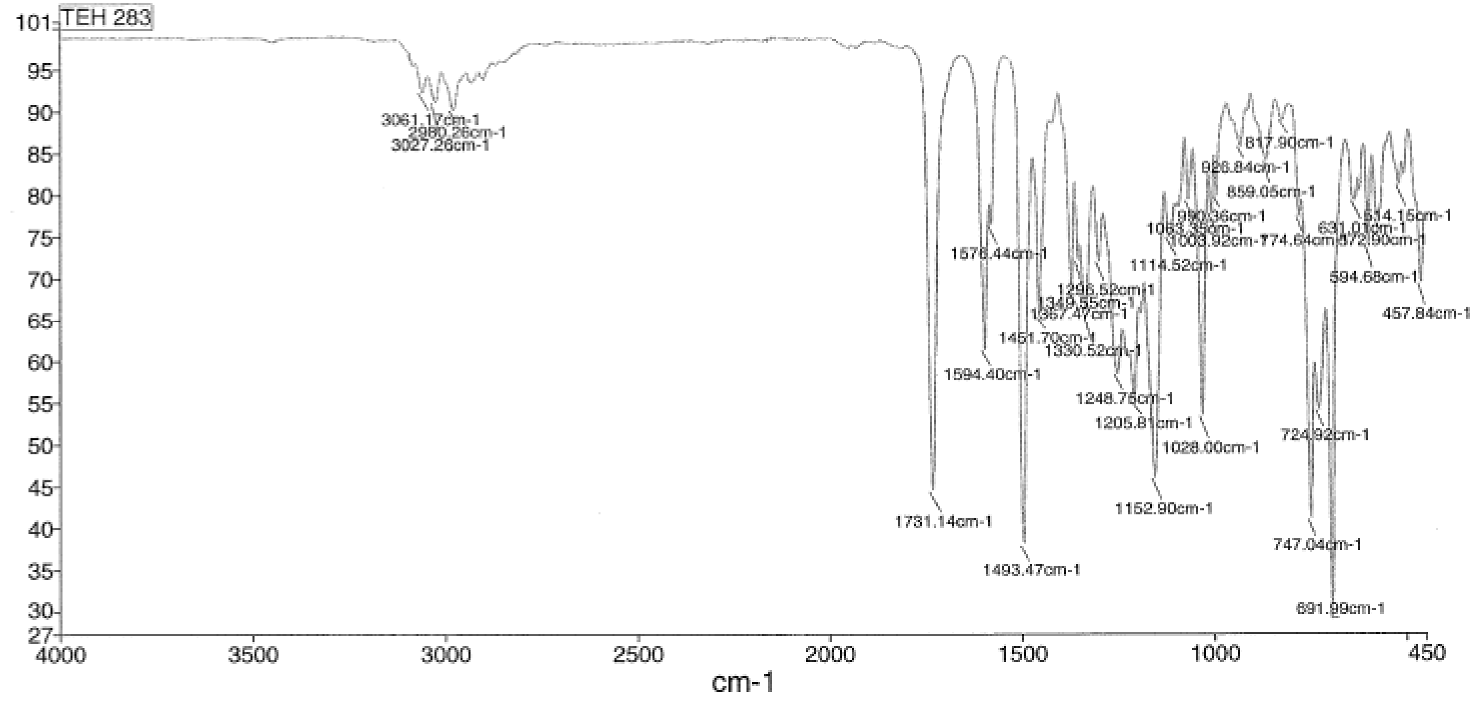

RJKT_01 07 2015_ 34 RJKT_01 07 2015_034

Ethyl 2-(2-[ethyl(4-fluorophenyl)amino]phenyl)acetate 14c

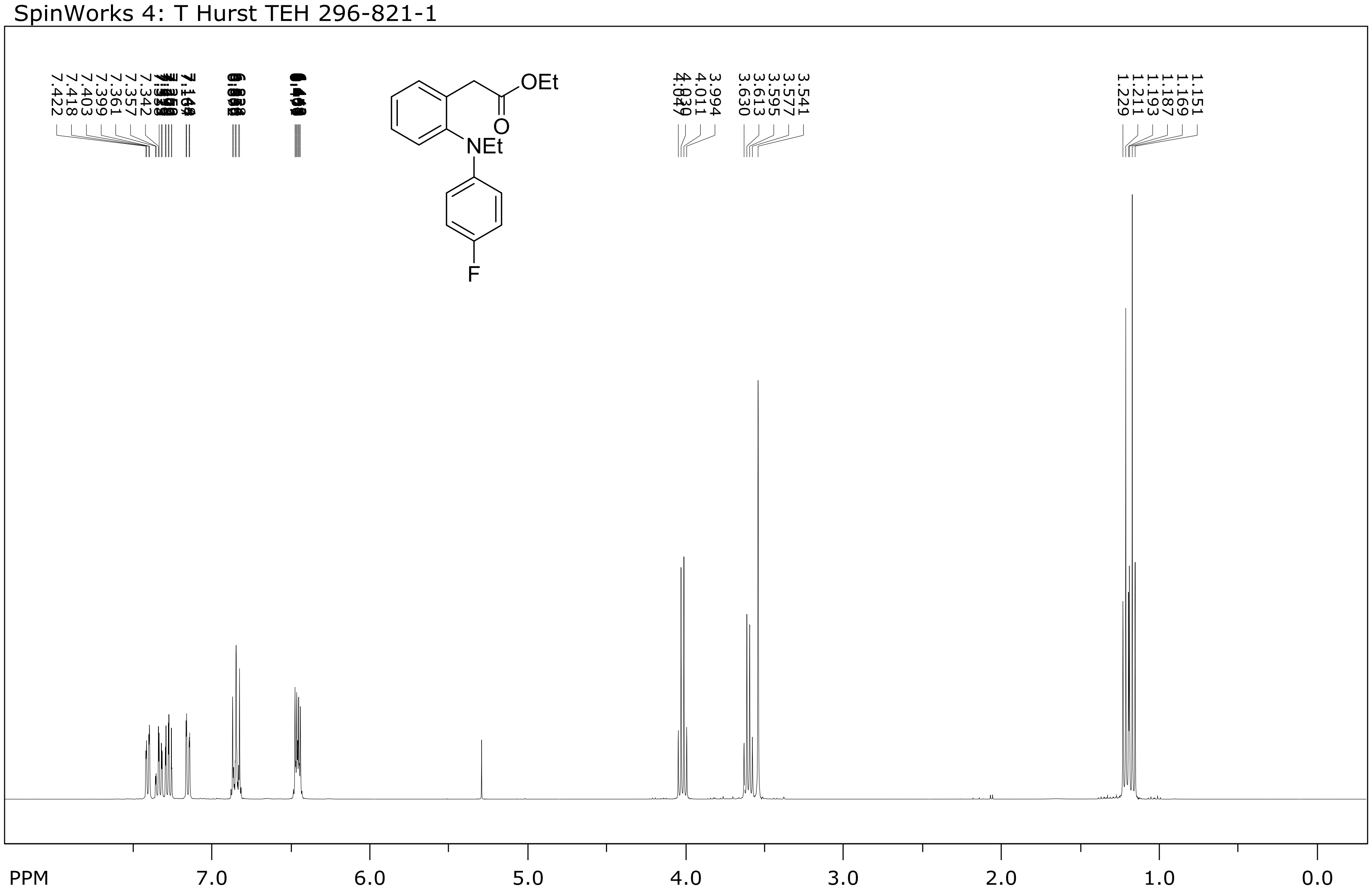

file: ...6-821-1\n5700teh_Proton_ft-1-1.jdf expt: undefined
transmitter freq.: 399.782198 MHz
time domain size: 32768 points
width: 7503.00 Hz = 18.7677 ppm = 0.228973 Hz/pt
number of scans: 8

freq. of 0 ppm: 399.780194 MHz
processed size: 16384 complex points
LB: 0.000 GF: 0.0000

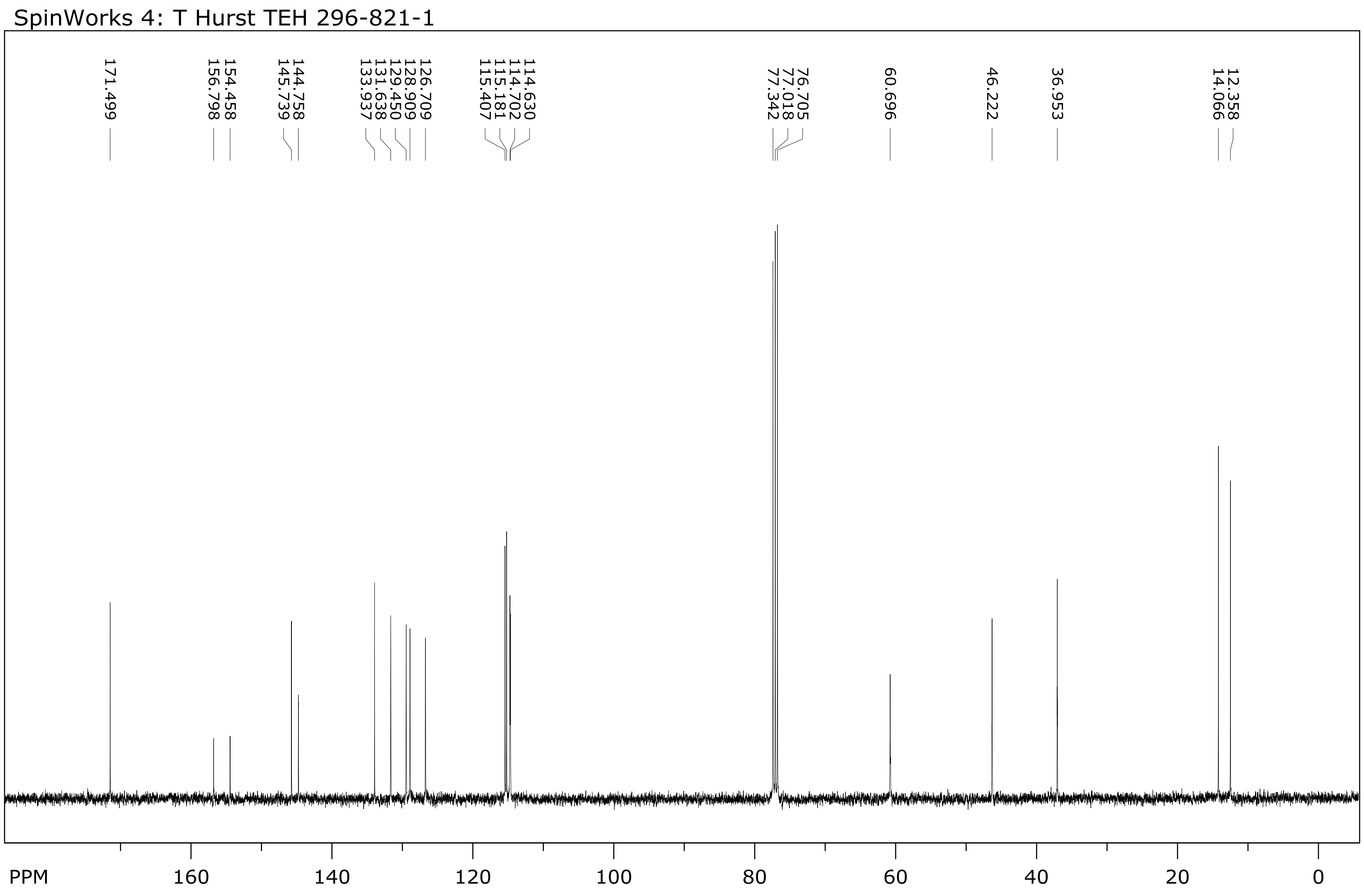

file: ...6-821-1\n5700teh_Carbon_ft-1-1.jdf expt: undefined
transmitter freq.: 100.525303 MHz
time domain size: 65536 points
width: 31407.04 Hz = 312.4292 ppm = 0.479233 Hz/pt
number of scans: 256

freq. of 0 ppm: 100.515263 MHz
processed size: 32768 complex points
LB: 0.000 GF: 0.0000

teh 296

York - Chemistry - Mass Spectrometry Service Report

Analysis Information Acquisition Date 08/07/2015 10:01:26

Analysis Filename	rjkt53377th_P1-E-2_01_59661.d
Method	400p_meoh1260_2c1s.m
Submission Name	rjkt53377th
Instrument	micrOTOF
ESI	Positive

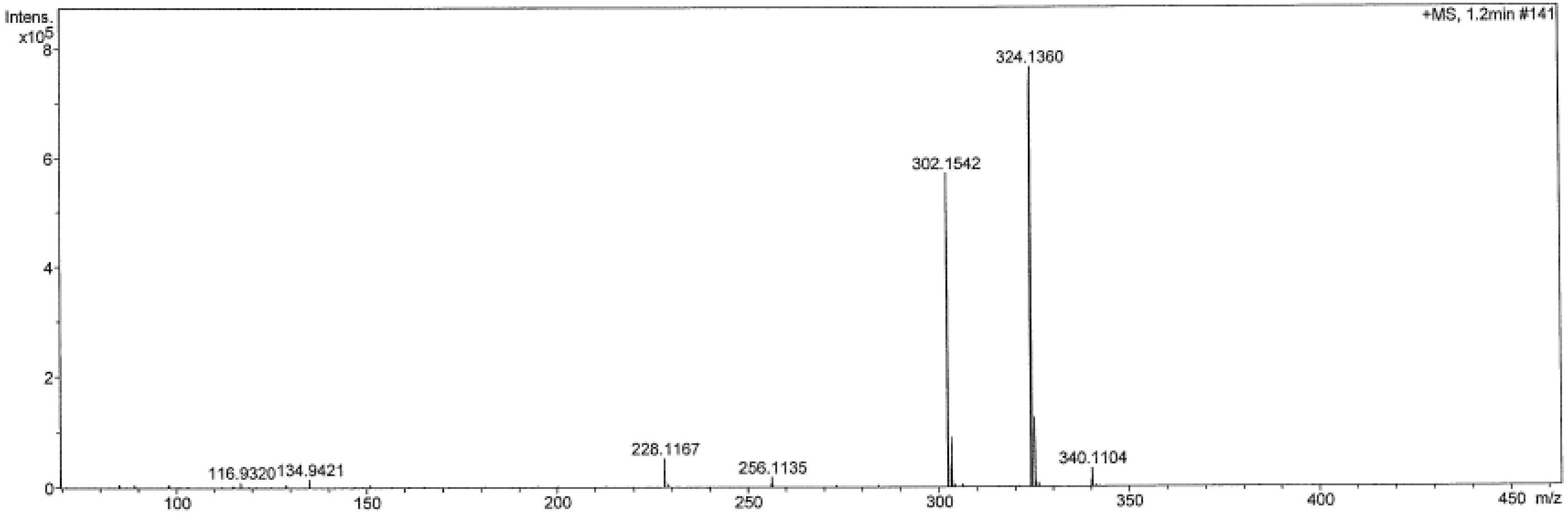

Meas. m/z	#	Formula	m/z	err [ppm]	err [mDa]	mSigma	Mean err [ppm]
302.1542	1	C 18 H 21 F N O 2	302.1551	2.8	0.8	24.1	2.6
324.1360	1	C 18 H 20 F N Na O 2	324.1370	3.2	1.0	21.2	2.9

PerkinElmer Spectrum Version 10.03.07
01 July 2015 16:57

Analyst Administrator
Date 01 July 2015 16:57

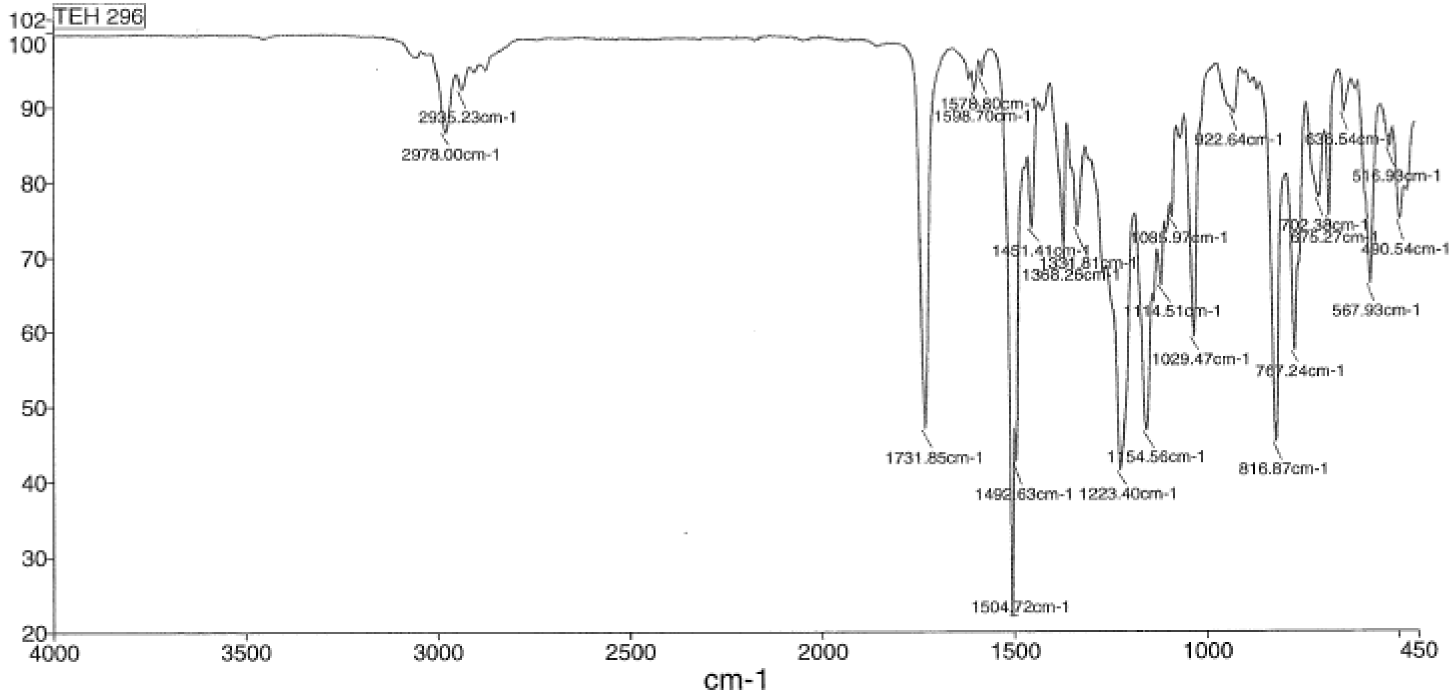

RJKT_01 07 2015_ 35 RJKT_01 07 2015_035

Ethyl 2-(2-[ethyl(3,5-dichlorophenyl)amino]phenyl)acetate 14d

SpinWorks 4: T Hurst TEH 307

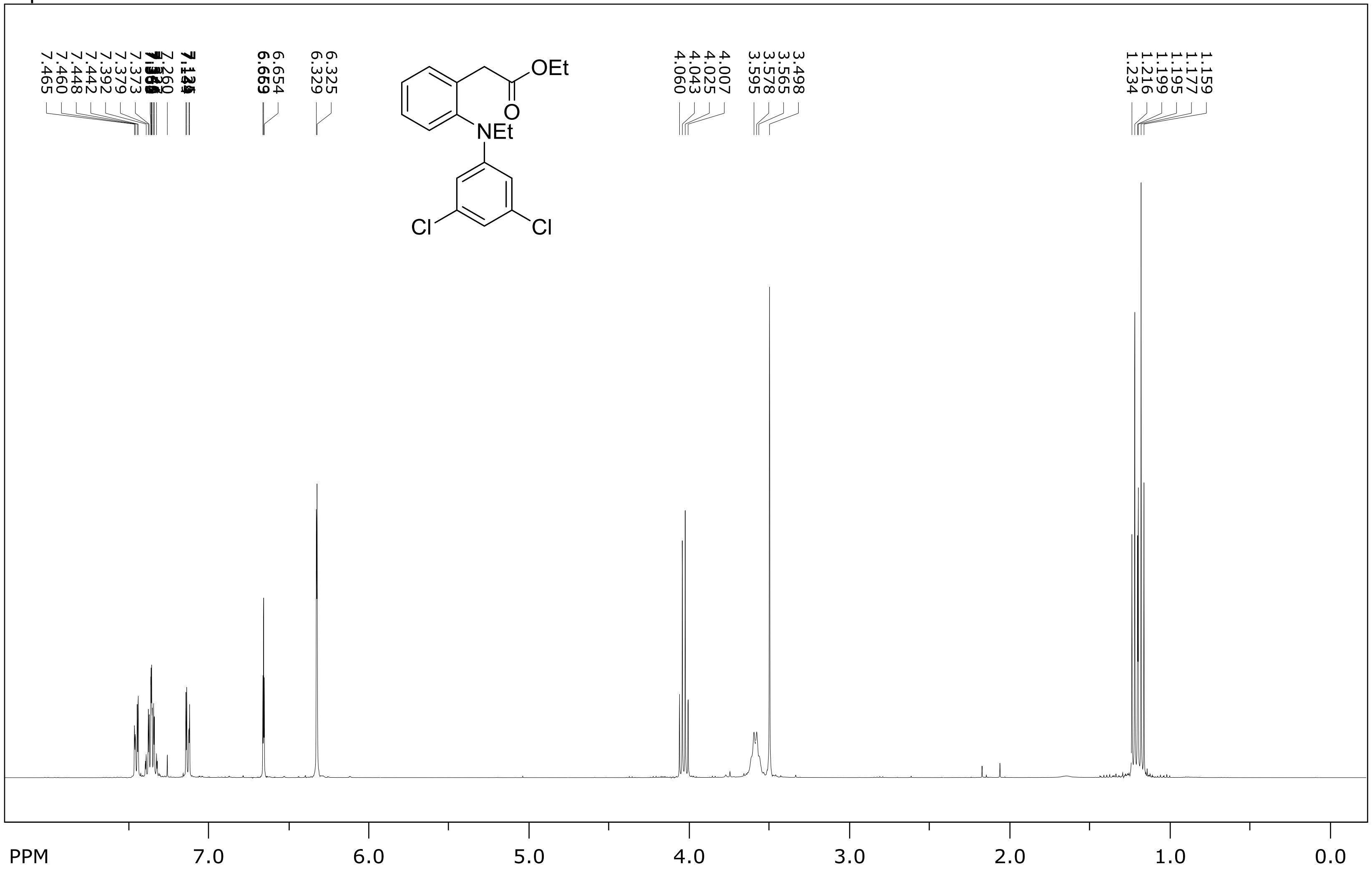

file: ...TEH 307\p5561teh_Proton_ft-1-1.jdf   expt: undefined
transmitter freq.: 399.782198 MHz
time domain size: 32768 points
width: 7503.00 Hz = 18.7677 ppm = 0.228973 Hz/pt
number of scans: 8

freq. of 0 ppm: 399.780194 MHz
processed size: 16384 complex points
LB: 0.000   GF: 0.0000

SpinWorks 4: T Hurst TEH 307

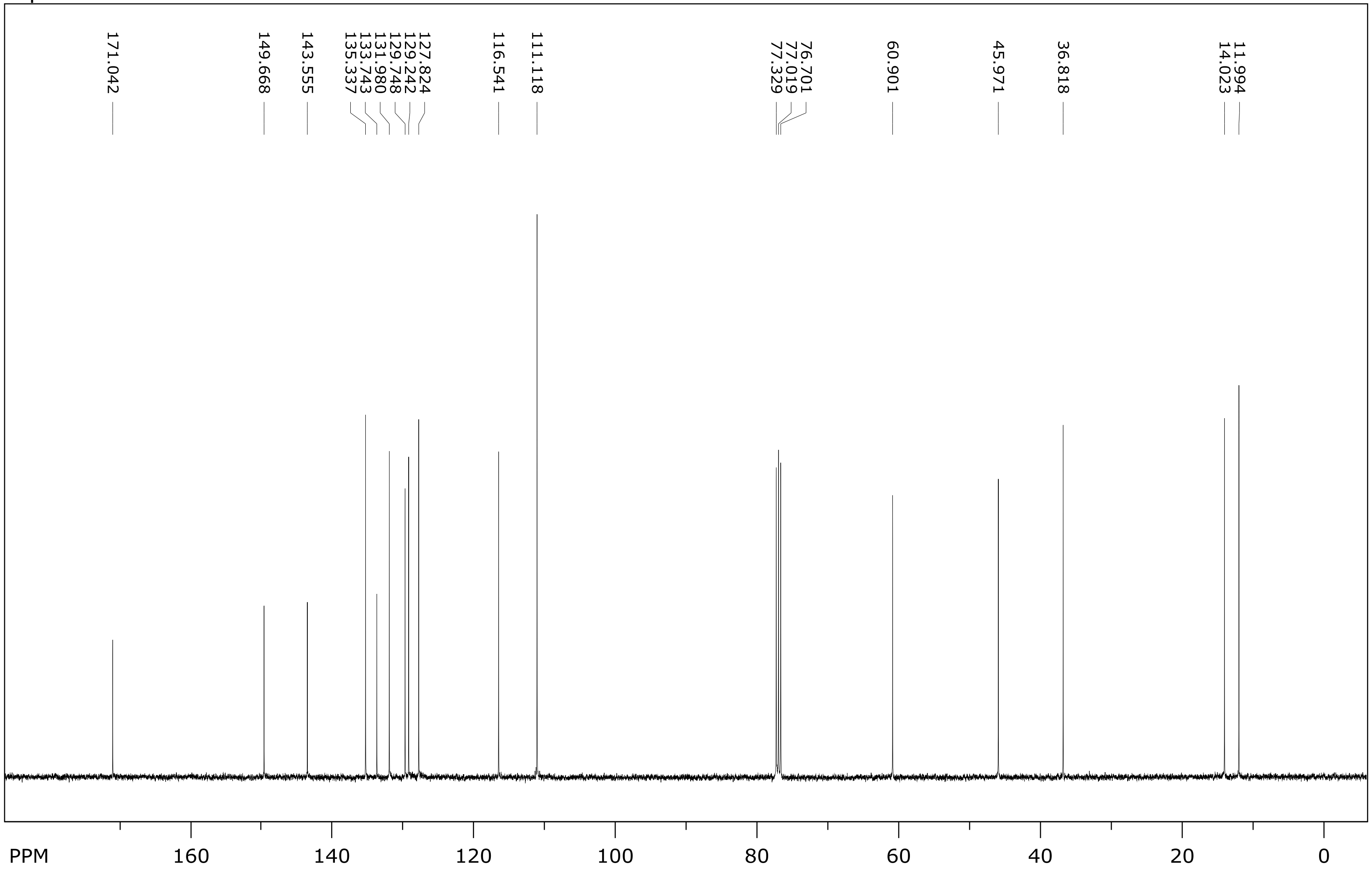

file: ...TEH 307\p5561teh_Carbon_ft-1-1.jdf expt: undefined
transmitter freq.: 100.525303 MHz
time domain size: 65536 points
width: 31407.04 Hz = 312.4292 ppm = 0.479233 Hz/pt
number of scans: 256

freq. of 0 ppm: 100.515264 MHz
processed size: 32768 complex points
LB: 0.000 GF: 0.0000

teh307

York - Chemistry - Mass Spectrometry Service Report

Analysis Information

Acquisition Date 09/07/2015 16:51:53

Analysis Filename	rjkt53495th_P1-C-9_01_59715.d
Method	400p_meoh1260_2c1s.m
Submission Name	rjkt53495th
Instrument	micrOTOF
ESI	Positive

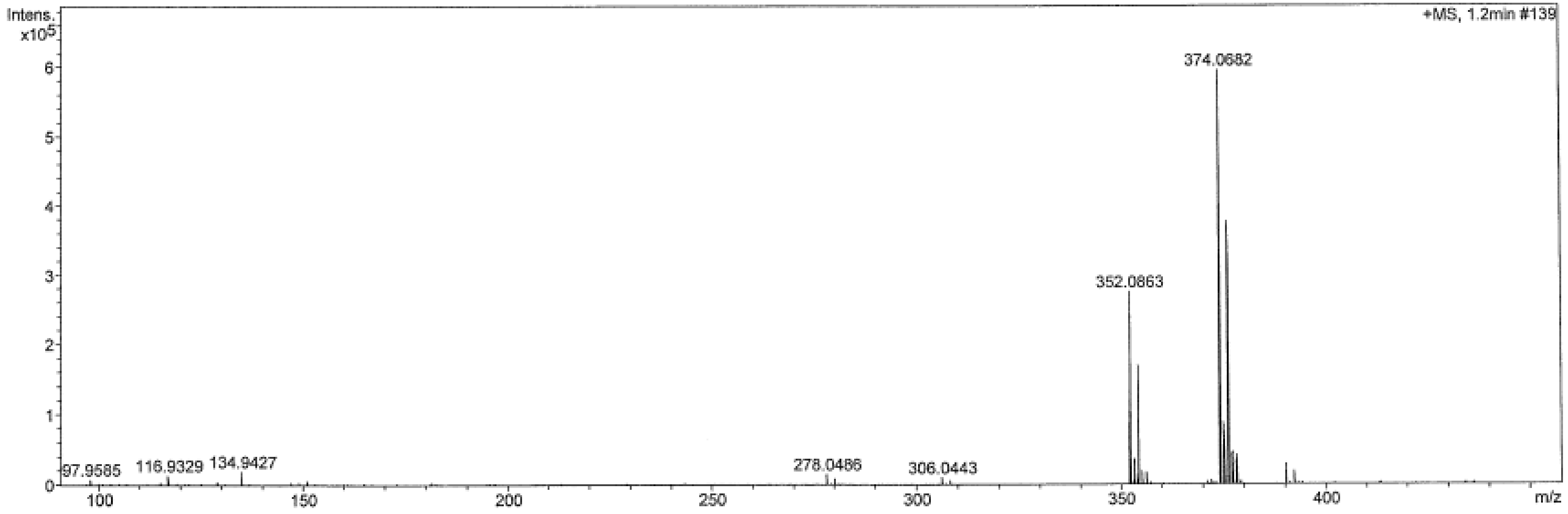

Meas. m/z	#	Formula	m/z	err [ppm]	err [mDa]	mSigma	Mean err [ppm]
352.0863	1	C 18 H 20 Cl 2 N O 2	352.0866	0.9	0.3	46.5	0.2
374.0682	1	C 18 H 19 Cl 2 N Na O 2	374.0685	0.8	0.3	36.2	0.8

PerkinElmer Spectrum Version 10.03.07
01 July 2015 16:59

Analyst Administrator
Date 01 July 2015 16:59

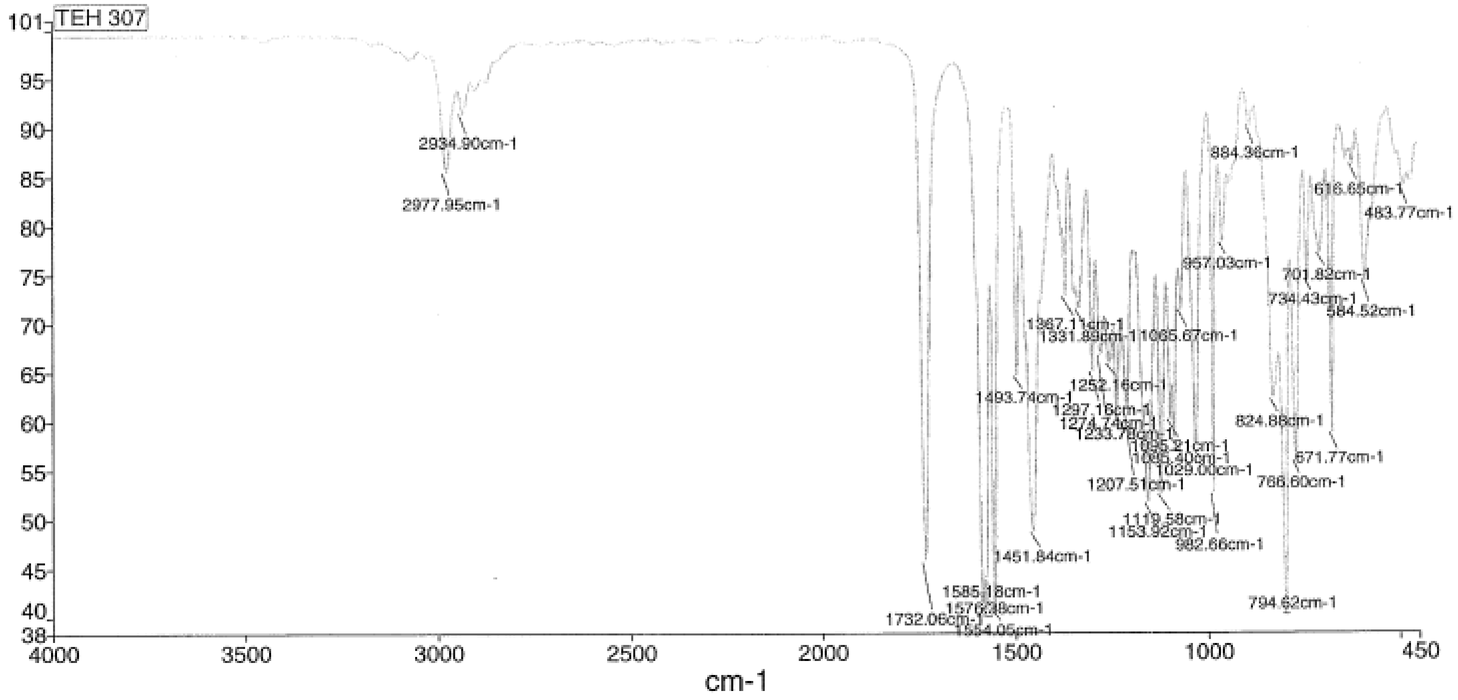

RJKT_01 07 2015_ 36 RJKT_01 07 2015_036

Ethyl 2-(2-[ethyl(3,5-dimethylphenyl)amino]phenyl)acetate 14e

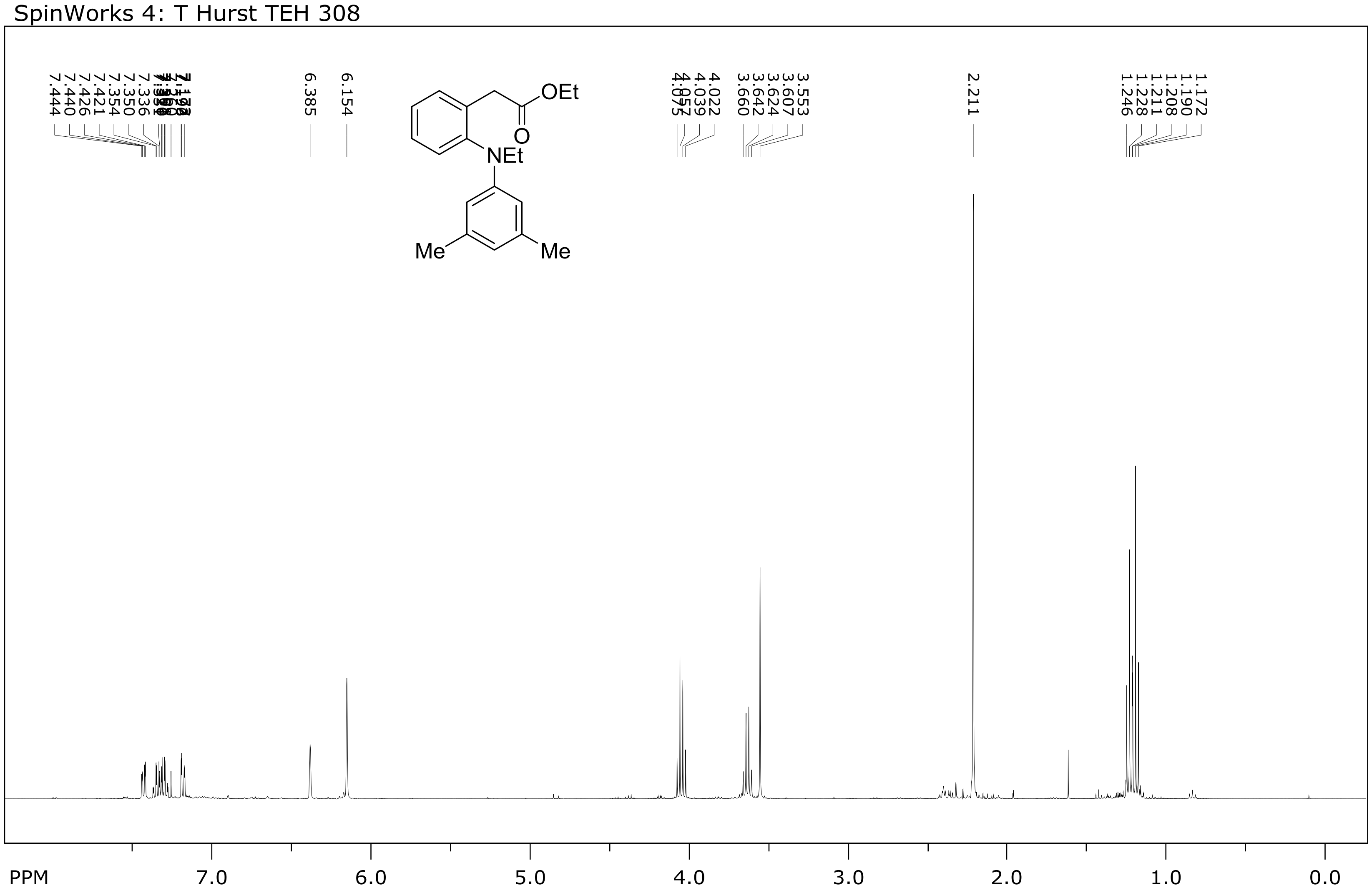

file: ...TEH 308\p5562teh_Proton_ft-1-1.jdf expt: undefined
transmitter freq.: 399.782198 MHz
time domain size: 32768 points
width: 7503.00 Hz = 18.7677 ppm = 0.228973 Hz/pt
number of scans: 8

freq. of 0 ppm: 399.780194 MHz
processed size: 16384 complex points
LB: 0.000 GF: 0.0000

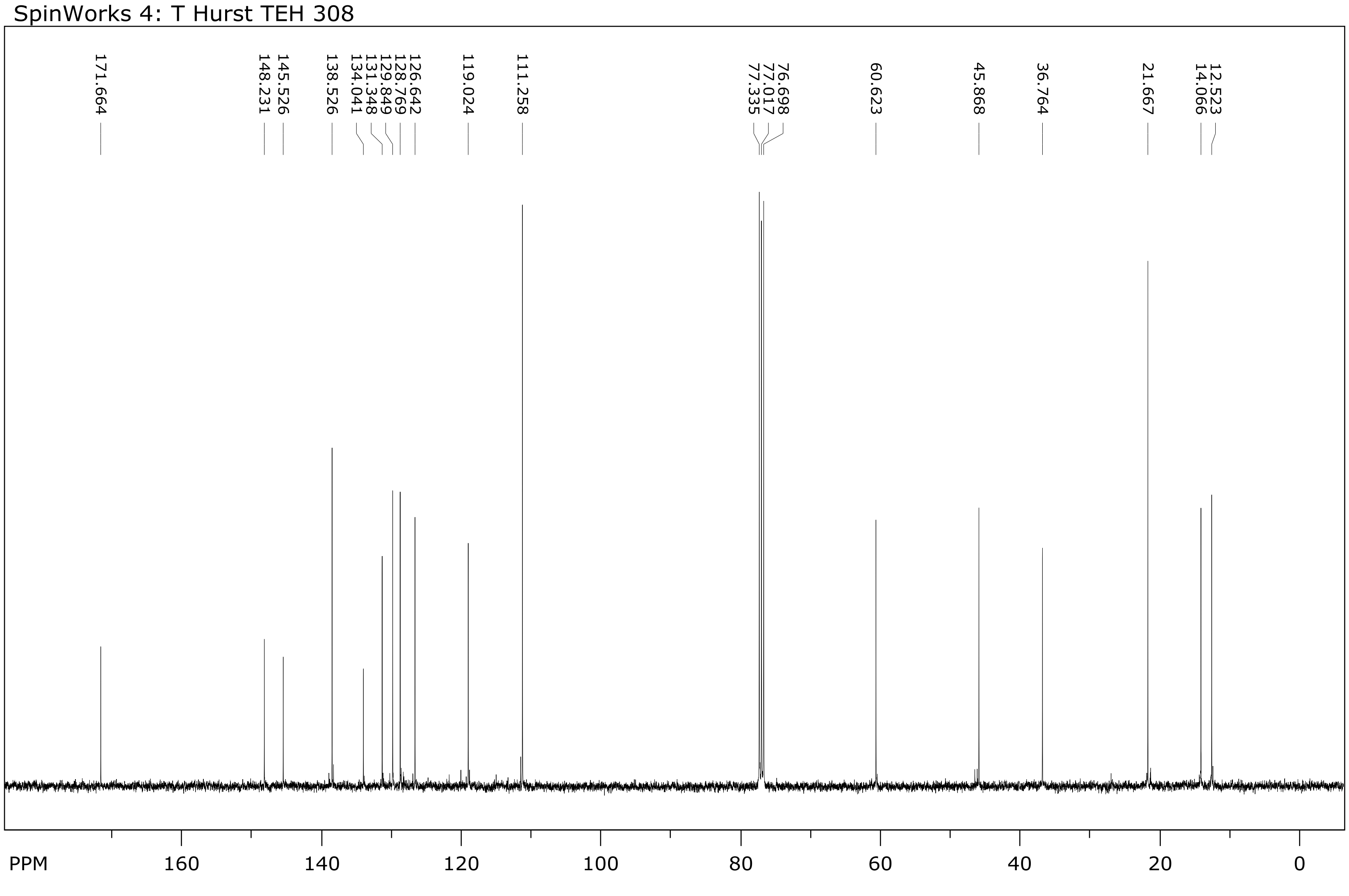

file: ...TEH 308\p5562teh_Carbon_ft-1-1.jdf expt: undefined
transmitter freq.: 100.525303 MHz
time domain size: 65536 points
width: 31407.04 Hz = 312.4292 ppm = 0.479233 Hz/pt
number of scans: 256

freq. of 0 ppm: 100.515264 MHz
processed size: 32768 complex points
LB: 0.000 GF: 0.0000

teh308

York - Chemistry - Mass Spectrometry Service Report

Analysis Information

Acquisition Date 09/07/2015 16:54:50

Analysis Filename rjkt53496th_P1-D-1_01_59716.d
Method 400p_meoh1260_2c1s.m
Submission Name rjkt53496th
Instrument micrOTOF
ESI Positive

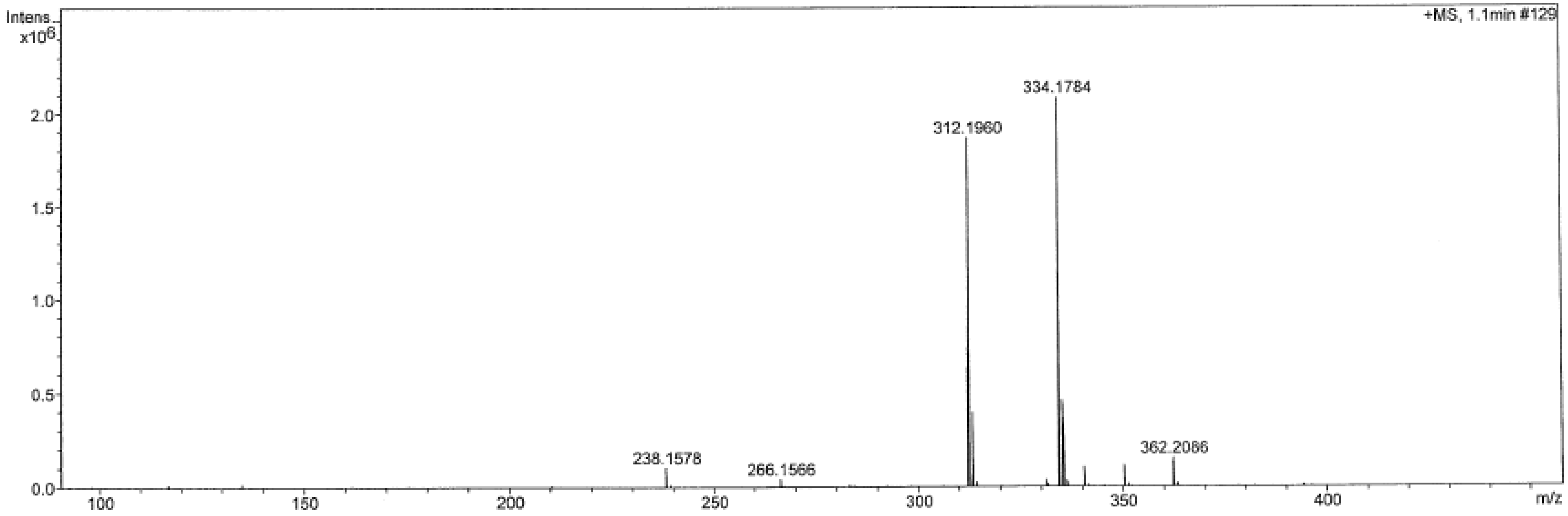

Meas. m/z	#	Formula	m/z	err [ppm]	err [mDa]	mSigma	Mean err [ppm]
312.1960	1	C 20 H 26 N O 2	312.1958	-0.6	-0.2	8.7	-0.6
334.1784	1	C 20 H 25 N Na O 2	334.1778	-2.0	-0.7	6.8	-2.3

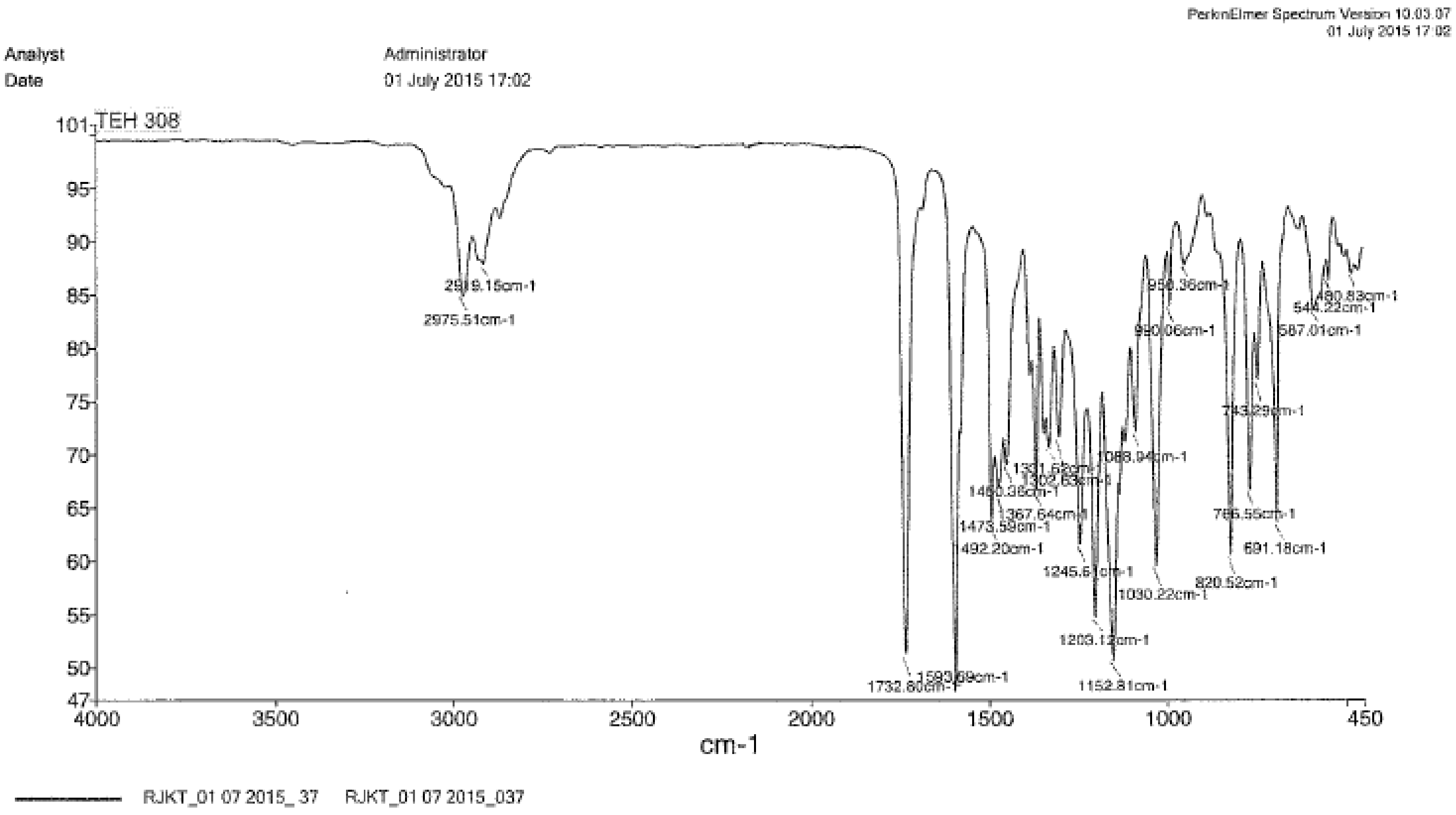
PerkinElmer Spectrum Version 10.03.07
01 July 2015 17:02
Analyst
Administrator
Date
01 July 2015 17:02
TEH 308
2975.51cm-1
1732.80cm-1
1593.69cm-1
1473.59cm-1
1367.64cm-1
1331.62cm-1
1302.63cm-1
1245.61cm-1
1203.12cm-1
1152.81cm-1
1088.94cm-1
1030.22cm-1
990.06cm-1
820.52cm-1
743.29cm-1
691.18cm-1
587.01cm-1
cm-1
RJKT_01 07 2015_ 37 RJKT_01 07 2015_037

Ethyl 2-(4-chloro-2-[ethyl(4-methoxyphenyl)amino]phenyl)acetate 14f

SpinWorks 4: T Hurst TEH 336

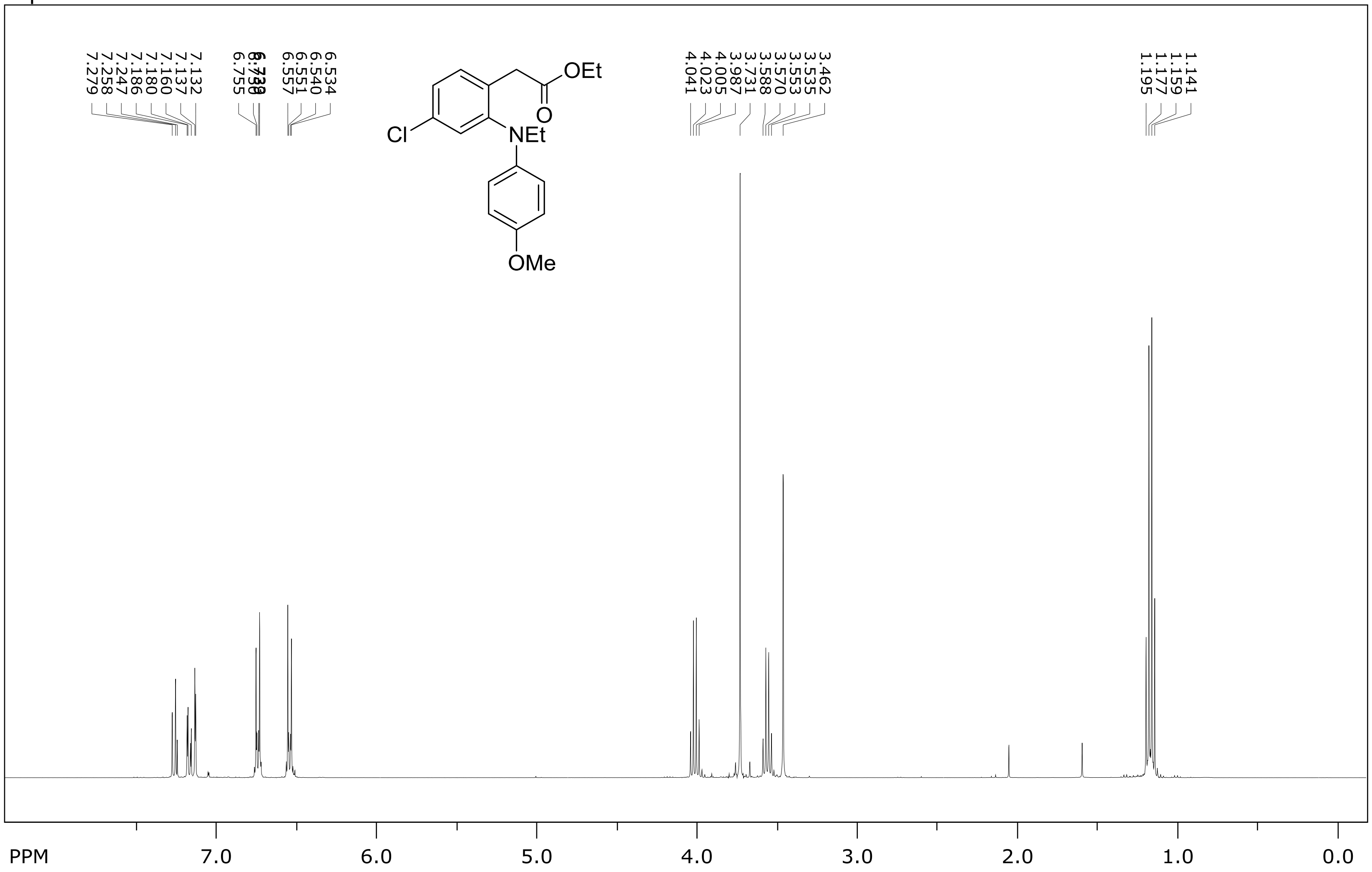

file: ...TEH 336\p6323teh_Proton_ft-1-1.jdf expt: undefined
transmitter freq.: 399.782198 MHz
time domain size: 32768 points
width: 7503.00 Hz = 18.7677 ppm = 0.228973 Hz/pt
number of scans: 8

freq. of 0 ppm: 399.780199 MHz
processed size: 16384 complex points
LB: 0.000 GF: 0.0000

SpinWorks 4: T Hurst TEH 336

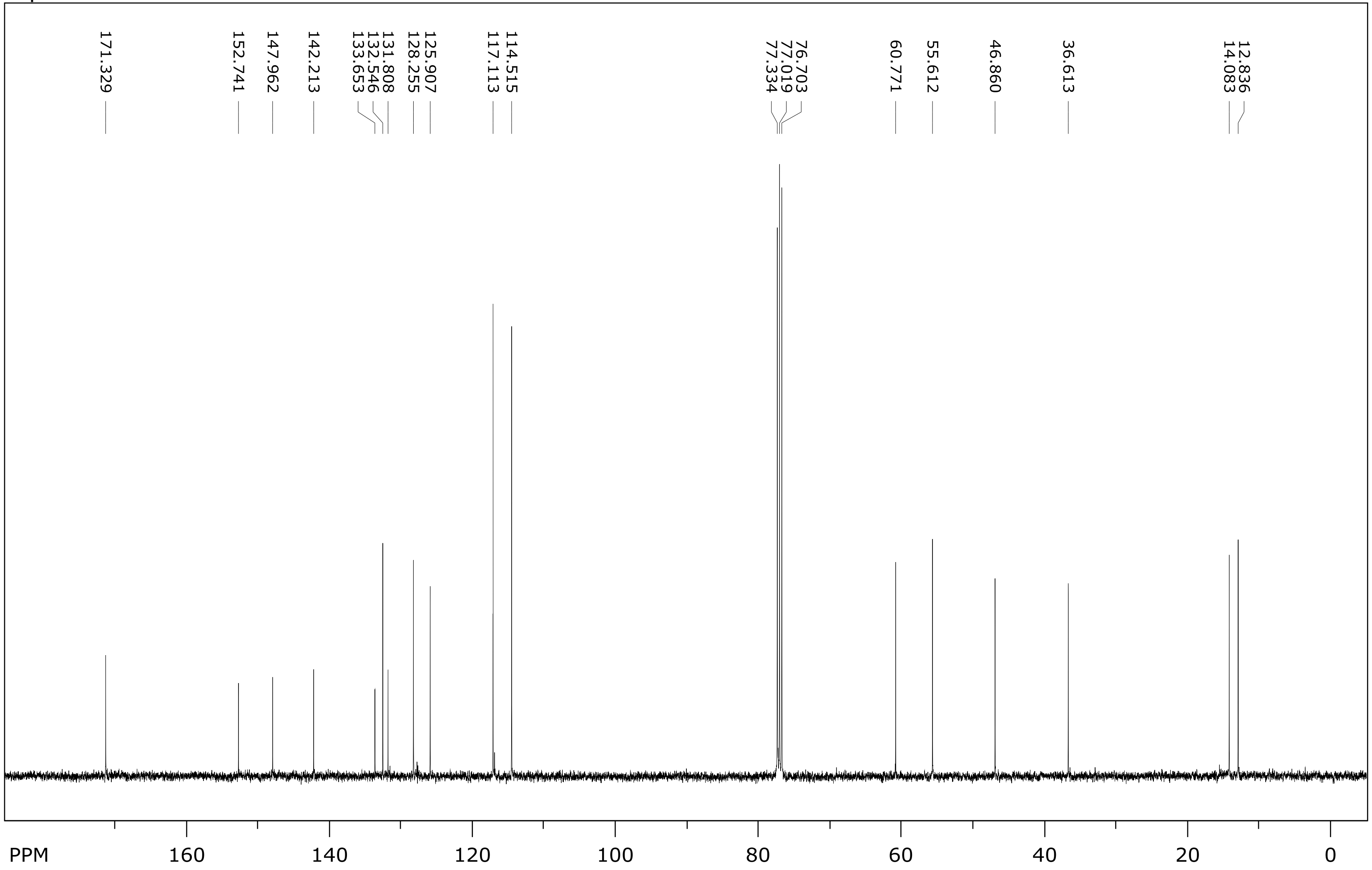

file: ...TEH 336\p6323teh_Carbon_ft-1-1.jdf expt: undefined
transmitter freq.: 100.525303 MHz
time domain size: 65536 points
width: 31407.04 Hz = 312.4292 ppm = 0.479233 Hz/pt
number of scans: 256

freq. of 0 ppm: 100.515261 MHz
processed size: 32768 complex points
LB: 0.000 GF: 0.0000

teh336

York - Chemistry - Mass Spectrometry Service Report

Analysis Information

Acquisition Date 09/07/2015 16:57:47

Analysis Filename	rjkt53497th_P1-D-2_01_59717.d
Method	400p_meoh1260_2c1s.m
Submission Name	rjkt53497th
Instrument	micrOTOF
ESI	Positive

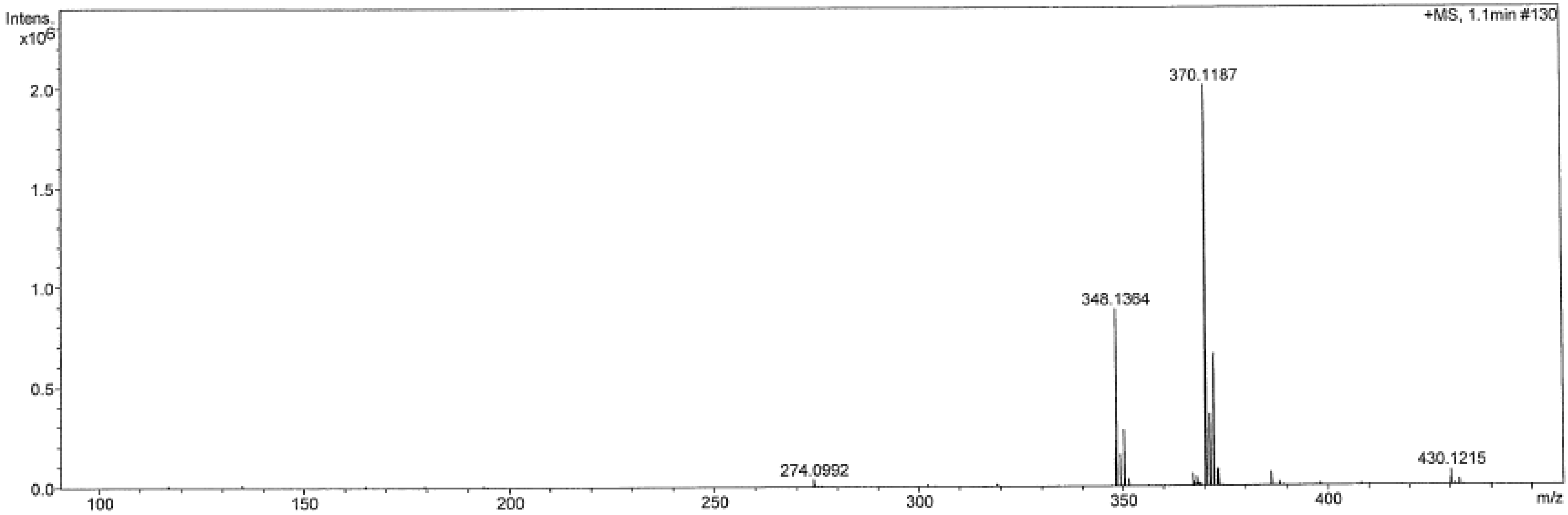

Meas. m/z	#	Formula	m/z	err [ppm]	err [mDa]	mSigma	Mean err [ppm]
348.1364	1	C 19 H 23 Cl N O 3	348.1361	-1.0	-0.3	24.2	-1.4
370.1187	1	C 19 H 22 Cl N Na O 3	370.1180	-1.8	-0.7	19.0	-0.9

PerkinElmer Spectrum Version 10.03.07
01 July 2015 17:04

Analyst Administrator
Date 01 July 2015 17:04

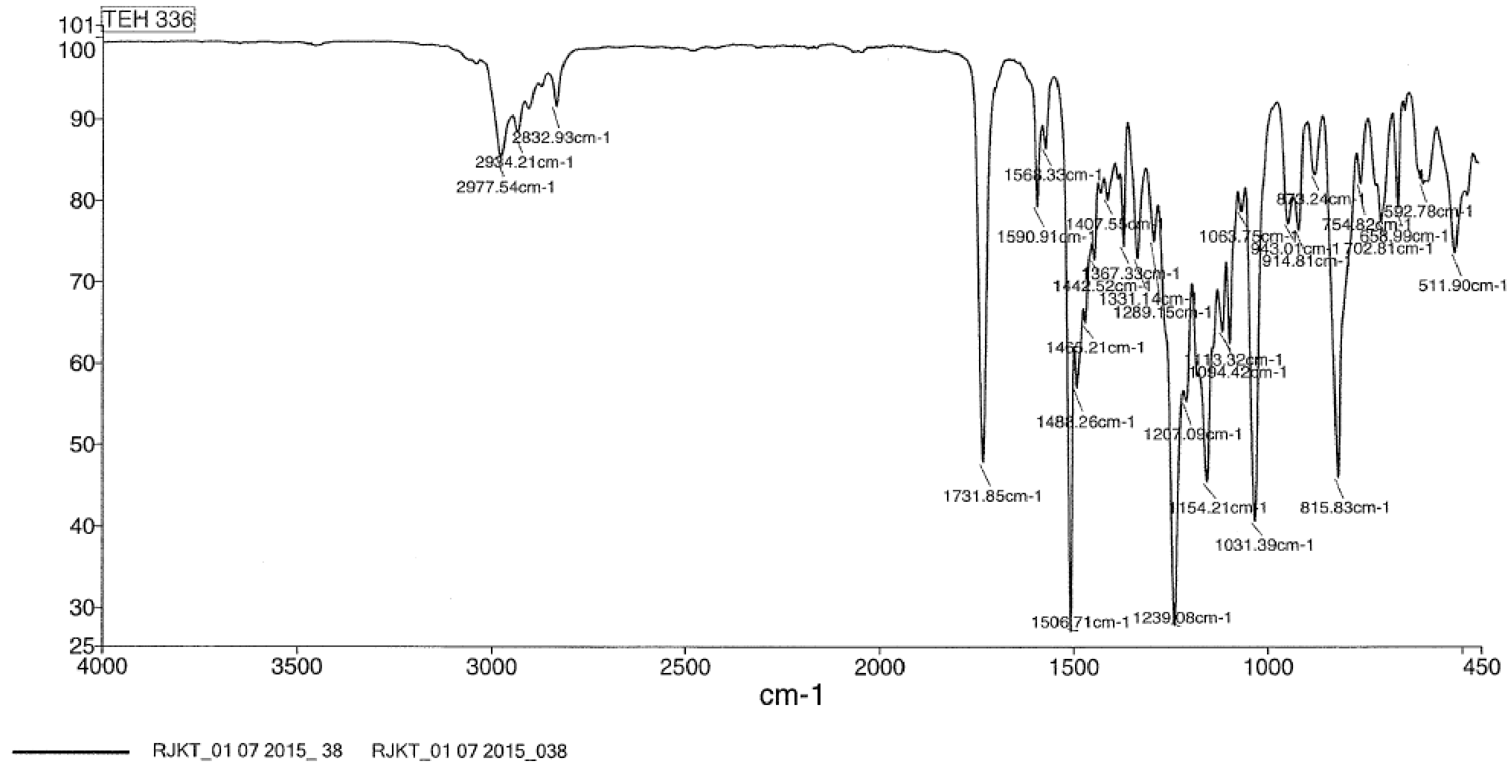

Ethyl 2-(2-phenoxyphenyl)acetate S1

SpinWorks 4: T Hurst TEH 322-891-1

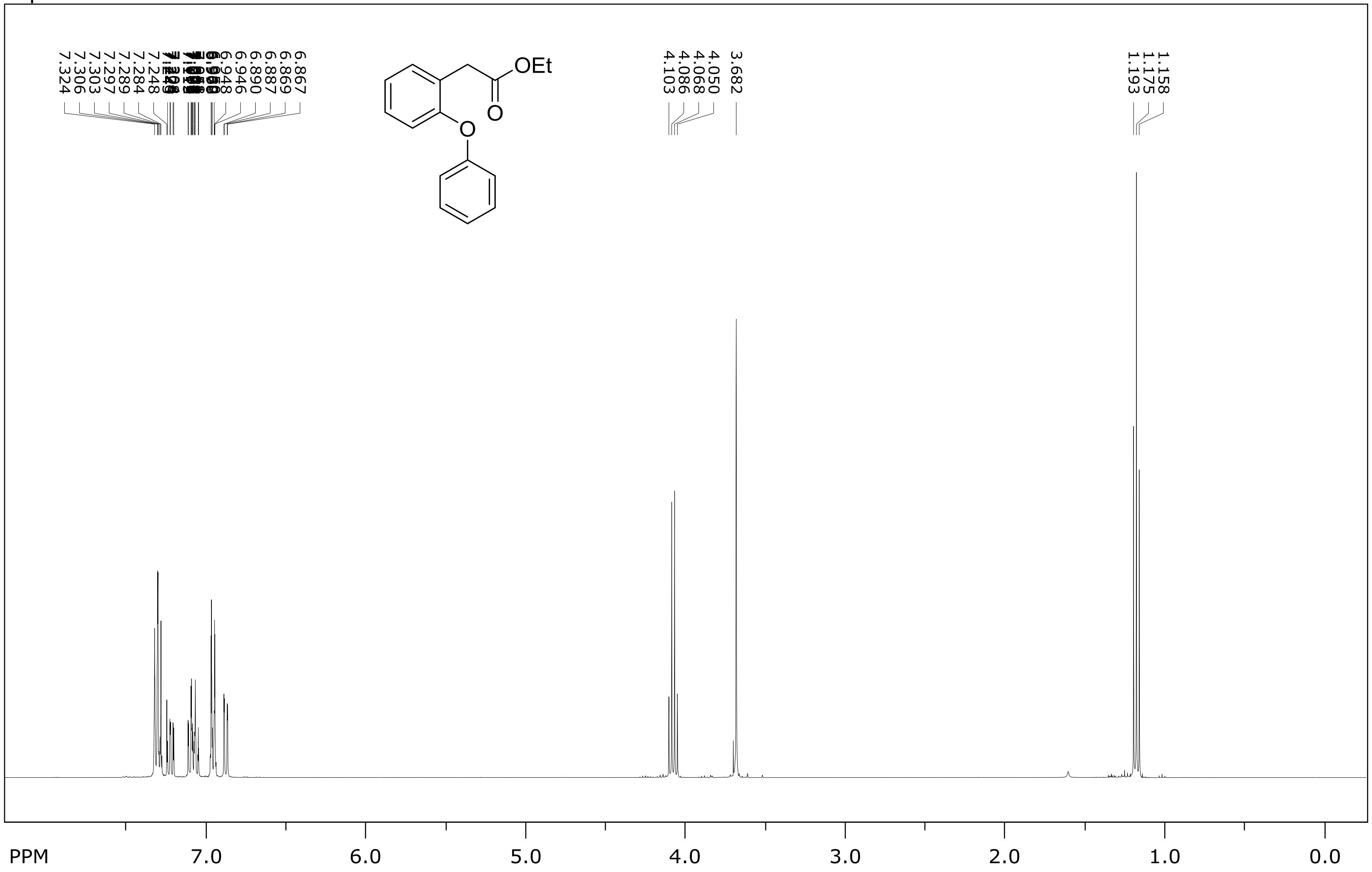

file: ...2-891-1\n9337teh_Proton_ft-1-1.jdf expt: undefined
transmitter freq.: 399.782198 MHz
time domain size: 32768 points
width: 7503.00 Hz = 18.7677 ppm = 0.228973 Hz/pt
number of scans: 8

freq. of 0 ppm: 399.780199 MHz
processed size: 16384 complex points
LB: 0.000 GF: 0.0000

SpinWorks 4: T Hurst TEH 322-891-1

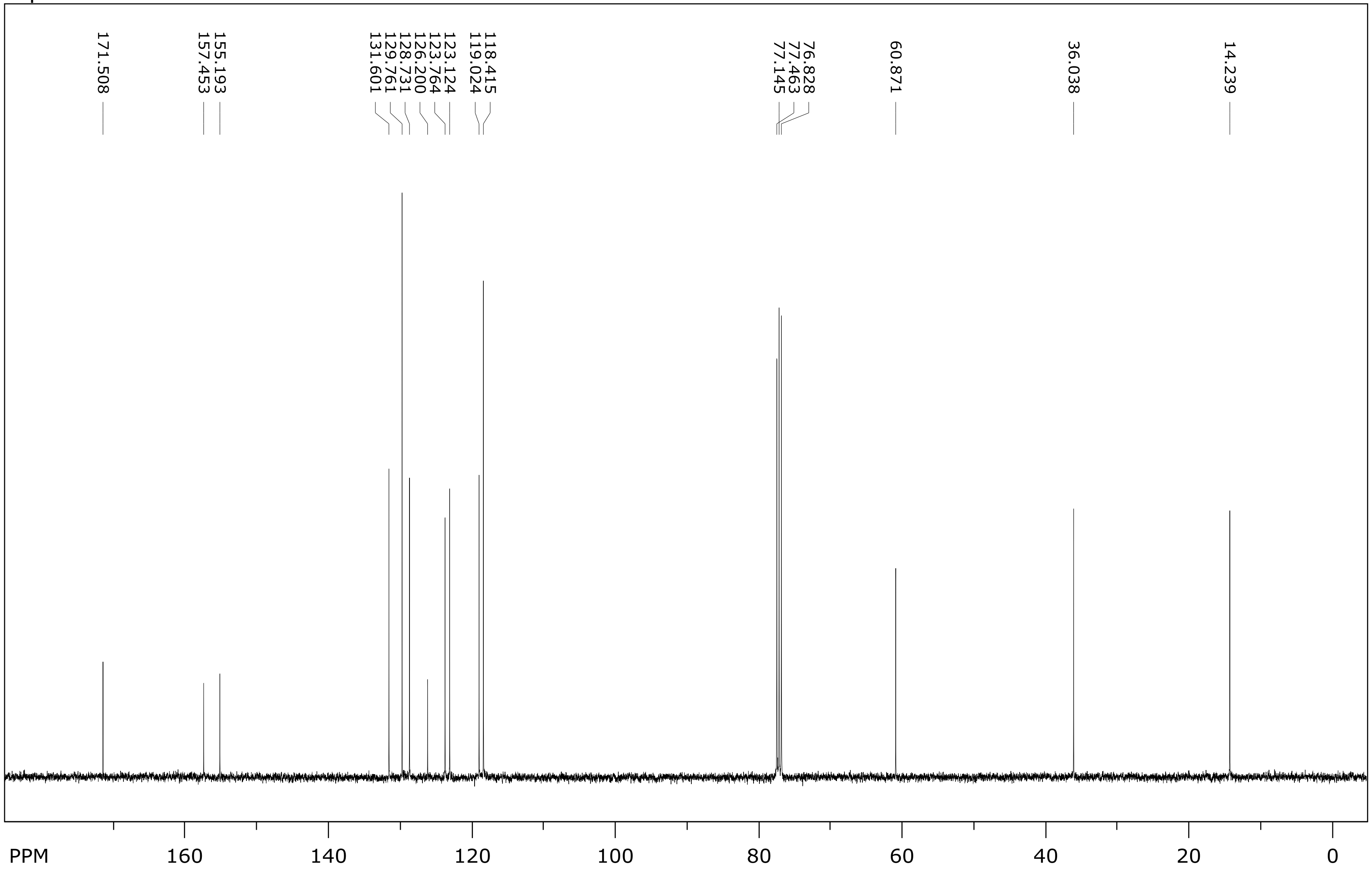

file: ...2-891-1\n9337teh_Carbon_ft-1-1.jdf expt: undefined
transmitter freq.: 100.525303 MHz
time domain size: 65536 points
width: 31407.04 Hz = 312.4292 ppm = 0.479233 Hz/pt
number of scans: 256

freq. of 0 ppm: 100.515251 MHz
processed size: 32768 complex points
LB: 0.000 GF: 0.0000

2-[2-(Phenylsulfanyl)phenyl]acetic acid S2

SpinWorks 4: T Hurst EXP 915

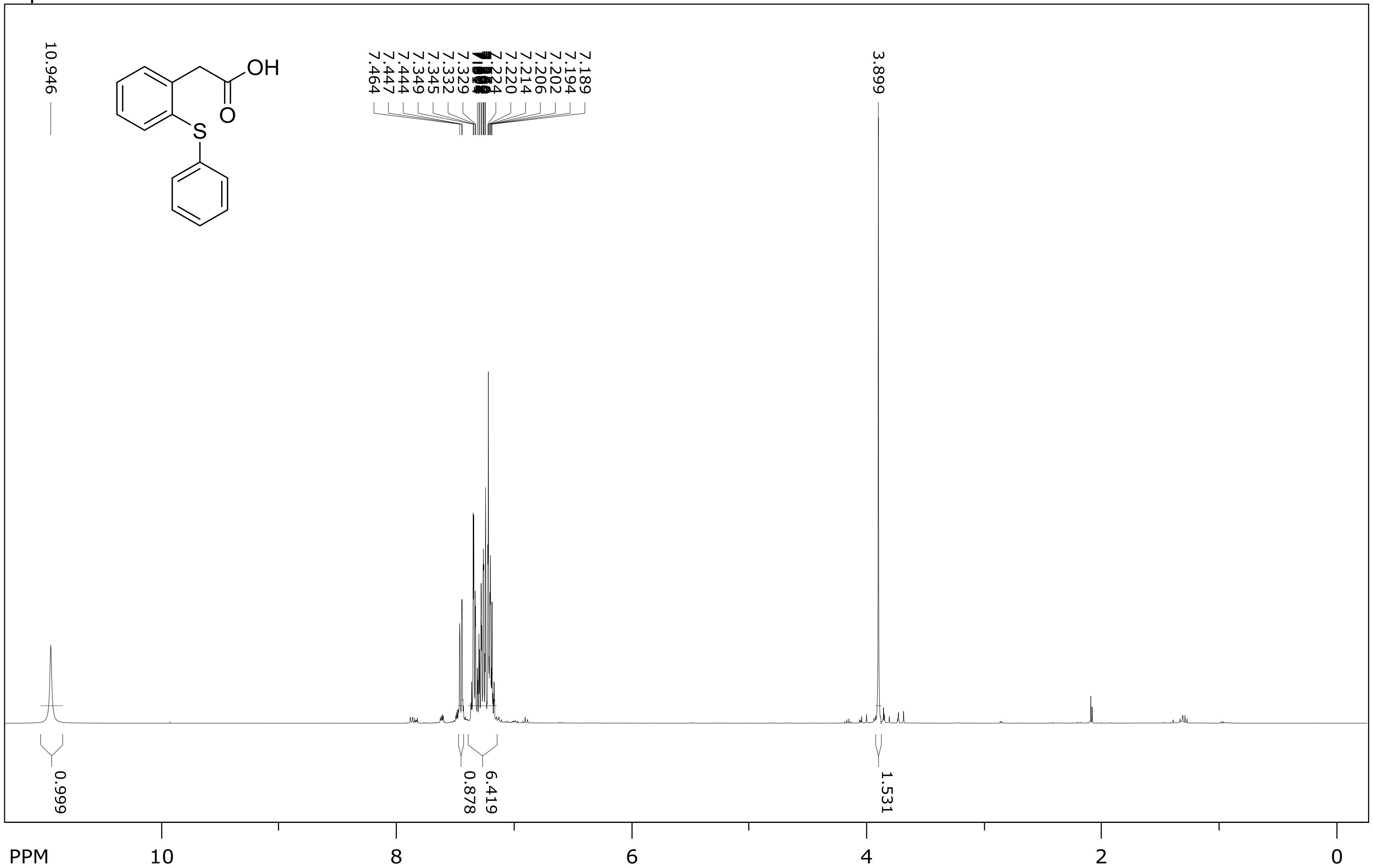

file: ...1-915-1\n9902teh_Proton_ft-1-1.jdf expt: undefined
transmitter freq.: 399.782198 MHz
time domain size: 32768 points
width: 7503.00 Hz = 18.7677 ppm = 0.228973 Hz/pt
number of scans: 8

freq. of 0 ppm: 399.780199 MHz
processed size: 16384 complex points
LB: 0.000 GF: 0.0000

SpinWorks 4: T Hurst EXP 915

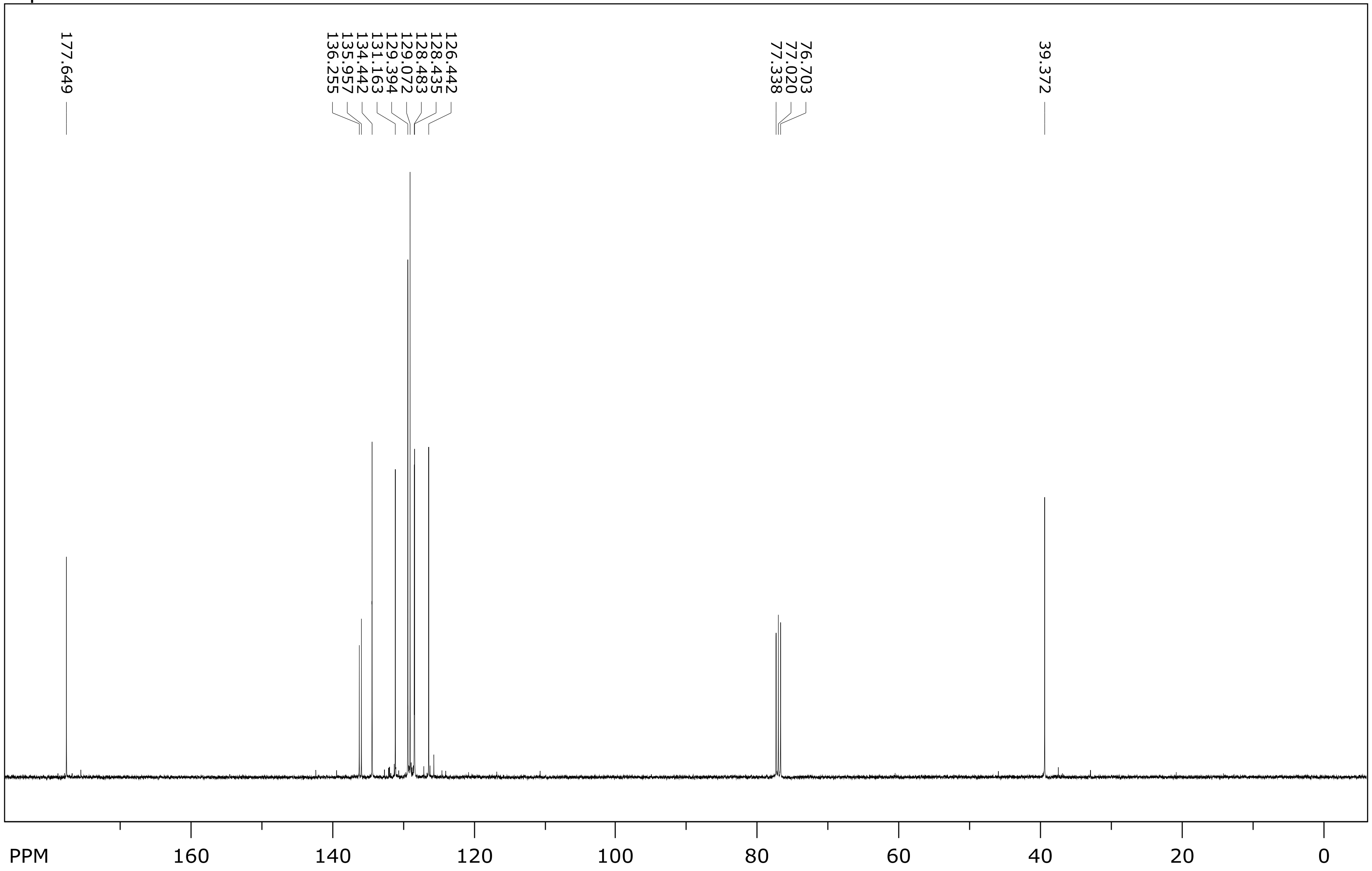

file: ...1-915-1\n9902teh_Carbon_ft-1-1.jdf   expt: undefined
transmitter freq.: 100.525303 MHz
time domain size: 65536 points
width: 31407.04 Hz = 312.4292 ppm = 0.479233 Hz/pt
number of scans: 256

freq. of 0 ppm: 100.515273 MHz
processed size: 32768 complex points
LB: 0.000   GF: 0.0000

Ethyl 2-[2-(phenylsulfanyl)phenyl]acetate S3

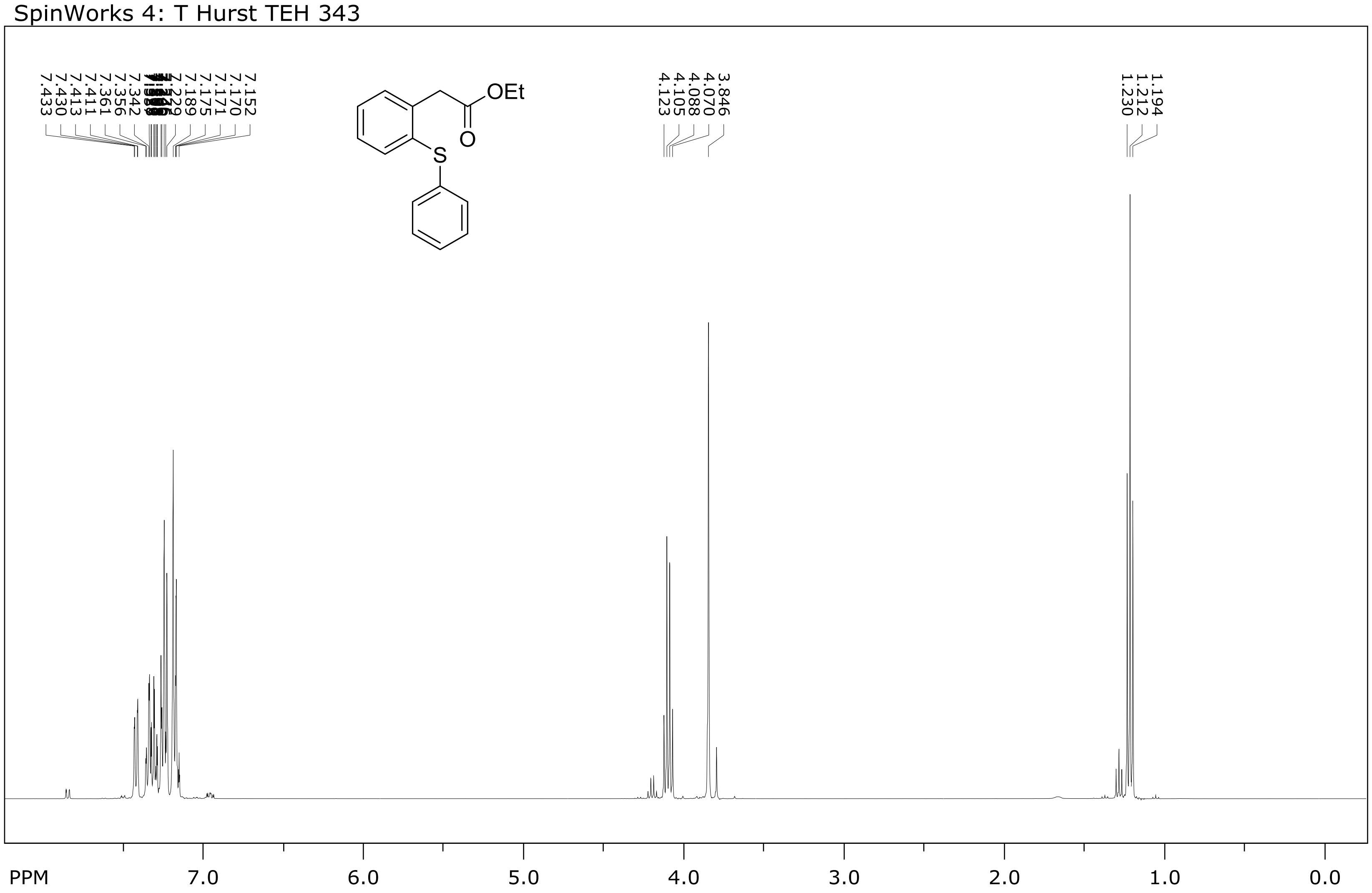

file: ...TEH 343\p6324teh_Proton_ft-1-1.jdf expt: undefined
transmitter freq.: 399.782198 MHz
time domain size: 32768 points
width: 7503.00 Hz = 18.7677 ppm = 0.228973 Hz/pt
number of scans: 8

freq. of 0 ppm: 399.780199 MHz
processed size: 16384 complex points
LB: 0.000 GF: 0.0000

SpinWorks 4: T Hurst TEH 343

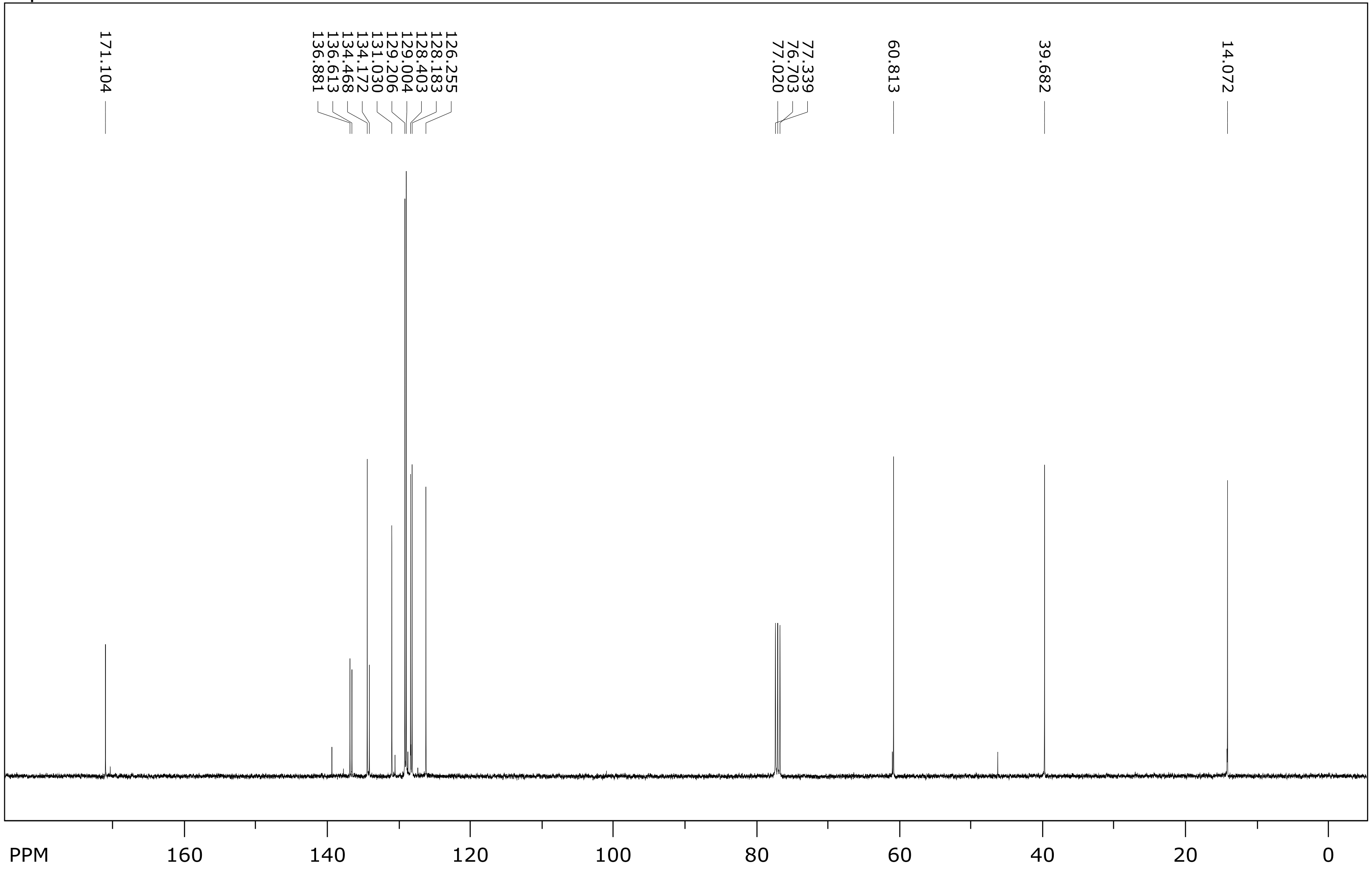

file: ...TEH 343\p6324teh_Carbon_ft-1-1.jdf expt: undefined
transmitter freq.: 100.525303 MHz
time domain size: 65536 points
width: 31407.04 Hz = 312.4292 ppm = 0.479233 Hz/pt
number of scans: 256

freq. of 0 ppm: 100.515269 MHz
processed size: 32768 complex points
LB: 0.000 GF: 0.0000

teh343

York - Chemistry - Mass Spectrometry Service Report

Analysis Information

Acquisition Date 10/08/2015 16:21:30

Analysis Filename	rjkt54043th_P1-A-9_01_60276.d
Method	400p_meoh1260_2c1s.m
Submission Name	rjkt54043th
Instrument	micrOTOF
ESI	Positive

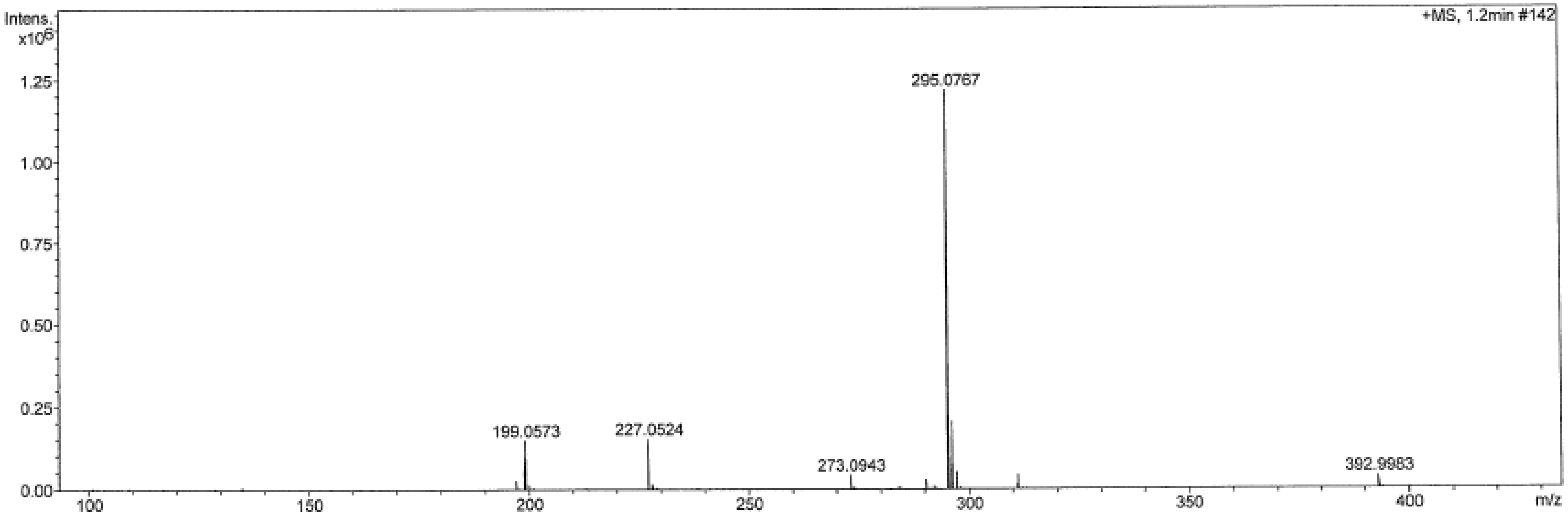

Meas. m/z	#	Formula	m/z	err [ppm]	err [mDa]	mSigma	Mean err [ppm]
273.0943	1	C 16 H 17 O 2 S	273.0944	0.3	0.1	23.1	-1.3
295.0767	1	C 16 H 16 Na O 2 S	295.0763	-1.2	-0.3	12.3	-1.5

PerkinElmer Spectrum Version 10.03.07
10 August 2015 16:33

Analyst Administrator

Date 10 August 2015 16:33

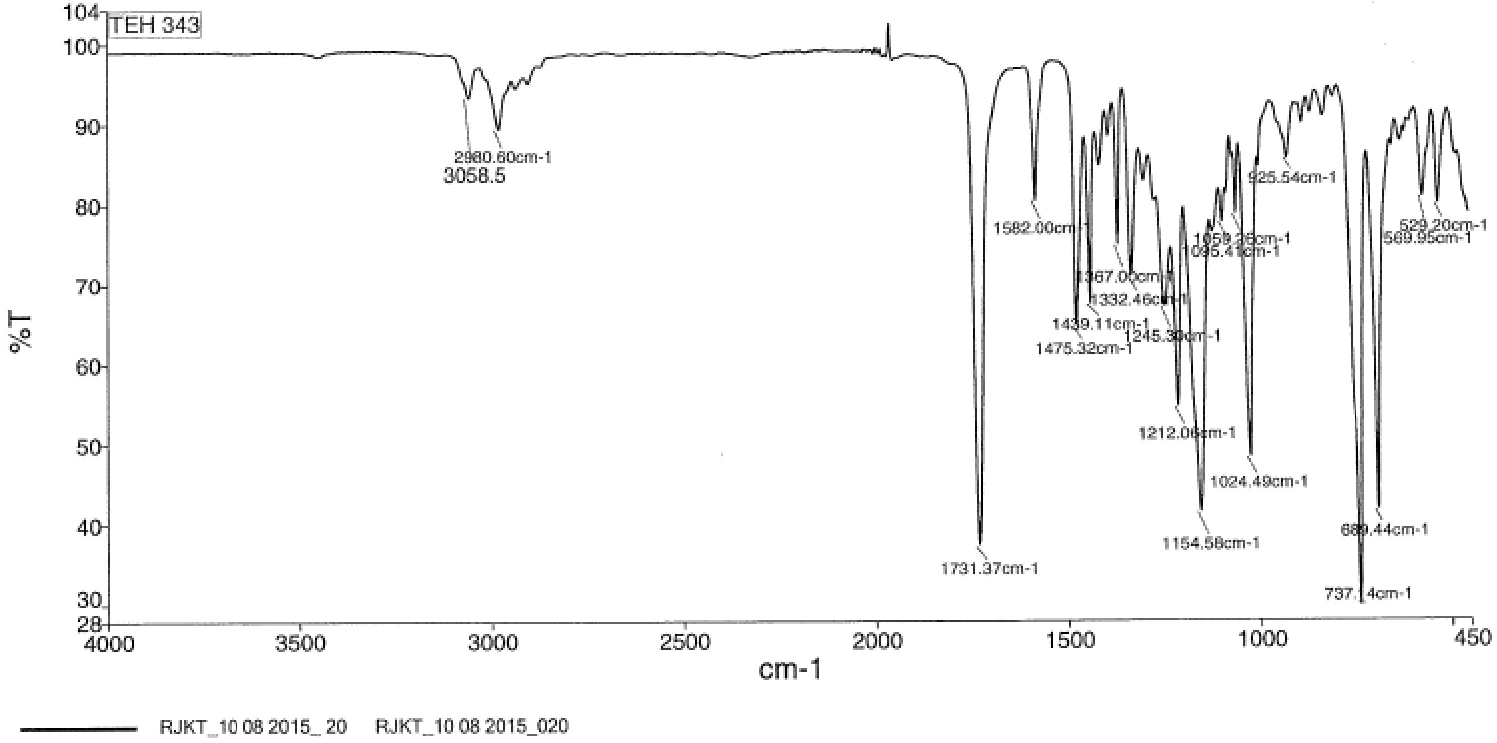

1,2-Diethyl 2-(2-[ethyl(phenyl)amino]phenyl)propanedioate 15a

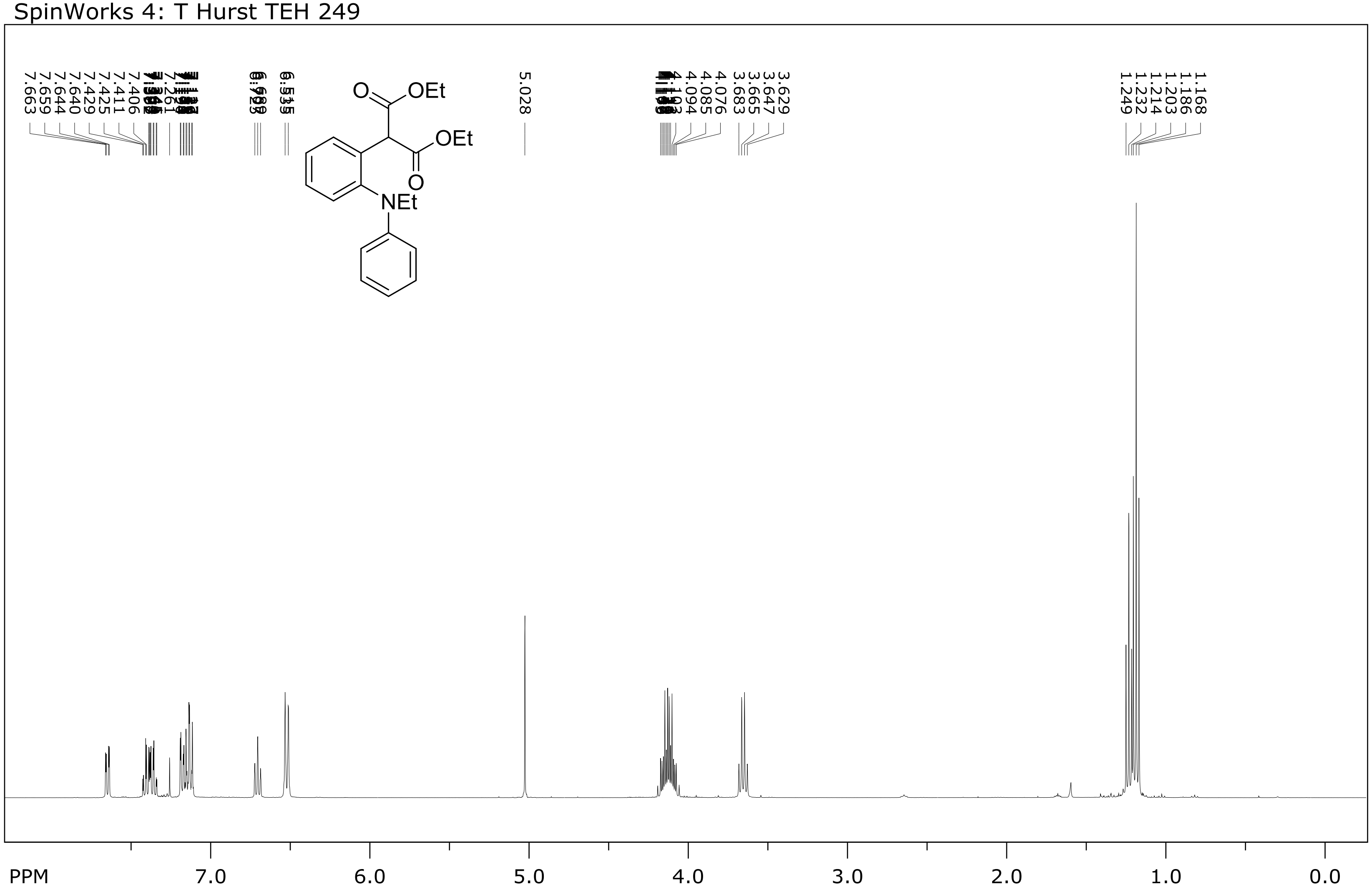

file: ...TEH 249\p6325teh_Proton_ft-1-1.jdf expt: undefined
transmitter freq.: 399.782198 MHz
time domain size: 32768 points
width: 7503.00 Hz = 18.7677 ppm = 0.228973 Hz/pt
number of scans: 8

freq. of 0 ppm: 399.780194 MHz
processed size: 16384 complex points
LB: 0.000 GF: 0.0000

SpinWorks 4: T Hurst TEH 249

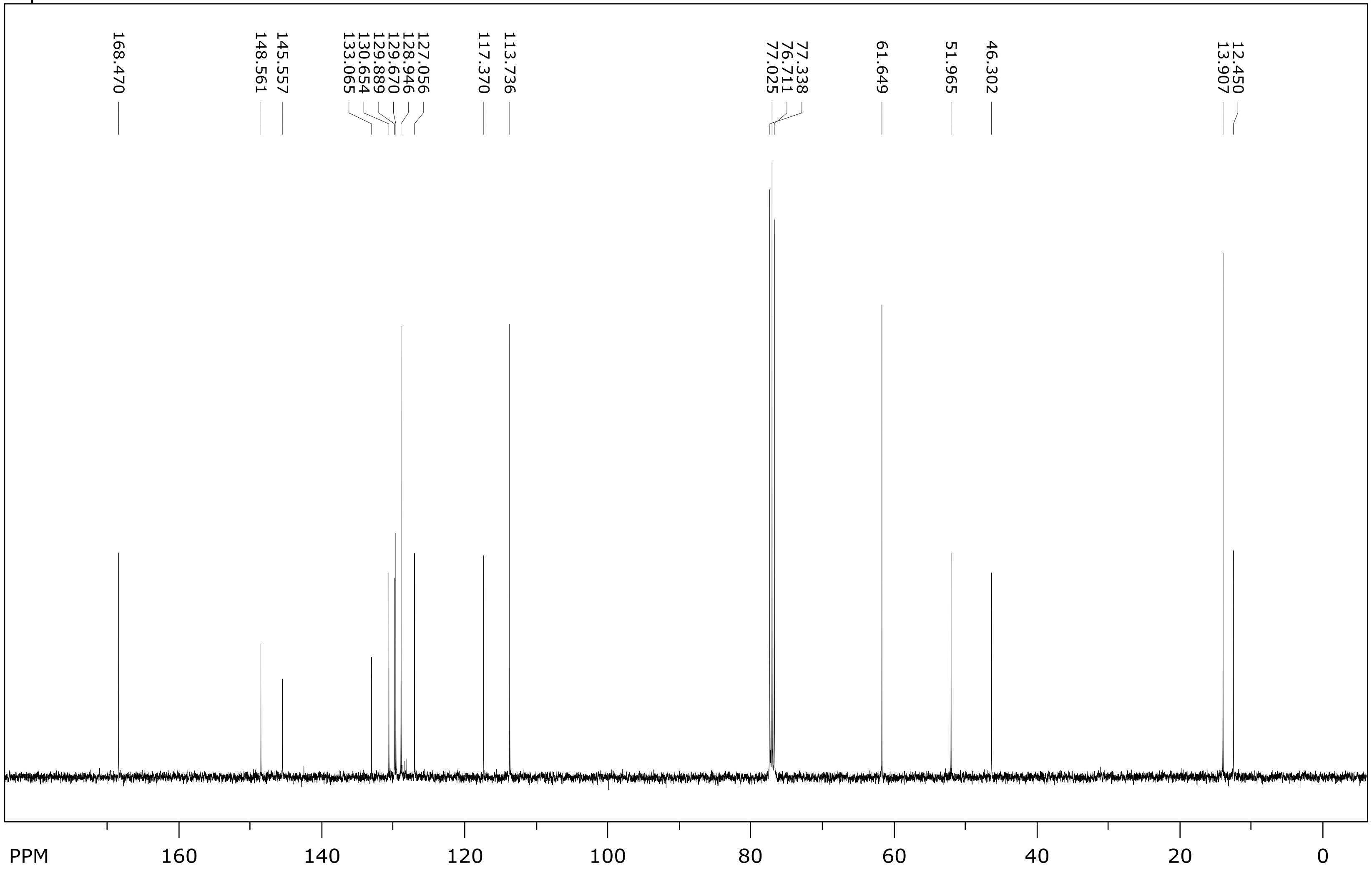

file: ...TEH 249\p6325teh_Carbon_ft-1-1.jdf   expt: undefined
transmitter freq.: 100.525303 MHz
time domain size: 65536 points
width: 31407.04 Hz = 312.4292 ppm = 0.479233 Hz/pt
number of scans: 256

freq. of 0 ppm: 100.515261 MHz
processed size: 32768 complex points
LB: 0.000   GF: 0.0000

teh249

York - Chemistry - Mass Spectrometry Service Report

Analysis Information Acquisition Date 09/07/2015 17:00:41

Analysis Filename	rjkt53498th_P1-D-3_01_59718.d
Method	400p_meoh1260_2c1s.m
Submission Name	rjkt53498th
Instrument	micrOTOF
ESI	Positive

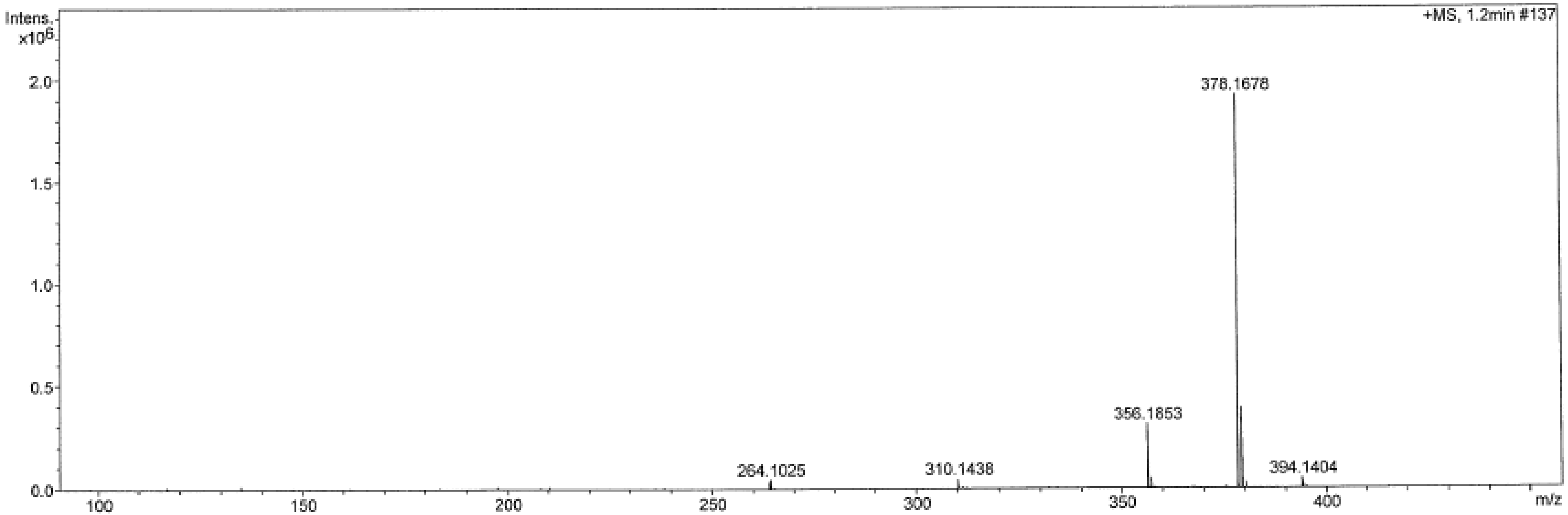

Meas. m/z	#	Formula	m/z	err [ppm]	err [mDa]	mSigma	Mean err [ppm]
356.1853	1	C 21 H 26 N O 4	356.1856	0.9	0.3	43.9	0.8
378.1678	1	C 21 H 25 N Na O 4	378.1676	-0.6	-0.2	18.5	0.6

PerkinElmer Spectrum Version 10.03.07
01 July 2015 17:07

Analyst Administrator
Date 01 July 2015 17:07

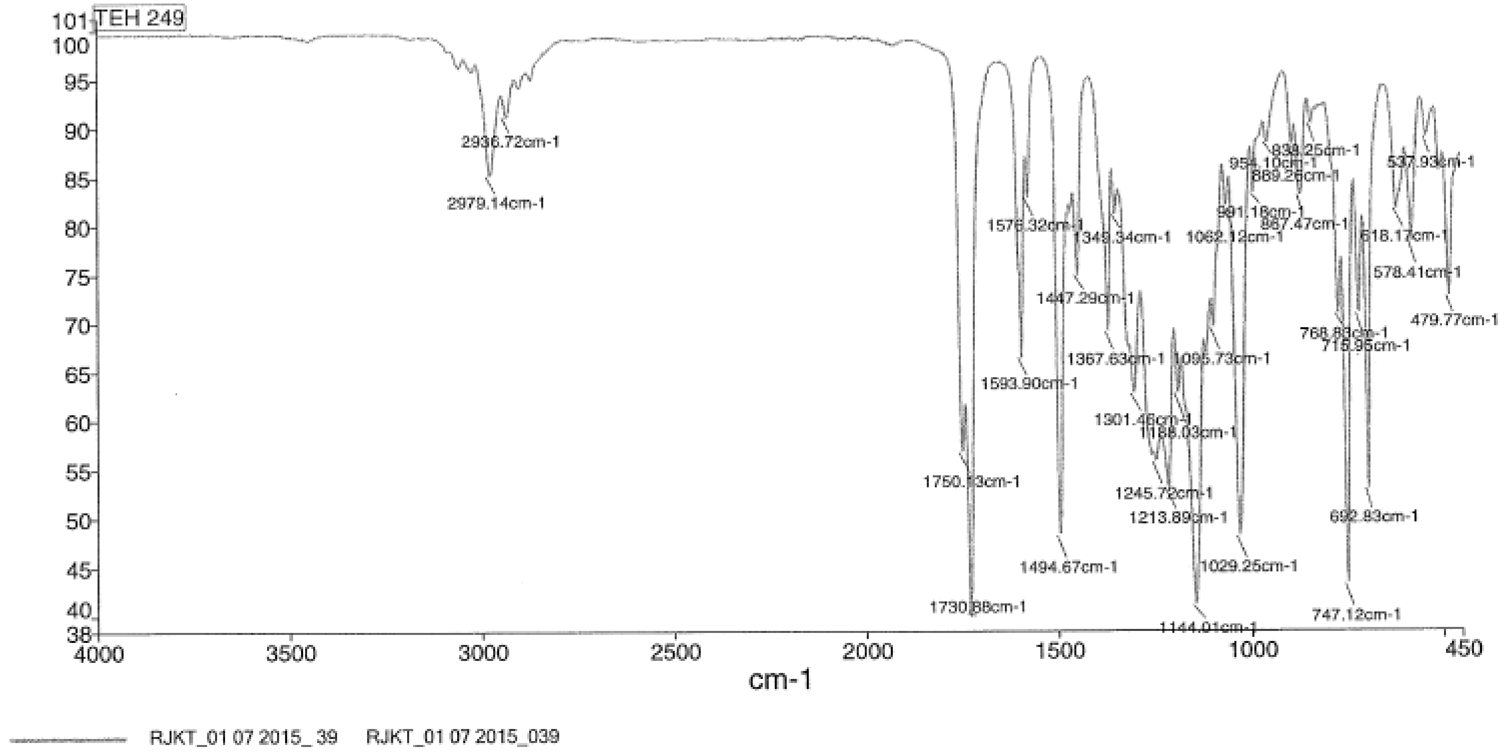

1,2-Diethyl 2-(2-[benzyl(phenyl)amino]phenyl)propanedioate 15b

SpinWorks 4: TEH 288-810-1

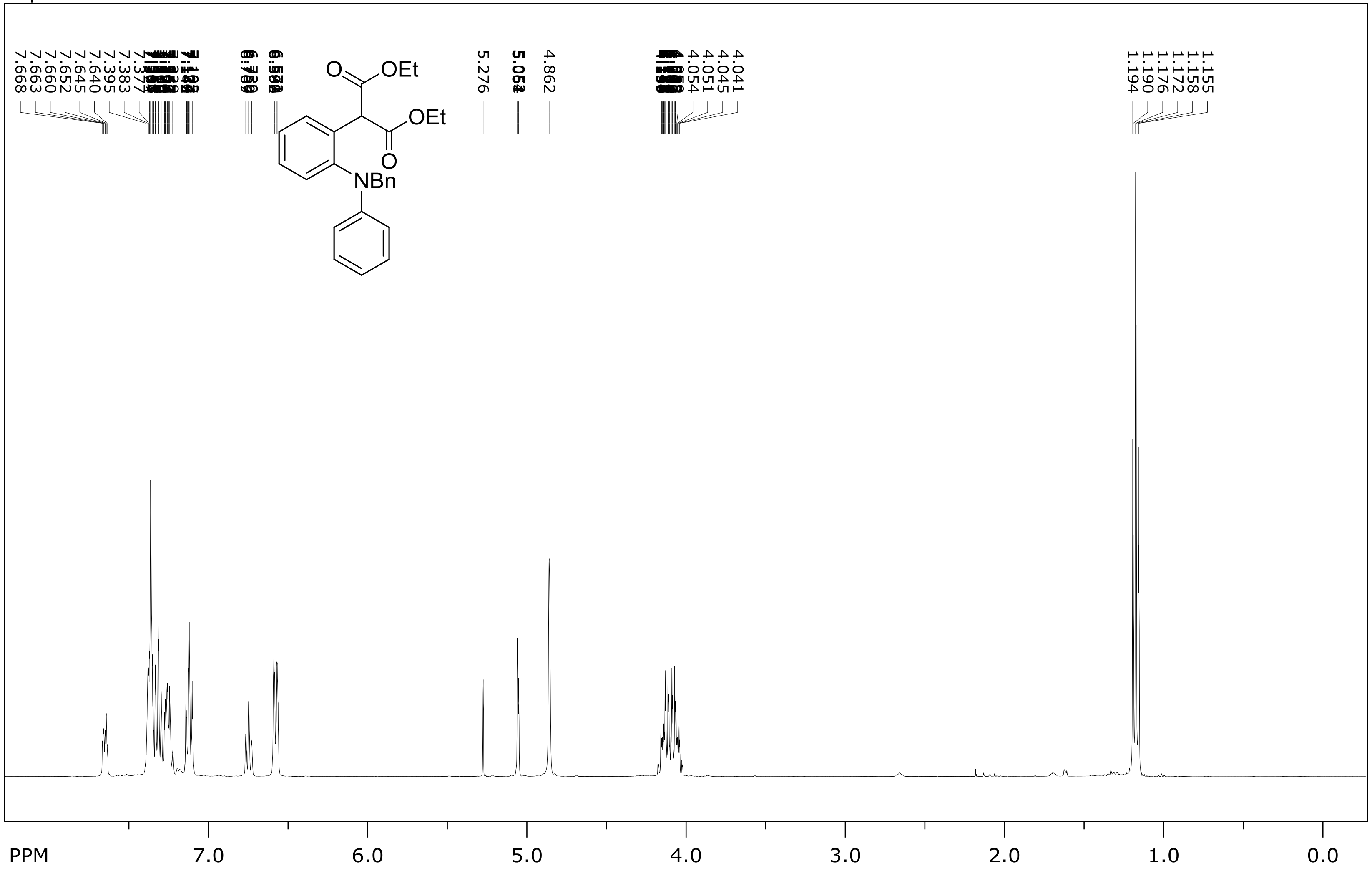

file: ...8-810-1\n4438teh_Proton_ft-1-1.jdf expt: undefined
transmitter freq.: 399.782198 MHz
time domain size: 32768 points
width: 7503.00 Hz = 18.7677 ppm = 0.228973 Hz/pt
number of scans: 8

freq. of 0 ppm: 399.780199 MHz
processed size: 16384 complex points
LB: 0.000 GF: 0.0000

SpinWorks 4: TEH 288-810-1

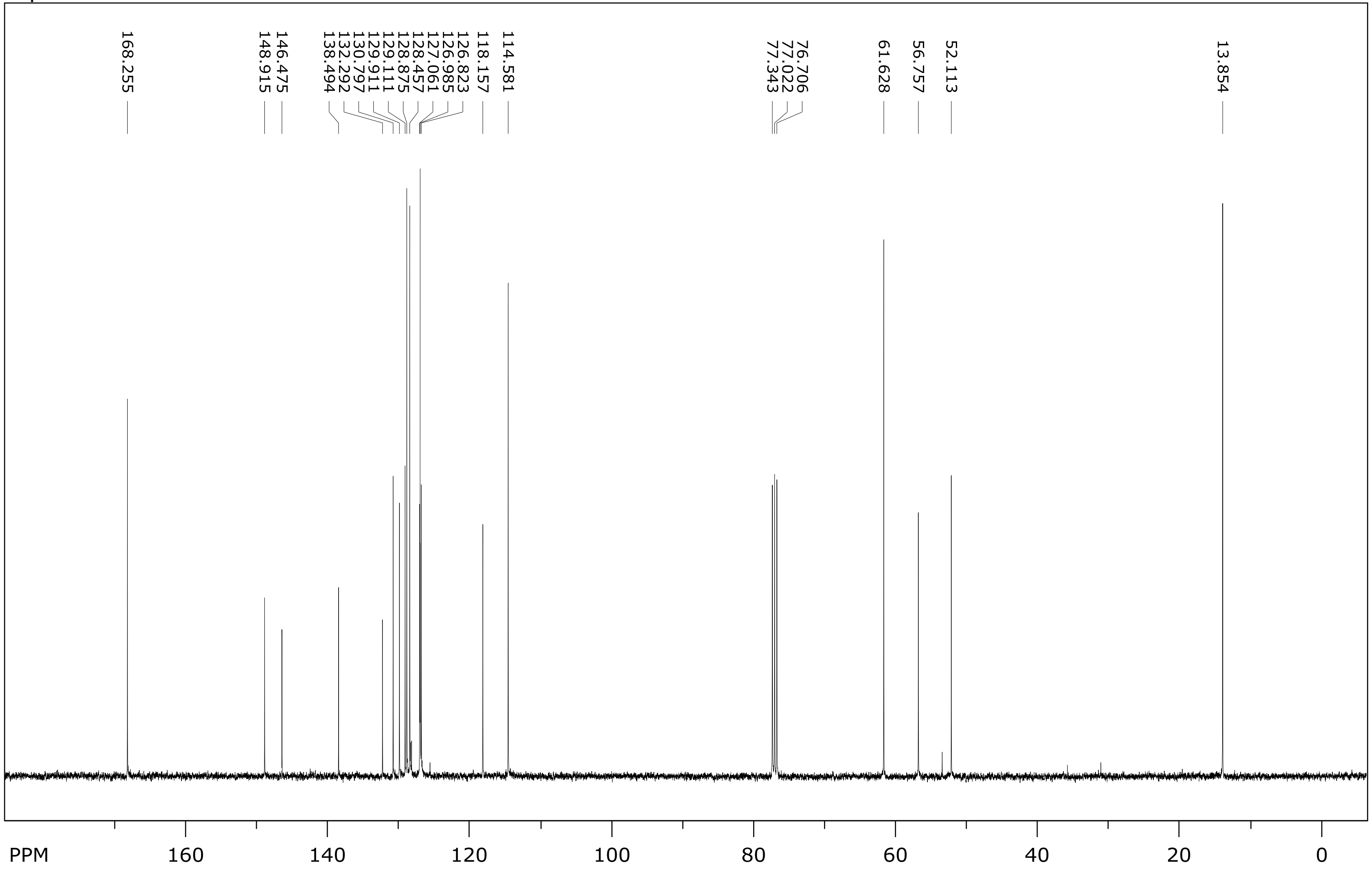

file: ...8-810-1\n4438teh_Carbon_ft-1-1.jdf expt: undefined
transmitter freq.: 100.525303 MHz
time domain size: 65536 points
width: 31407.04 Hz = 312.4292 ppm = 0.479233 Hz/pt
number of scans: 256

freq. of 0 ppm: 100.515269 MHz
processed size: 32768 complex points
LB: 0.000 GF: 0.0000

teh288

York - Chemistry - Mass Spectrometry Service Report

Analysis Information

Acquisition Date 09/07/2015 17:03:39

Analysis Filename	rjkt53499th_P1-D-4_01_59719.d
Method	600p_meoh1260_2c1s.m
Submission Name	rjkt53499th
Instrument	micrOTOF
ESI	Positive

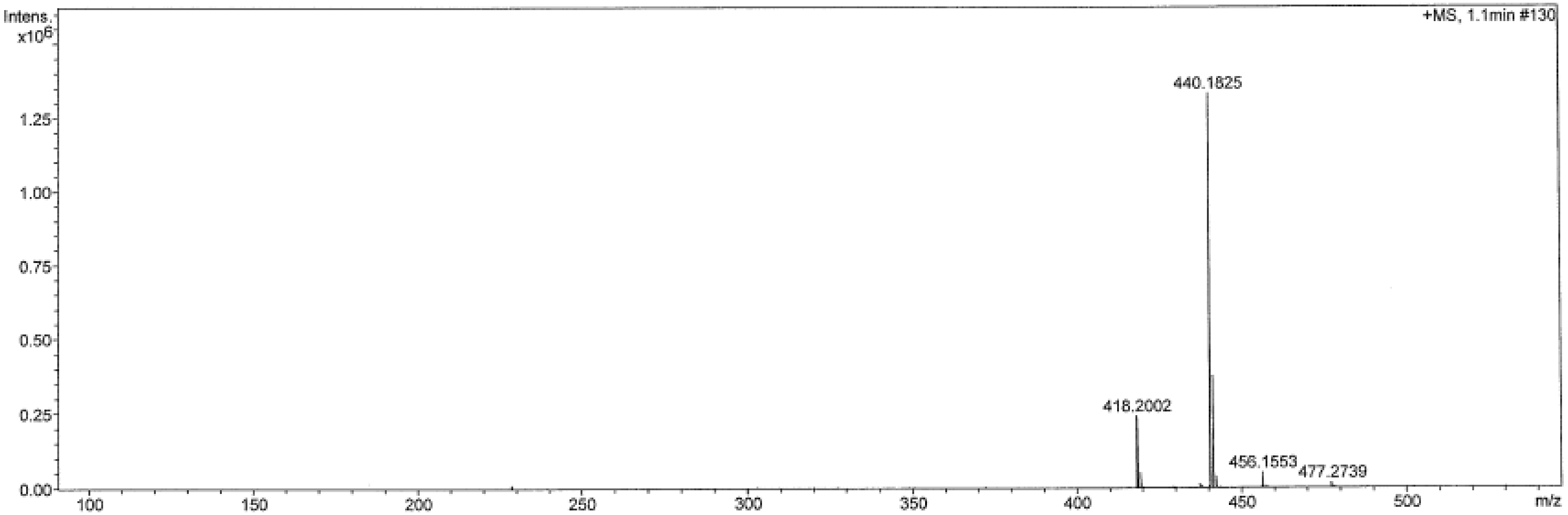

Meas. m/z	#	Formula	m/z	err [ppm]	err [mDa]	mSigma	Mean err [ppm]
418.2002	1	C 26 H 28 N O 4	418.2013	2.7	1.1	46.5	2.5
440.1825	1	C 26 H 27 N Na O 4	440.1832	1.7	0.7	10.8	2.4

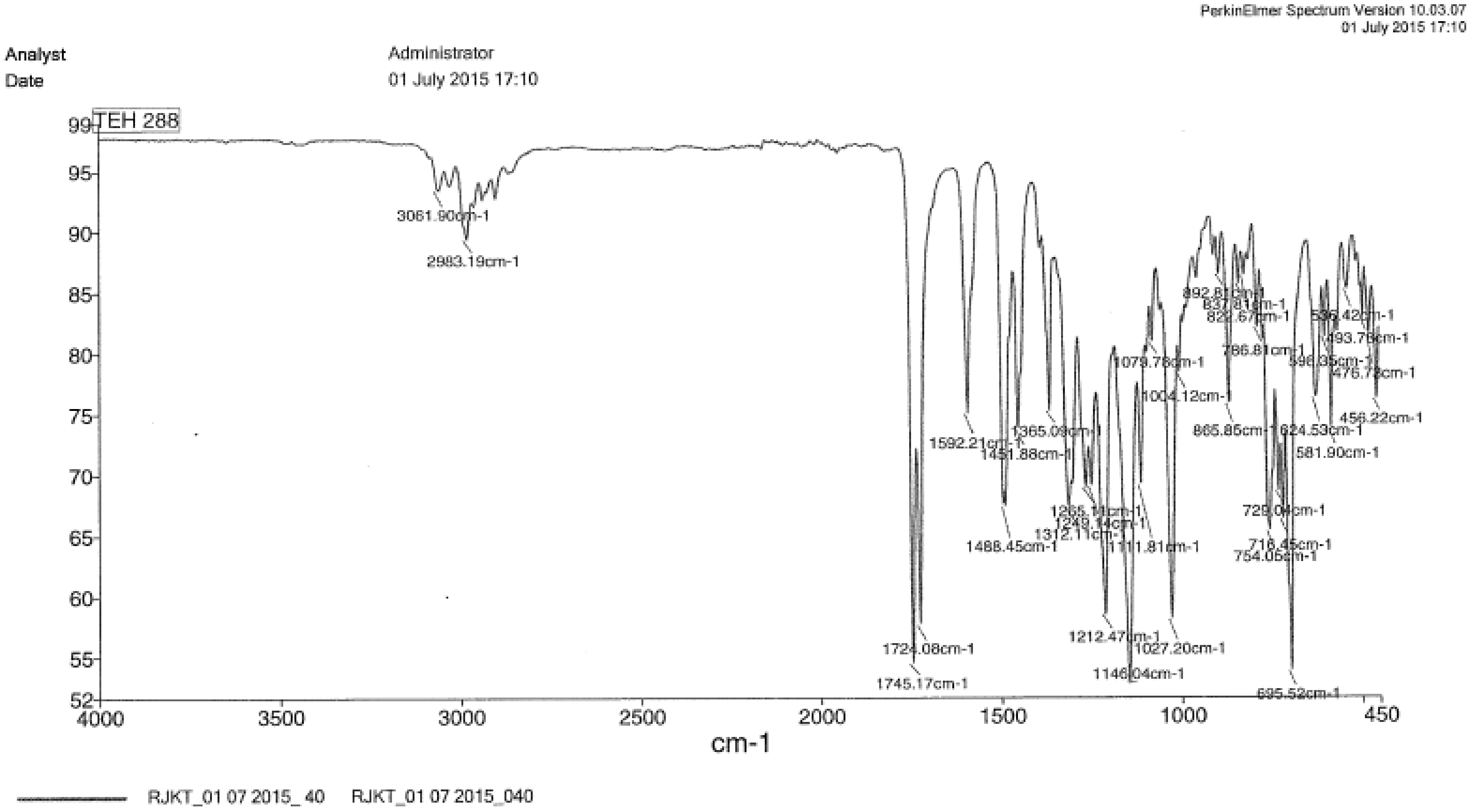
PerkinElmer Spectrum Version 10.03.07
01 July 2015 17:10
Analyst
Date
Administrator
01 July 2015 17:10
TEH 288
3061.90cm-1
2983.19cm-1
1745.17cm-1
1724.08cm-1
1592.21cm-1
1488.45cm-1
1451.88cm-1
1365.09cm-1
1312.11cm-1
1265.11cm-1
1249.14cm-1
1212.47cm-1
1146.04cm-1
1111.81cm-1
1079.78cm-1
1027.20cm-1
1004.12cm-1
892.81cm-1
837.81cm-1
822.67cm-1
865.85cm-1
786.81cm-1
754.05cm-1
729.04cm-1
716.45cm-1
695.52cm-1
624.53cm-1
596.35cm-1
581.90cm-1
536.42cm-1
493.78cm-1
476.72cm-1
456.22cm-1
cm-1
RJKT_01 07 2015_ 40 RJKT_01 07 2015_040

1-Benzyl-3-ethyl 2-(2-[ethyl(phenyl)amino]phenyl)propanedioate 15c

SpinWorks 4: T Hurst TEH 271

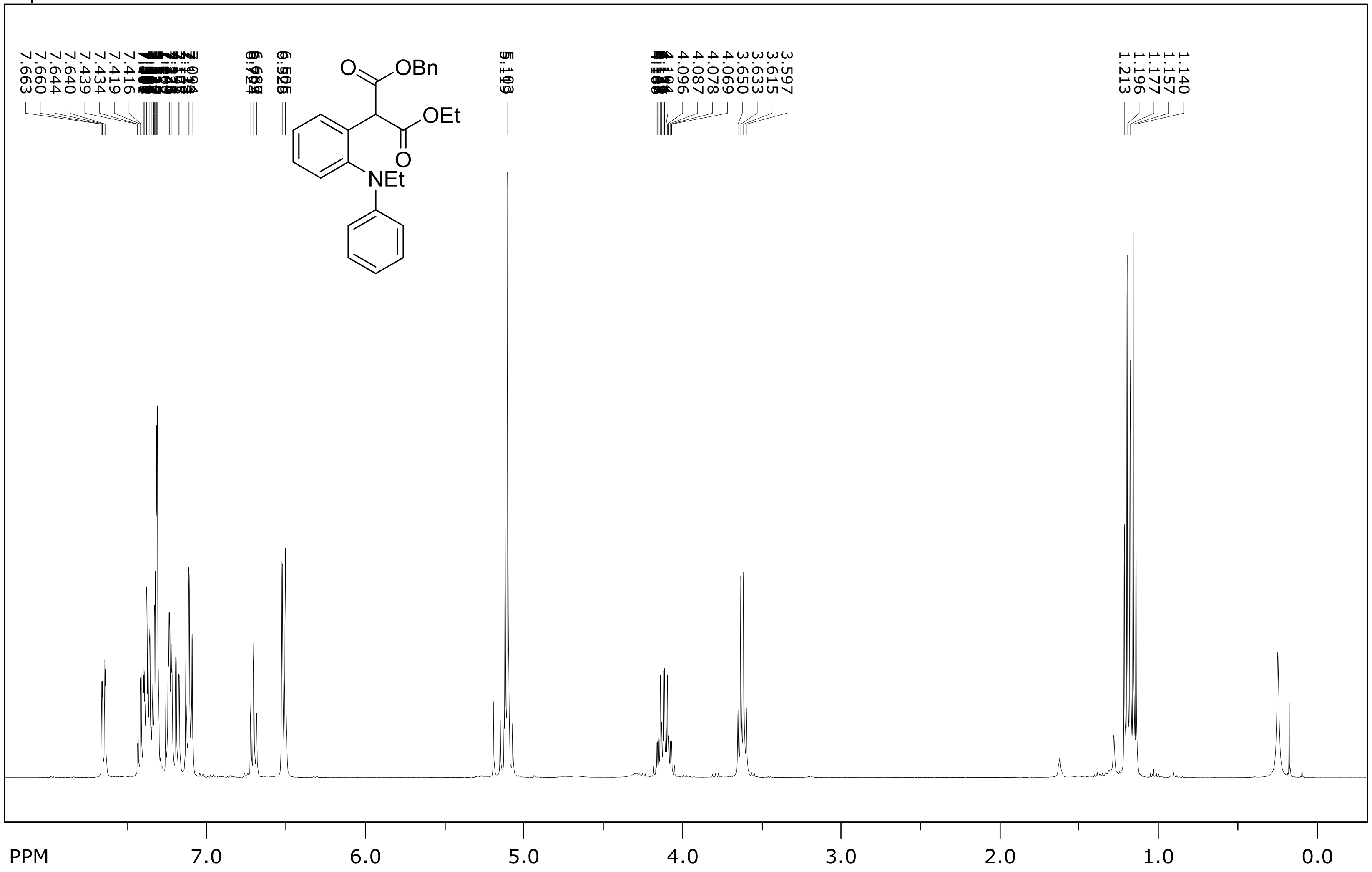

file: ...TEH 271\p6332teh_Proton_ft-1-1.jdf expt: undefined
transmitter freq.: 399.782198 MHz
time domain size: 32768 points
width: 7503.00 Hz = 18.7677 ppm = 0.228973 Hz/pt
number of scans: 8

freq. of 0 ppm: 399.780194 MHz
processed size: 16384 complex points
LB: 0.000 GF: 0.0000

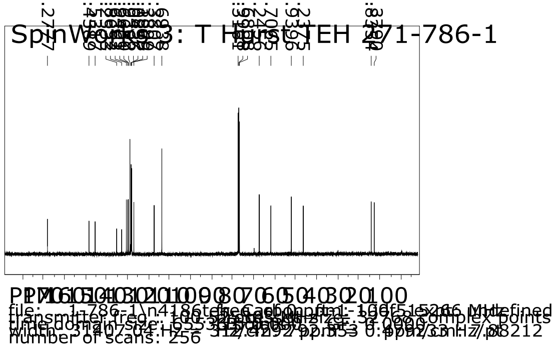
number of scans: 256

teh271

York - Chemistry - Mass Spectrometry Service Report

Analysis Information

Acquisition Date 13/07/2015 12:22:54

Analysis Filename	rjkt53521th_P1-A-5_01_59748.d
Method	600p_meoh1260_2c1s.m
Submission Name	rjkt53521th
Instrument	micrOTOF
ESI	Positive

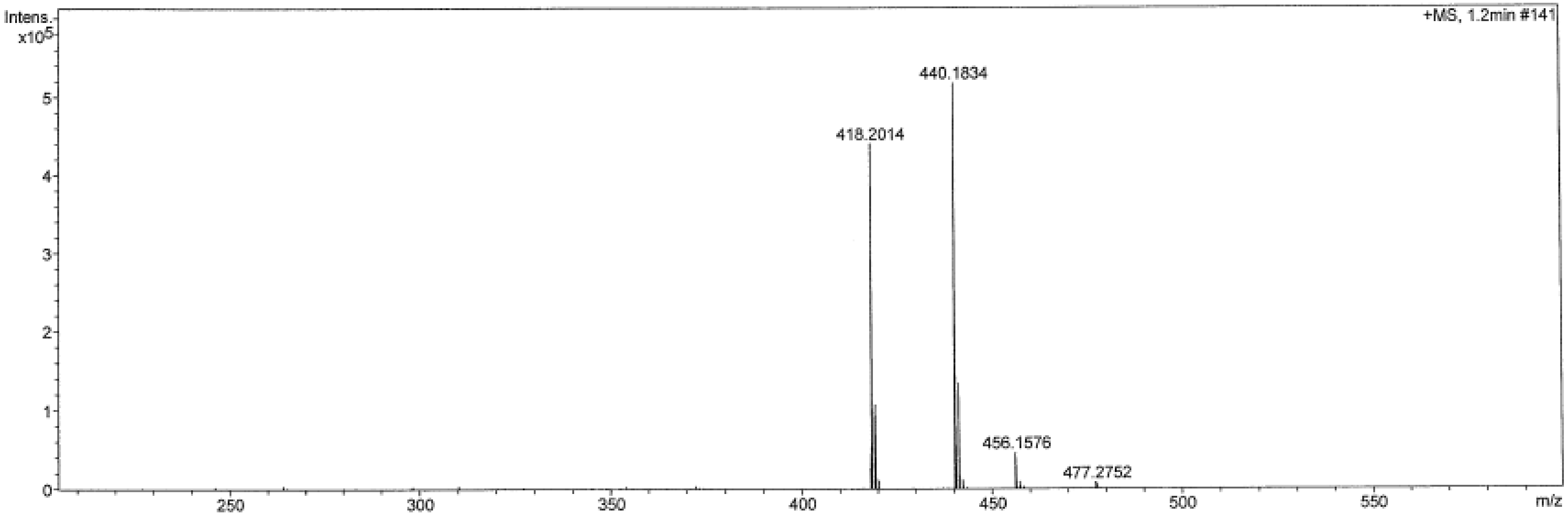

Meas. m/z	#	Formula	m/z	err [ppm]	err [mDa]	mSigma	Mean err [ppm]
418.2014	1	C 26 H 28 N O 4	418.2013	-0.2	-0.1	27.9	0.1
440.1834	1	C 26 H 27 N Na O 4	440.1832	-0.4	-0.2	20.7	-0.2

PerkinElmer Spectrum Version 10.03.07
01 July 2015 17:13

Analyst Administrator
Date 01 July 2015 17:13

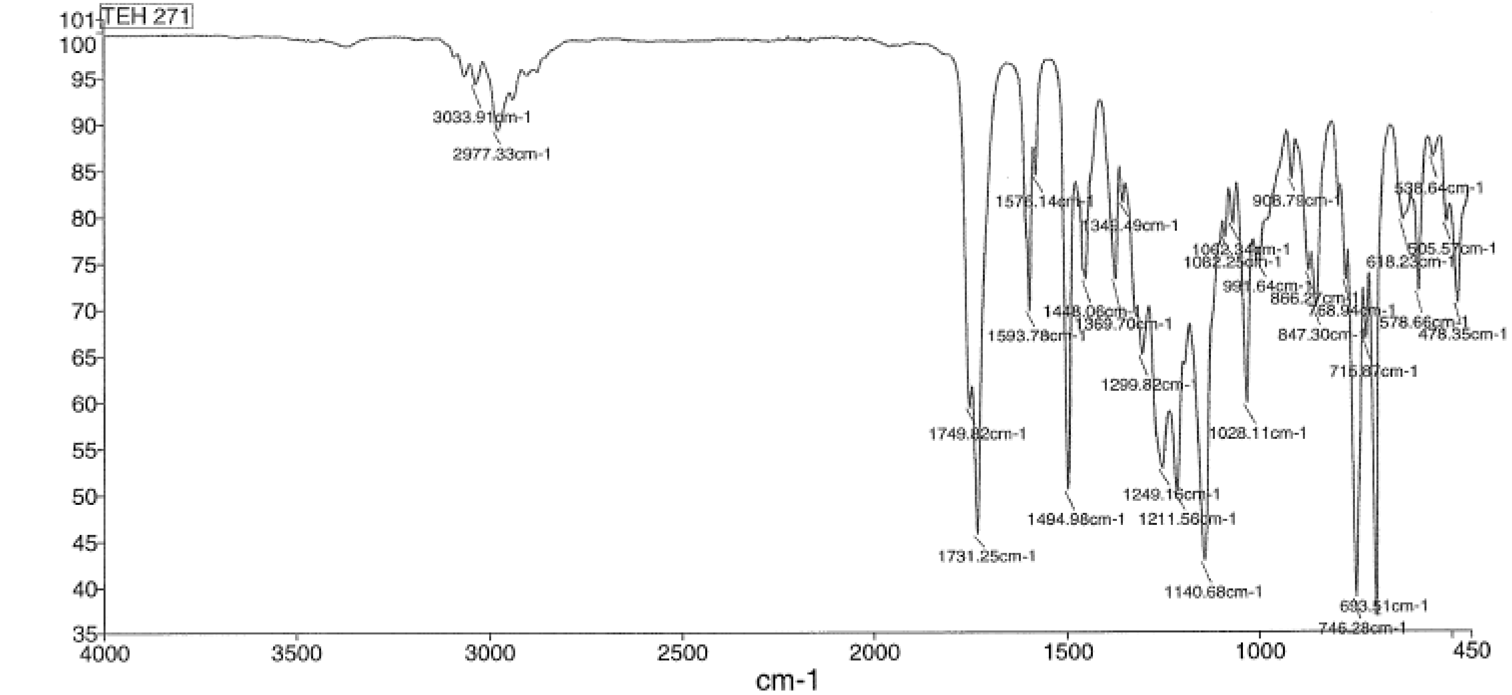

RJKT_01 07 2015_ 41 RJKT_01 07 2015_041

1-Allyl-3-ethyl 2-(2-[ethyl(phenyl)amino]phenyl)propanedioate 15d

SpinWorks 4: T Hurst TEH 272

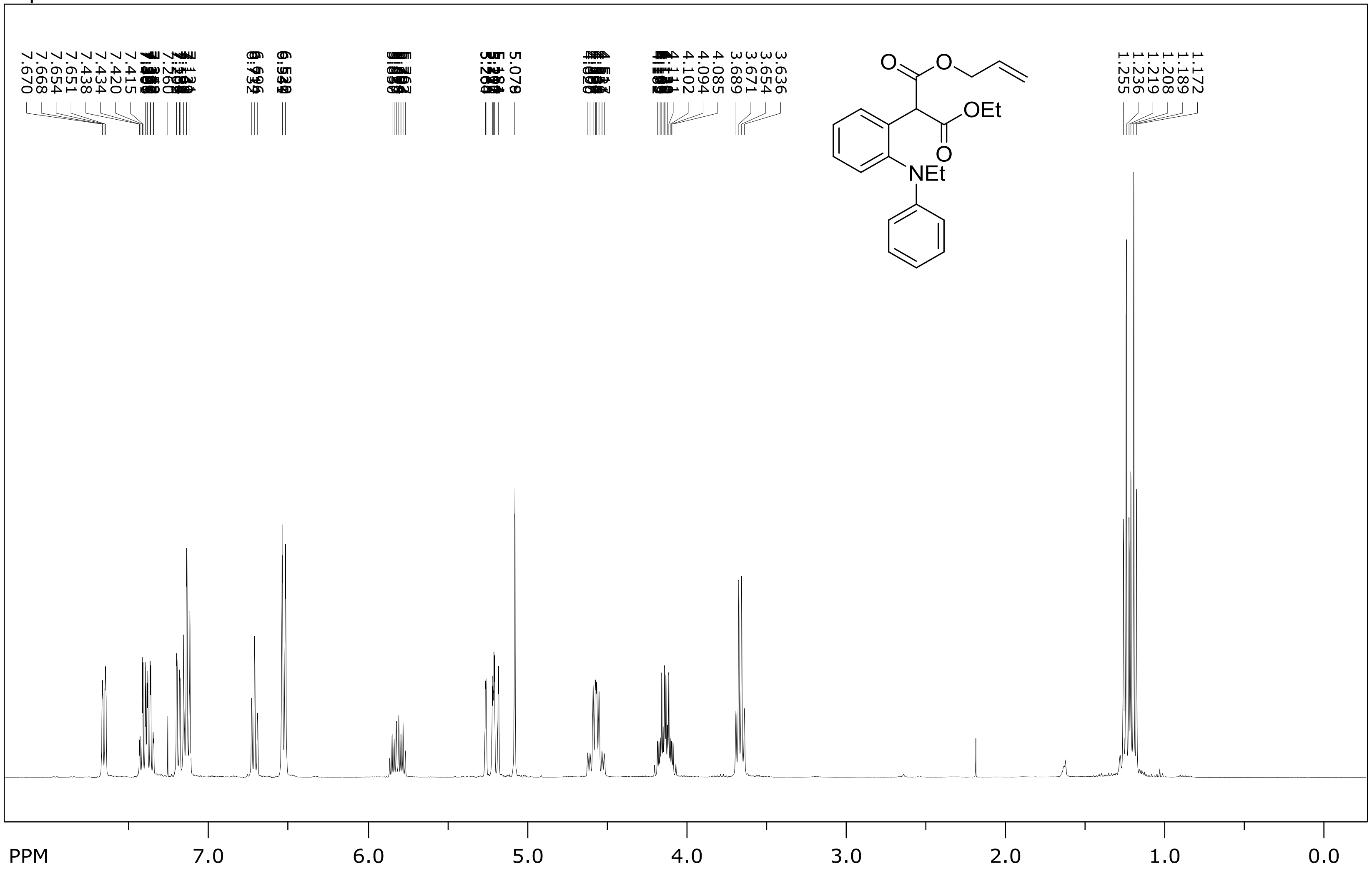

file: ...TEH 272\p5564teh_Proton_ft-1-1.jdf expt: undefined
transmitter freq.: 399.782198 MHz
time domain size: 32768 points
width: 7503.00 Hz = 18.7677 ppm = 0.228973 Hz/pt
number of scans: 8

freq. of 0 ppm: 399.780194 MHz
processed size: 16384 complex points
LB: 0.000 GF: 0.0000

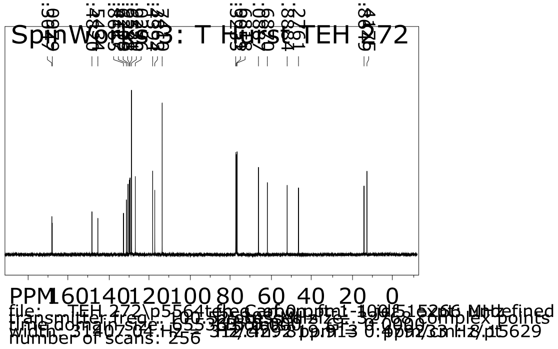
PPM
160 140 120 100 80 60 40 20 0
number of scans: 256

teh272

York - Chemistry - Mass Spectrometry Service Report

Analysis Information

Acquisition Date 13/07/2015 12:27:42

Analysis Filename	rjkt53522th_P1-A-6_01_59749.d
Method	400p_meoh1260_2c1s.m
Submission Name	rjkt53522th
Instrument	micrOTOF
ESI	Positive

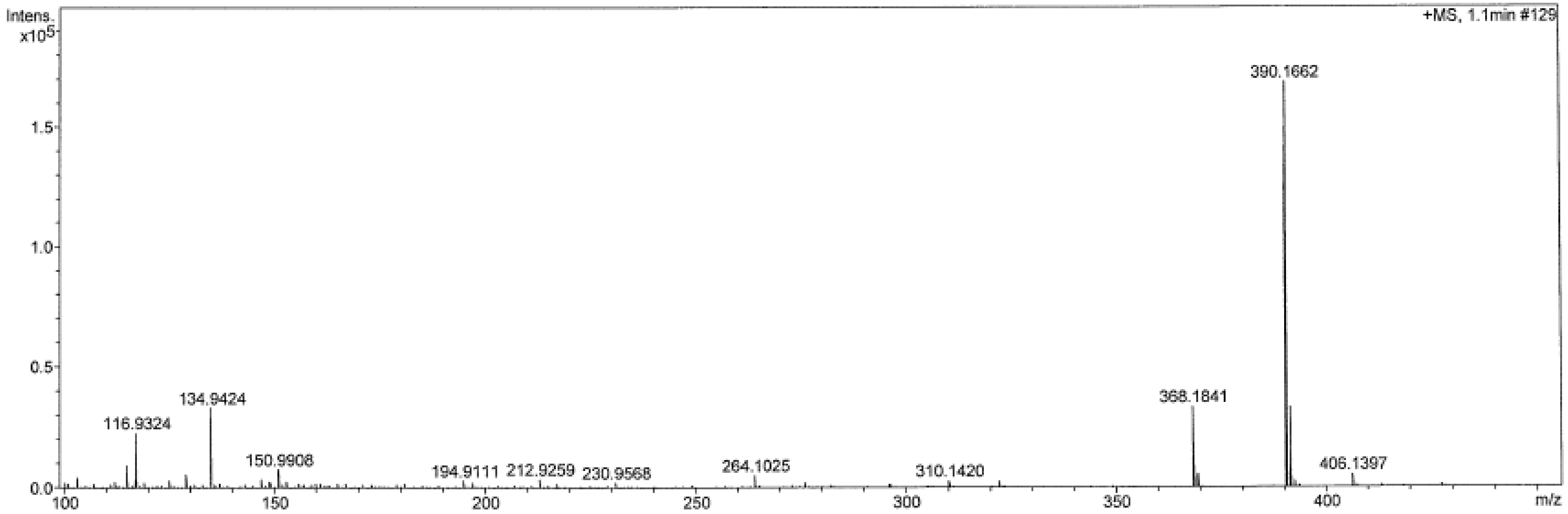

Meas. m/z	#	Formula	m/z	err [ppm]	err [mDa]	mSigma	Mean err [ppm]
368.1841	1	C 22 H 26 N O 4	368.1856	4.1	1.5	50.5	3.9
390.1662	1	C 22 H 25 N Na O 4	390.1676	3.6	1.4	28.6	4.4

PerkinElmer Spectrum Version 10.03.07
01 July 2015 17:15

Analyst Administrator
Date 01 July 2015 17:15

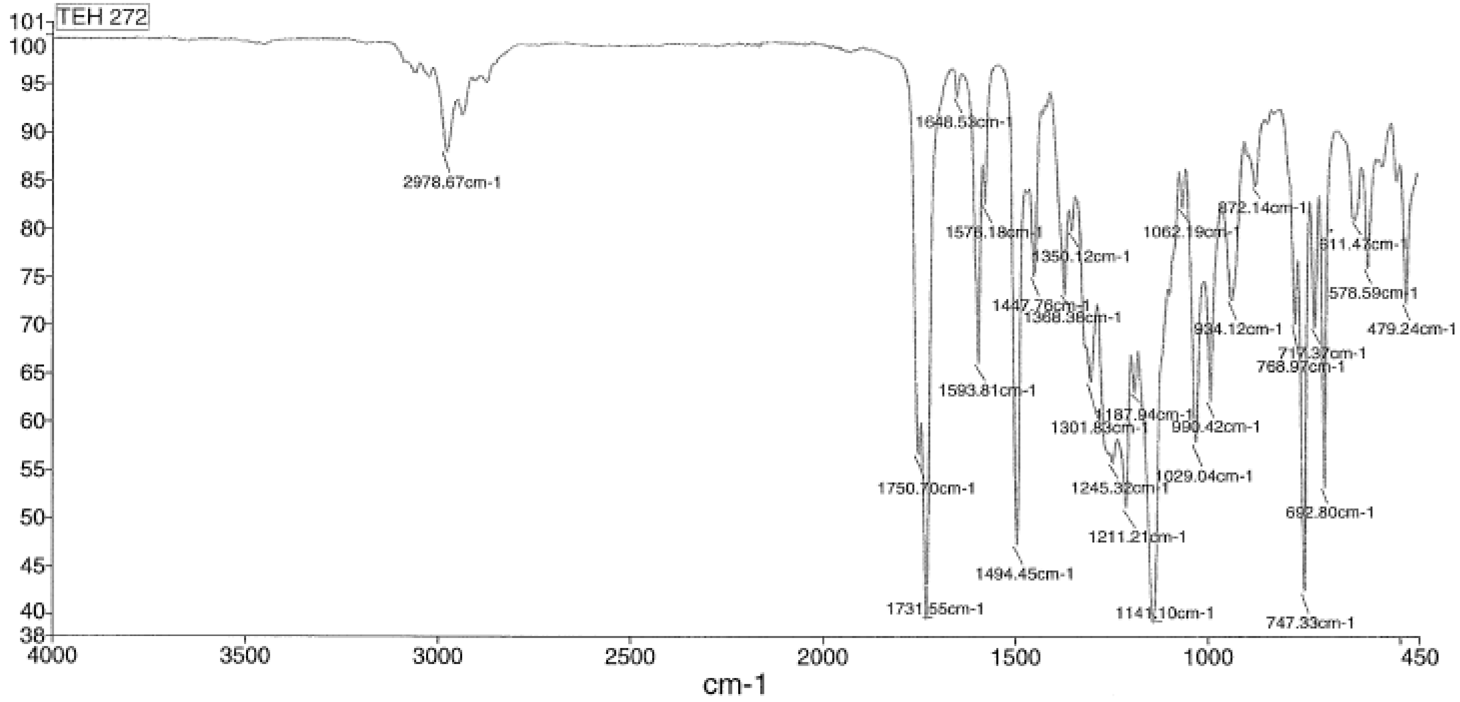

RJKT_01 07 2015_ 42 RJKT_01 07 2015_042

1-Ethyl-3-(9*H*-fluoren-9-ylmethyl) 2-(2-[ethyl(phenyl)amino]phenyl)propanedioate 15e

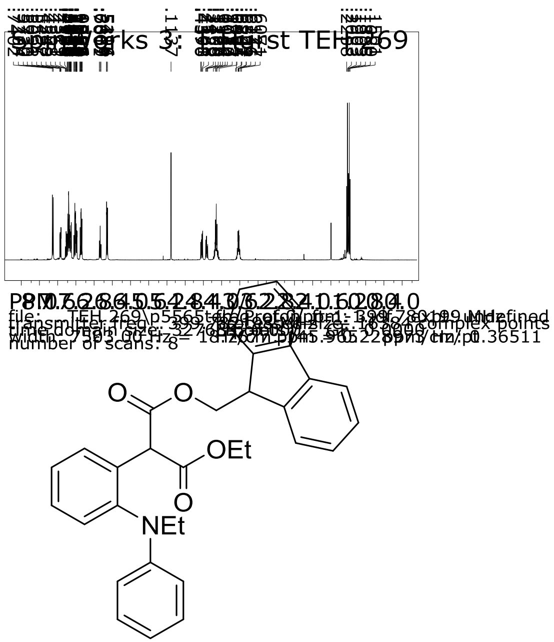

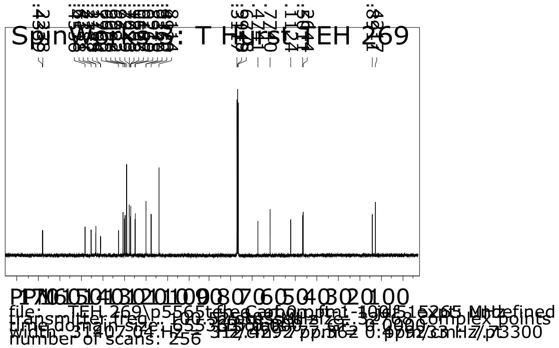

teh269

York - Chemistry - Mass Spectrometry Service Report

Analysis Information

Acquisition Date 13/07/2015 12:30:38

Analysis Filename	rjkt53523th_P1-A-7_01_59750.d
Method	600p_meoh1260_2c1s.m
Submission Name	rjkt53523th
Instrument	micrOTOF
ESI	Positive

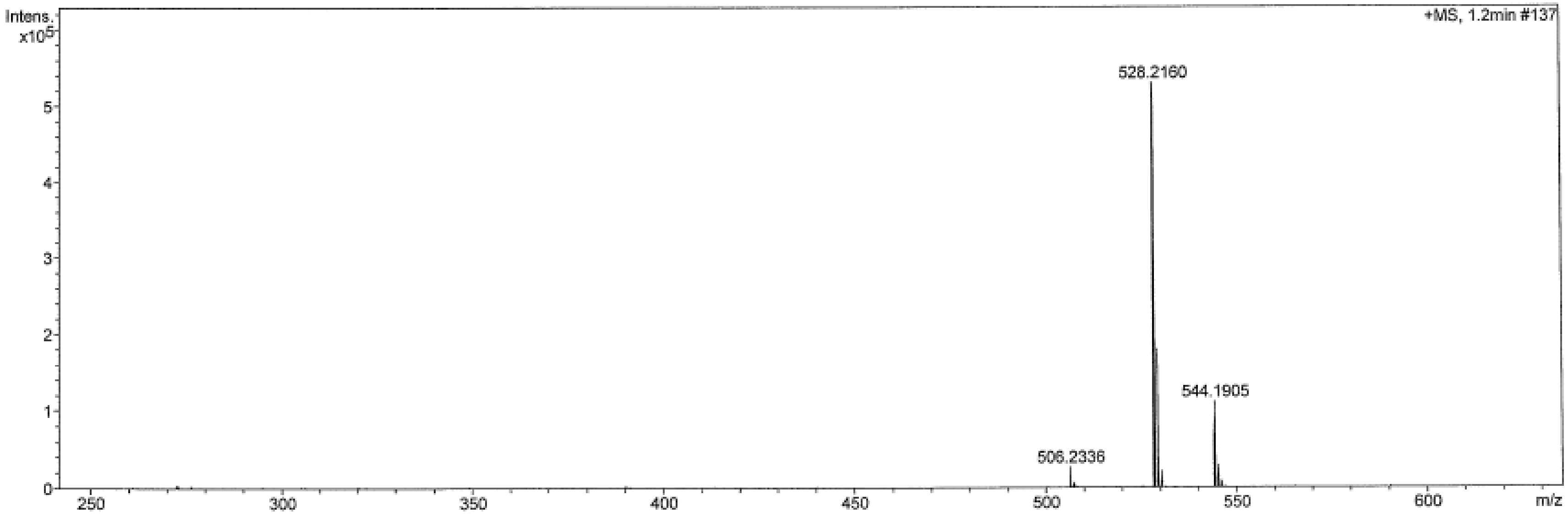

Meas. m/z	#	Formula	m/z	err [ppm]	err [mDa]	mSigma	Mean err [ppm]
506.2336	1	C 33 H 32 N O 4	506.2326	-2.1	-1.1	61.0	-2.4
528.2160	1	C 33 H 31 N Na O 4	528.2145	-2.8	-1.5	20.8	-2.5

PerkinElmer Spectrum Version 10.03.07
01 July 2015 17:18

Analyst Administrator
Date 01 July 2015 17:18

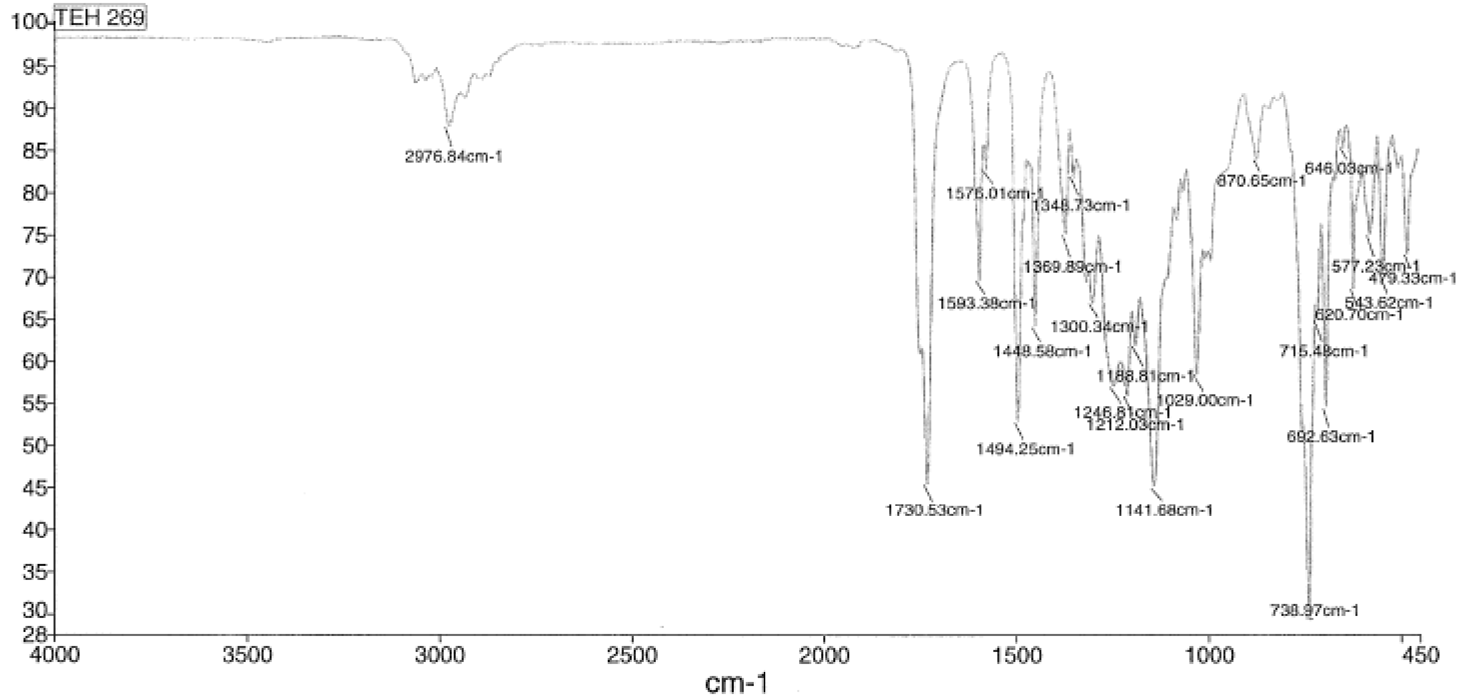

RJKT_01 07 2015_ 43 RJKT_01 07 2015_043

1-Ethyl-3-(*L*-menthyl) 2-(2-[ethyl(phenyl)amino]phenyl)propanedioate 15f

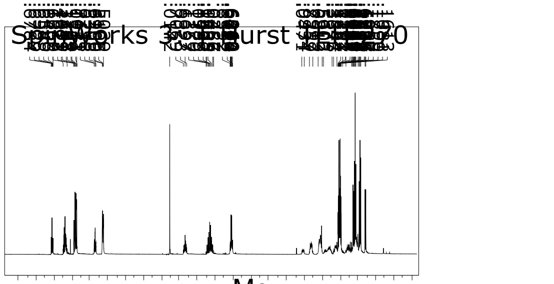

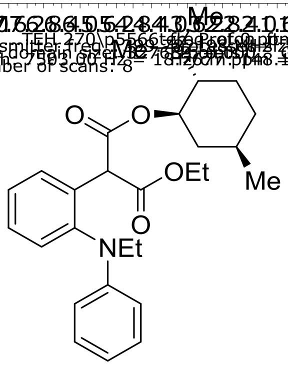

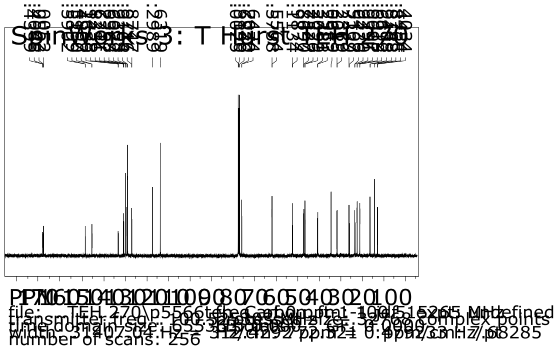

teh270

York - Chemistry - Mass Spectrometry Service Report

Analysis Information

Acquisition Date 13/07/2015 12:33:34

Analysis Filename	rjkt53524th_P1-A-8_01_59751.d
Method	600p_meoh1260_2c1s.m
Submission Name	rjkt53524th
Instrument	micrOTOF
ESI	Positive

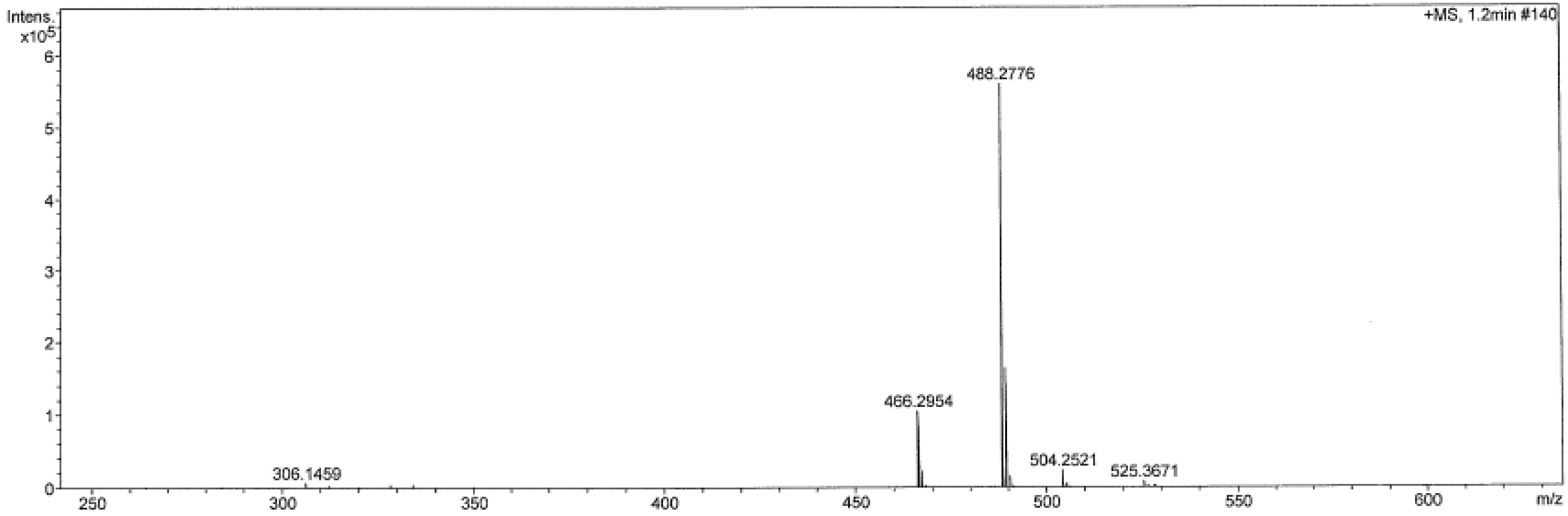

Meas. m/z	#	Formula	m/z	err [ppm]	err [mDa]	mSigma	Mean err [ppm]
466.2954	1	C 29 H 40 N O 4	466.2952	-0.4	-0.2	52.7	-0.7
488.2776	1	C 29 H 39 N Na O 4	488.2771	-0.9	-0.4	20.3	-1.0

PerkinElmer Spectrum Version 10.03.07
01 July 2015 17:22

Analyst Administrator
Date 01 July 2015 17:22

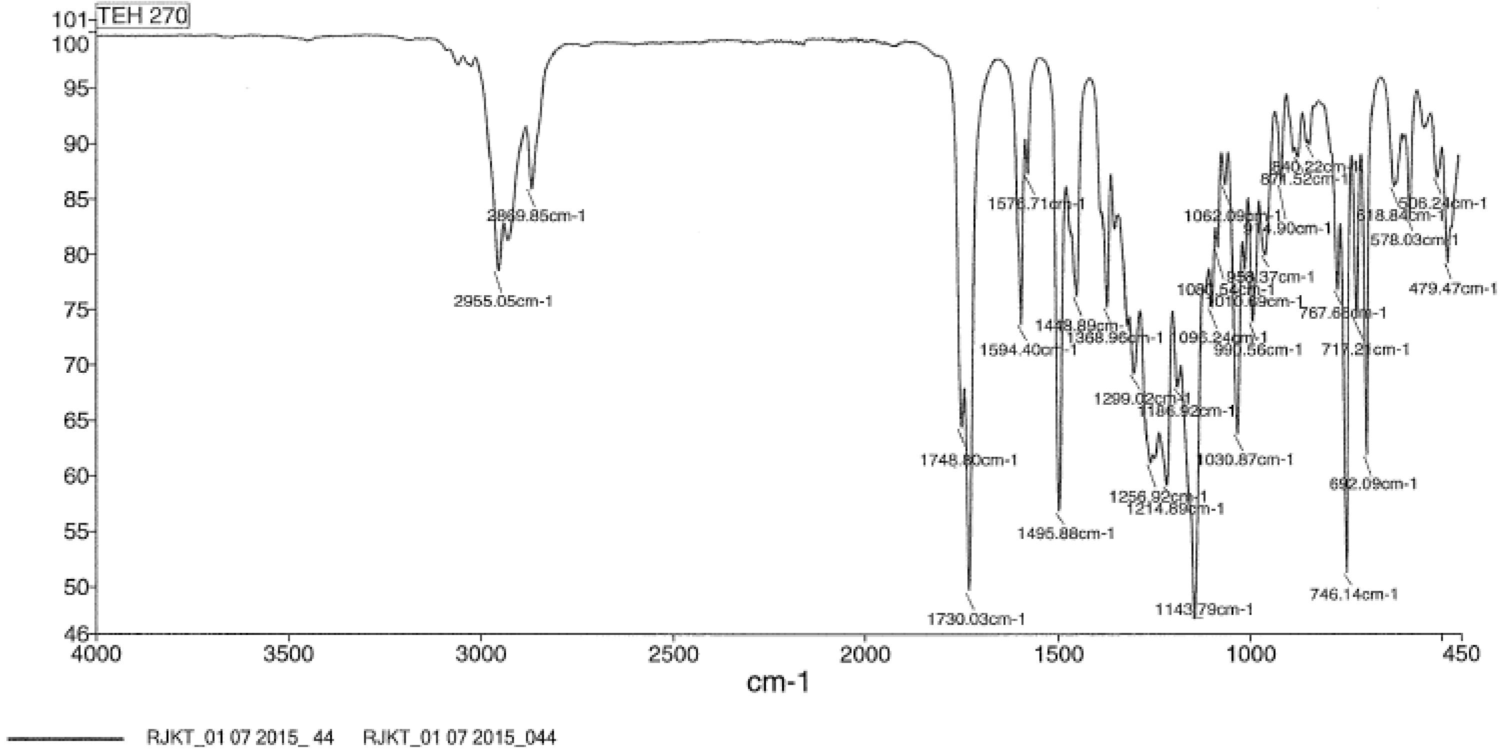

1,2-Diethyl 2-(2-[ethyl(4-fluorophenyl)amino]phenyl)propanedioate 15g

SpinWorks 4: T Hurst TEH 297

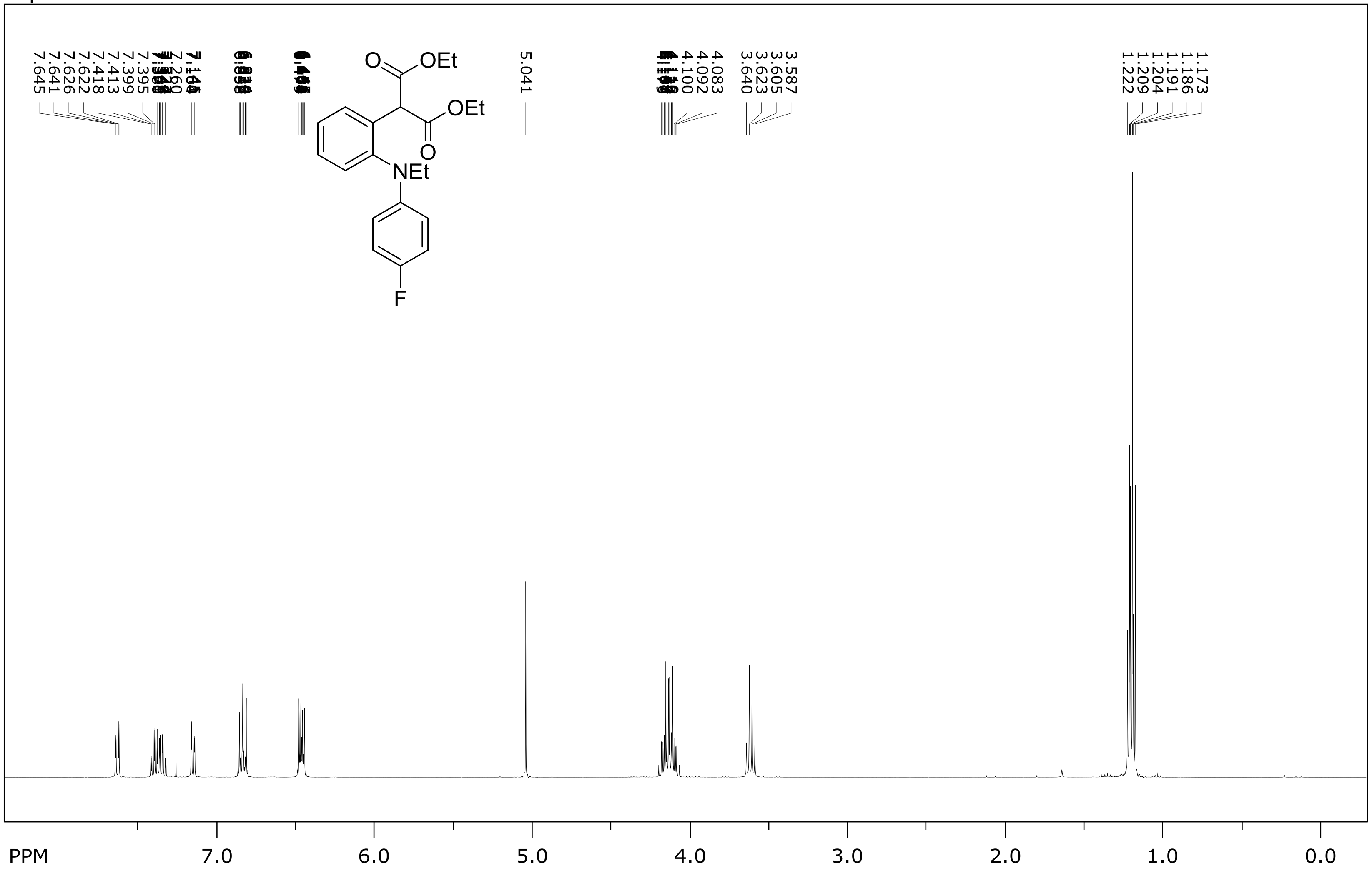

file: ...TEH 297\p6326teh_Proton_ft-1-1.jdf expt: undefined
transmitter freq.: 399.782198 MHz
time domain size: 32768 points
width: 7503.00 Hz = 18.7677 ppm = 0.228973 Hz/pt
number of scans: 8

freq. of 0 ppm: 399.780194 MHz
processed size: 16384 complex points
LB: 0.000 GF: 0.0000

SpinWorks 4: T Hurst TEH 297

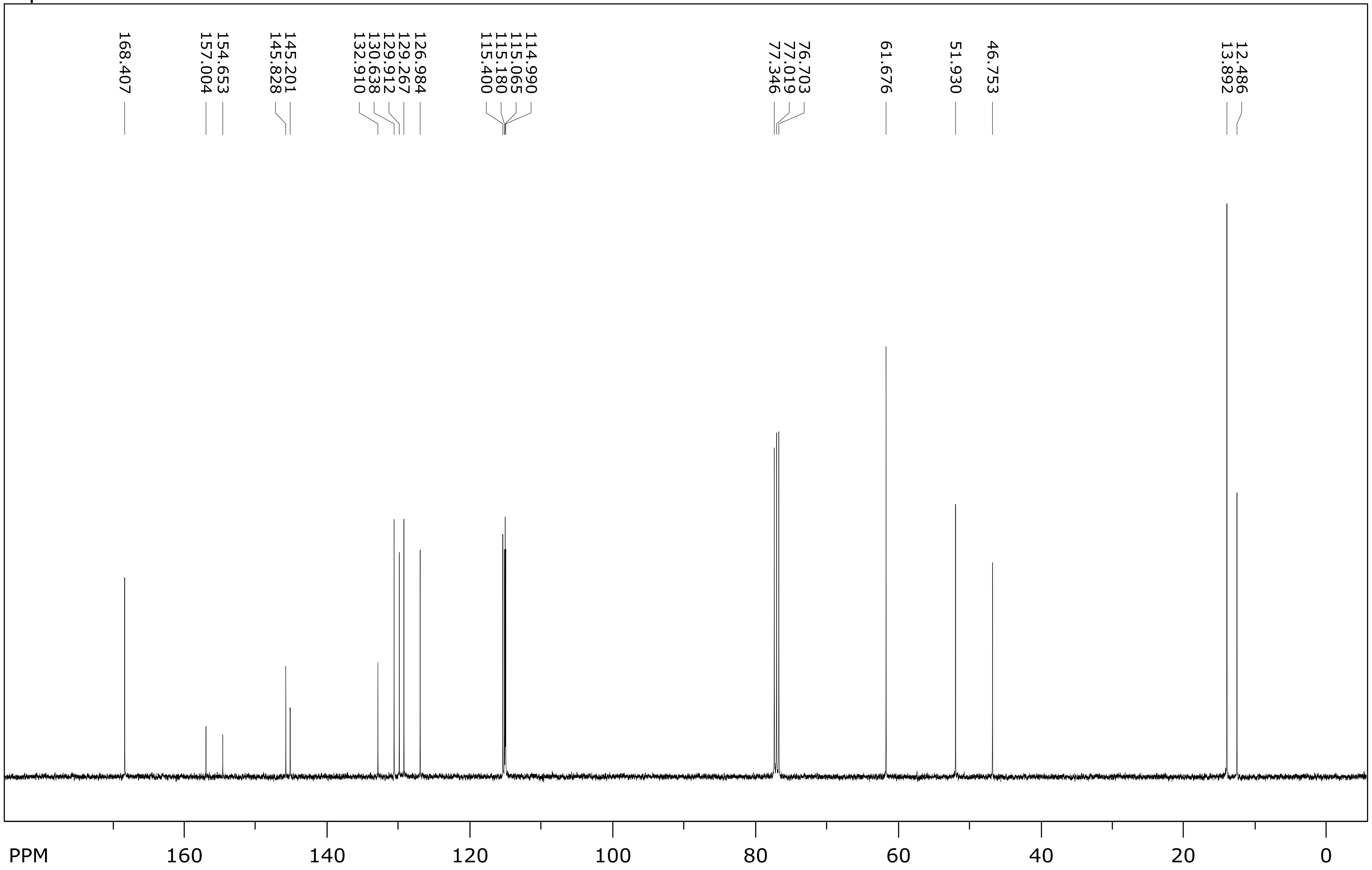

file: ...TEH 297\p6326teh_Carbon_ft-1-1.jdf expt: undefined
transmitter freq.: 100.525303 MHz
time domain size: 65536 points
width: 31407.04 Hz = 312.4292 ppm = 0.479233 Hz/pt
number of scans: 256

freq. of 0 ppm: 100.515263 MHz
processed size: 32768 complex points
LB: 0.000 GF: 0.0000

teh297

York - Chemistry - Mass Spectrometry Service Report

Analysis Information

Acquisition Date 13/07/2015 12:36:30

Analysis Filename	rjkt53525th_P1-A-9_01_59752.d
Method	400p_meoh1260_2c1s.m
Submission Name	rjkt53525th
Instrument	micrOTOF
ESI	Positive

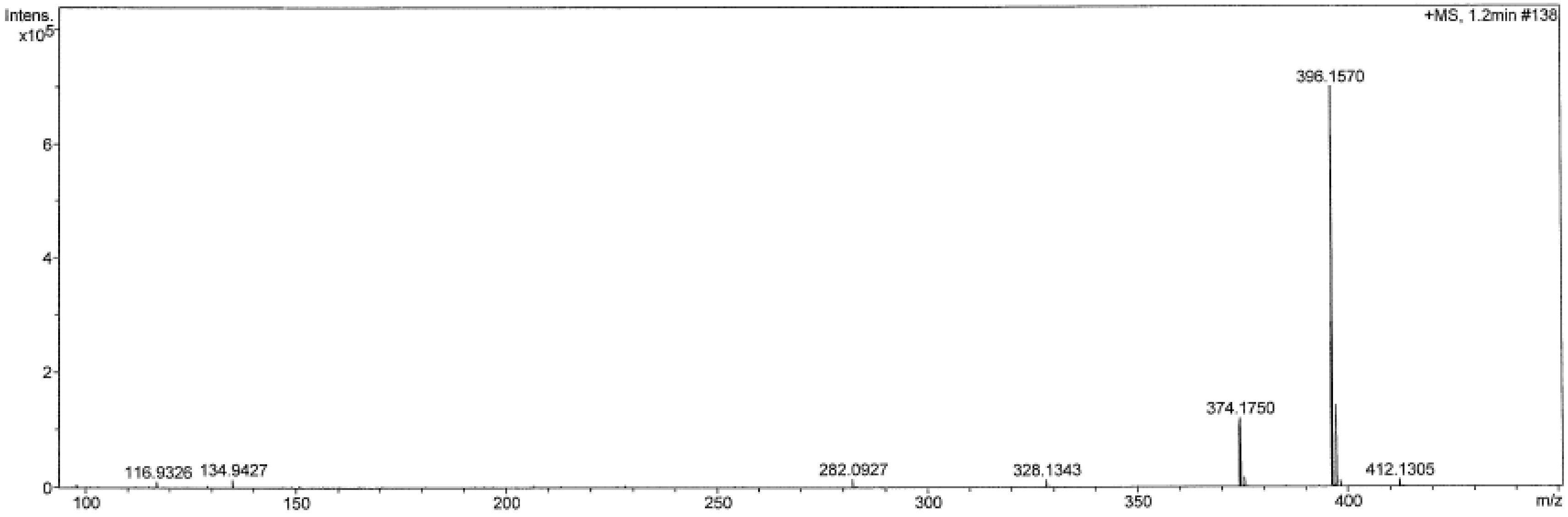

Meas. m/z	#	Formula	m/z	err [ppm]	err [mDa]	mSigma	Mean err [ppm]
374.1750	1	C 21 H 25 F N O 4	374.1762	3.1	1.2	51.2	2.8
396.1570	1	C 21 H 24 F N Na O 4	396.1582	3.0	1.2	19.5	4.2

PerkinElmer Spectrum Version 10.03.07
01 July 2015 17:24

Analyst Administrator
Date 01 July 2015 17:24

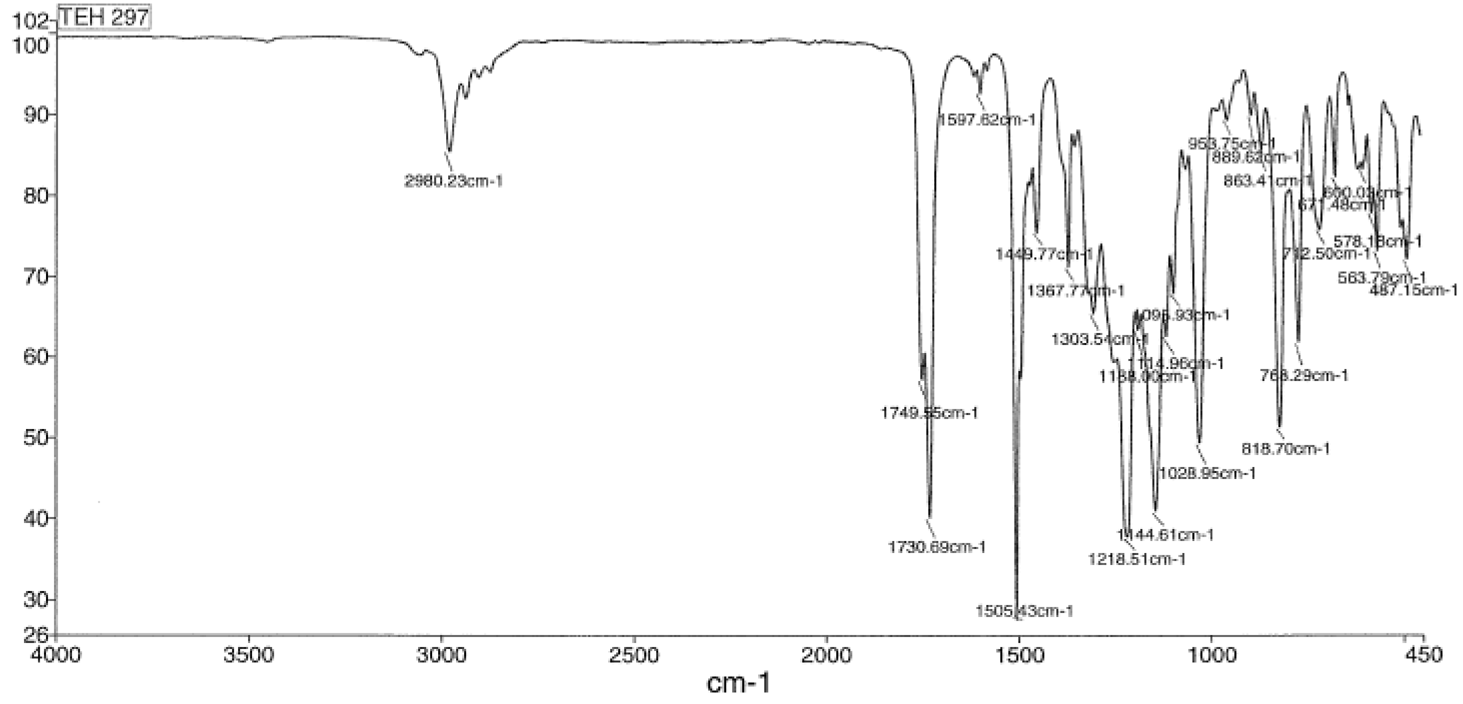

RJKT_01 07 2015_ 45 RJKT_01 07 2015_045

1,2-Diethyl 2-(2-[ethyl(3,5-dichlorophenyl)amino]phenyl)propanedioate 15h

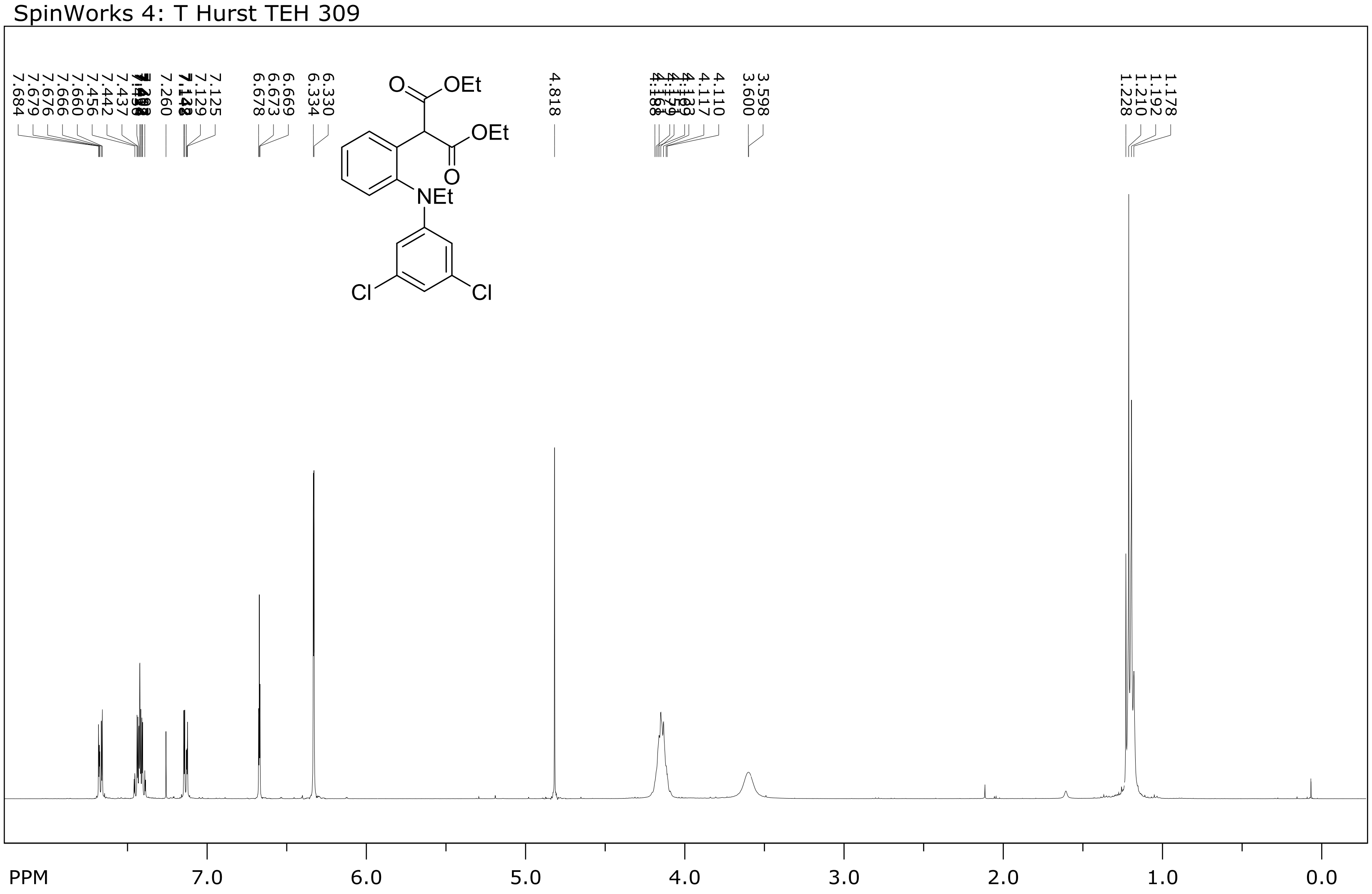

file: ...TEH 309\p6327teh_Proton_ft-1-1.jdf expt: undefined
transmitter freq.: 399.782198 MHz
time domain size: 32768 points
width: 7503.00 Hz = 18.7677 ppm = 0.228973 Hz/pt
number of scans: 8

freq. of 0 ppm: 399.780194 MHz
processed size: 16384 complex points
LB: 0.000 GF: 0.0000

SpinWorks 4: T Hurst TEH 309

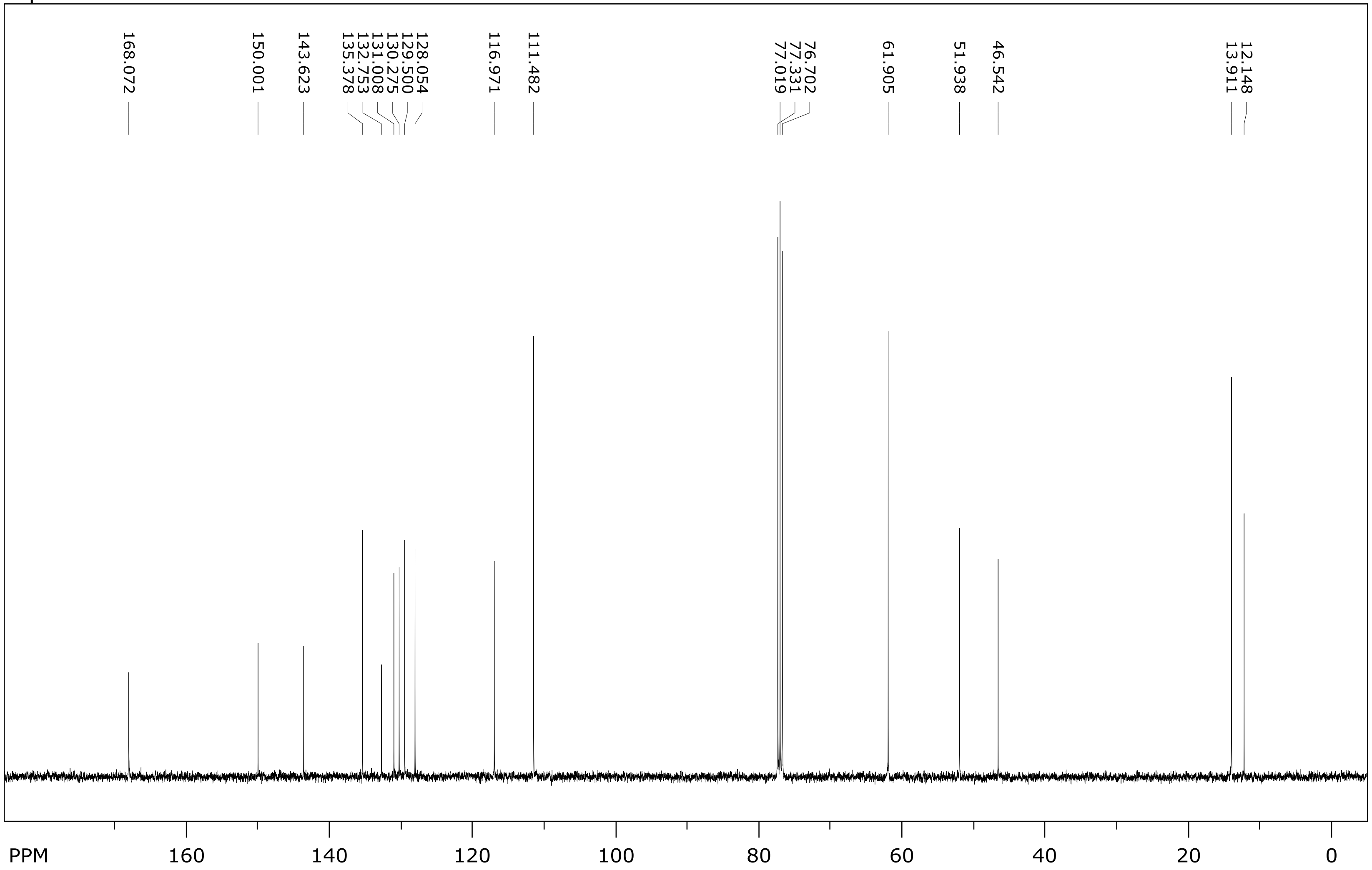

file: ...TEH 309\p6327teh_Carbon_ft-1-1.jdf   expt: undefined
transmitter freq.: 100.525303 MHz
time domain size: 65536 points
width: 31407.04 Hz = 312.4292 ppm = 0.479233 Hz/pt
number of scans: 256

freq. of 0 ppm: 100.515261 MHz
processed size: 32768 complex points
LB: 0.000   GF: 0.0000

teh309

York - Chemistry - Mass Spectrometry Service Report

Analysis Information

Acquisition Date 17/07/2015 11:19:35

Analysis Filename	rjkt53626th_P1-E-6_01_59861.d
Method	600p_meoh1260_2c1s.m
Submission Name	rjkt53626th
Instrument	micrOTOF
ESI	Positive

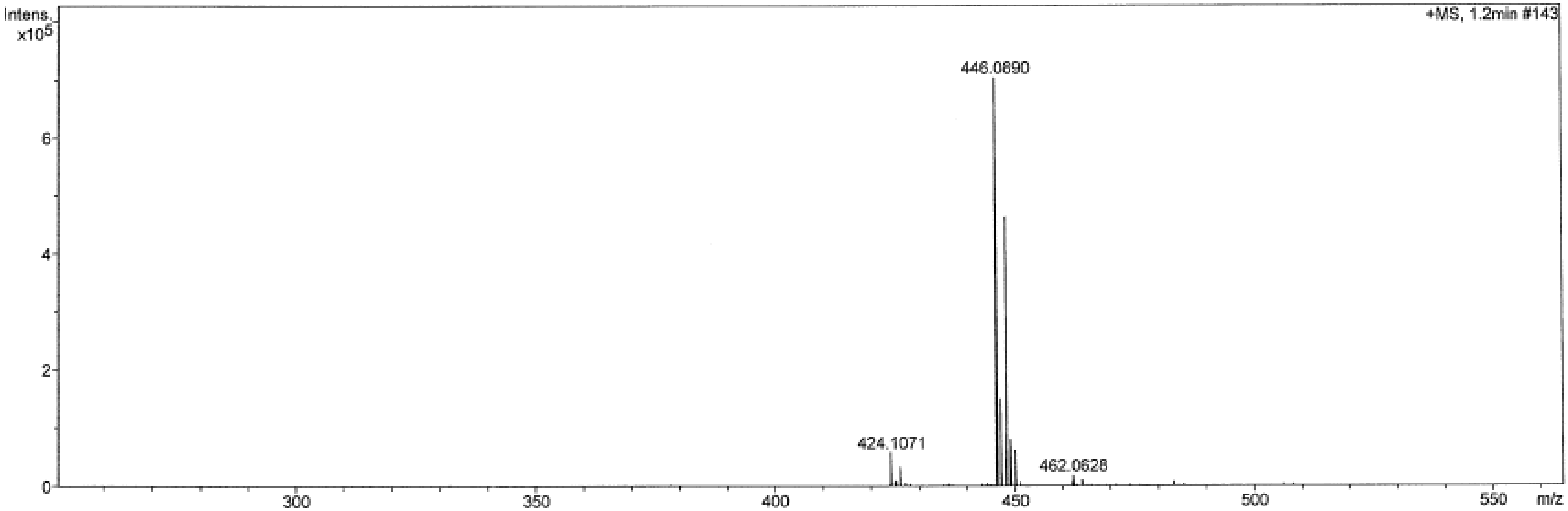

Meas. m/z	#	Formula	m/z	err [ppm]	err [mDa]	mSigma	Mean err [ppm]
424.1071	1	C 21 H 24 Cl 2 N O 4	424.1077	1.4	0.6	60.2	0.8
446.0890	1	C 21 H 23 Cl 2 N Na O 4	446.0896	1.4	0.6	23.4	1.3

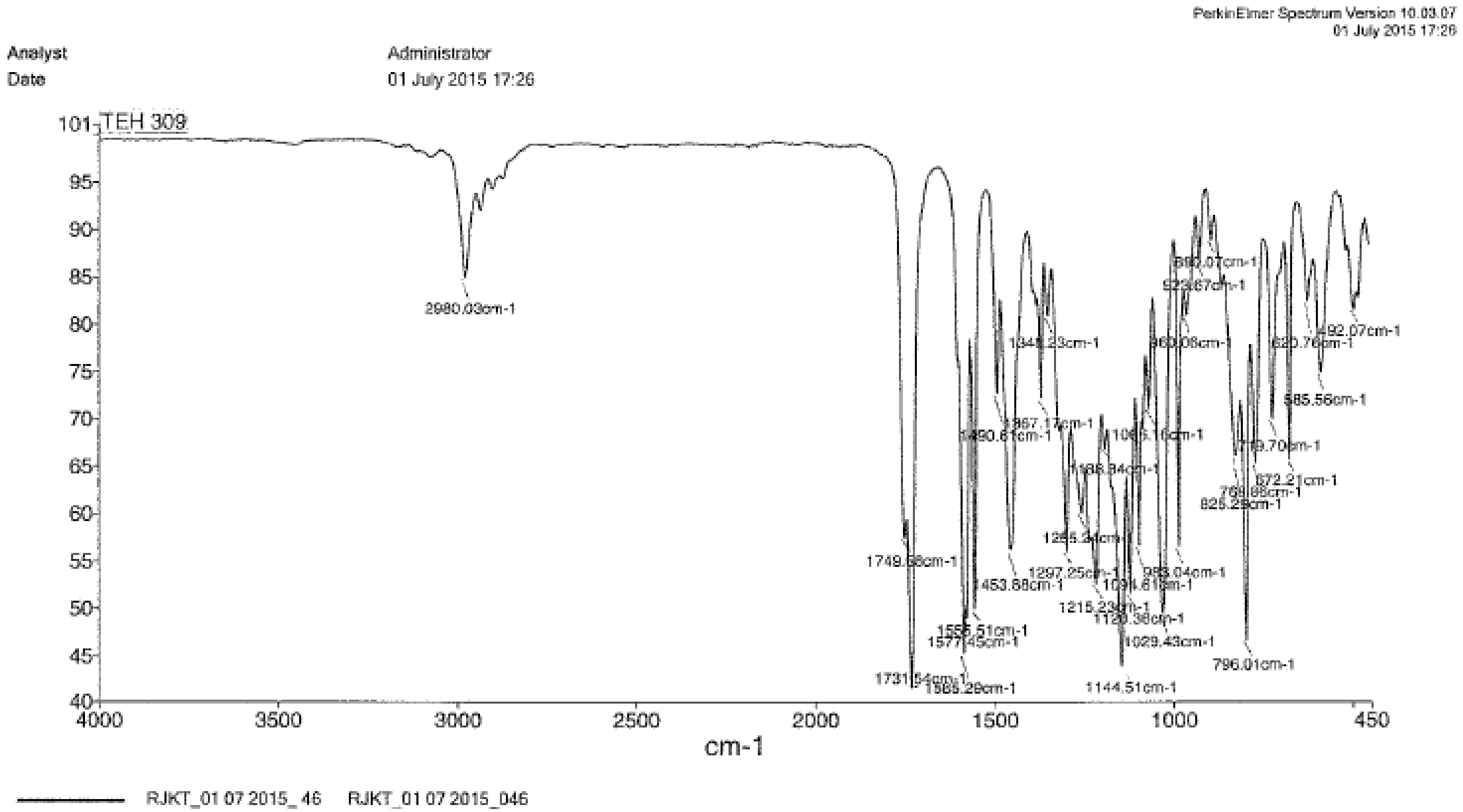
PerkinElmer Spectrum Version 10.03.07
01 July 2015 17:26
Analyst
Administrator
Date
01 July 2015 17:26
TEH 309
2980.03cm-1
1749.68cm-1
1731.54cm-1
1585.29cm-1
1577.45cm-1
1555.51cm-1
1453.88cm-1
1144.51cm-1
1029.43cm-1
796.01cm-1
825.29cm-1
672.21cm-1
585.56cm-1
492.07cm-1
960.06cm-1
cm-1
RJKT_01 07 2015_ 46 RJKT_01 07 2015_046

1,2-Diethyl 2-(2-[ethyl(3,5-dimethylphenyl)amino]phenyl)propanedioate 15i

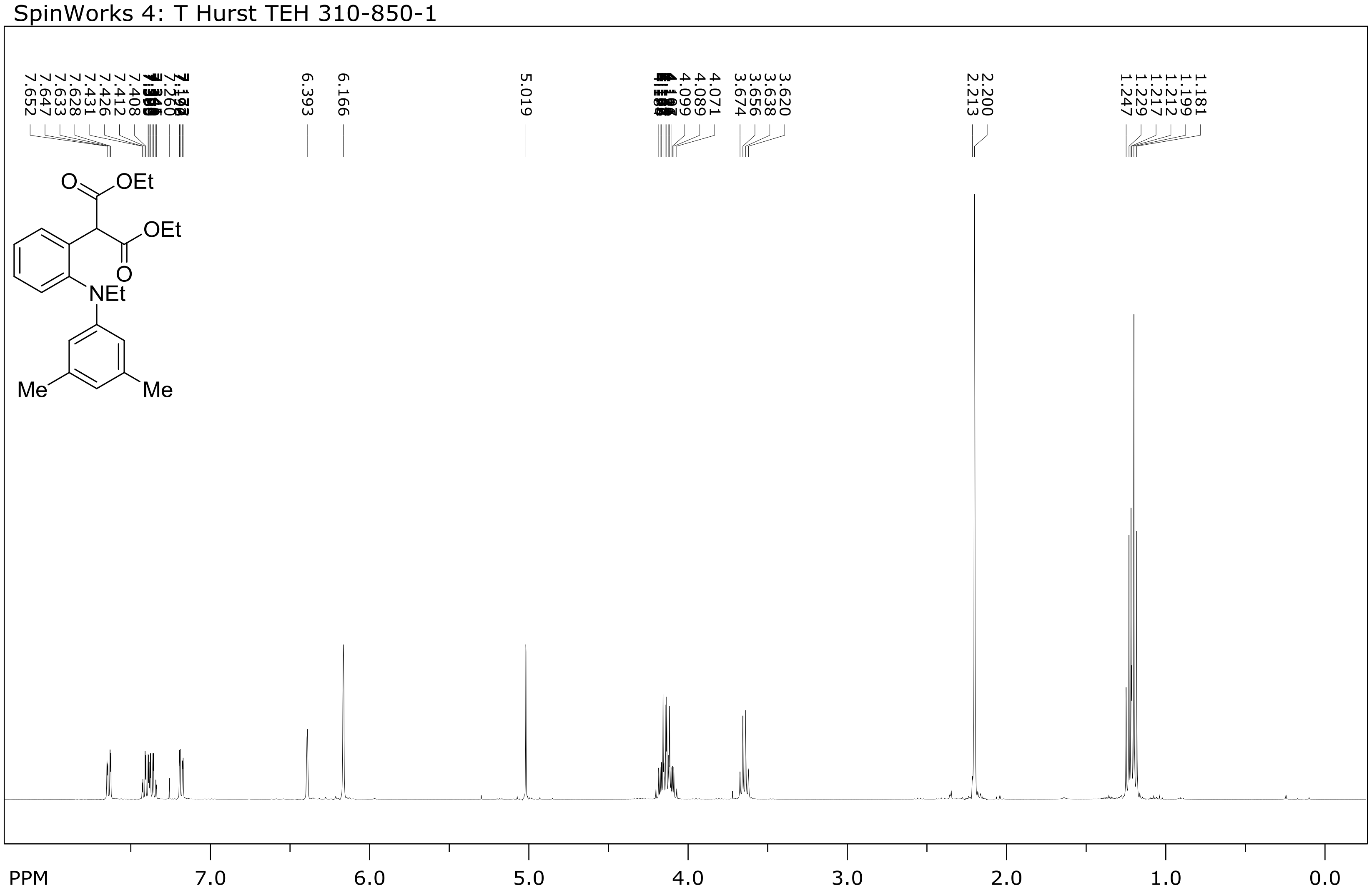

file: ...0-850-1\n7154teh_Proton_ft-1-1.jdf   expt: undefined
transmitter freq.: 399.782198 MHz
time domain size: 32768 points
width: 7503.00 Hz = 18.7677 ppm = 0.228973 Hz/pt
number of scans: 8

freq. of 0 ppm: 399.780194 MHz
processed size: 16384 complex points
LB: 0.000   GF: 0.0000

SpinWorks 4: T Hurst TEH 310-850-1

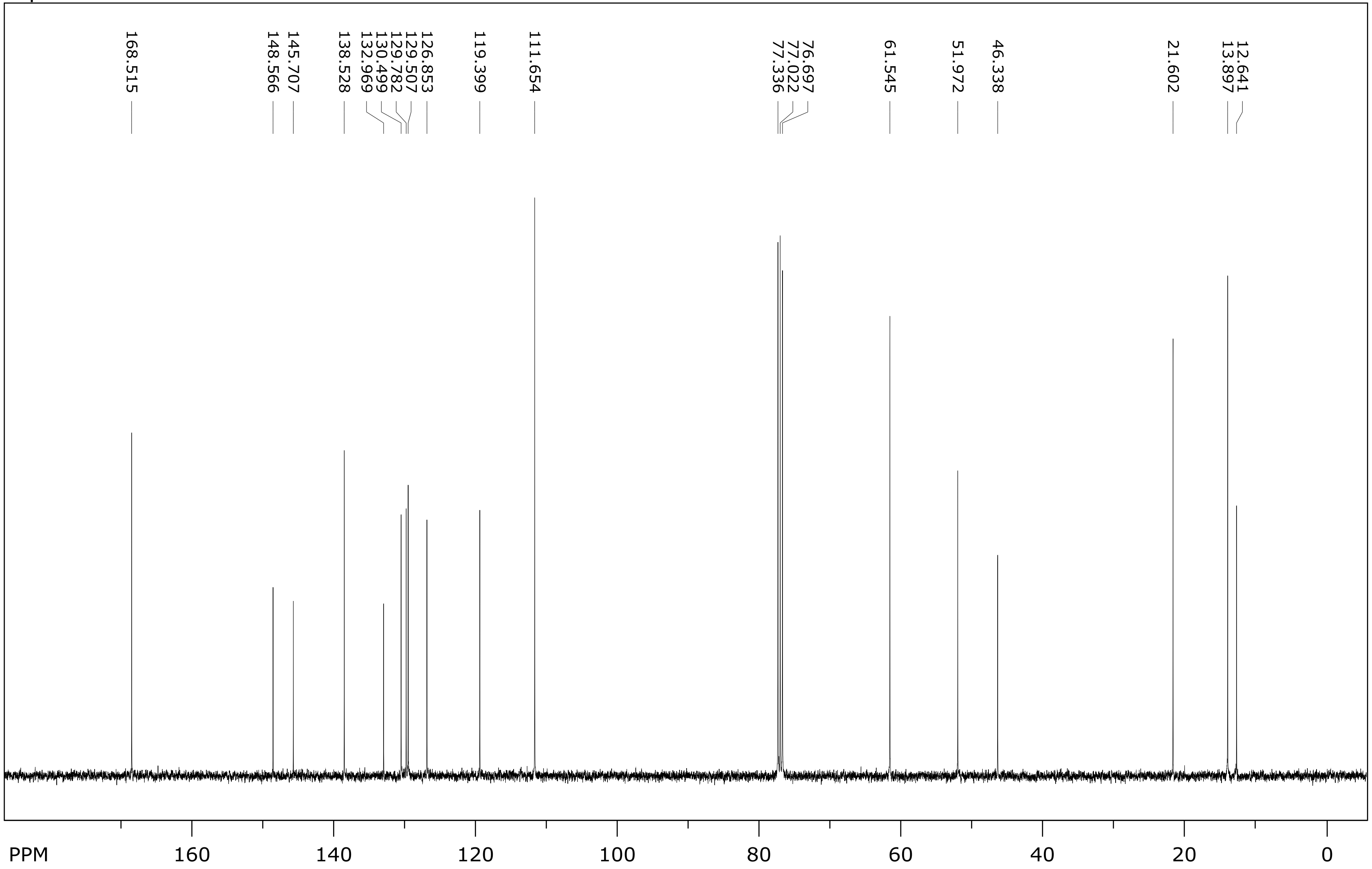

file: ...0-850-1\n7154teh_Carbon_ft-1-1.jdf expt: undefined
transmitter freq.: 100.525303 MHz
time domain size: 65536 points
width: 31407.04 Hz = 312.4292 ppm = 0.479233 Hz/pt
number of scans: 256

freq. of 0 ppm: 100.515264 MHz
processed size: 32768 complex points
LB: 0.000 GF: 0.0000

teh310

York - Chemistry - Mass Spectrometry Service Report

Analysis Information Acquisition Date 17/07/2015 11:22:32

Analysis Filename	rjkt53627th_P1-E-7_01_59862.d
Method	400p_meoh1260_2c1s.m
Submission Name	rjkt53627th
Instrument	micrOTOF
ESI	Positive

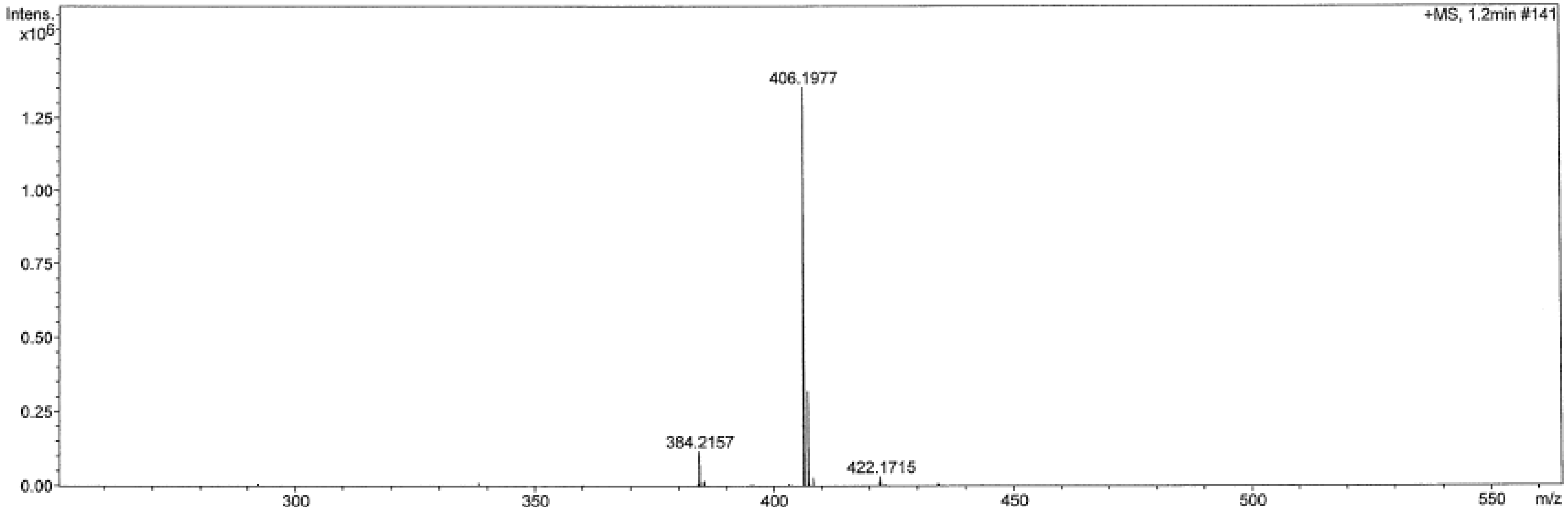

Meas. m/z	#	Formula	m/z	err [ppm]	err [mDa]	mSigma	Mean err [ppm]
384.2157	1	C 23 H 30 N O 4	384.2169	3.3	1.3	53.9	3.2
406.1977	1	C 23 H 29 N Na O 4	406.1989	2.9	1.2	14.9	4.2

PerkinElmer Spectrum Version 10.03.07
01 July 2015 17:29

Analyst Administrator
Date 01 July 2015 17:29

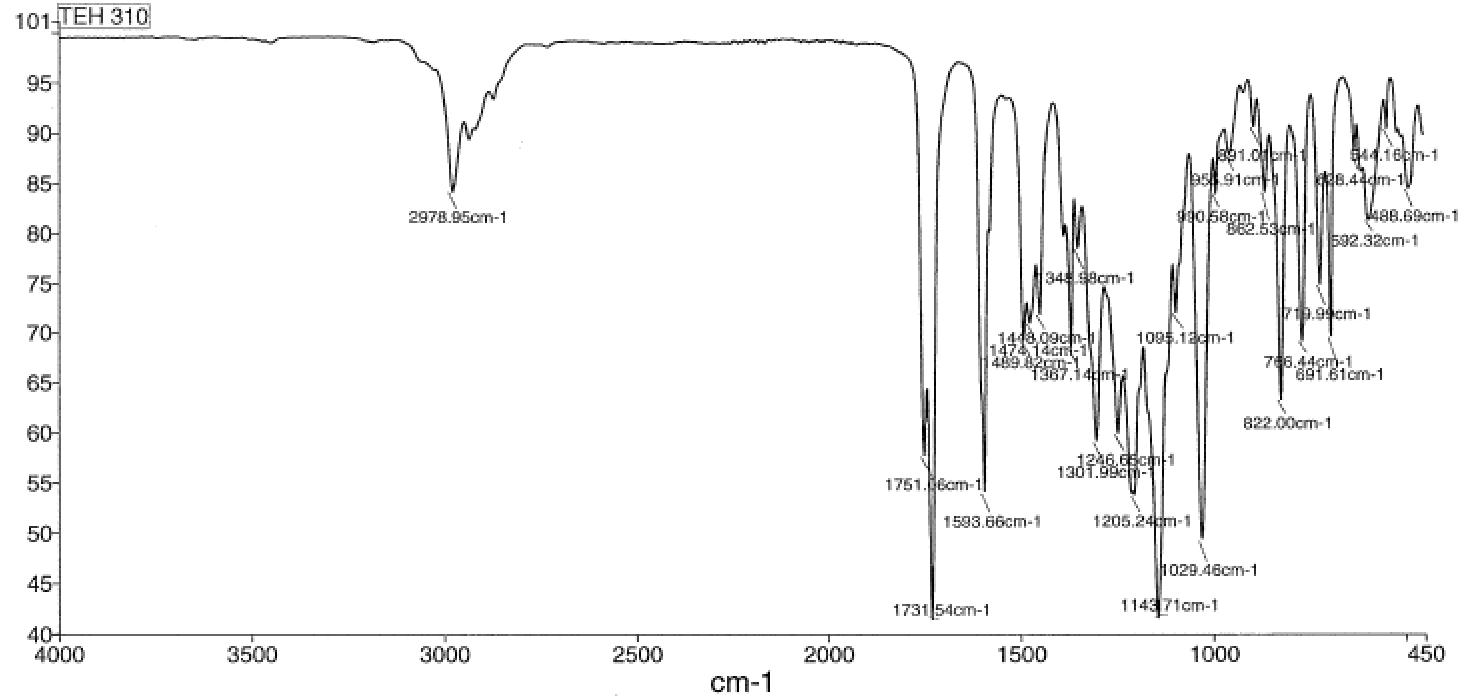

RJKT_01 07 2015_ 47 RJKT_01 07 2015_047

1,2-Diethyl 2-(4-chloro-2-[ethyl(4-methoxyphenyl)amino]phenyl)propanedioate 15j

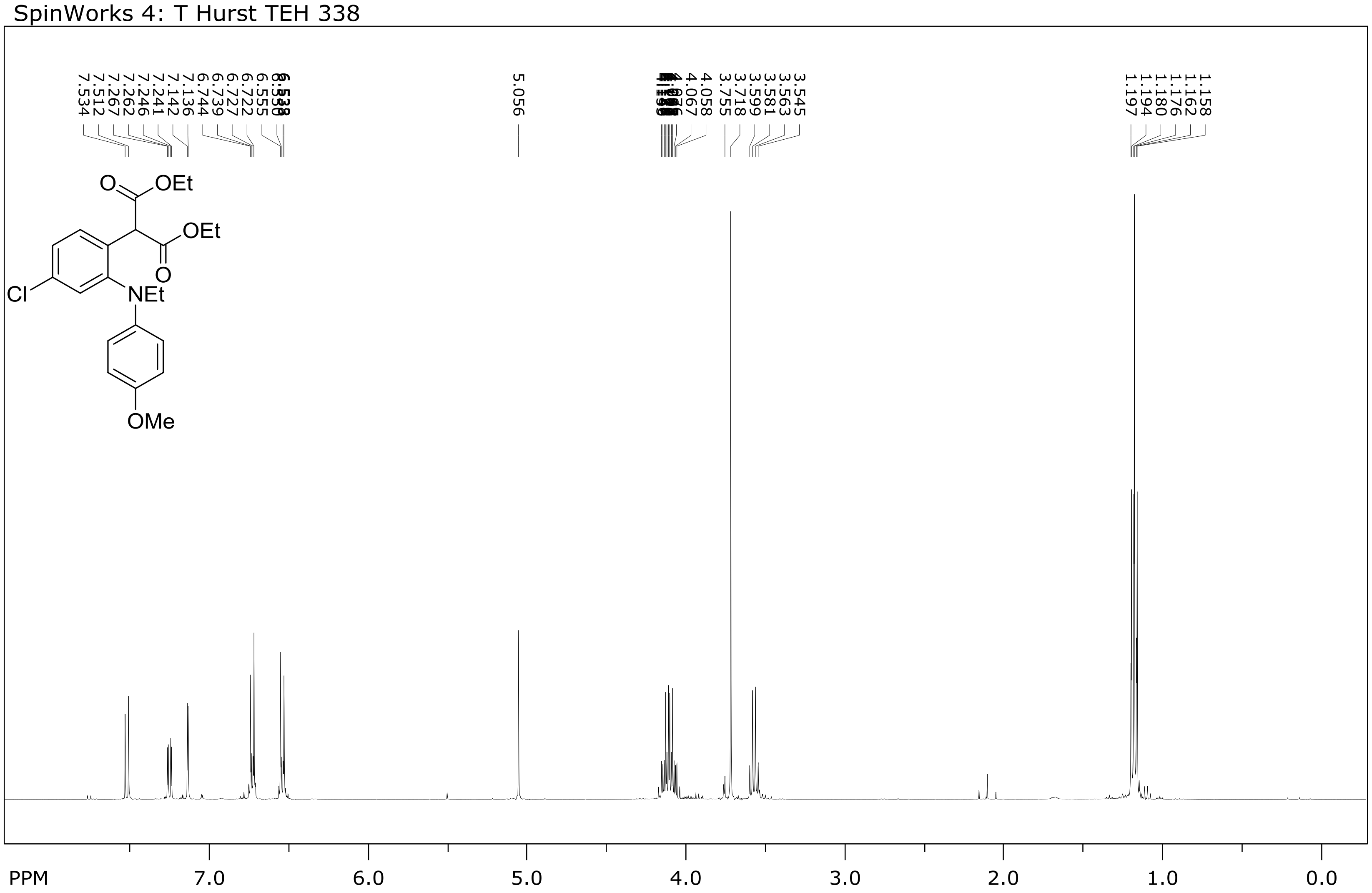

file: ...TEH 338\p6551teh_Proton_ft-1-1.jdf expt: undefined
transmitter freq.: 399.782198 MHz
time domain size: 32768 points
width: 7503.00 Hz = 18.7677 ppm = 0.228973 Hz/pt
number of scans: 8

freq. of 0 ppm: 399.780199 MHz
processed size: 16384 complex points
LB: 0.000 GF: 0.0000

SpinWorks 4: T Hurst TEH 338

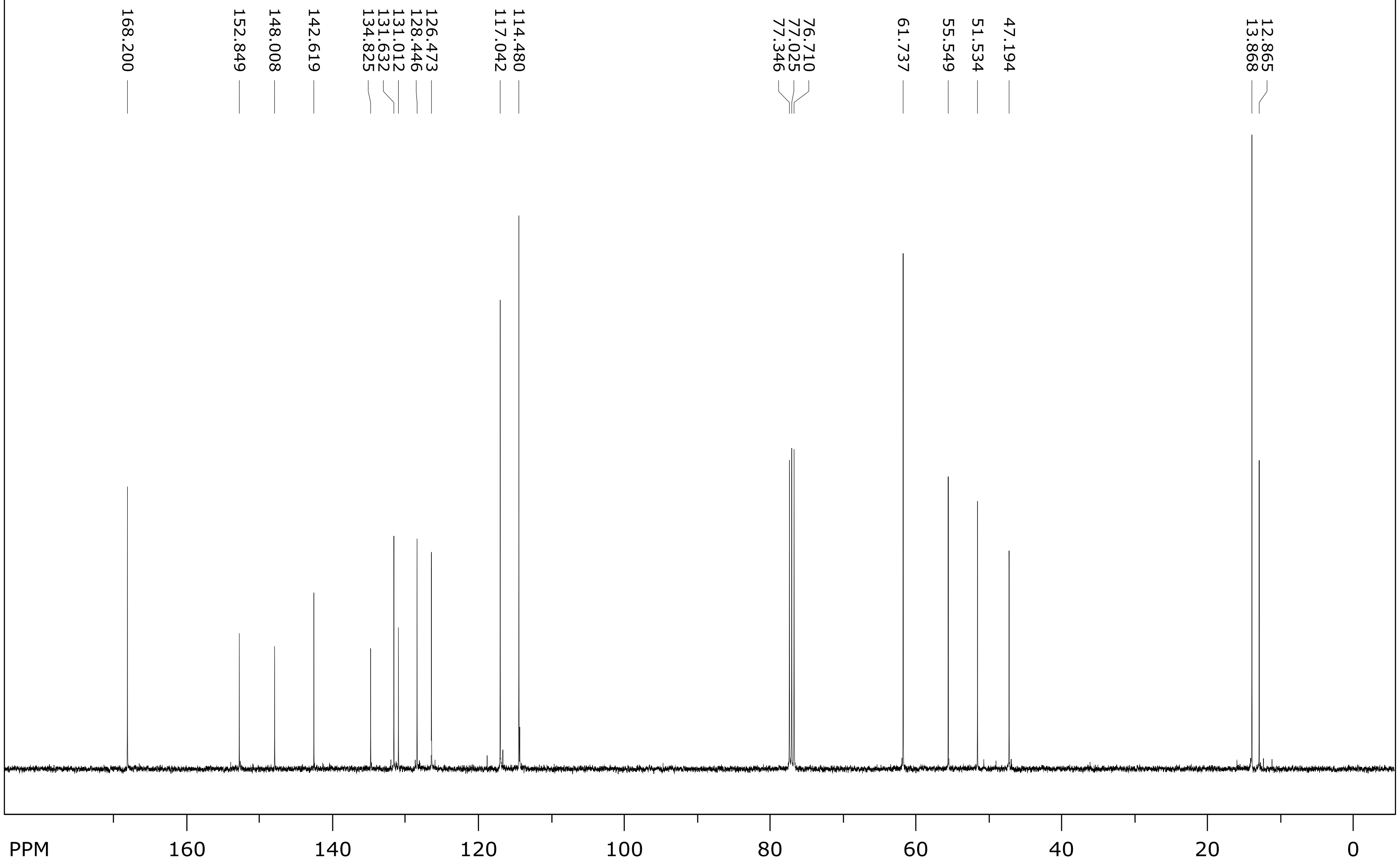

file: ...TEH 338\p6551teh_Carbon_ft-1-1.jdf   expt: undefined
transmitter freq.: 100.525303 MHz
time domain size: 65536 points
width: 31407.04 Hz = 312.4292 ppm = 0.479233 Hz/pt
number of scans: 256

freq. of 0 ppm: 100.515265 MHz
processed size: 32768 complex points
LB: 0.000   GF: 0.0000

teh338

York - Chemistry - Mass Spectrometry Service Report

Analysis Information

Acquisition Date 17/07/2015 11:25:30

Analysis Filename rjkt53628th_P1-E-8_01_59863.d
Method 600p_meoh1260_2c1s.m
Submission Name rjkt53628th
Instrument micrOTOF
ESI Positive

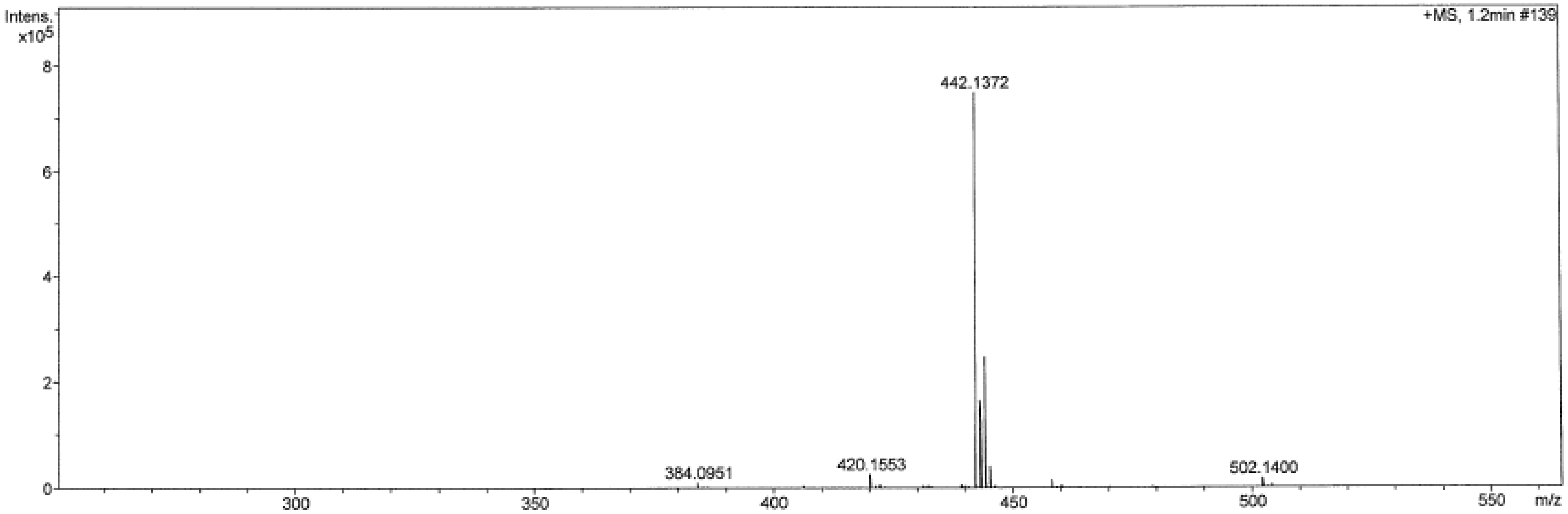

Meas. m/z	#	Formula	m/z	err [ppm]	err [mDa]	mSigma	Mean err [ppm]
420.1553	1	C 22 H 27 Cl N O 5	420.1572	4.5	1.9	47.5	3.2
442.1372	1	C 22 H 26 Cl N Na O 5	442.1392	4.5	2.0	20.3	4.7

PerkinElmer Spectrum Version 10.03.07
01 July 2015 17:31

Analyst Administrator
Date 01 July 2015 17:31

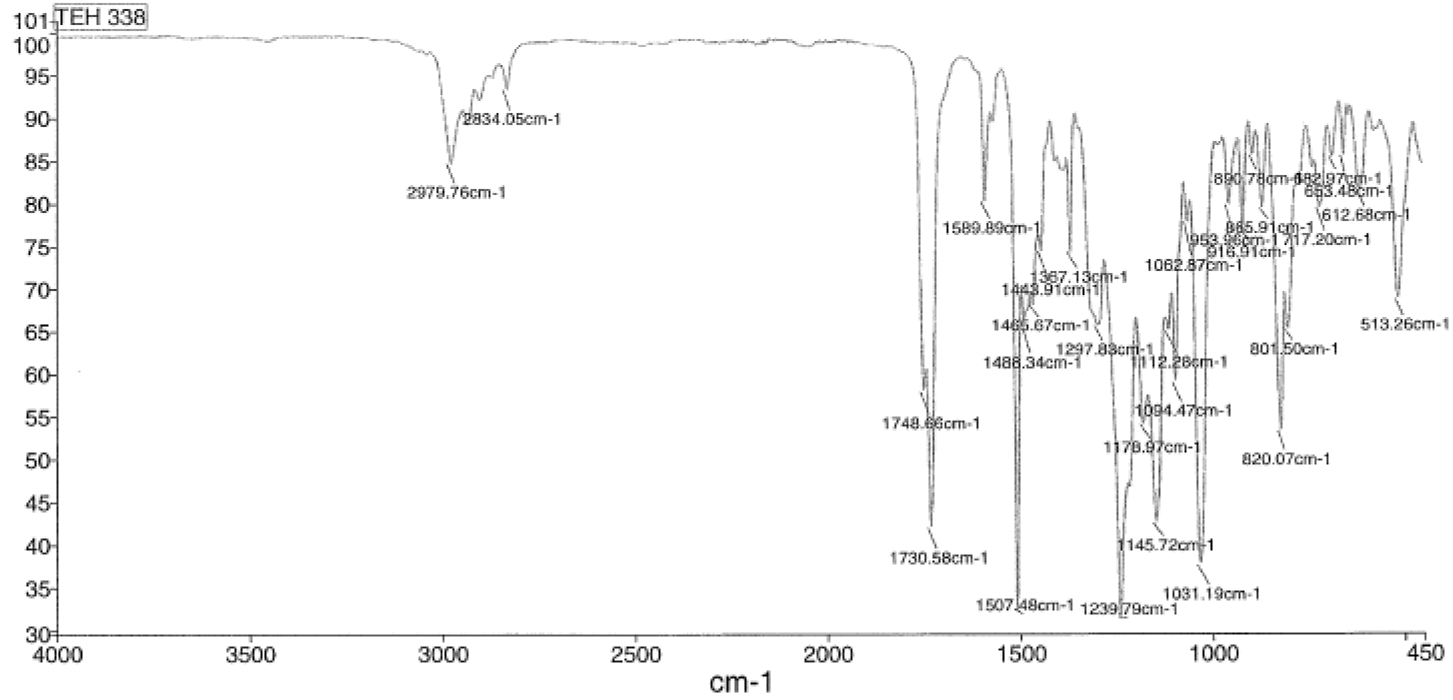

RJKT_01 07 2015_ 48 RJKT_01 07 2015_048

N-(4-*tert*-Butylphenyl)-2-iodopyridin-3-amine S4

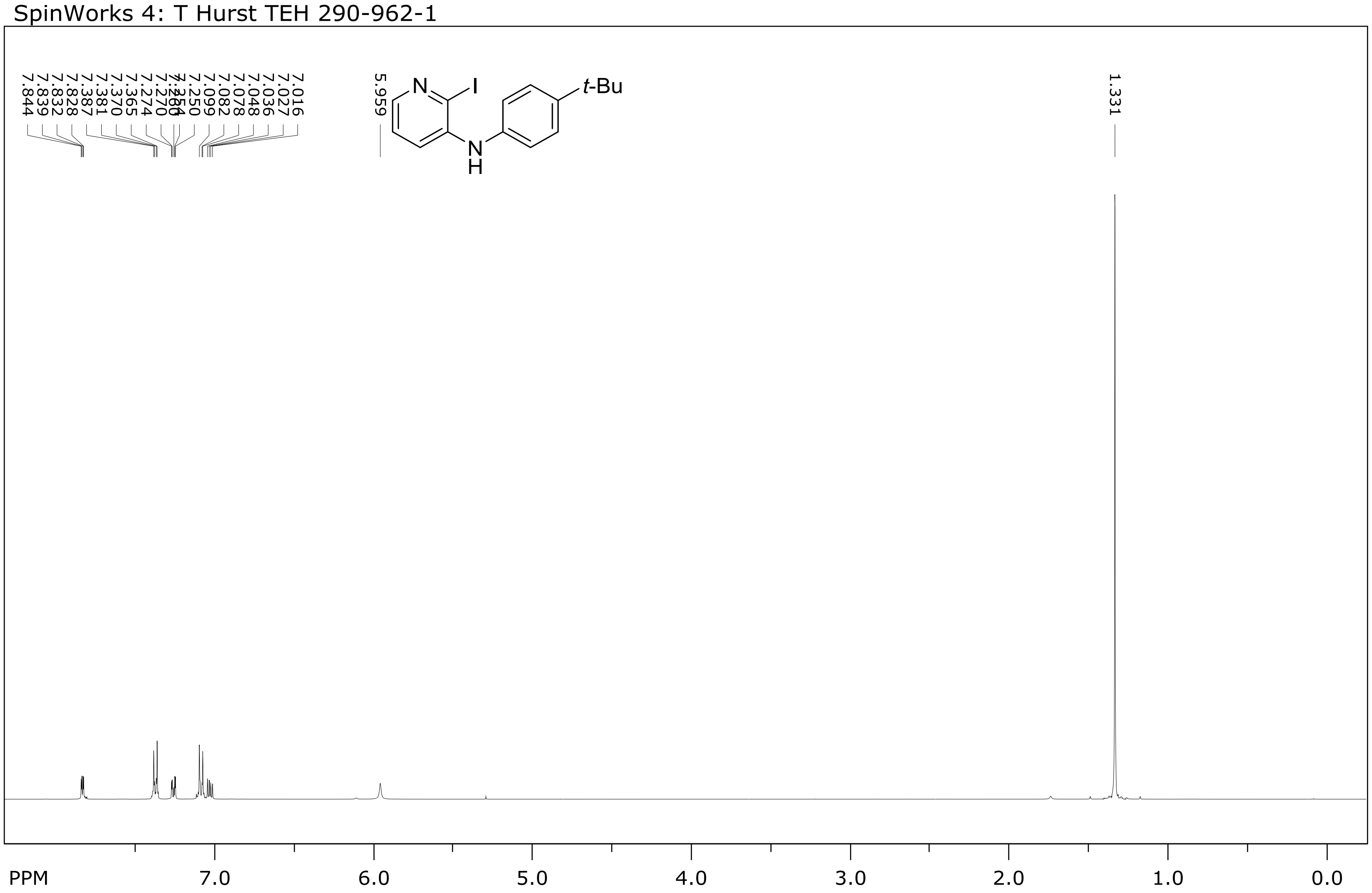

file: ...0-962-1\p6301teh_Proton_ft-1-1.jdf expt: undefined
transmitter freq.: 399.782198 MHz
time domain size: 32768 points
width: 7503.00 Hz = 18.7677 ppm = 0.228973 Hz/pt
number of scans: 8

freq. of 0 ppm: 399.780195 MHz
processed size: 16384 complex points
LB: 0.000 GF: 0.0000

SpinWorks 4: T Hurst TEH 290-962-1

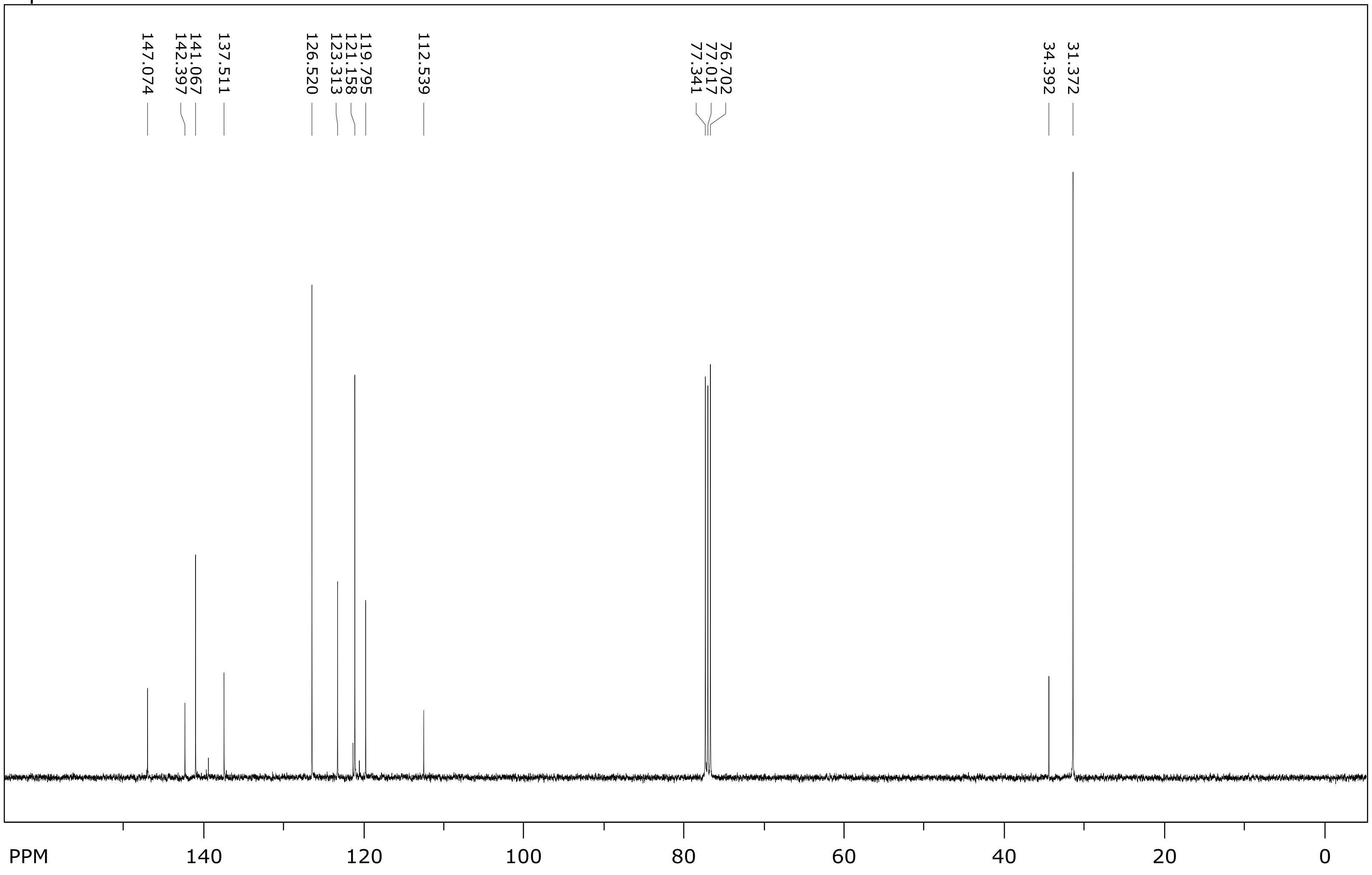

file: ...0-962-1\p6301teh_Carbon_ft-1-1.jdf expt: undefined
transmitter freq.: 100.525303 MHz
time domain size: 65536 points
width: 31407.04 Hz = 312.4292 ppm = 0.479233 Hz/pt
number of scans: 256

freq. of 0 ppm: 100.515262 MHz
processed size: 32768 complex points
LB: 0.000 GF: 0.0000

teh290

York - Chemistry - Mass Spectrometry Service Report

Analysis Information

Acquisition Date 14/10/2015 13:56:01

Analysis Filename	rjkt55050th_P1-E-5_01_61511.d
Method	400p_meoh1260_2c1s.m
Submission Name	rjkt55050th
Instrument	micrOTOF
ESI	Positive

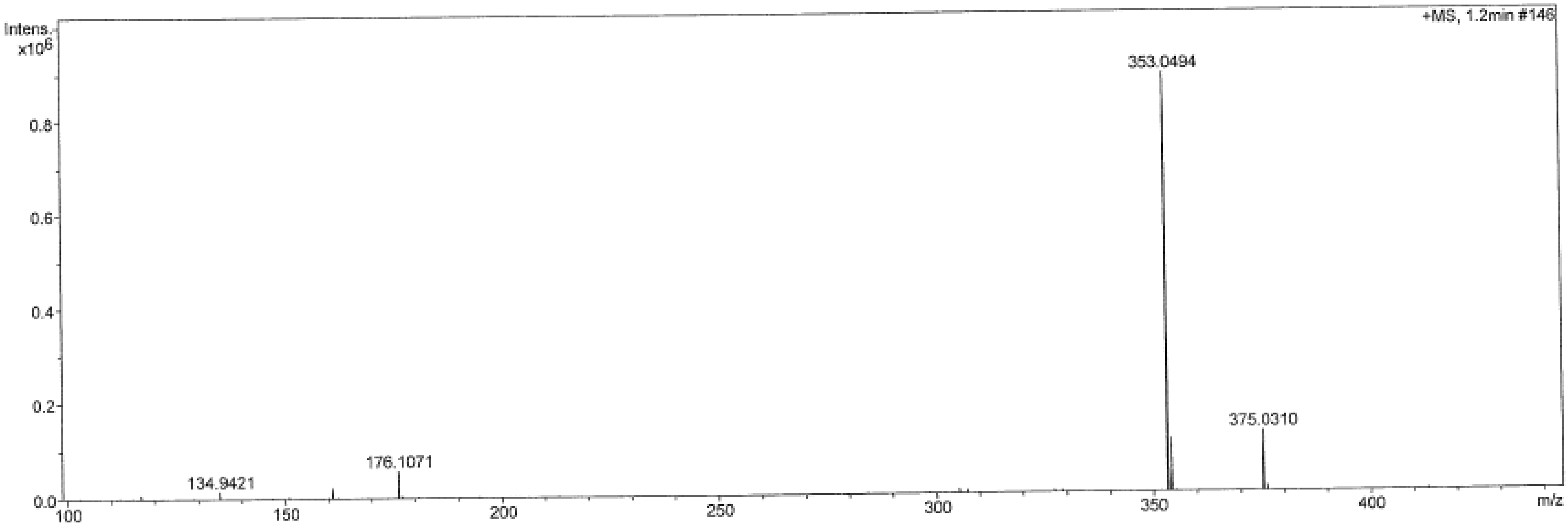

Meas. m/z	#	Formula	m/z	err [ppm]	err [mDa]	mSigma	Mean err [ppm]
353.0494	1	C 15 H 18 I N 2	353.0509	4.2	1.5	26.3	4.2
375.0310	1	C 15 H 17 I N 2 Na	375.0329	5.0	1.9	50.7	5.0

PerkinElmer Spectrum Version 10.03.07
14 October 2015 21:11

Analyst PEService
Date 14 October 2015 21:11

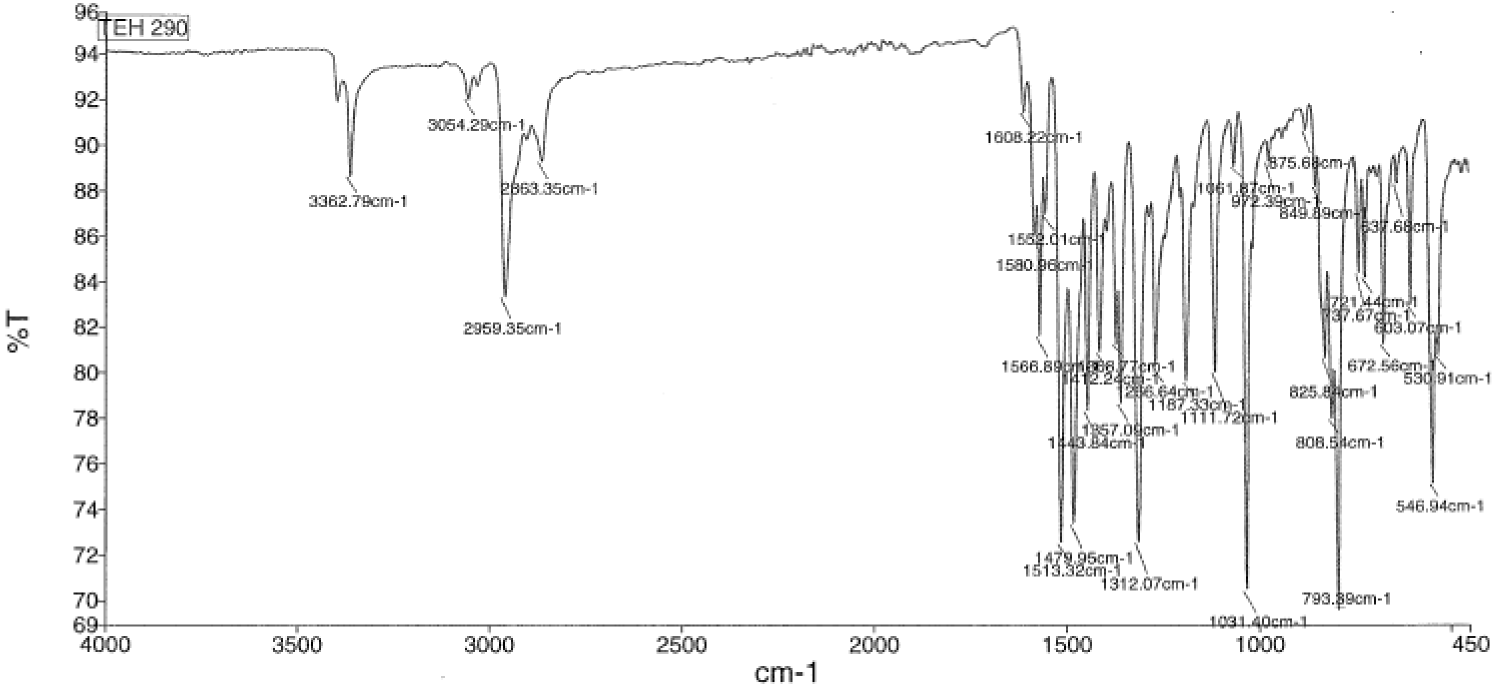

PEService 28 Sample 028 By PEService Date Wednesday, October 14 2015

_N_-(4-_tert_-Butylphenyl)-2-iodo-_N_-methylpyridin-3-amine S5

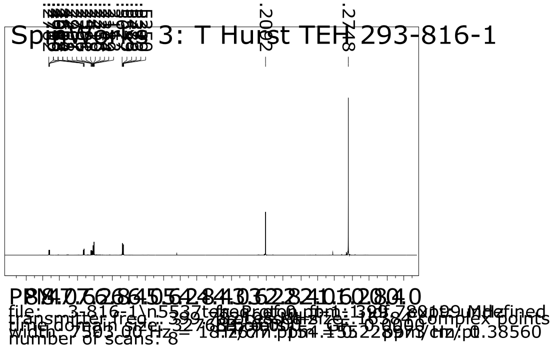

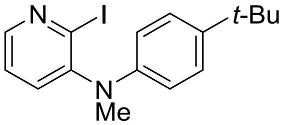

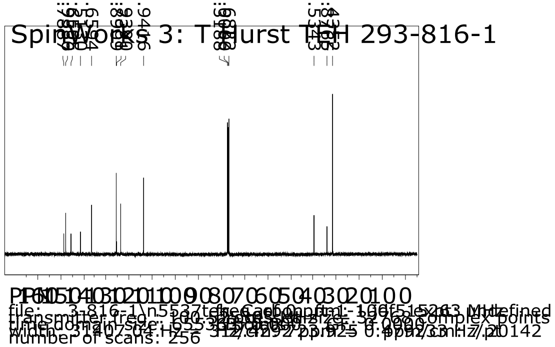
number of scans: 256

teh293

York - Chemistry - Mass Spectrometry Service Report

Analysis Information

Acquisition Date 14/10/2015 14:04:35

Analysis Filename	rjkt55051th_P1-E-6_01_61513.d
Method	400p_meoh1260_2c1s.m
Submission Name	rjkt55051th
Instrument	micrOTOF
ESI	Positive

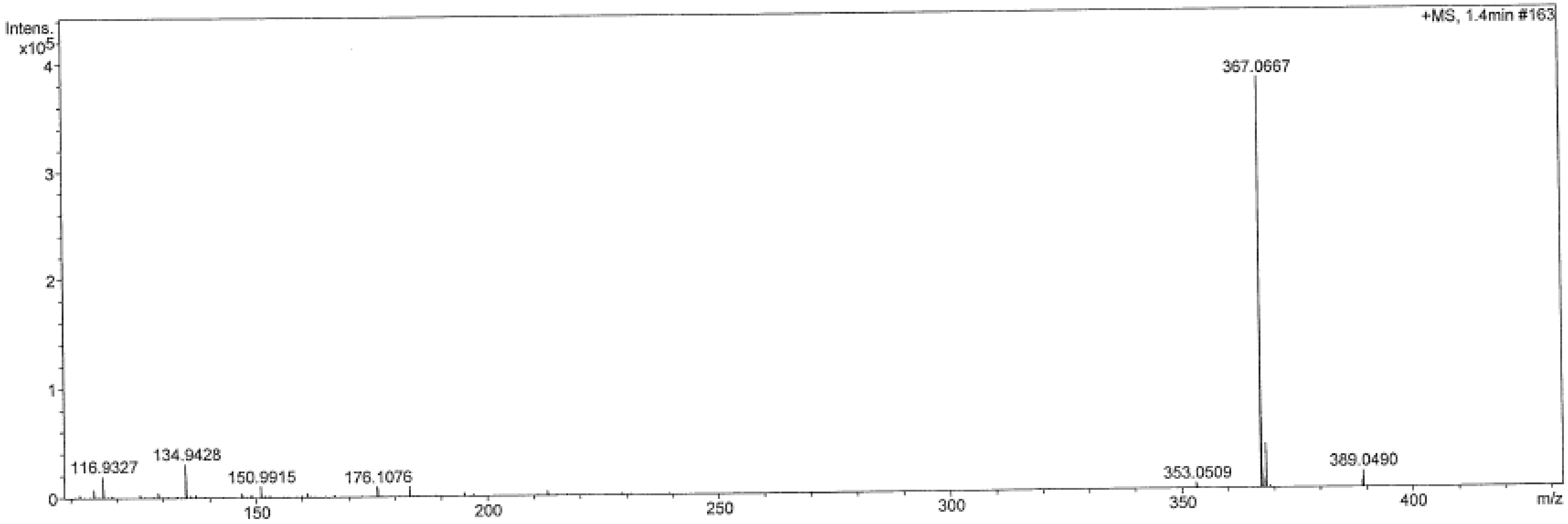

Meas. m/z	#	Formula	m/z	err [ppm]	err [mDa]	mSigma	Mean err [ppm]
367.0667	1	C 16 H 20 I N 2	367.0666	-0.2	-0.1	43.6	-0.1
389.0490	1	C 16 H 19 I N 2 Na	389.0485	-1.3	-0.5	47.1	-2.2

PerkinElmer Spectrum Version 10.03.07
27 October 2015 13:30

Analyst PEService
Date 27 October 2015 13:30

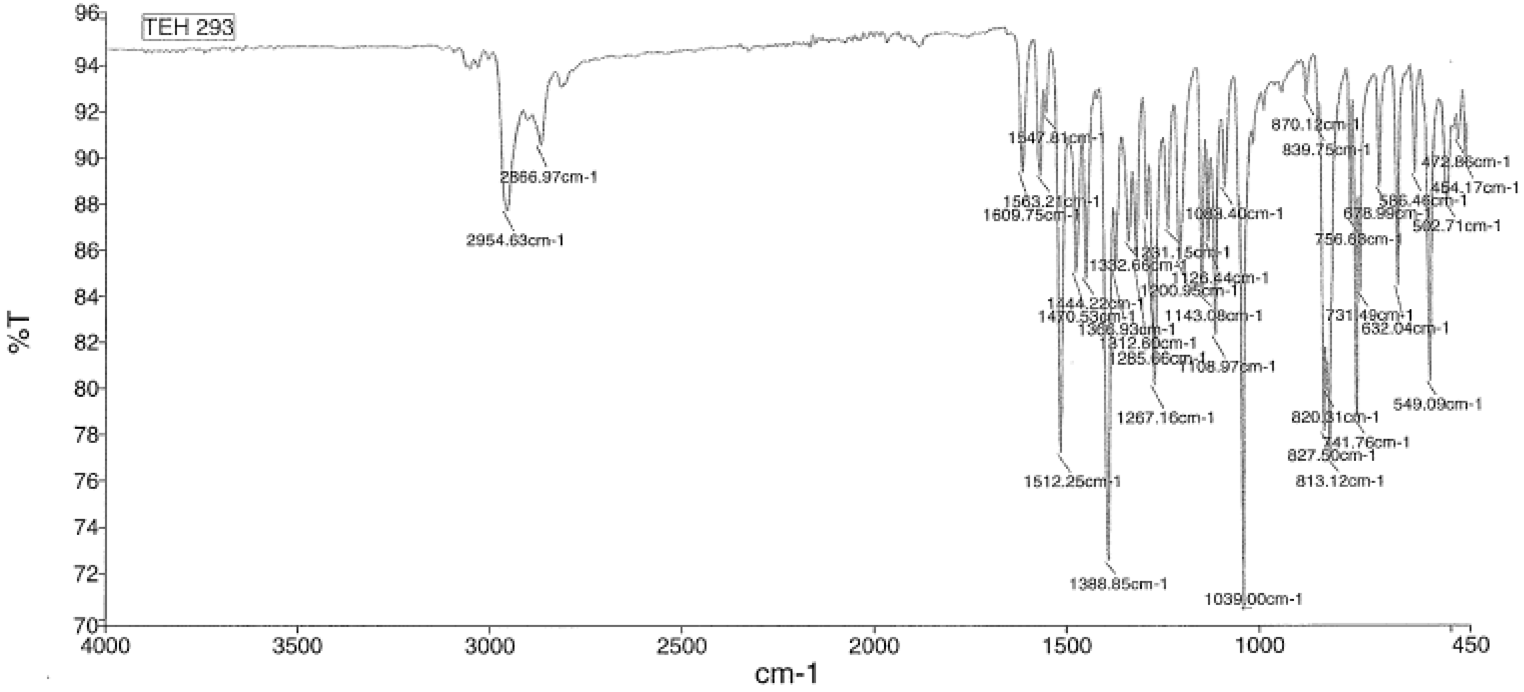

PEService 139 Sample 139 By PEService Date Tuesday, October 27 2015

1,3-Diethyl 2-{3-[(4-*tert*-butylphenyl)(methyl)amino]pyridine-2-yl}propanedioate 15k

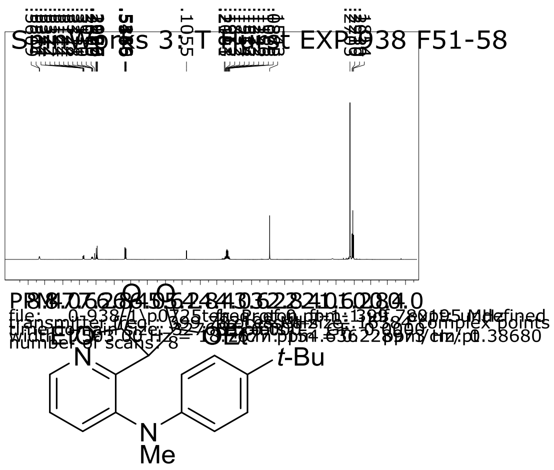

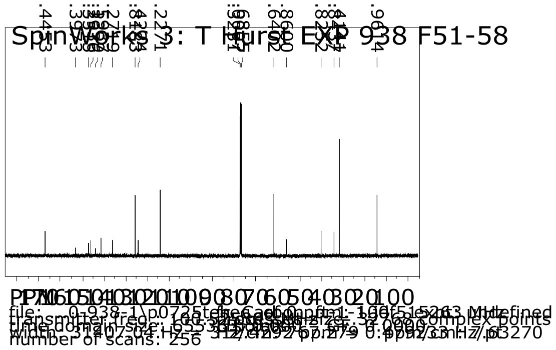
SpinWorks 3: T Hurst EXP 938 F51-58
number of scans: 256

teh350

York - Chemistry - Mass Spectrometry Service Report

Analysis Information

Acquisition Date 14/10/2015 14:07:31

Analysis Filename	rjkt55052th_P1-E-7_01_61514.d
Method	600p_meoh1260_2c1s.m
Submission Name	rjkt55052th
Instrument	micrOTOF
ESI	Positive

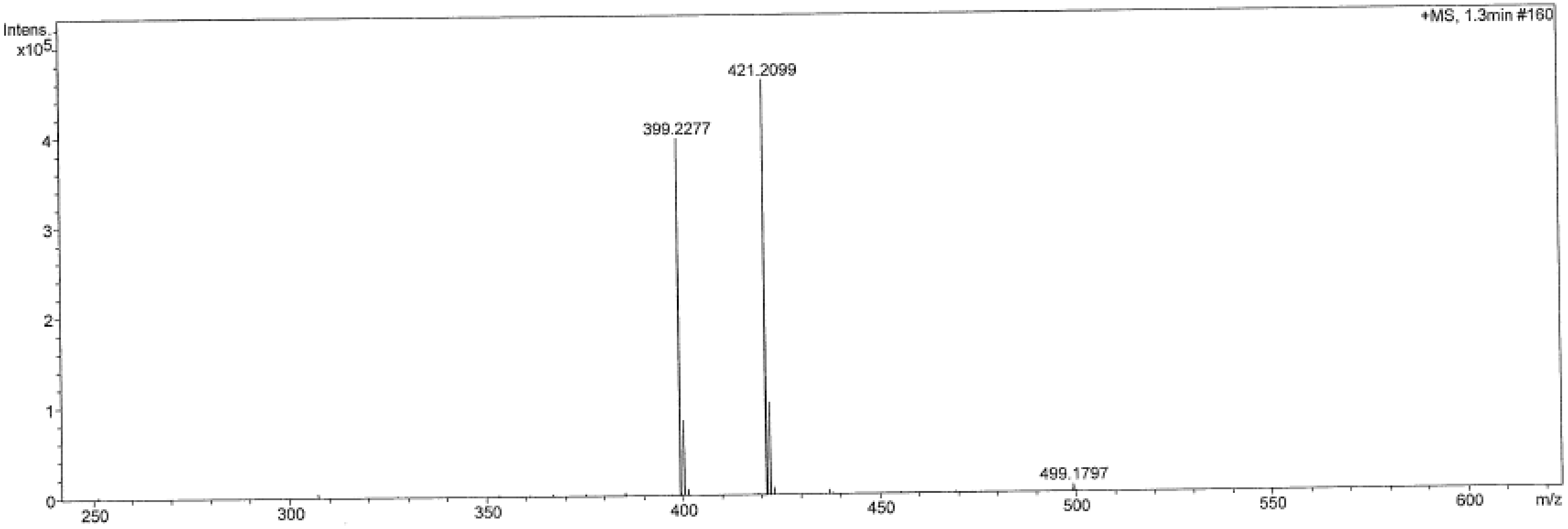

Meas. m/z	#	Formula	m/z	err [ppm]	err [mDa]	mSigma	Mean err [ppm]
399.2277	1	C 23 H 31 N 2 O 4	399.2278	0.3	0.1	31.8	0.9
421.2099	1	C 23 H 30 N 2 Na O 4	421.2098	-0.2	-0.1	23.3	0.3

PerkinElmer Spectrum Version 10.03.07
14 October 2015 21:17

Analyst PEService
Date 14 October 2015 21:17

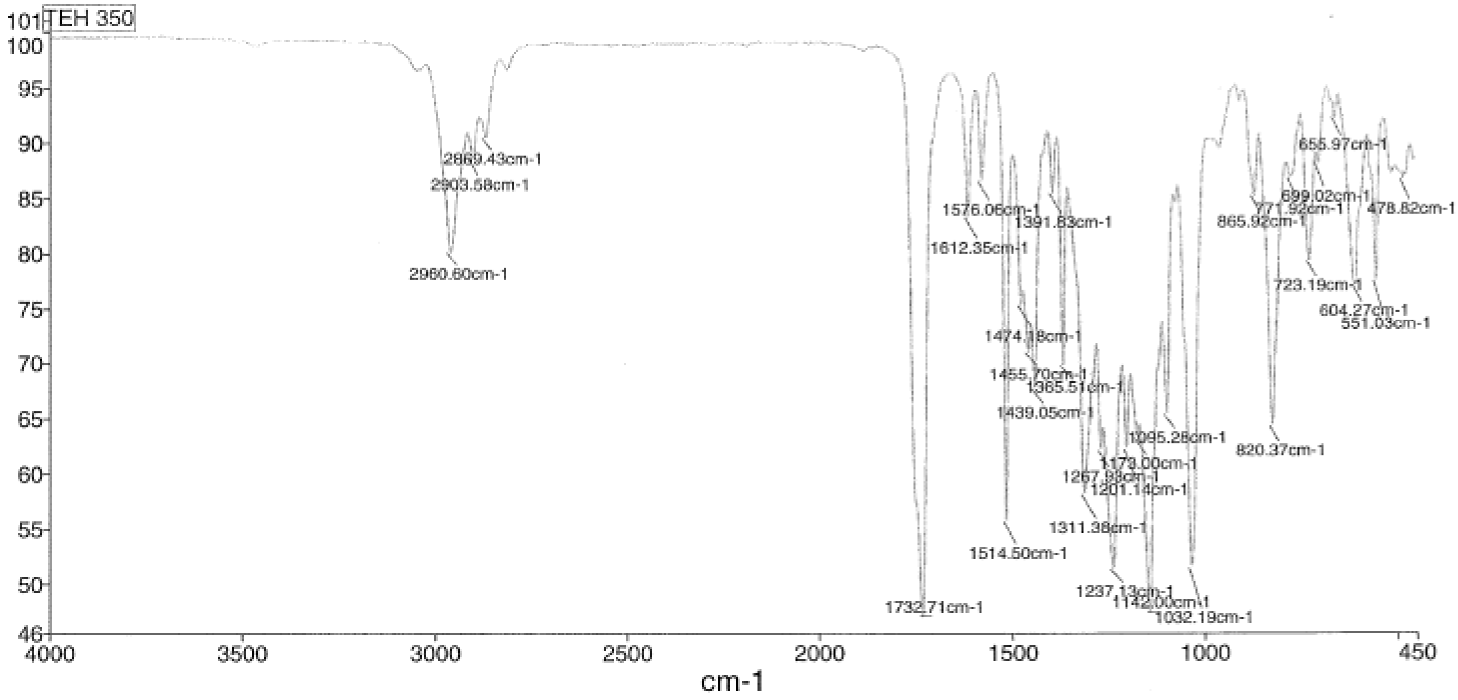

PEService 31 Sample 031 By PEService Date Wednesday, October 14 2015

1,3-diethyl 2-(2-phenoxyphenyl)propanedioate 15l

SpinWorks 4: T Hurst TEH 326A-900-1

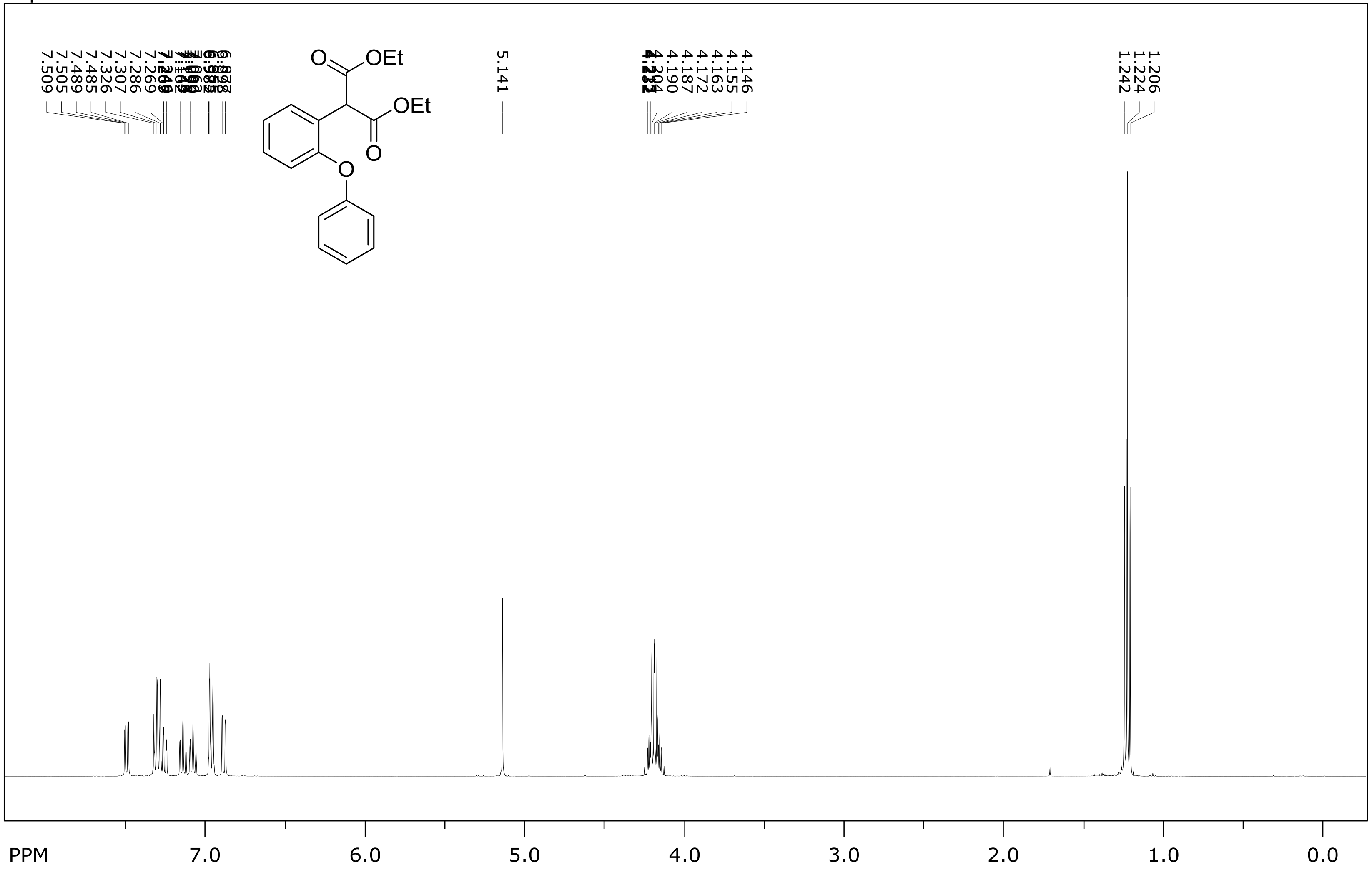

file: ...A-900-1\n9402teh_Proton_ft-1-1.jdf expt: undefined
transmitter freq.: 399.782198 MHz
time domain size: 32768 points
width: 7503.00 Hz = 18.7677 ppm = 0.228973 Hz/pt
number of scans: 8

freq. of 0 ppm: 399.780199 MHz
processed size: 16384 complex points
LB: 0.000 GF: 0.0000

SpinWorks 4: T Hurst TEH 326A-900-1

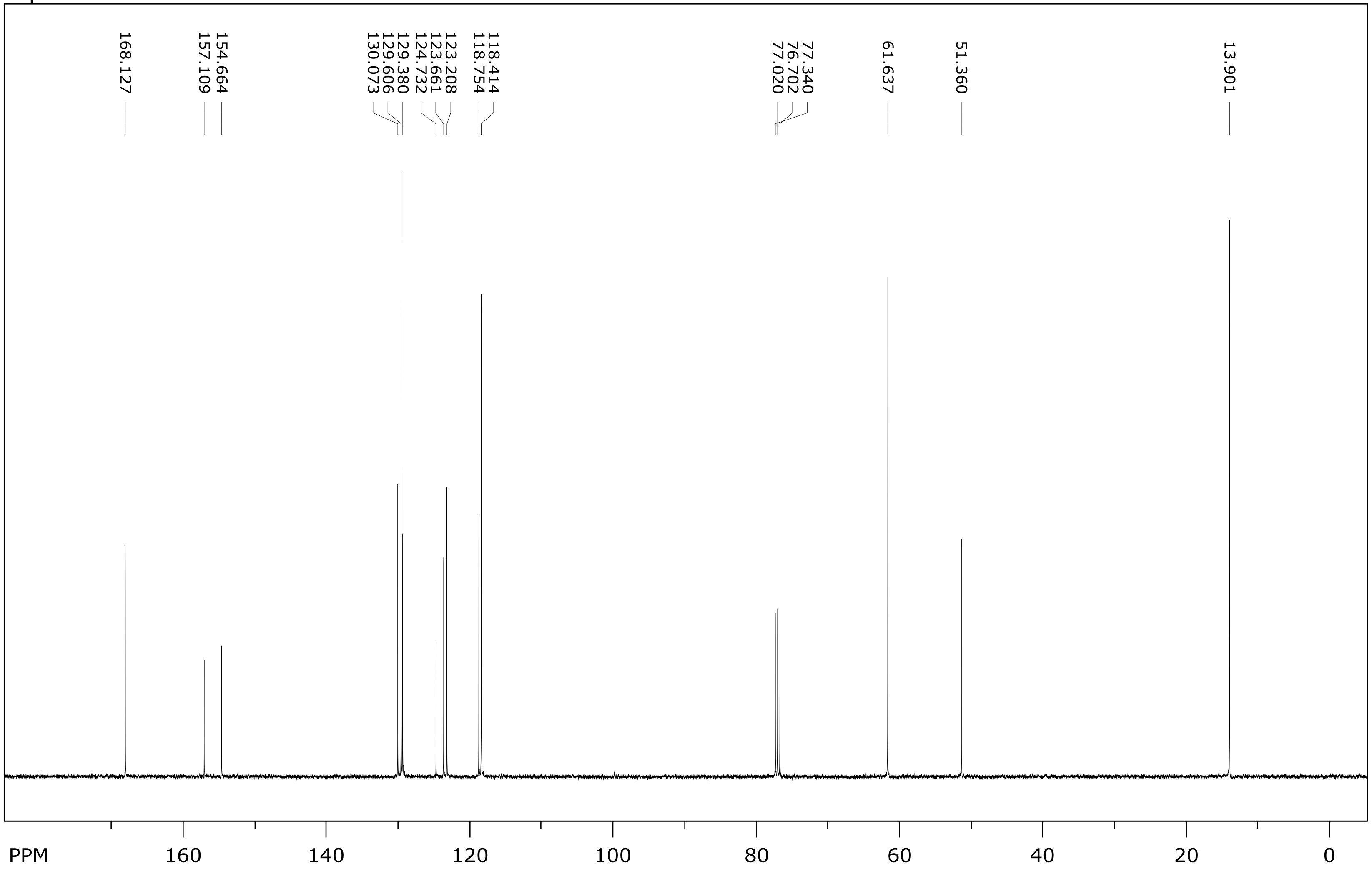

file: ...A-900-1\n9402teh_Carbon_ft-1-1.jdf expt: undefined
transmitter freq.: 100.525303 MHz
time domain size: 65536 points
width: 31407.04 Hz = 312.4292 ppm = 0.479233 Hz/pt
number of scans: 256

freq. of 0 ppm: 100.515270 MHz
processed size: 32768 complex points
LB: 0.000 GF: 0.0000

teh326a

York - Chemistry - Mass Spectrometry Service Report

Analysis Information

Acquisition Date 10/08/2015 16:24:26

Analysis Filename	rjkt54044th_P1-B-1_01_60277.d
Method	400p_meoh1260_2c1s.m
Submission Name	rjkt54044th
Instrument	micrOTOF
ESI	Positive

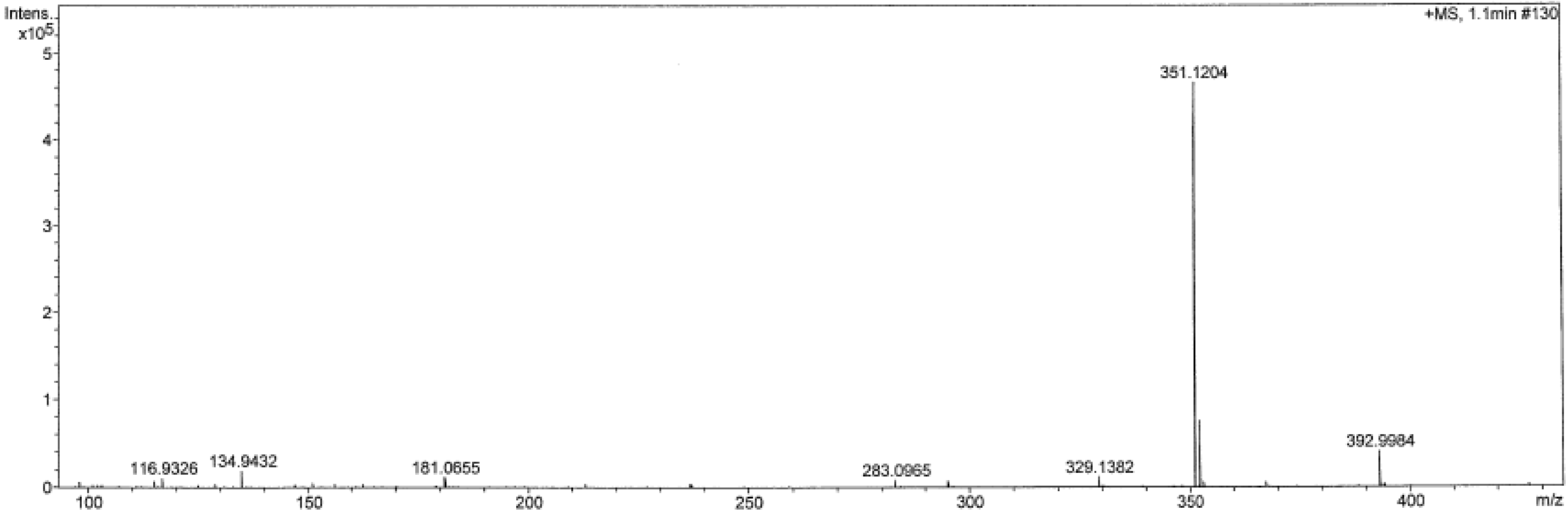

Meas. m/z	#	Formula	m/z	err [ppm]	err [mDa]	mSigma	Mean err [ppm]
329.1382	1	C 19 H 21 O 5	329.1384	0.4	0.1	27.8	-1.3
351.1204	1	C 19 H 20 Na O 5	351.1203	-0.3	-0.1	25.8	-0.5

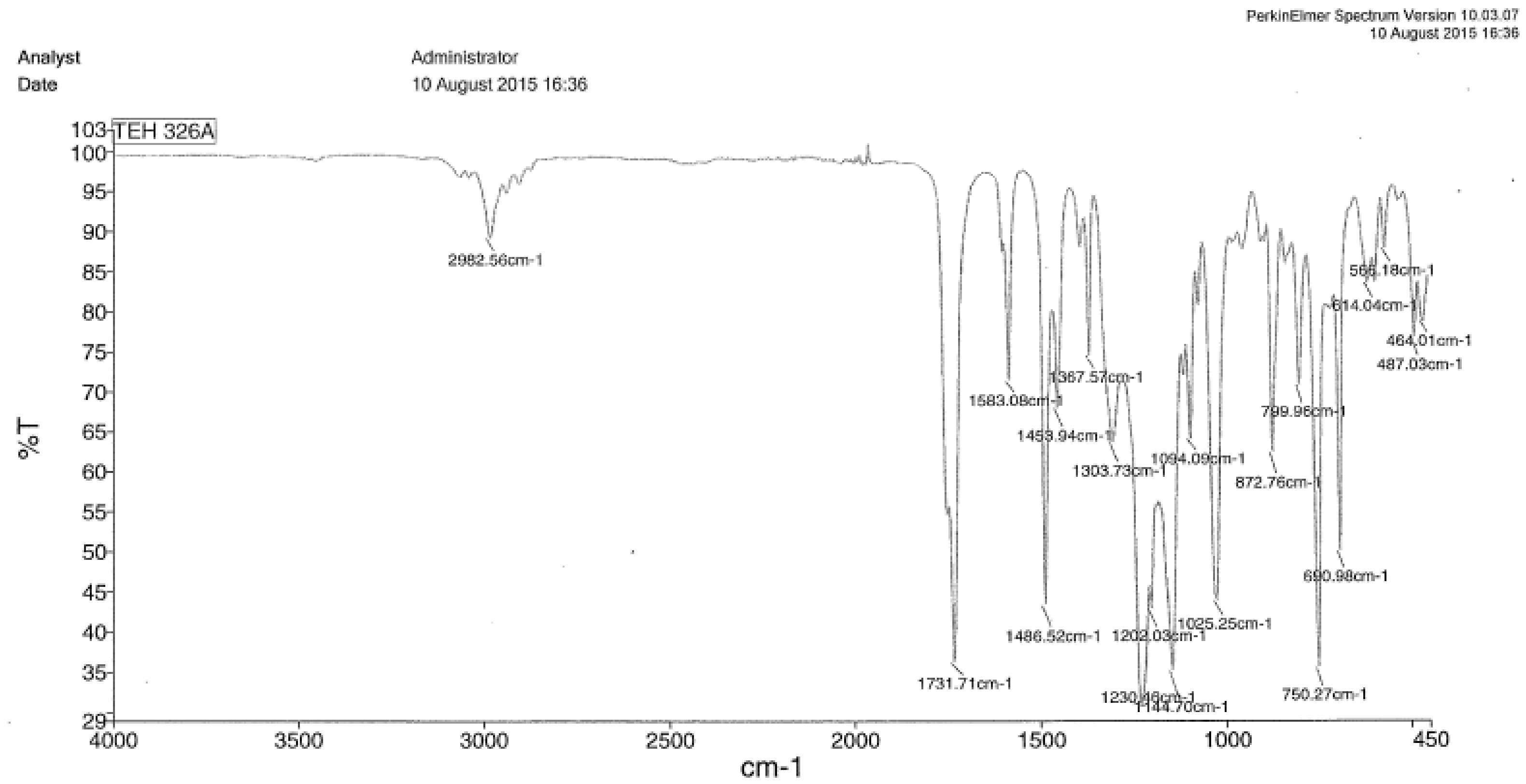
PerkinElmer Spectrum Version 10.03.07
10 August 2015 16:36
Analyst
Administrator
Date
10 August 2015 16:36
TEH 326A
%T
cm-1
2982.56cm-1
1731.71cm-1
1583.08cm-1
1486.52cm-1
1453.94cm-1
1367.57cm-1
1303.73cm-1
1230.46cm-1
1202.03cm-1
1144.70cm-1
1094.09cm-1
1025.25cm-1
872.76cm-1
799.96cm-1
750.27cm-1
690.98cm-1
614.04cm-1
566.18cm-1
487.03cm-1
464.01cm-1

1,3-diethyl 2-[2-(phenylsulfanyl)phenyl]propanedioate 15m

SpinWorks 4: T Hurst TEH 344

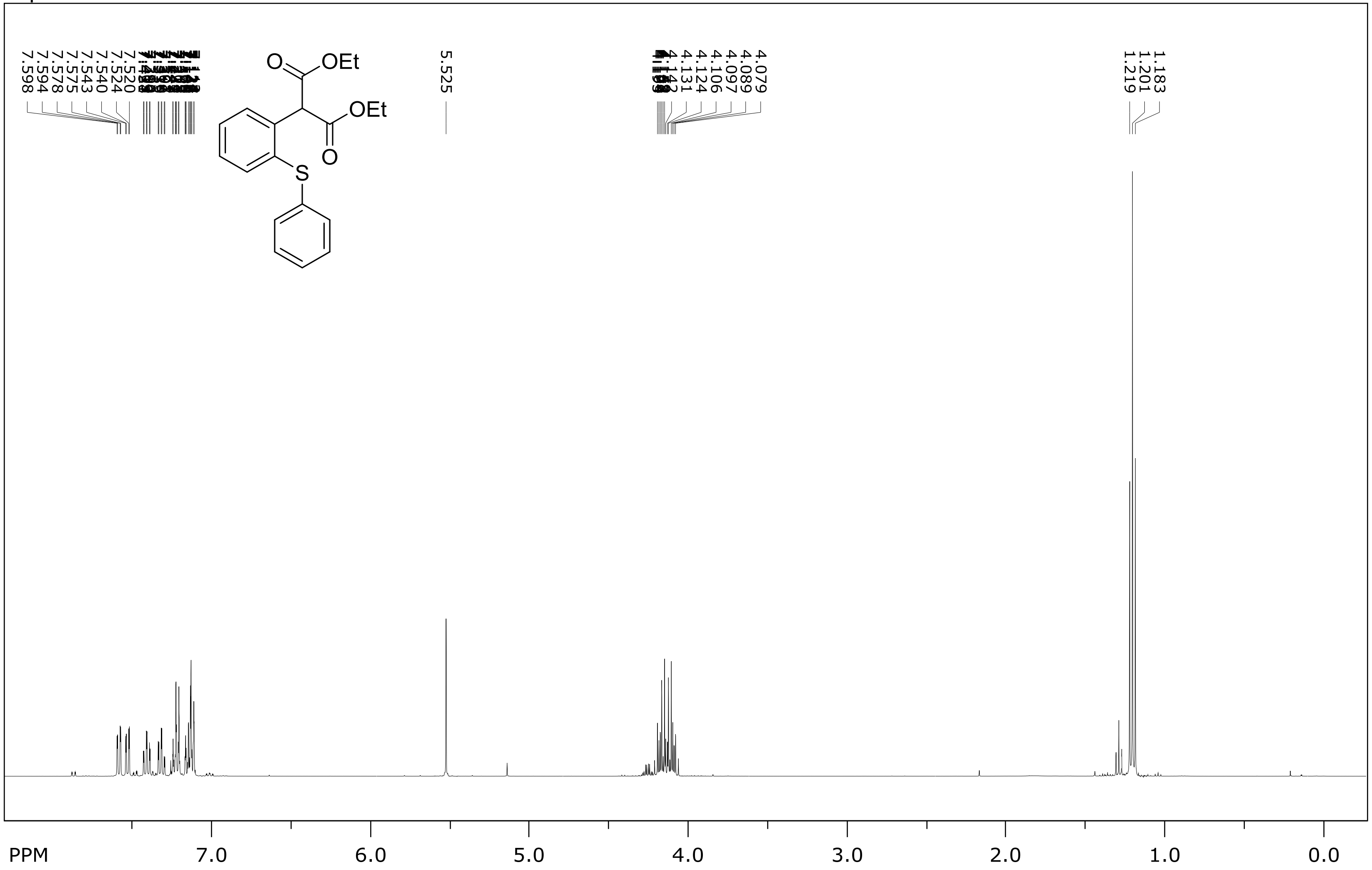

file: ...TEH 344\p6552teh_Proton_ft-1-1.jdf expt: undefined
transmitter freq.: 399.782198 MHz
time domain size: 32768 points
width: 7503.00 Hz = 18.7677 ppm = 0.228973 Hz/pt
number of scans: 8

freq. of 0 ppm: 399.780194 MHz
processed size: 16384 complex points
LB: 0.000 GF: 0.0000

SpinWorks 4: T Hurst TEH 344

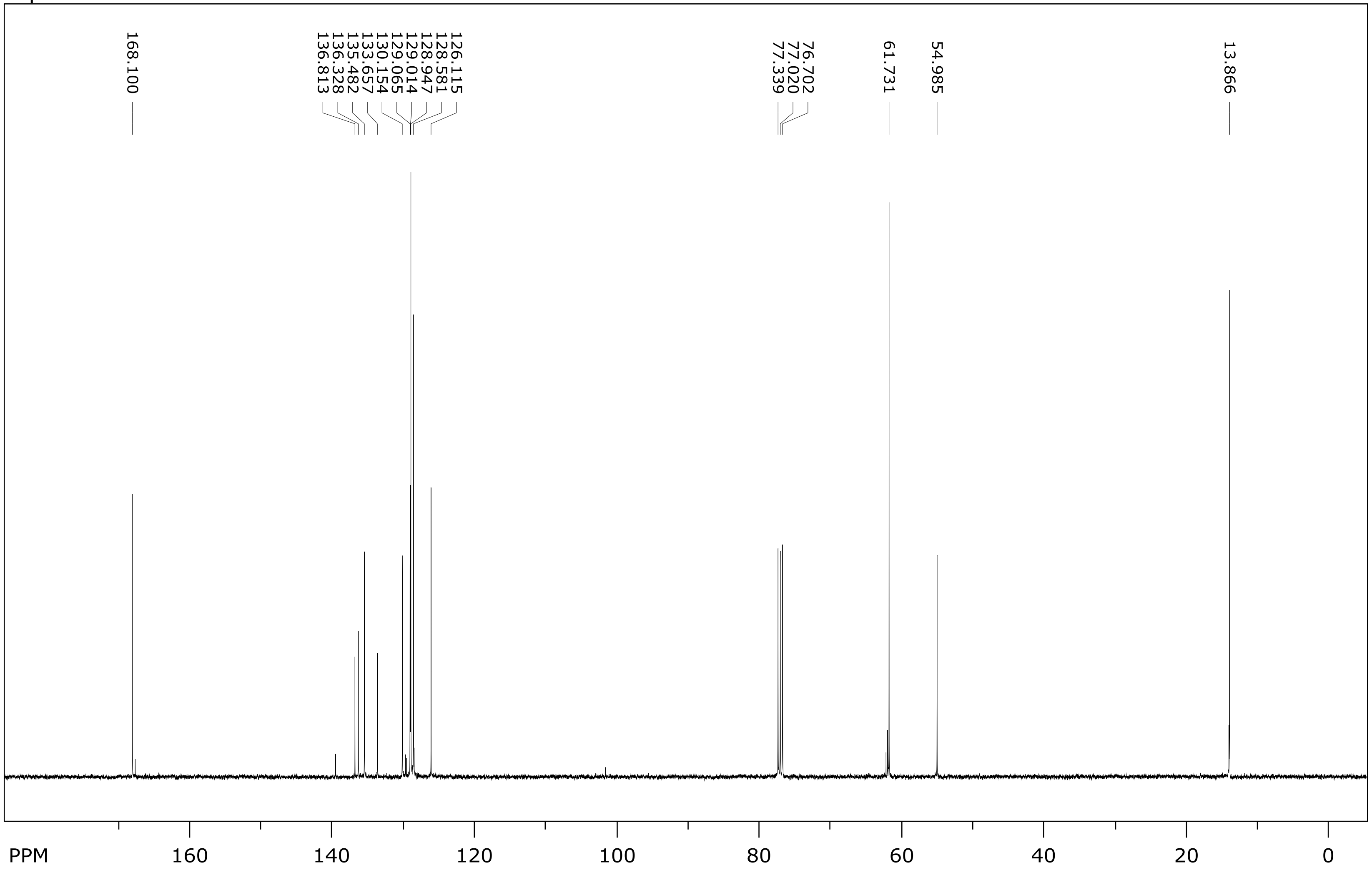

file: ...TEH 344\p6552teh_Carbon_ft-1-1.jdf expt: undefined
transmitter freq.: 100.525303 MHz
time domain size: 65536 points
width: 31407.04 Hz = 312.4292 ppm = 0.479233 Hz/pt
number of scans: 256

freq. of 0 ppm: 100.515268 MHz
processed size: 32768 complex points
LB: 0.000 GF: 0.0000

teh344

York - Chemistry - Mass Spectrometry Service Report

Analysis Information

Acquisition Date 10/08/2015 16:27:22

Analysis Filename	rjkt54045th_P1-B-2_01_60278.d
Method	400p_meoh1260_2c1s.m
Submission Name	rjkt54045th
Instrument	micrOTOF
ESI	Positive

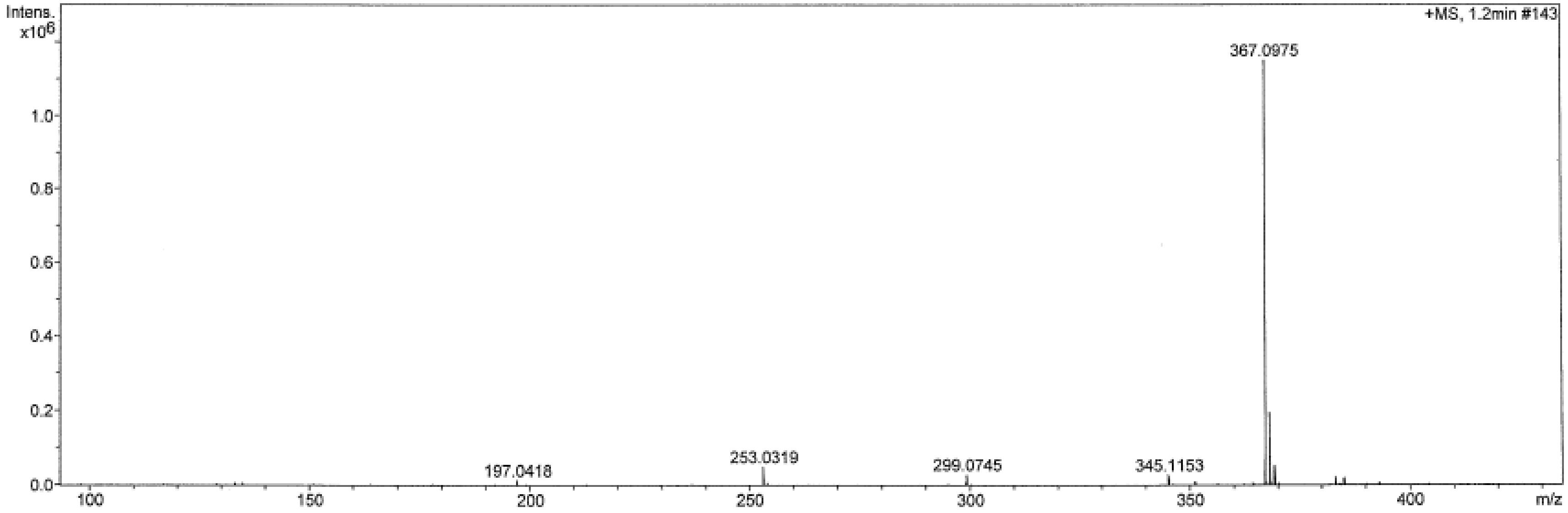

Meas. m/z	#	Formula	m/z	err [ppm]	err [mDa]	mSigma	Mean err [ppm]
345.1153	1	C 19 H 21 O 4 S	345.1155	0.6	0.2	38.3	-0.3
367.0975	1	C 19 H 20 Na O 4 S	367.0975	-0.1	-0.0	26.2	-0.2

PerkinElmer Spectrum Version 10.03.07
10 August 2015 16:39

Analyst Administrator

Date 10 August 2015 16:39

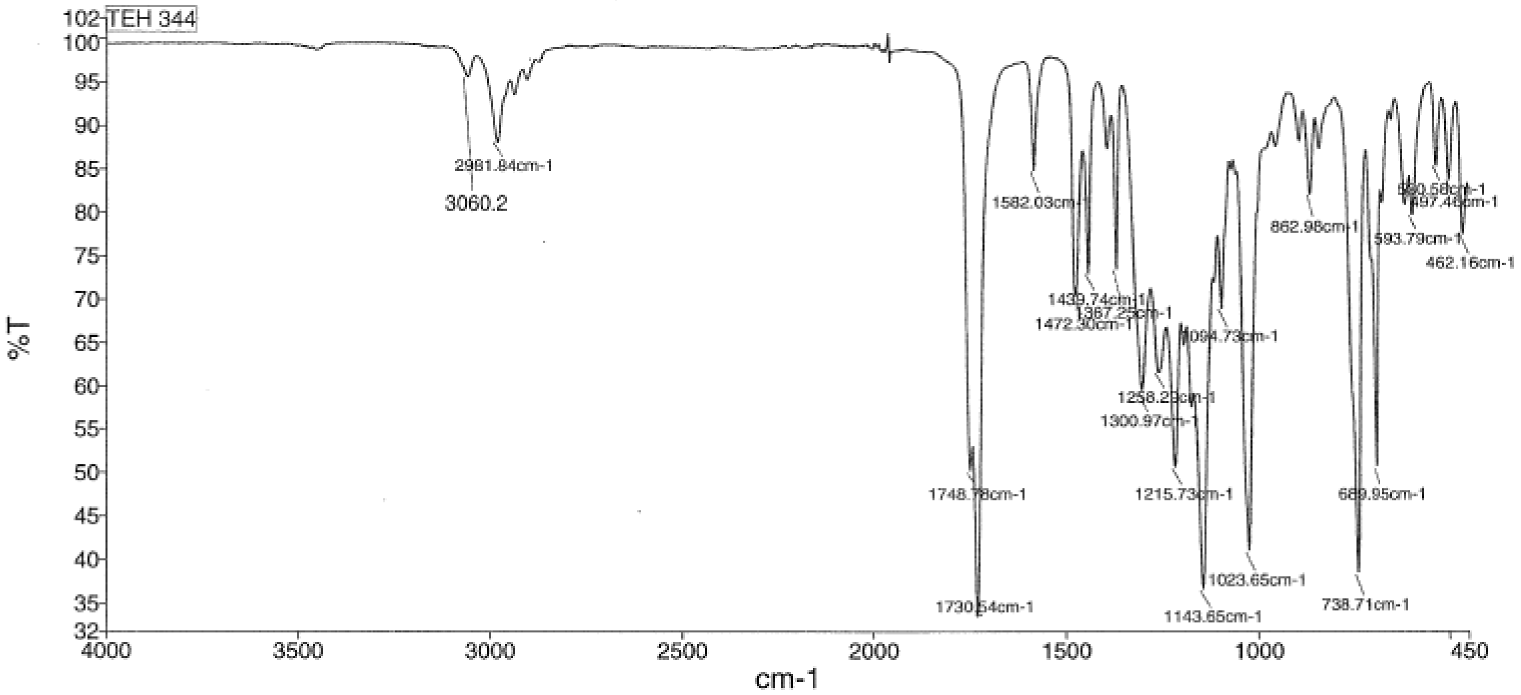

9,9-Diethyl 10-ethyl-9,10-dihydroacridine-9,9-dicarboxylate 16a

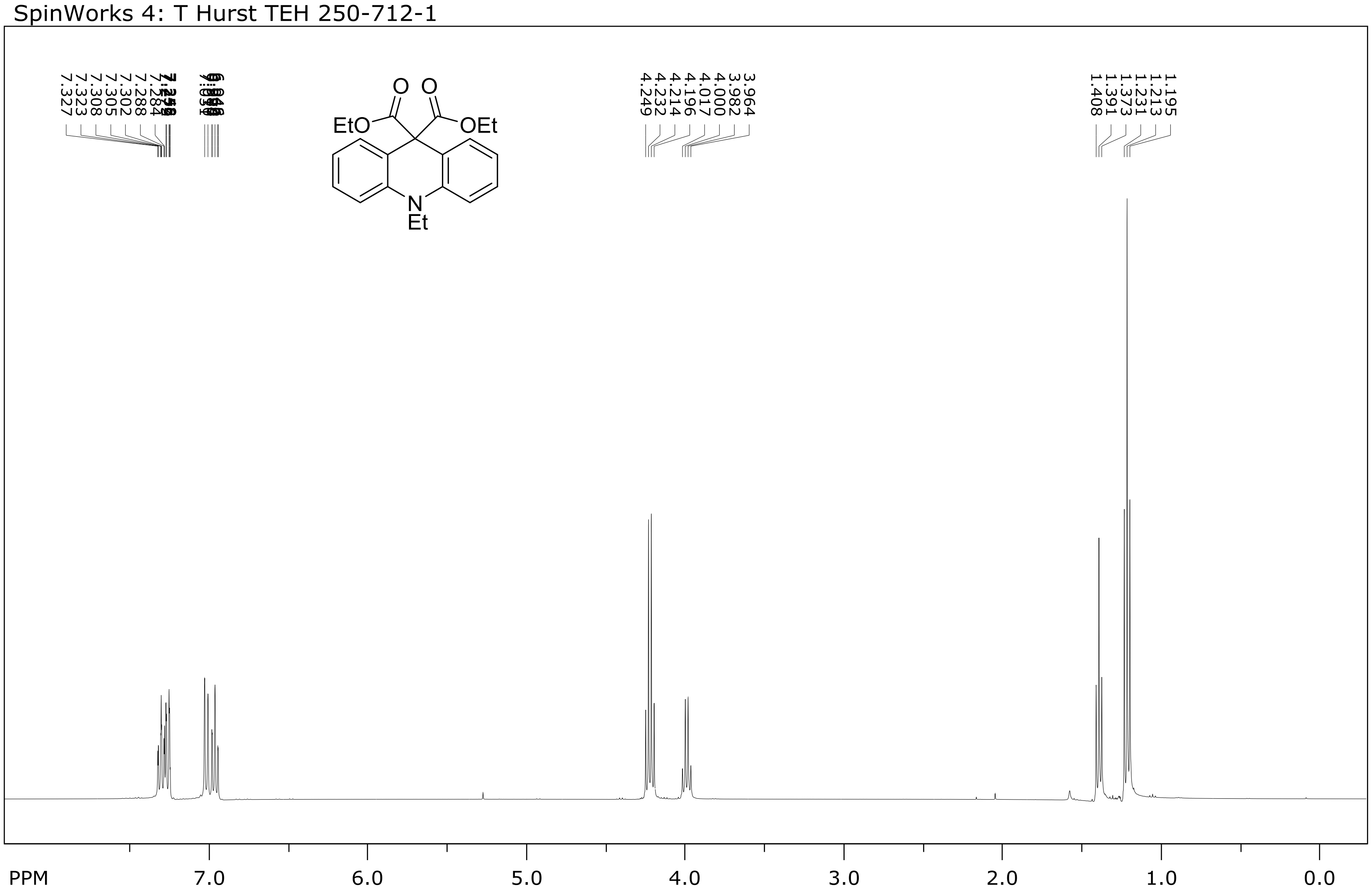

file: ...EH 250-712-1\c8015teh_PROTON-1.jdf expt: single_pulse.ex2
transmitter freq.: 399.782198 MHz
time domain size: 32768 points
width: 7503.00 Hz = 18.7677 ppm = 0.228973 Hz/pt
number of scans: 16

freq. of 0 ppm: 399.780199 MHz
processed size: 32768 complex points
LB: 0.458 GF: 0.0000

SpinWorks 4: T Hurst TEH 250-712-1

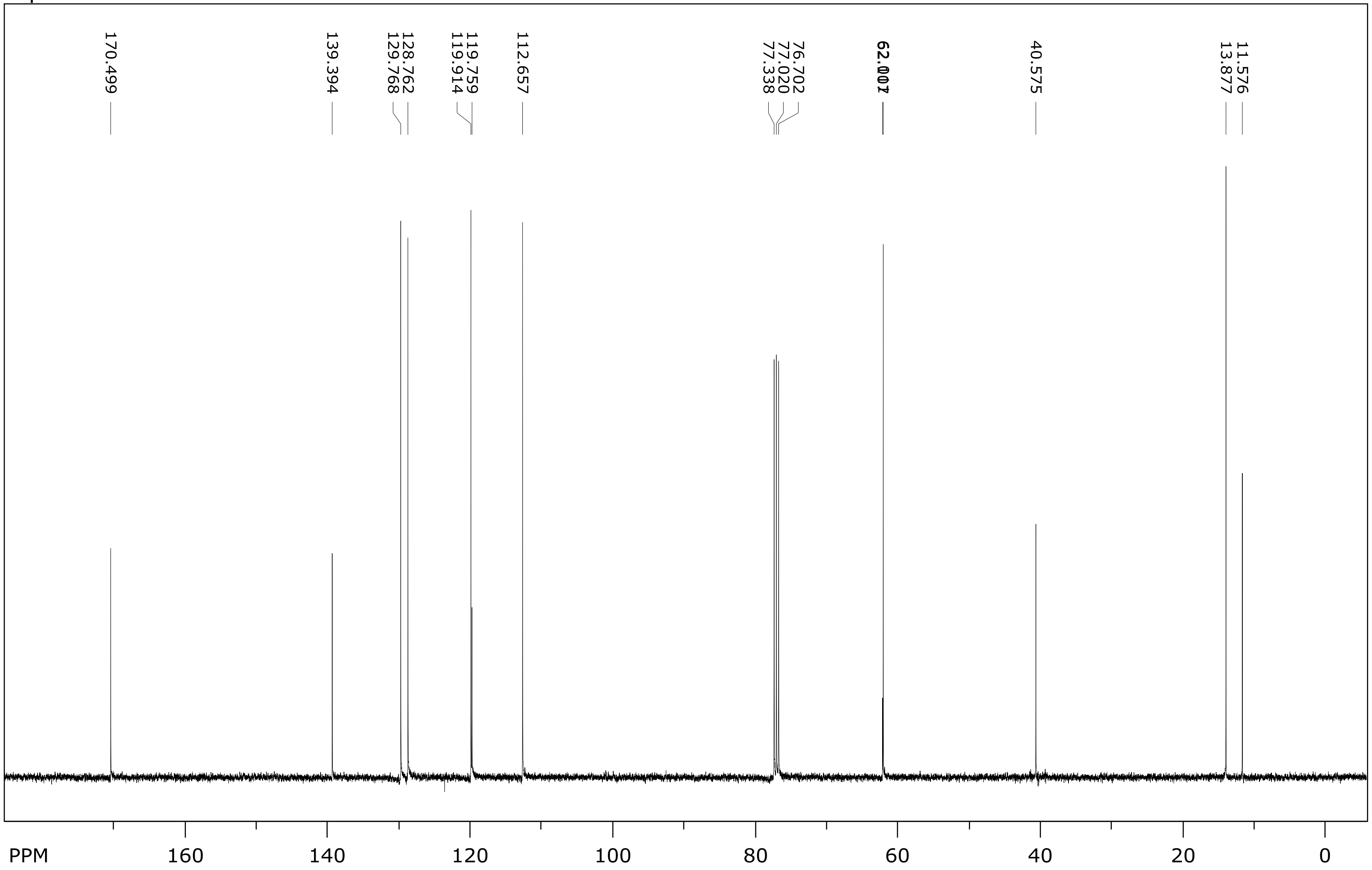

file: ...EH 250-712-1\c8016teh_CARBON-1.jdf expt: single_pulse_dec
transmitter freq.: 100.525303 MHz
time domain size: 65536 points
width: 31407.04 Hz = 312.4292 ppm = 0.479233 Hz/pt
number of scans: 256

freq. of 0 ppm: 100.515264 MHz
processed size: 65536 complex points
LB: 0.958 GF: 0.0000

teh250

York - Chemistry - Mass Spectrometry Service Report

Analysis Information

Acquisition Date 17/07/2015 11:28:25

Analysis Filename	rjkt53629th_P1-E-9_01_59864.d
Method	400p_meoh1260_2c1s.m
Submission Name	rjkt53629th
Instrument	micrOTOF
ESI	Positive

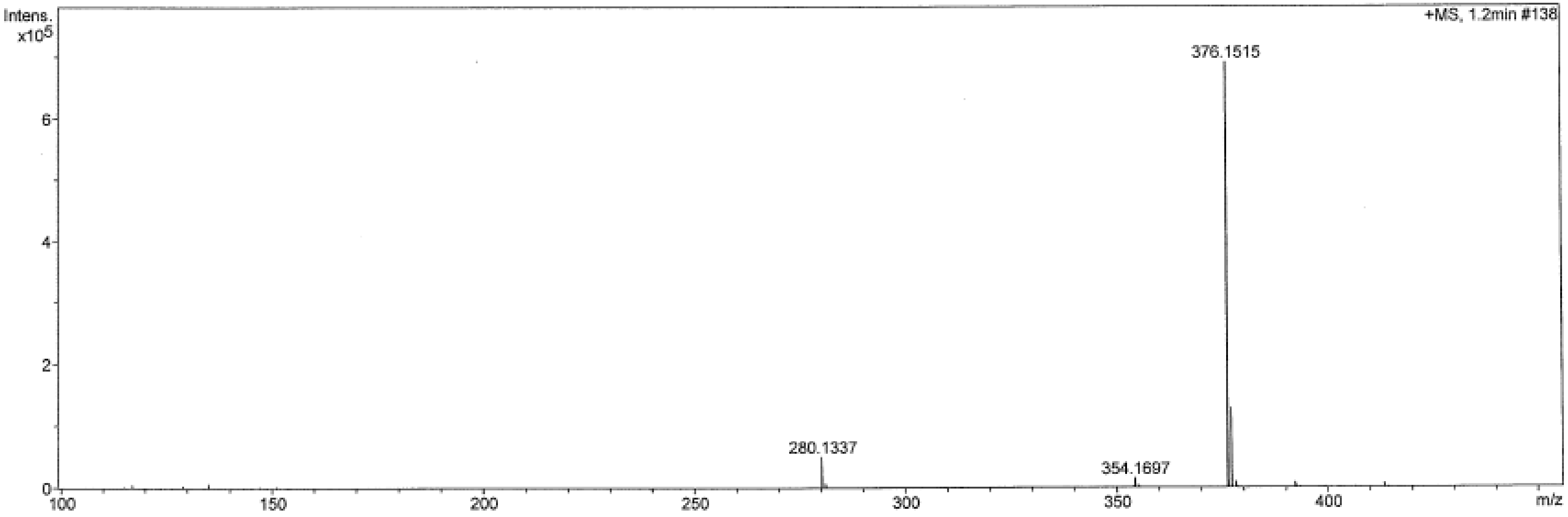

Meas. m/z	#	Formula	m/z	err [ppm]	err [mDa]	mSigma	Mean err [ppm]
354.1697	1	C 21 H 24 N O 4	354.1700	0.9	0.3	29.4	0.7
376.1515	1	C 21 H 23 N Na O 4	376.1519	1.1	0.4	27.8	1.8

PerkinElmer Spectrum Version 10.03.07
01 July 2015 17:33

Analyst	Administrator
Date	01 July 2015 17:33

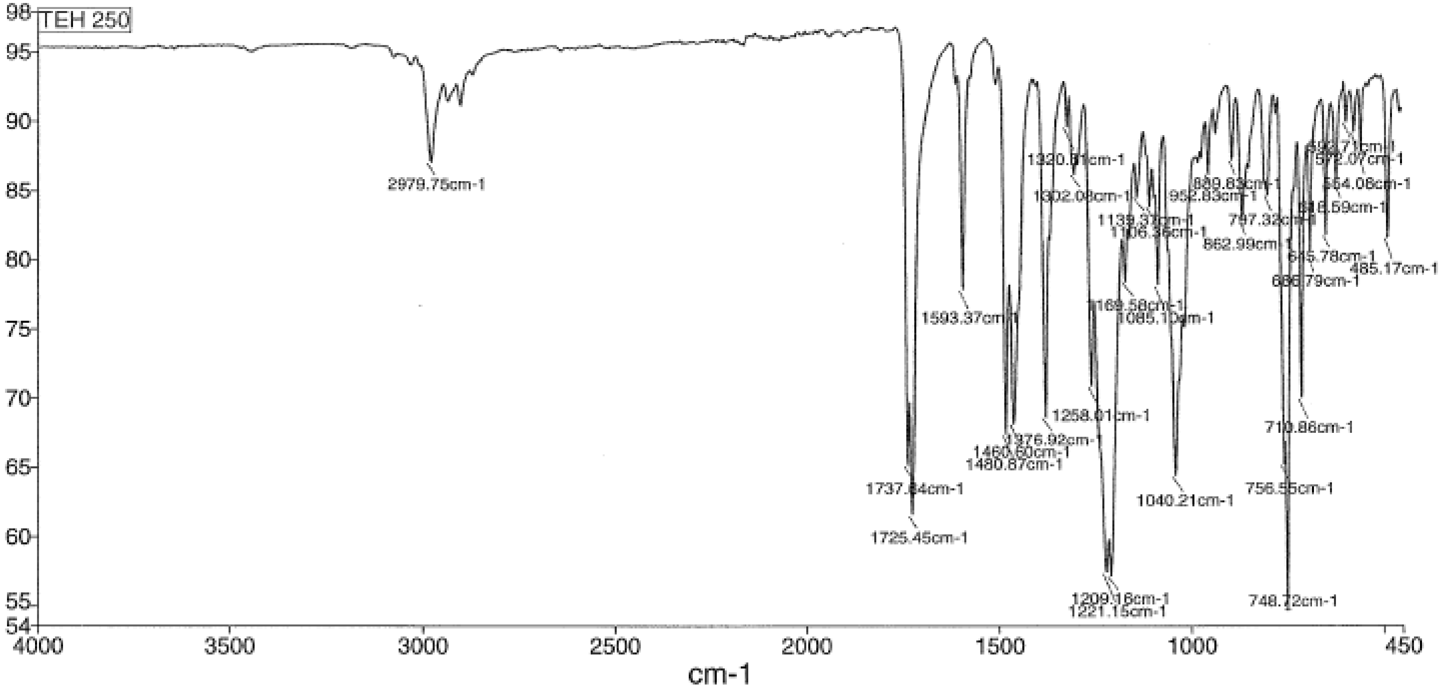

RJKT_01 07 2015_ 49 RJKT_01 07 2015_049

9,9-Diethyl 10-benzyl-9,10-dihydroacridine-9,9-dicarboxylate 16b

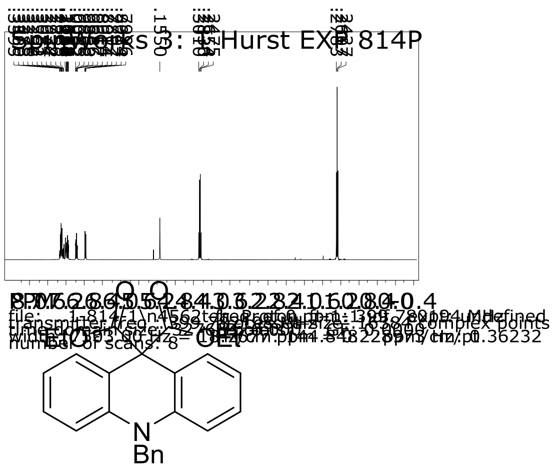

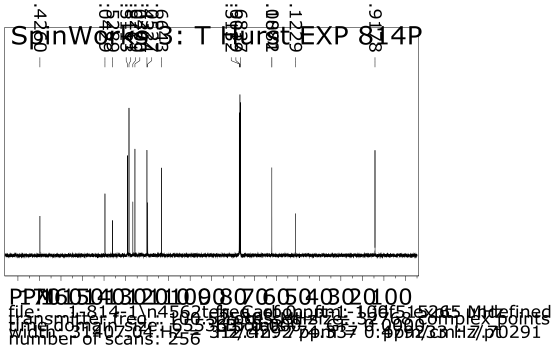
SpinWorks 3: T Hurst EXP 814P
number of scans: 256

teh291

York - Chemistry - Mass Spectrometry Service Report

Analysis Information

Acquisition Date 17/07/2015 11:31:22

Analysis Filename	rjkt53630th_P1-F-1_01_59865.d
Method	600p_meoh1260_2c1s.m
Submission Name	rjkt53630th
Instrument	micrOTOF
ESI	Positive

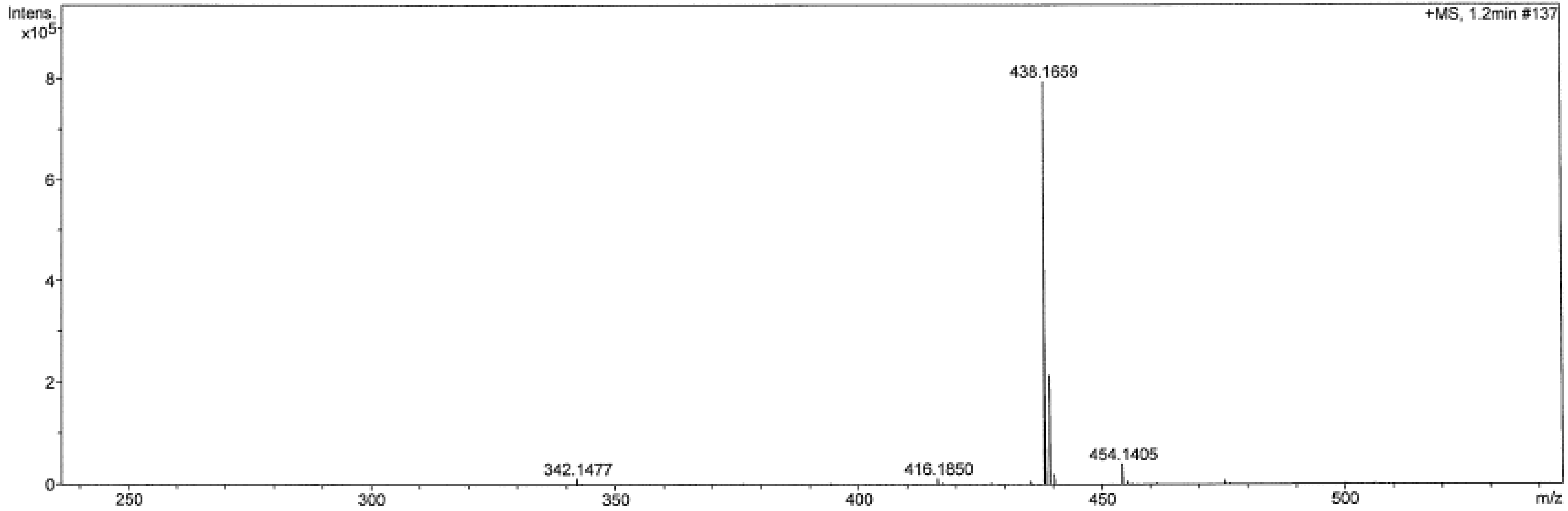

Meas. m/z	#	Formula	m/z	err [ppm]	err [mDa]	mSigma	Mean err [ppm]
416.1850	1	C 26 H 26 N O 4	416.1856	1.6	0.7	54.8	1.5
438.1659	1	C 26 H 25 N Na O 4	438.1676	3.7	1.6	15.8	4.3

PerkinElmer Spectrum Version 10.03.07
01 July 2015 17:36

Analyst Administrator
Date 01 July 2015 17:36

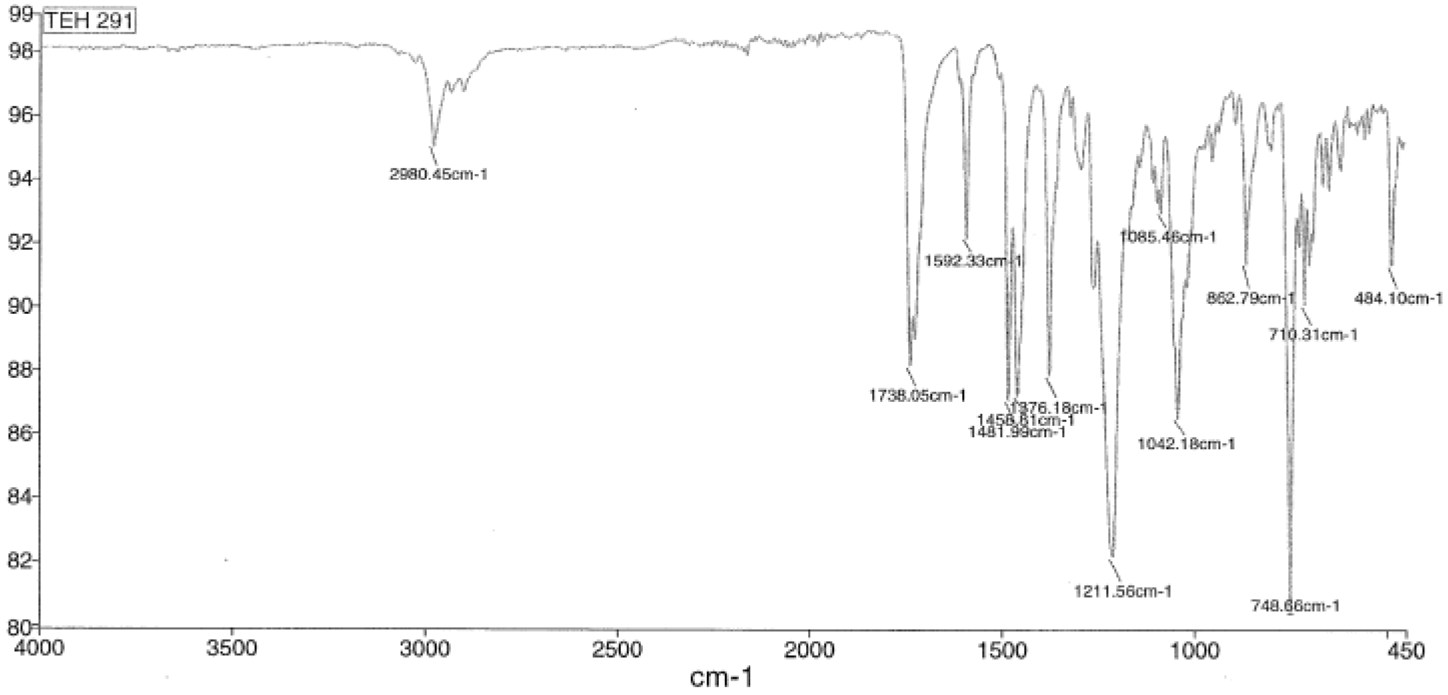

RJKT_01 07 2015_ 50 RJKT_01 07 2015_050

9-Benzyl-9-ethyl 10-ethyl-9,10-dihydroacridine-9,9-dicarboxylate 16c

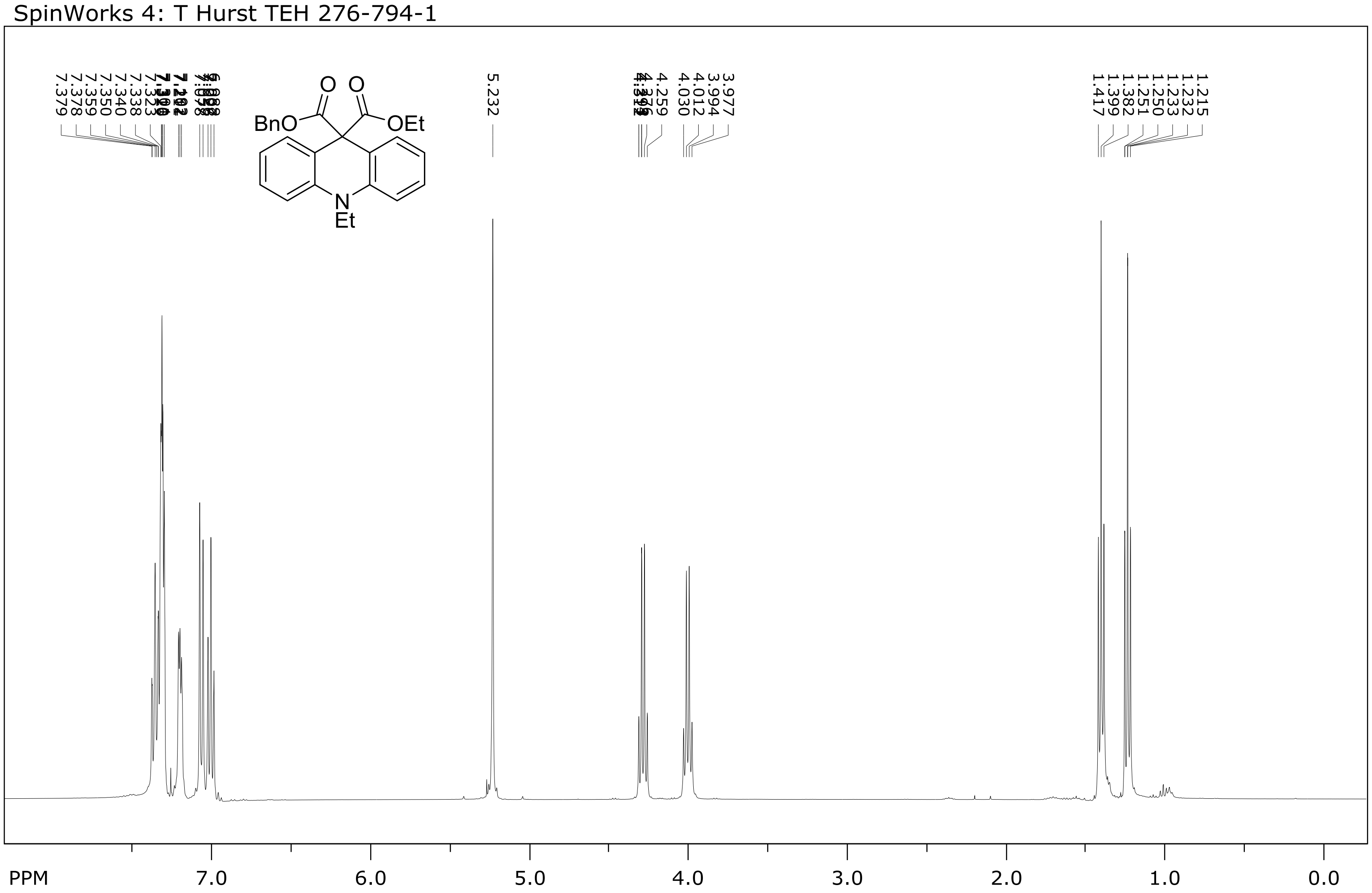

file: ...EH 276-794-1\d4251teh_PROTON-1.jdf expt: single_pulse.ex2
transmitter freq.: 399.782198 MHz
time domain size: 32768 points
width: 7503.00 Hz = 18.7677 ppm = 0.228973 Hz/pt
number of scans: 16

freq. of 0 ppm: 399.780194 MHz
processed size: 32768 complex points
LB: 0.458 GF: 0.0000

SpinWorks 4: T Hurst TEH 276-794-1

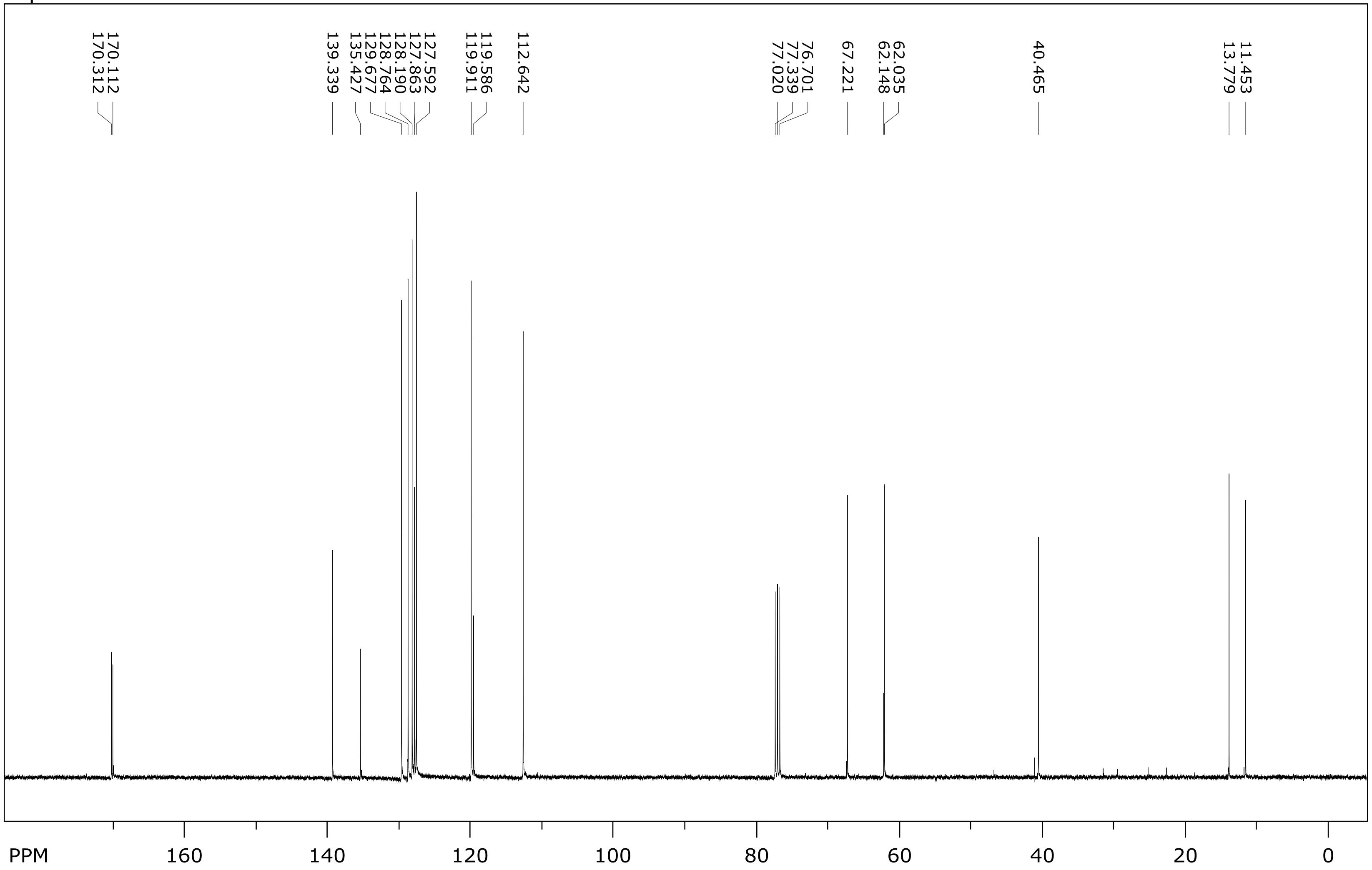

file: ...EH 276-794-1\d4252teh_CARBON-1.jdf expt: single_pulse_dec
transmitter freq.: 100.525303 MHz
time domain size: 65536 points
width: 31407.04 Hz = 312.4292 ppm = 0.479233 Hz/pt
number of scans: 256

freq. of 0 ppm: 100.515277 MHz
processed size: 65536 complex points
LB: 0.958 GF: 0.0000

teh276

York - Chemistry - Mass Spectrometry Service Report

Analysis Information

Acquisition Date 23/01/2015 16:34:22

Analysis Filename	rjkt50543th_P1-A-2_01_56291.d
Method	600p_meoh1260_2c1s.m
Submission Name	rjkt50543th
Instrument	micrOTOF
ESI	Positive

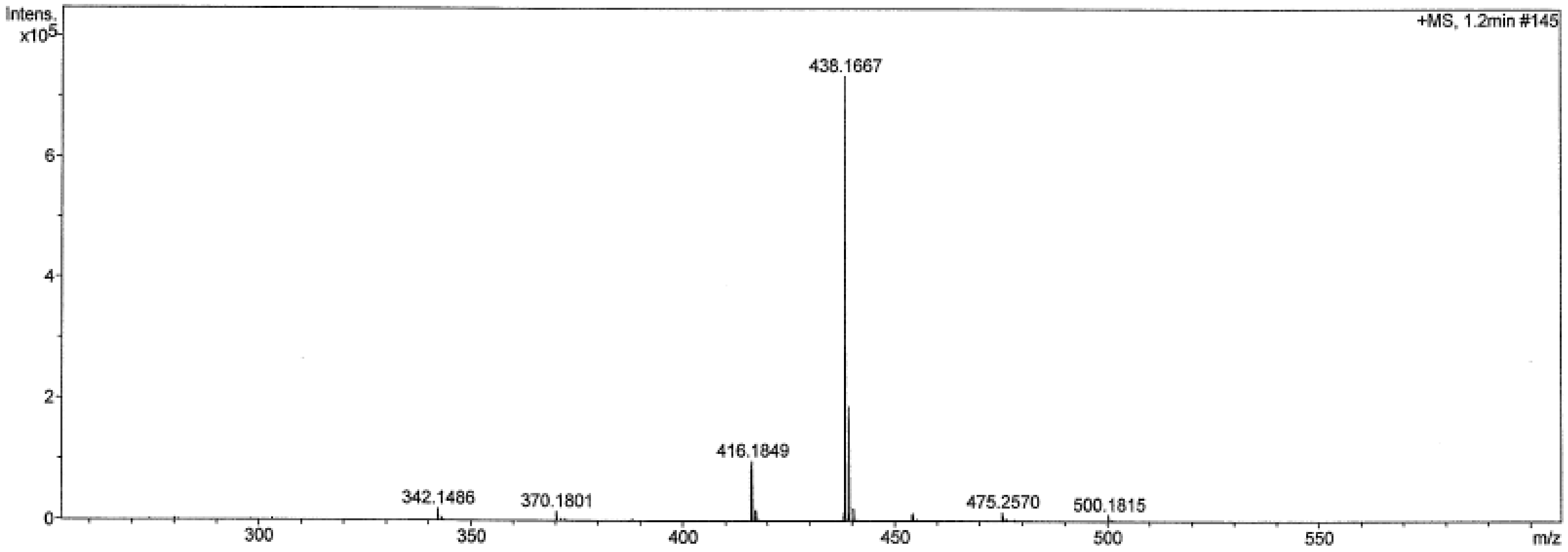

Meas. m/z	#	Formula	m/z	err [ppm]	err [mDa]	mSigma	Mean err [ppm]
416.1849	1	C 26 H 26 N O 4	416.1856	1.9	0.8	57.4	2.0
438.1667	1	C 26 H 25 N Na O 4	438.1676	1.9	0.8	20.3	2.3

PerkinElmer Spectrum Version 10.03.07
01 July 2015 17:40

Analyst Administrator
Date 01 July 2015 17:40

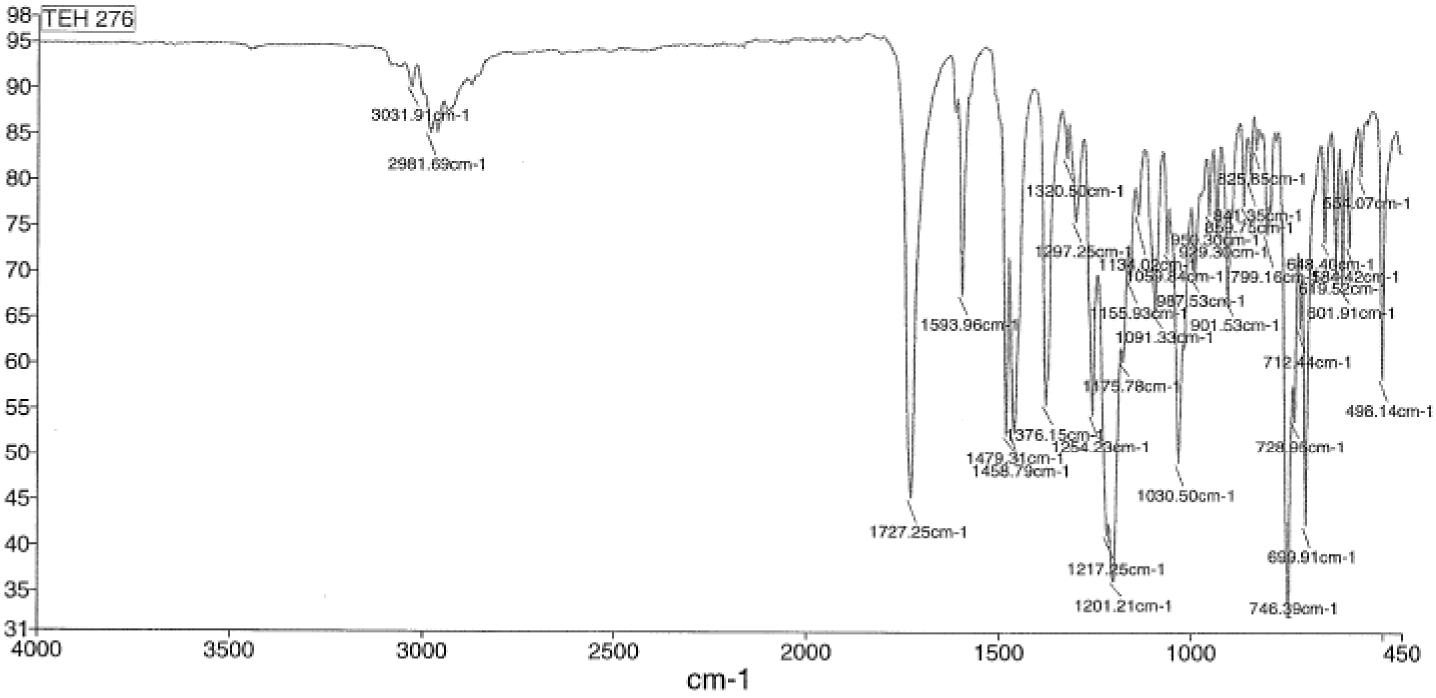

RJKT_01 07 2015_ 51 RJKT_01 07 2015_051

9-Allyl-9-ethyl 10-ethyl-9,10-dihydroacridine-9,9-dicarboxylate 16d

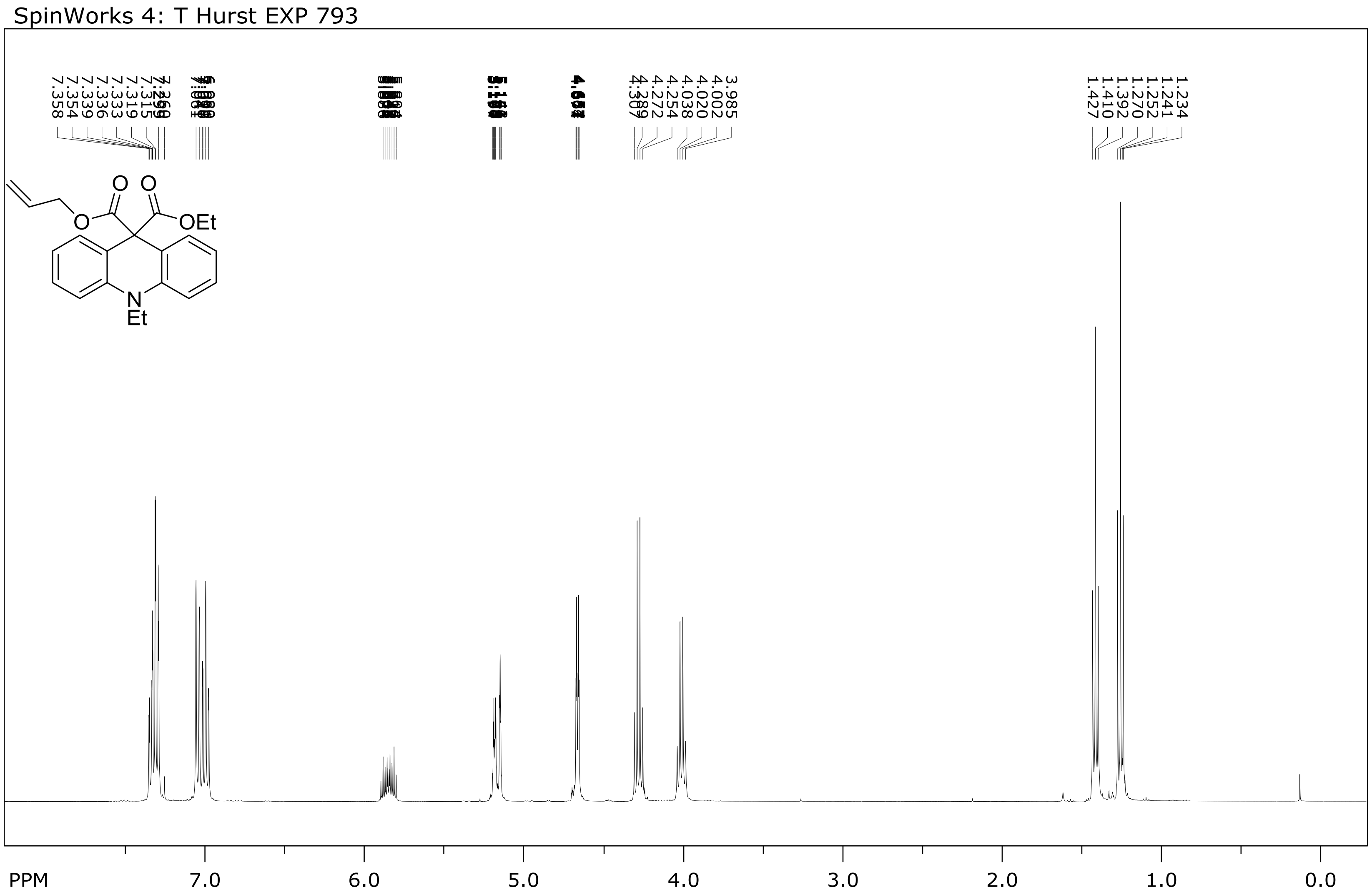

file: ...EH 275-793-1\d3424teh_PROTON-1.jdf   expt: single_pulse.ex2
transmitter freq.: 399.782198 MHz
time domain size: 32768 points
width: 7503.00 Hz = 18.7677 ppm = 0.228973 Hz/pt
number of scans: 16

freq. of 0 ppm: 399.780194 MHz
processed size: 32768 complex points
LB: 0.458   GF: 0.0000

SpinWorks 4: T Hurst EXP 793

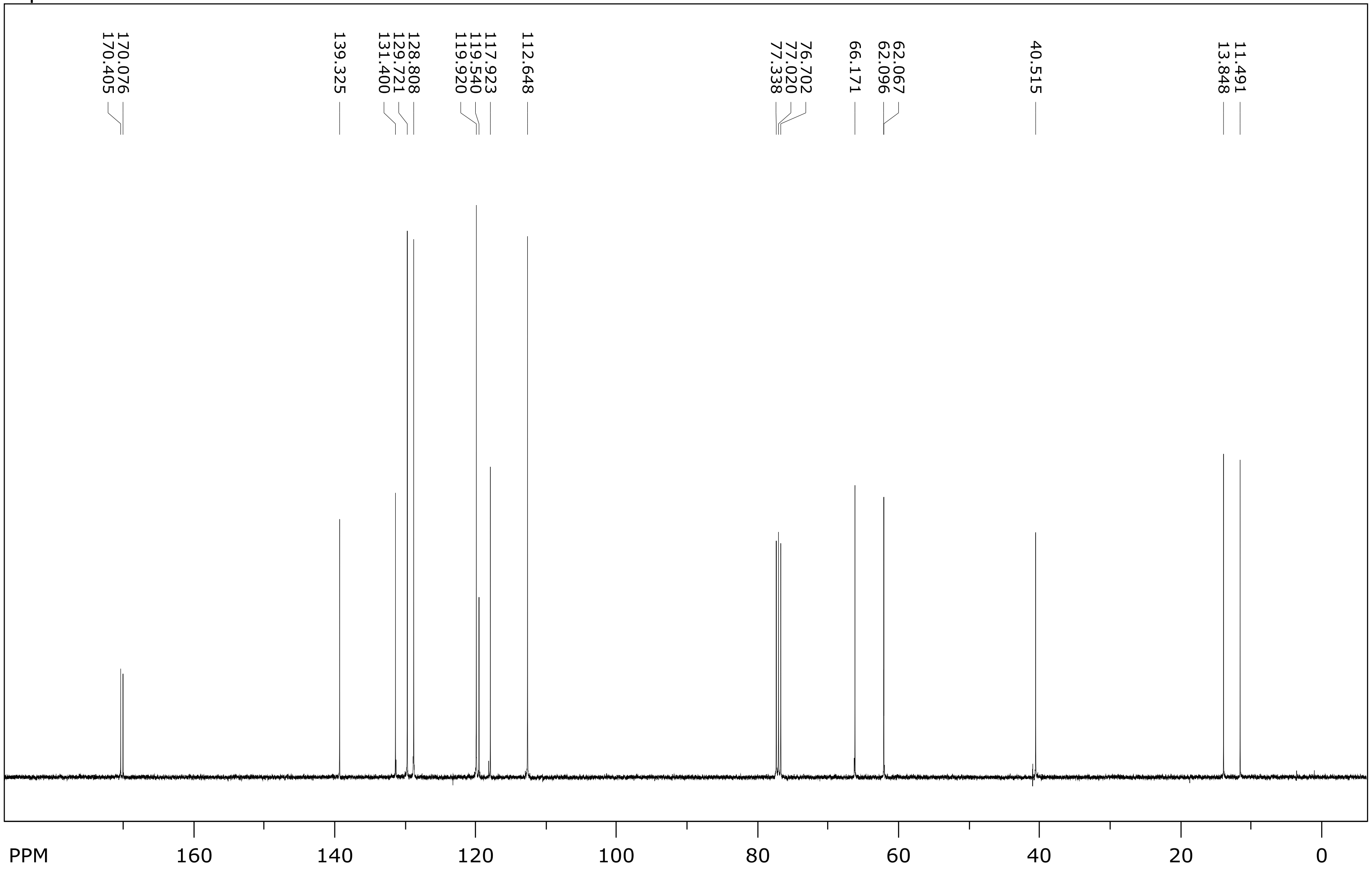

file: ...EH 275-793-1\d3425teh_CARBON-1.jdf expt: single_pulse_dec
transmitter freq.: 100.525303 MHz
time domain size: 65536 points
width: 31407.04 Hz = 312.4292 ppm = 0.479233 Hz/pt
number of scans: 256

freq. of 0 ppm: 100.515271 MHz
processed size: 65536 complex points
LB: 0.958 GF: 0.0000

teh275

York - Chemistry - Mass Spectrometry Service Report

Analysis Information

Acquisition Date 23/01/2015 16:31:25

Analysis Filename	rjkt50542th_P1-A-1_01_56290.d
Method	400p_meoh1260_2c1s.m
Submission Name	rjkt50542th
Instrument	micrOTOF
ESI	Positive

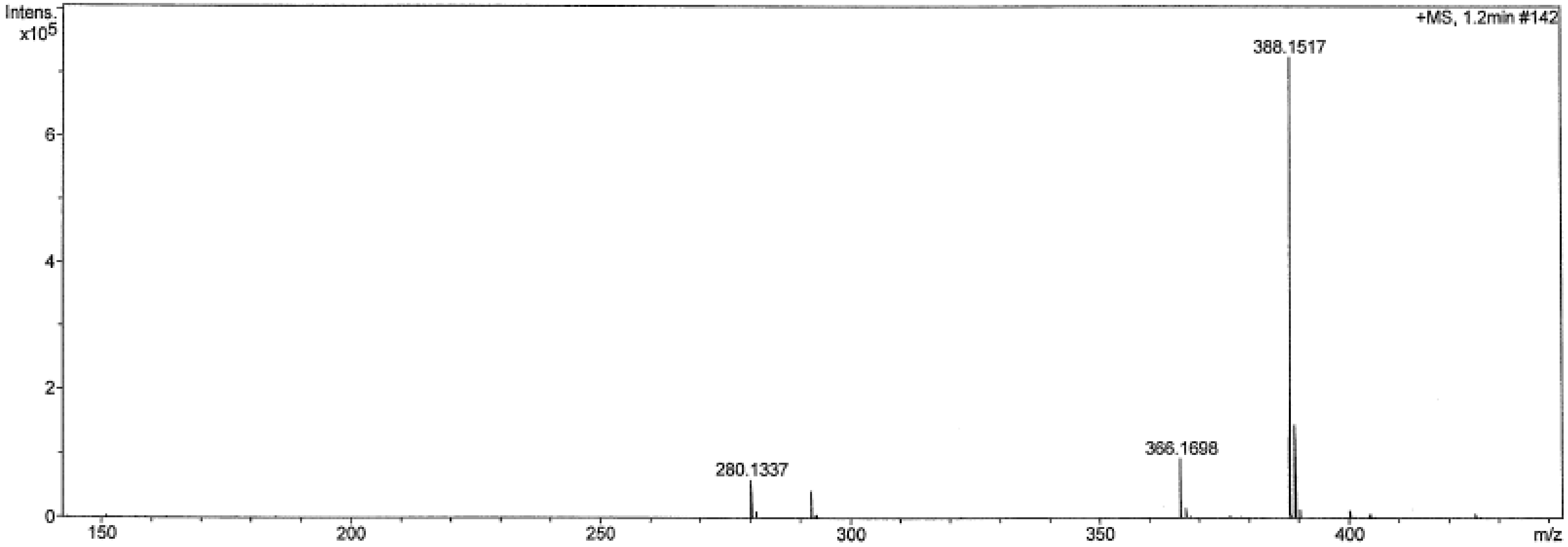

Meas. m/z	#	Formula	m/z	err [ppm]	err [mDa]	mSigma	Mean err [ppm]
366.1698	1	C 22 H 24 N O 4	366.1700	0.4	0.1	46.1	0.5
388.1517	1	C 22 H 23 N Na O 4	388.1519	0.7	0.3	26.1	1.7

PerkinElmer Spectrum Version 10.03.07
01 July 2015 17:43

Analyst Administrator
Date 01 July 2015 17:43

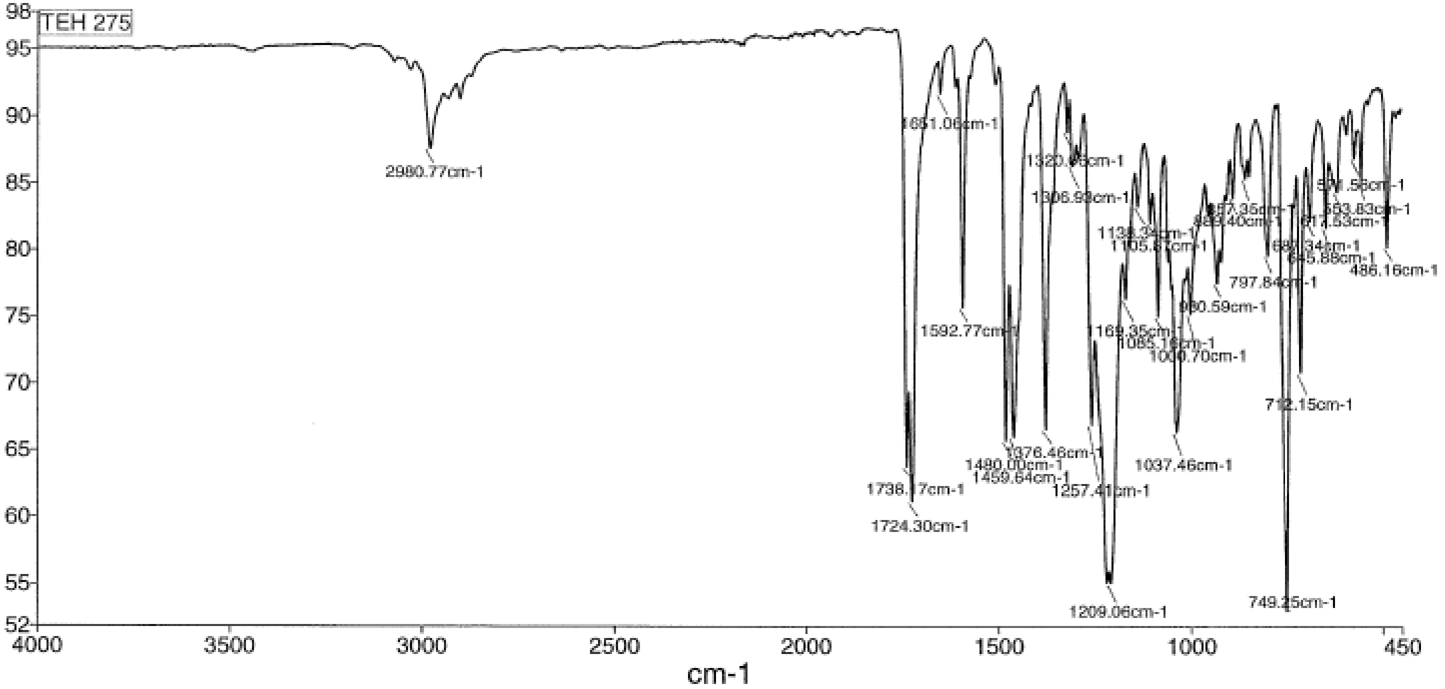

RJKT_01 07 2015_ 52 RJKT_01 07 2015_052

9-Ethyl-9-(9*H*-fluoren-9-ylmethyl) 10-ethyl-9,10-dihydroacridine-9,9-dicarboxylate 16e

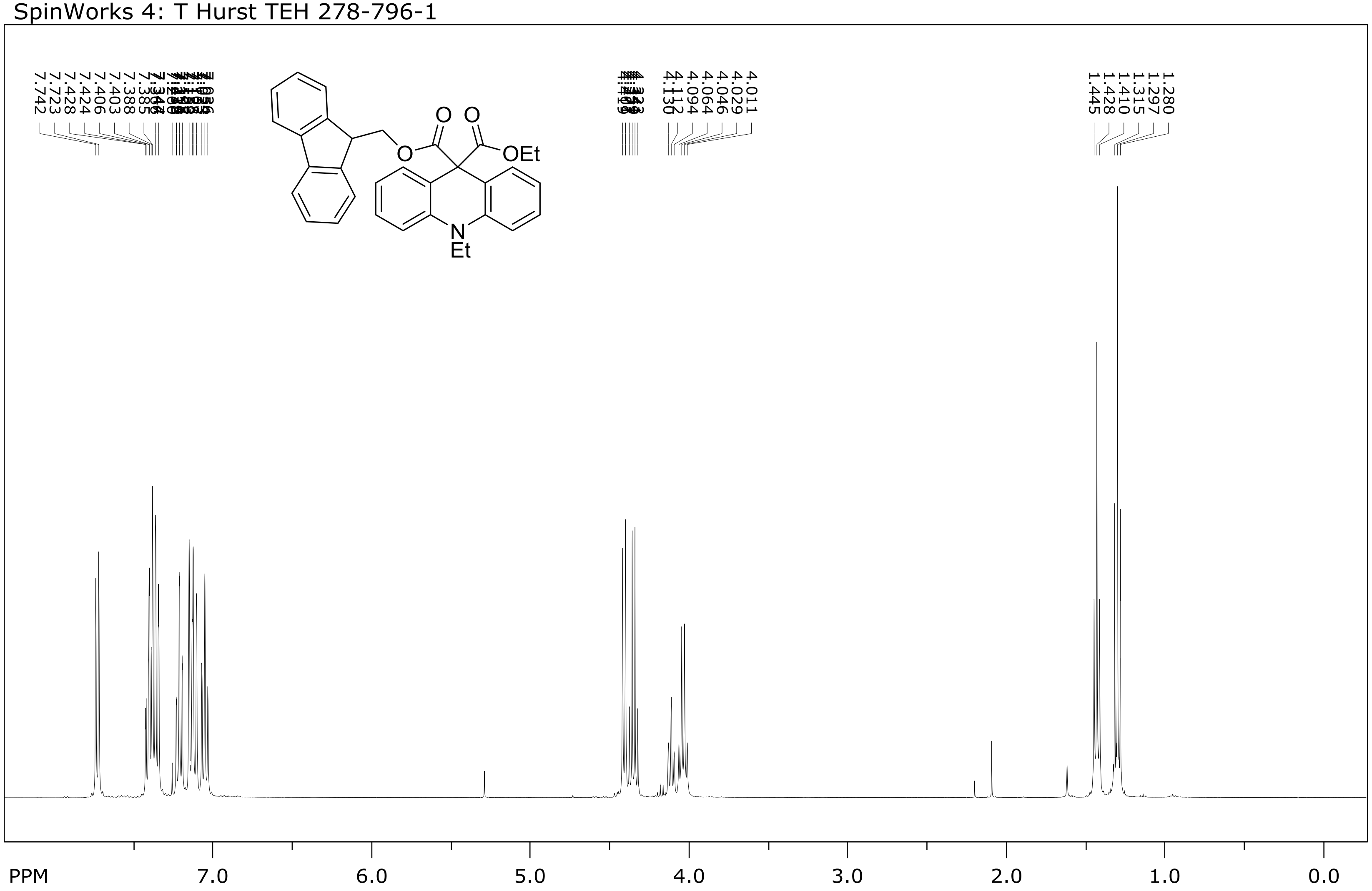

file: ...EH 278-796-1\d4255teh_PROTON-1.jdf expt: single_pulse.ex2
transmitter freq.: 399.782198 MHz
time domain size: 32768 points
width: 7503.00 Hz = 18.7677 ppm = 0.228973 Hz/pt
number of scans: 16

freq. of 0 ppm: 399.780194 MHz
processed size: 32768 complex points
LB: 0.458 GF: 0.0000

SpinWorks 4: T Hurst TEH 278-796-1

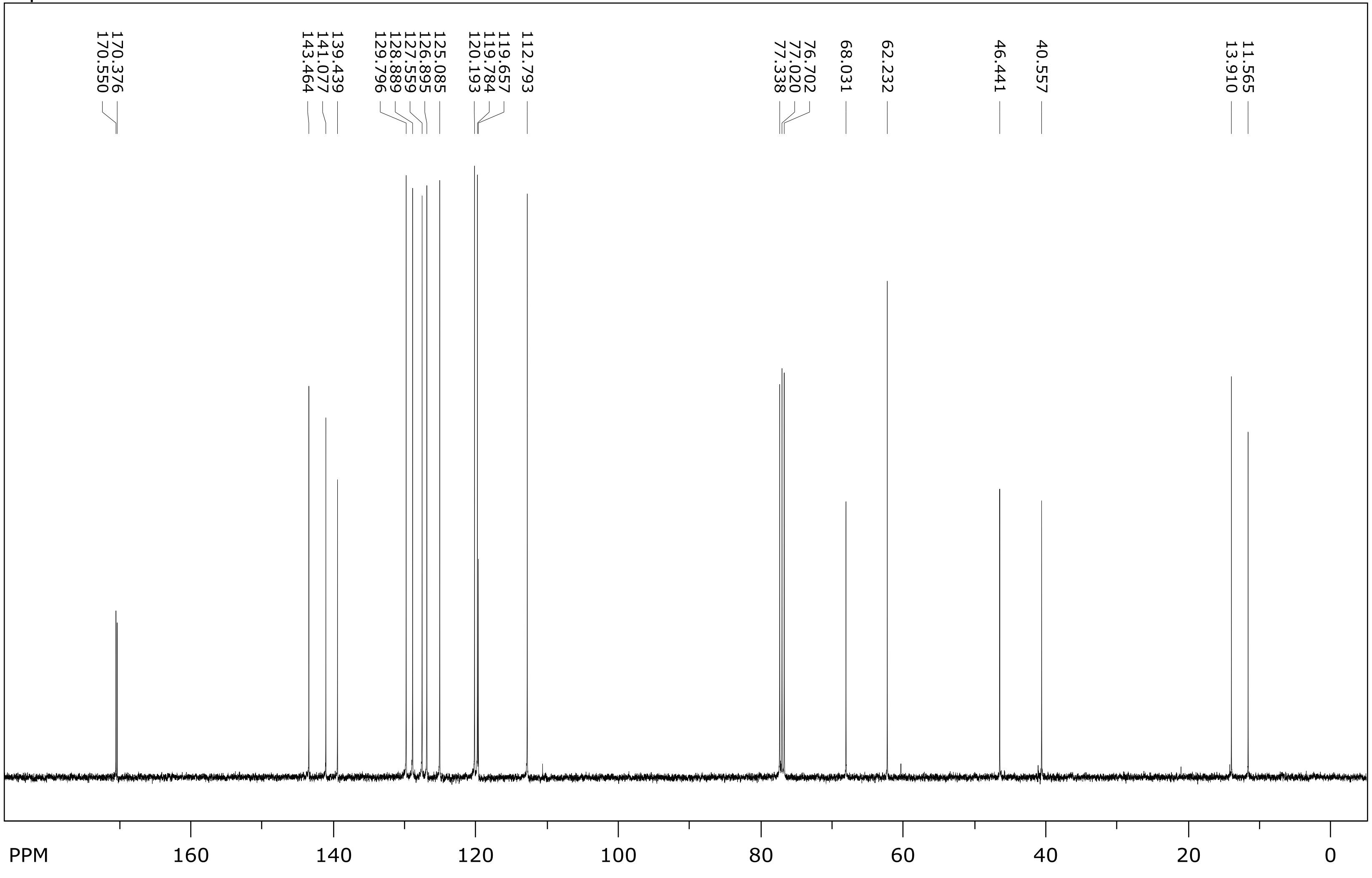

file: ...EH 278-796-1\d4256teh_CARBON-1.jdf expt: single_pulse_dec
transmitter freq.: 100.525303 MHz
time domain size: 65536 points
width: 31407.04 Hz = 312.4292 ppm = 0.479233 Hz/pt
number of scans: 256

freq. of 0 ppm: 100.515272 MHz
processed size: 65536 complex points
LB: 0.958 GF: 0.0000

teh278

York - Chemistry - Mass Spectrometry Service Report

Analysis Information

Acquisition Date 23/01/2015 16:40:14

Analysis Filename	rjkt50545th_P1-A-4_01_56293.d
Method	600p_meoh1260_2c1s.m
Submission Name	rjkt50545th
Instrument	micrOTOF
ESI	Positive

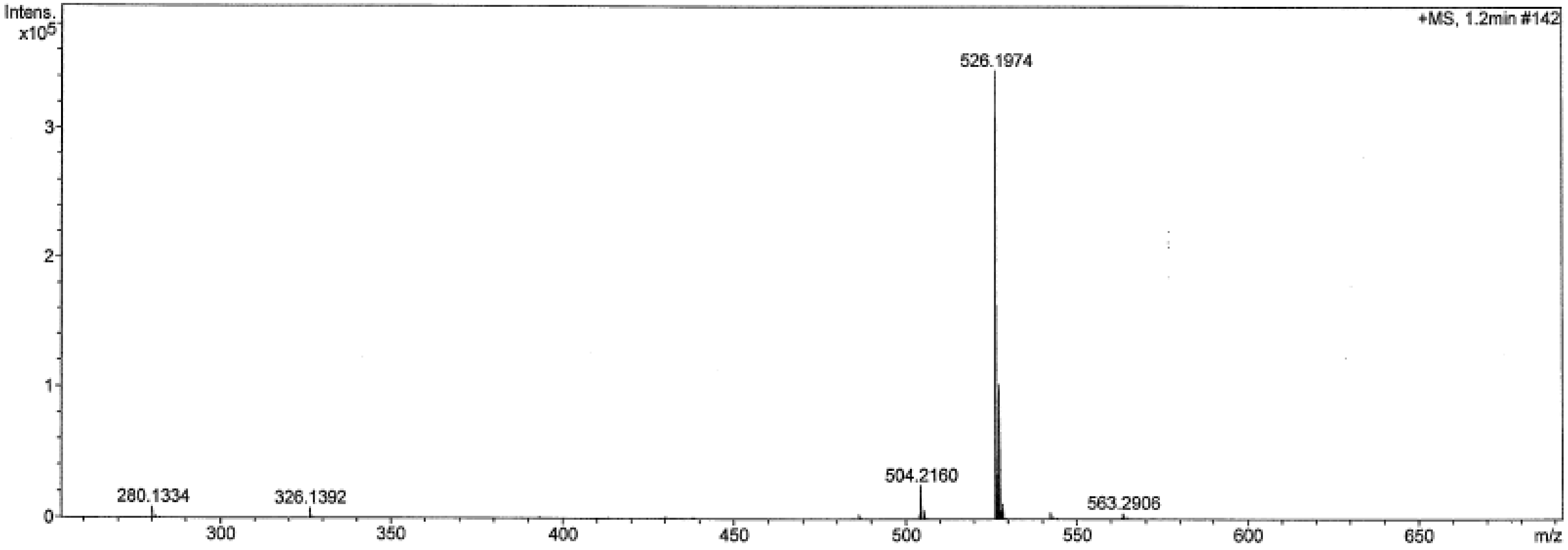

Meas. m/z	#	Formula	m/z	err [ppm]	err [mDa]	mSigma	Mean err [ppm]
504.2160	1	C 33 H 30 N O 4	504.2169	1.8	0.9	46.0	1.6
526.1974	1	C 33 H 29 N Na O 4	526.1989	2.7	1.4	36.8	2.7

PerkinElmer Spectrum Version 10.03.07
01 July 2015 17:45

Analyst Administrator
Date 01 July 2015 17:45

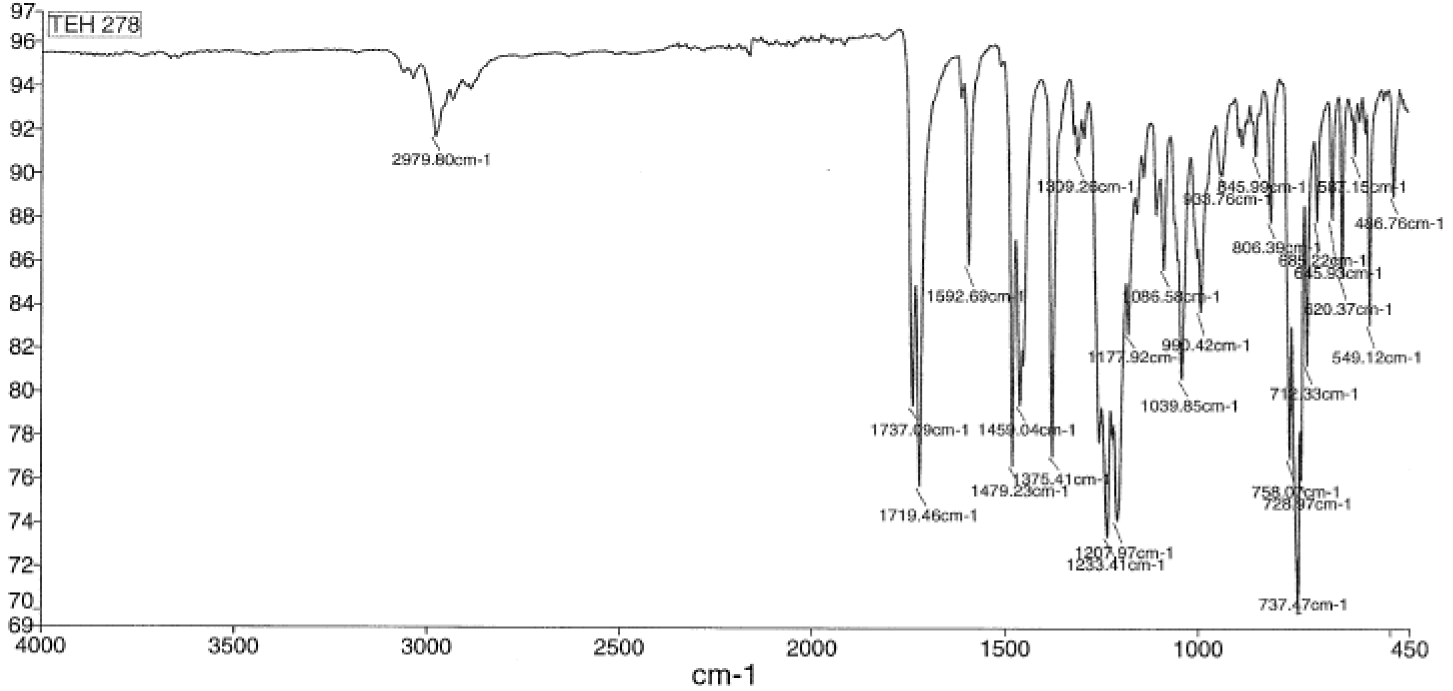

RJKT_01 07 2015_ 53 RJKT_01 07 2015_053

9-Ethyl-9-(*L*-menthyl) 10-ethyl-9,10-dihydroacridine-9,9-dicarboxylate 16f

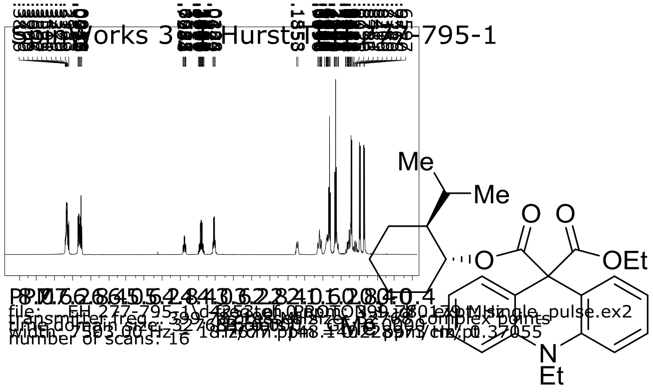

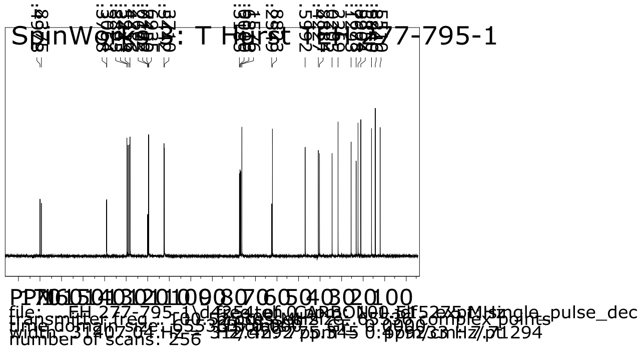
PPM
number of scans: 256

teh277

York - Chemistry - Mass Spectrometry Service Report

Analysis Information

Acquisition Date 23/01/2015 16:37:18

Analysis Filename	rjkt50544th_P1-A-3_01_56292.d
Method	600p_meoh1260_2c1s.m
Submission Name	rjkt50544th
Instrument	micrOTOF
ESI	Positive

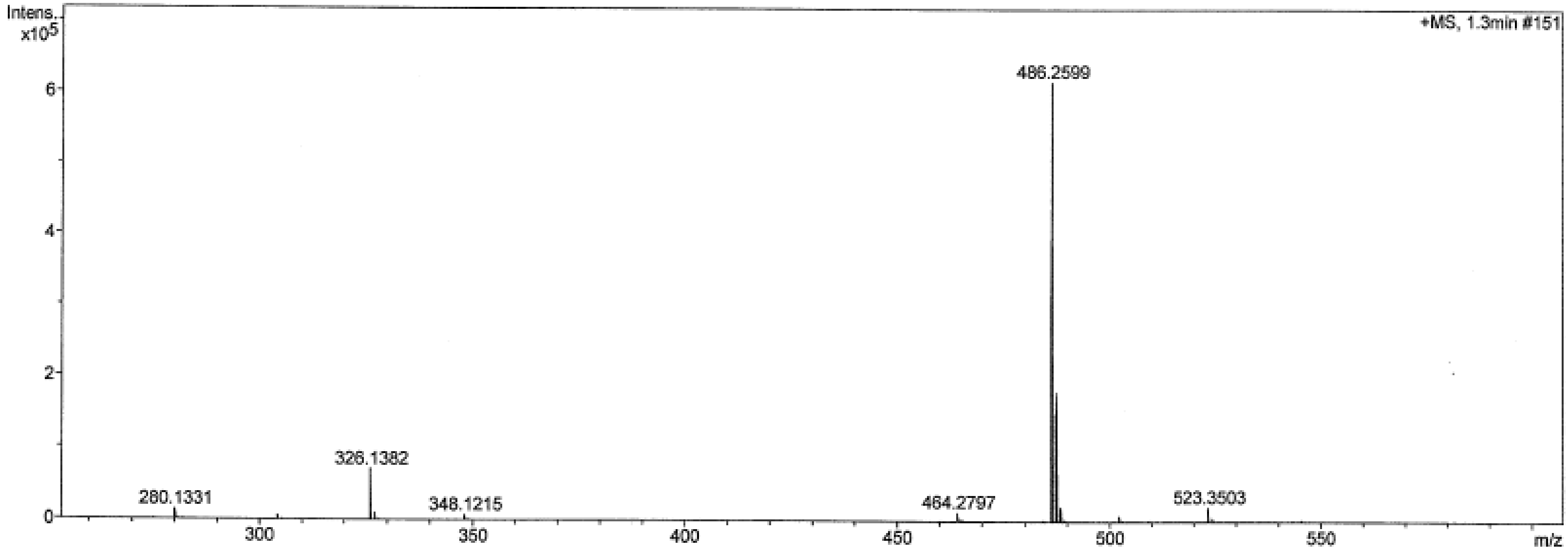

Meas. m/z	#	Formula	m/z	err [ppm]	err [mDa]	mSigma	Mean err [ppm]
486.2599	1	C 29 H 37 N Na O 4	486.2615	3.2	1.6	20.0	3.3

PerkinElmer Spectrum Version 10.03.07
01 July 2015 17:48

Analyst	Administrator
Date	01 July 2015 17:48

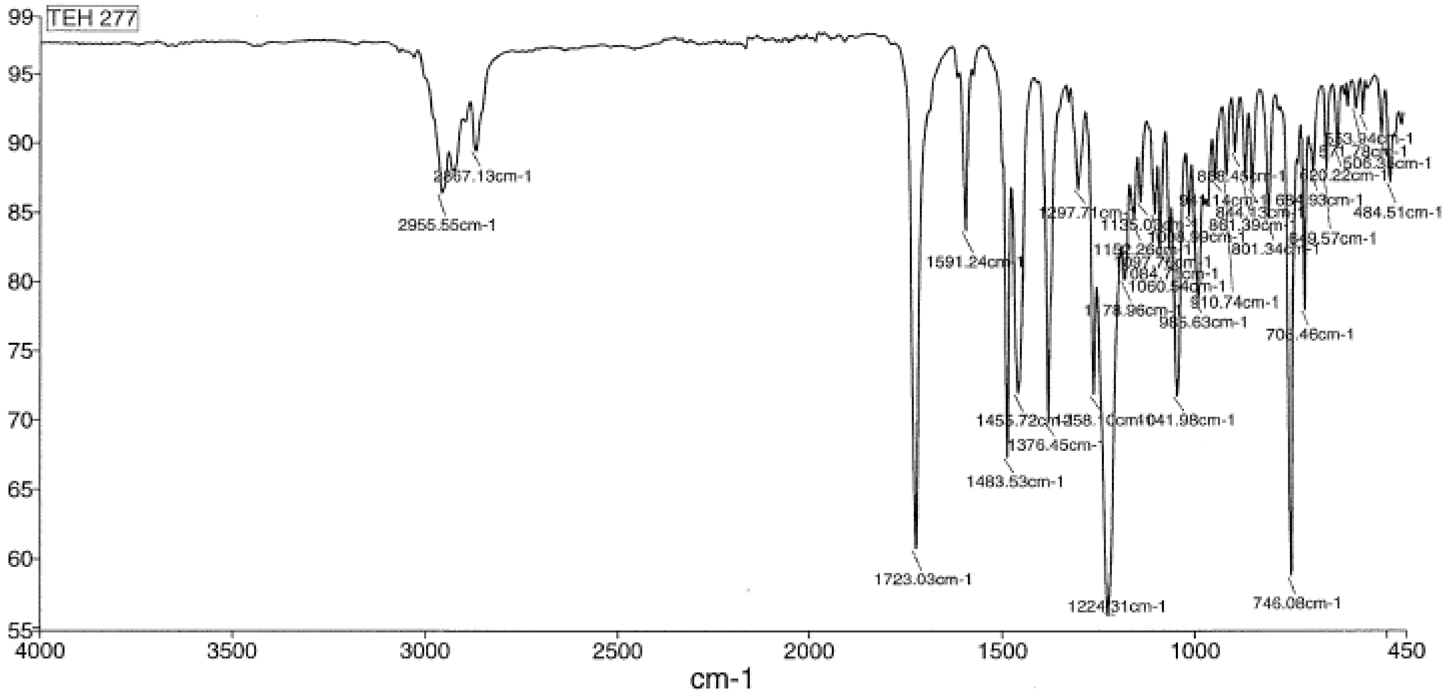

RJKT_01 07 2015_ 54 RJKT_01 07 2015_054

9,9-Diethyl 10-ethyl-2-fluoro-9,10-dihydroacridine-9,9-dicarboxylate 16g

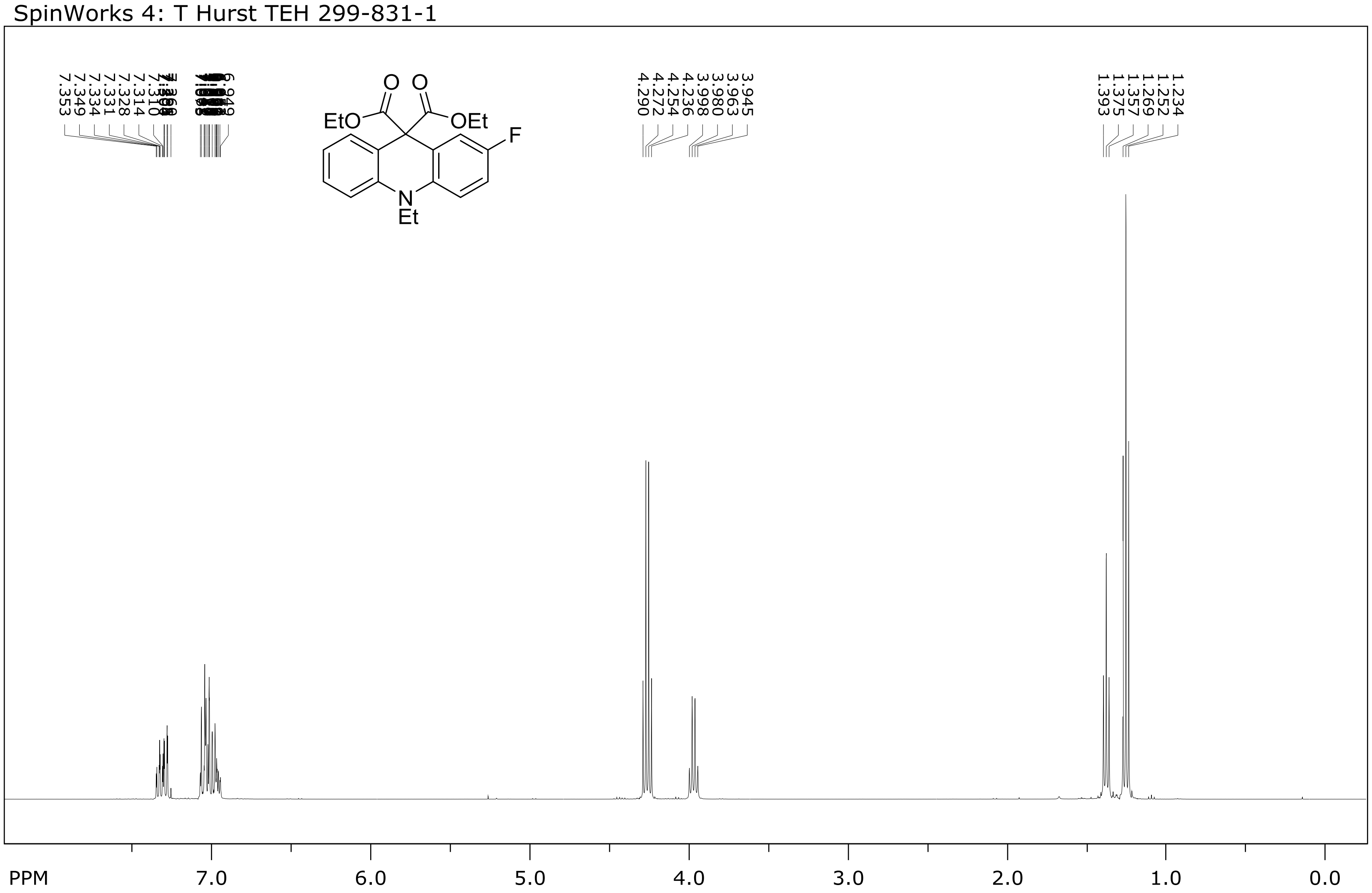

file: ...9-831-1\n6760teh_Proton_ft-1-1.jdf expt: undefined
transmitter freq.: 399.782198 MHz
time domain size: 32768 points
width: 7503.00 Hz = 18.7677 ppm = 0.228973 Hz/pt
number of scans: 8

freq. of 0 ppm: 399.780194 MHz
processed size: 16384 complex points
LB: 0.000 GF: 0.0000

SpinWorks 4: T Hurst TEH 299-831-1

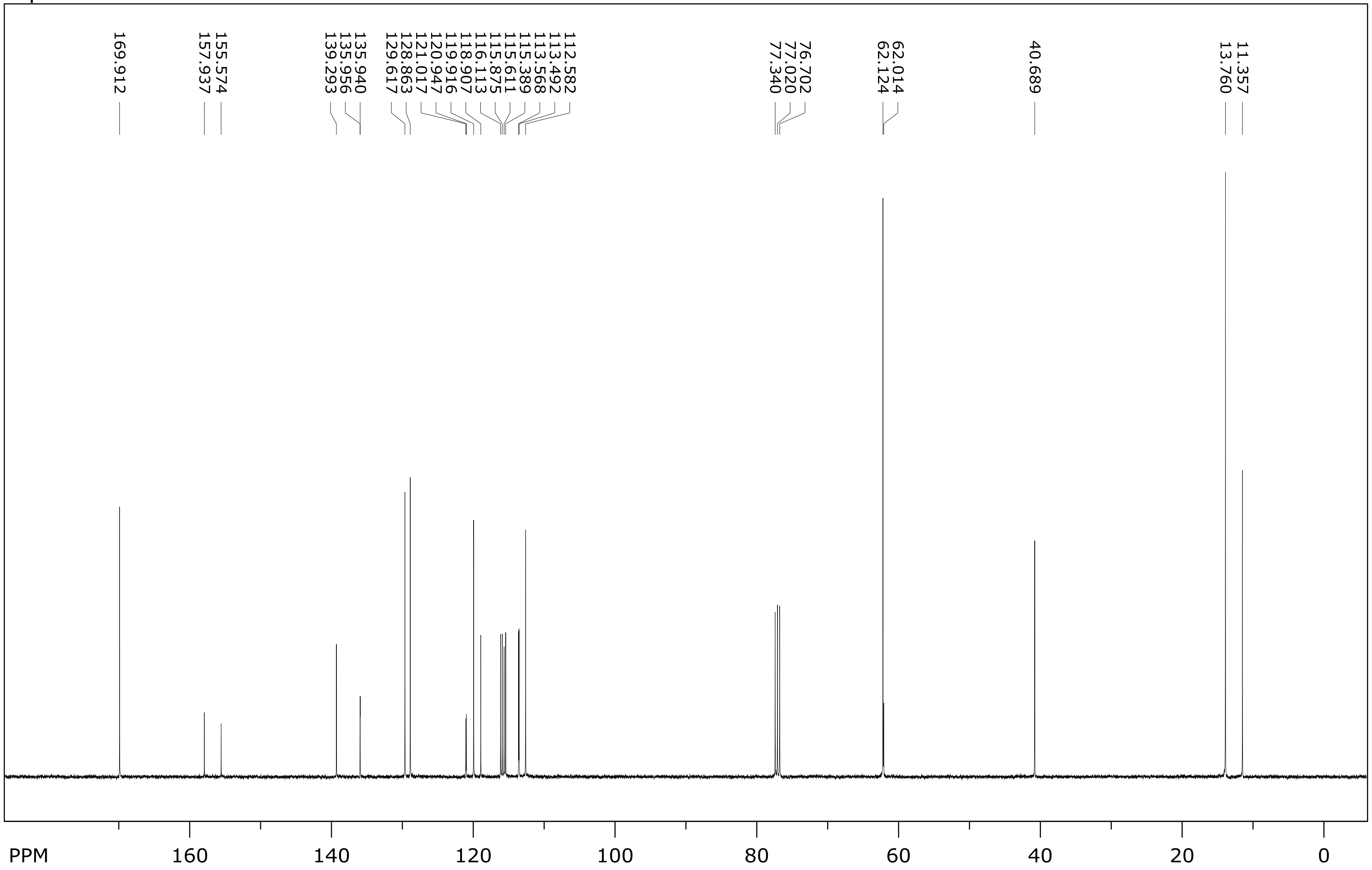

file: ...9-831-1\n6760teh_Carbon_ft-1-1.jdf expt: undefined
transmitter freq.: 100.525303 MHz
time domain size: 65536 points
width: 31407.04 Hz = 312.4292 ppm = 0.479233 Hz/pt
number of scans: 256

freq. of 0 ppm: 100.515276 MHz
processed size: 32768 complex points
LB: 0.000 GF: 0.0000

teh299

York - Chemistry - Mass Spectrometry Service Report

Analysis Information

Acquisition Date 21/07/2015 14:30:31

Analysis Filename	rjkt53684th_P1-E-2_01_59913.d
Method	400p_meoh1260_2c1s.m
Submission Name	rjkt53684th
Instrument	micrOTOF
ESI	Positive

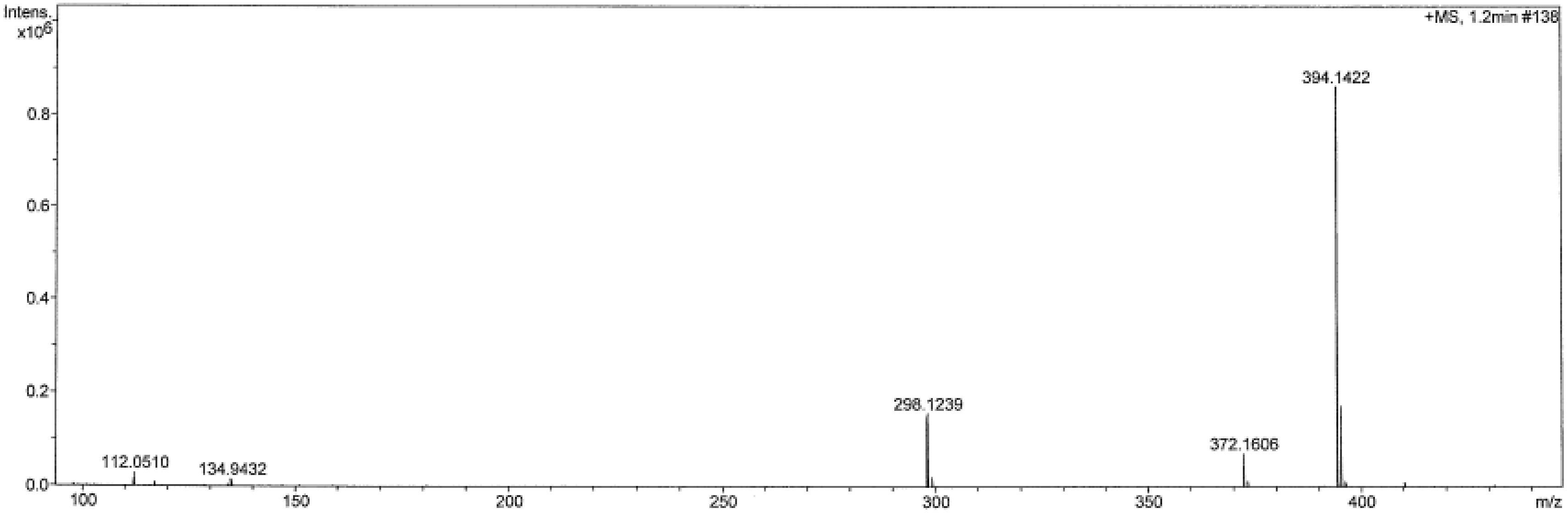

Meas. m/z	#	Formula	m/z	err [ppm]	err [mDa]	mSigma	Mean err [ppm]
372.1606	1	C 21 H 23 F N O 4	372.1606	-0.2	-0.1	40.9	-0.5
394.1422	1	C 21 H 22 F N Na O 4	394.1425	0.7	0.3	21.0	2.0

PerkinElmer Spectrum Version 10.03.07
01 July 2015 17:50

Analyst Administrator
Date 01 July 2015 17:50

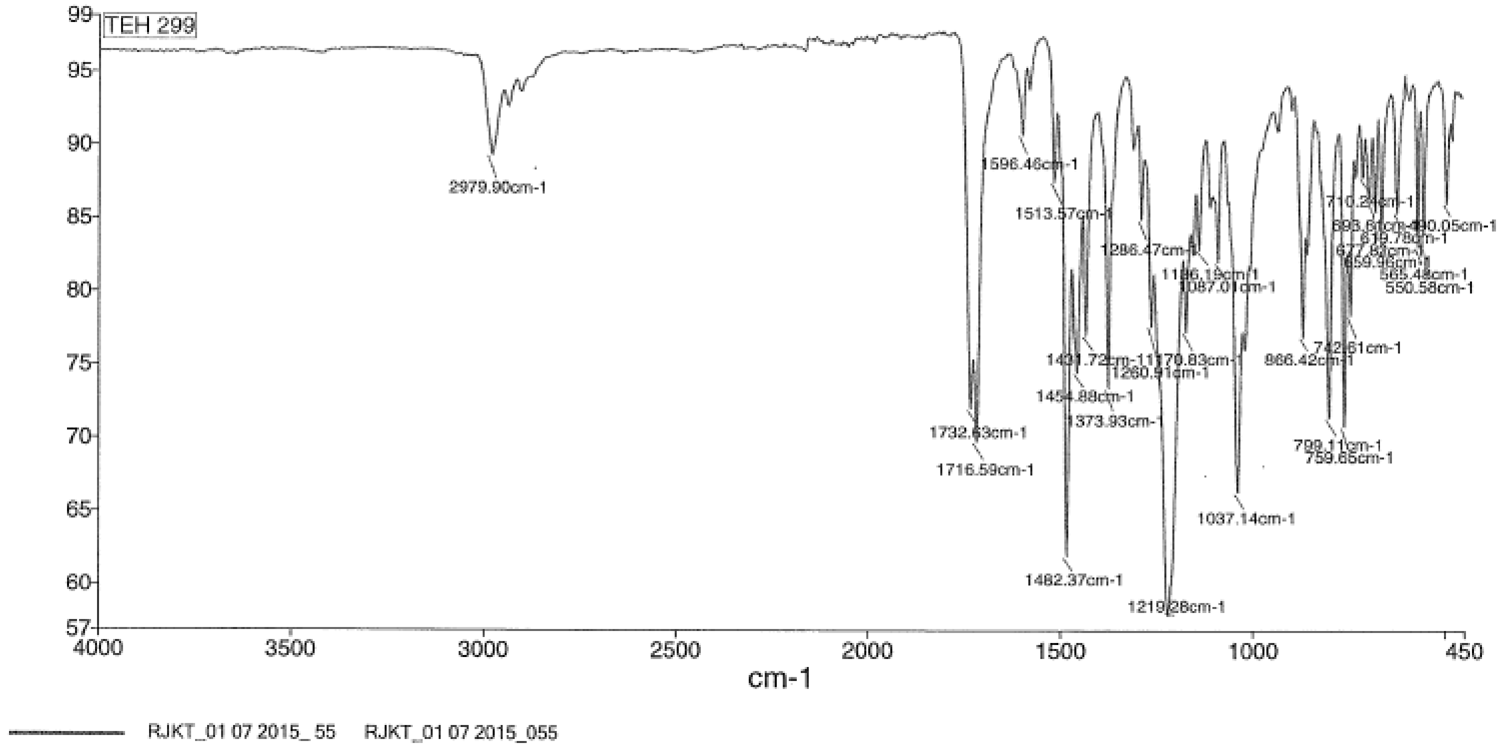

9,9-Diethyl 1,3-dichloro-10-ethyl-9,10-dihydroacridine-9,9-dicarboxylate 16h

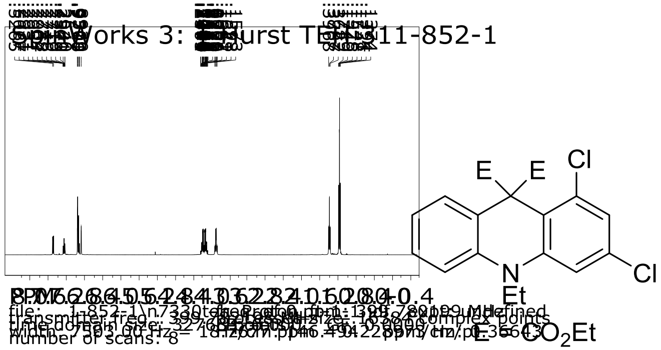

SpinWorks 4: T Hurst TEH 311-852-1

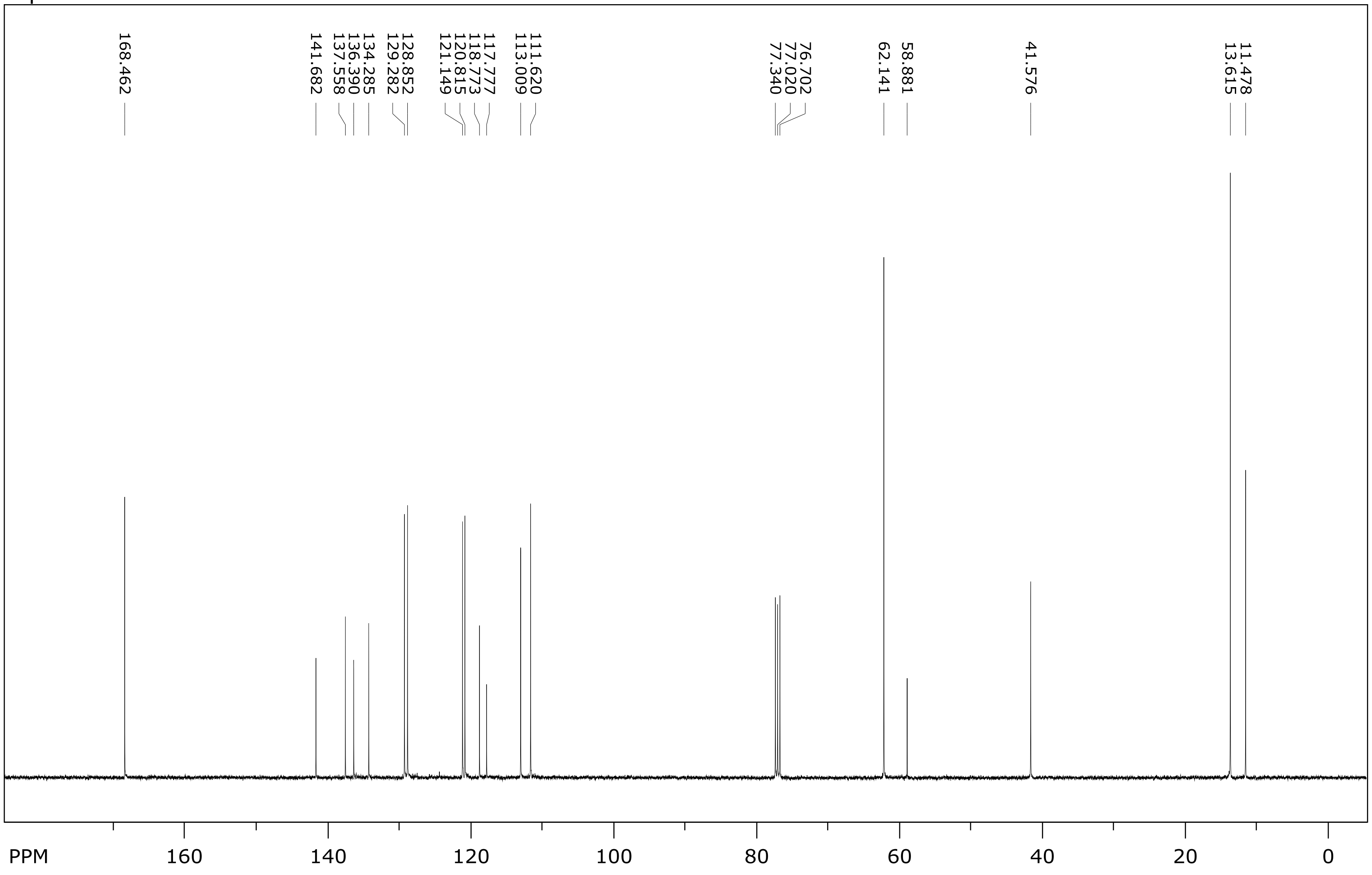

file: ...1-852-1\n7330teh_Carbon_ft-1-1.jdf   expt: undefined
transmitter freq.: 100.525303 MHz
time domain size: 65536 points
width: 31407.04 Hz = 312.4292 ppm = 0.479233 Hz/pt
number of scans: 256

freq. of 0 ppm: 100.515275 MHz
processed size: 32768 complex points
LB: 0.000   GF: 0.0000

teh311

York - Chemistry - Mass Spectrometry Service Report

Analysis Information

Acquisition Date 21/07/2015 14:33:28

Analysis Filename	rjkt53685th_P1-E-3_01_59914.d
Method	600p_meoh1260_2c1s.m
Submission Name	rjkt53685th
Instrument	micrOTOF
ESI	Positive

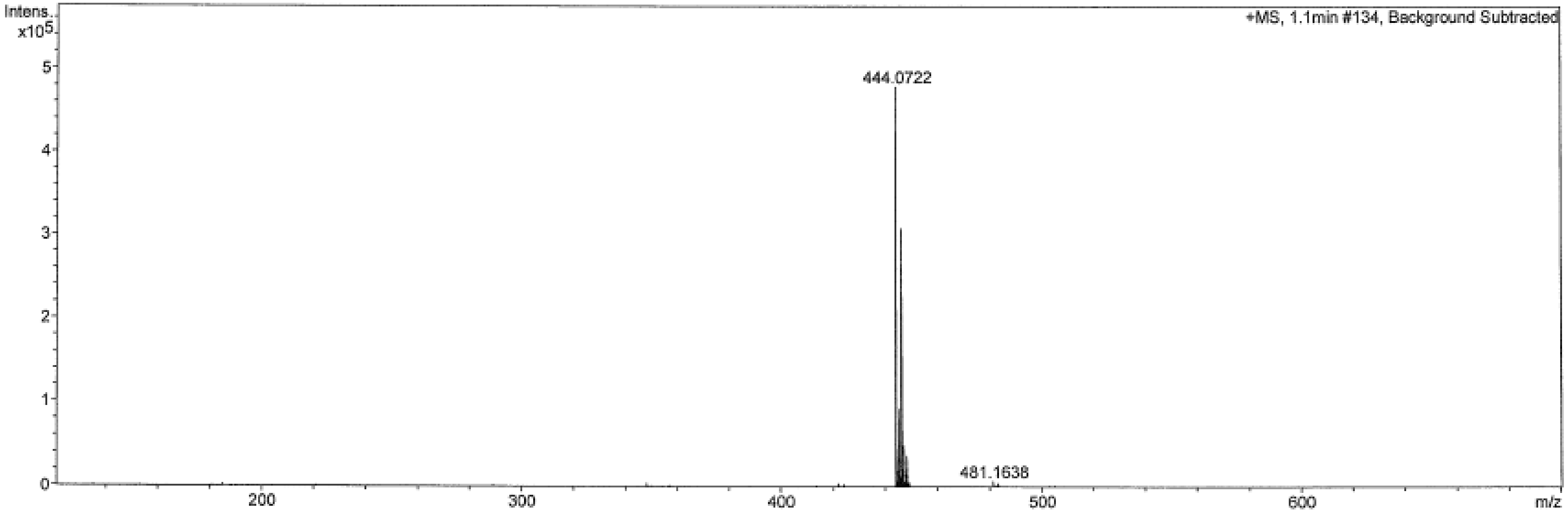

Meas. m/z	#	Formula	m/z	err [ppm]	err [mDa]	mSigma	Mean err [ppm]
444.0722	1	C 21 H 21 Cl 2 N Na O 4	444.0740	4.0	1.8	35.5	3.6

PerkinElmer Spectrum Version 10.03.07
01 July 2015 17:53

Analyst Administrator
Date 01 July 2015 17:53

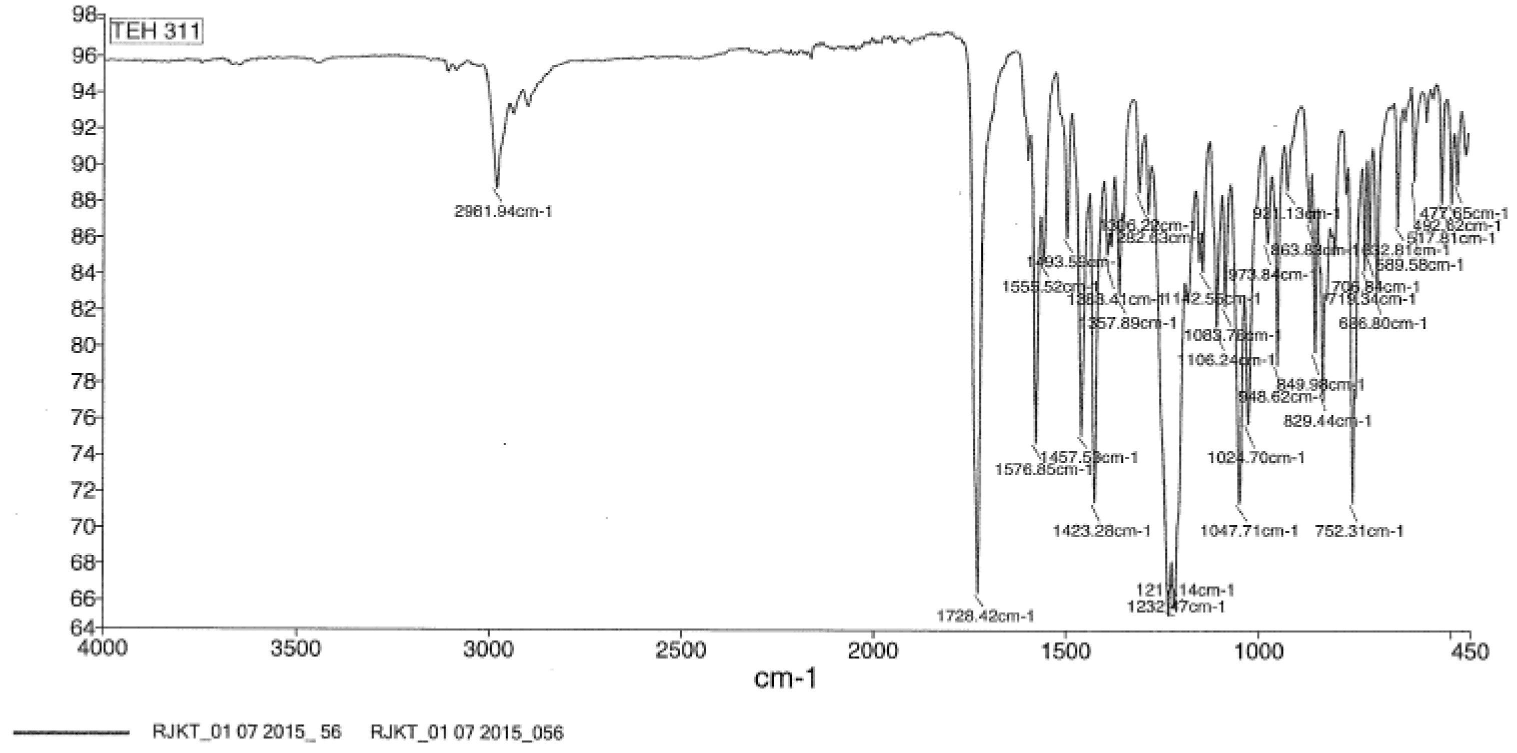

9,9-Diethyl 10-ethyl-1,3-dimethyl-9,10-dihydroacridine-9,9-dicarboxylate 16i

SpinWorks 4: T Hurst TEH 312-853-1

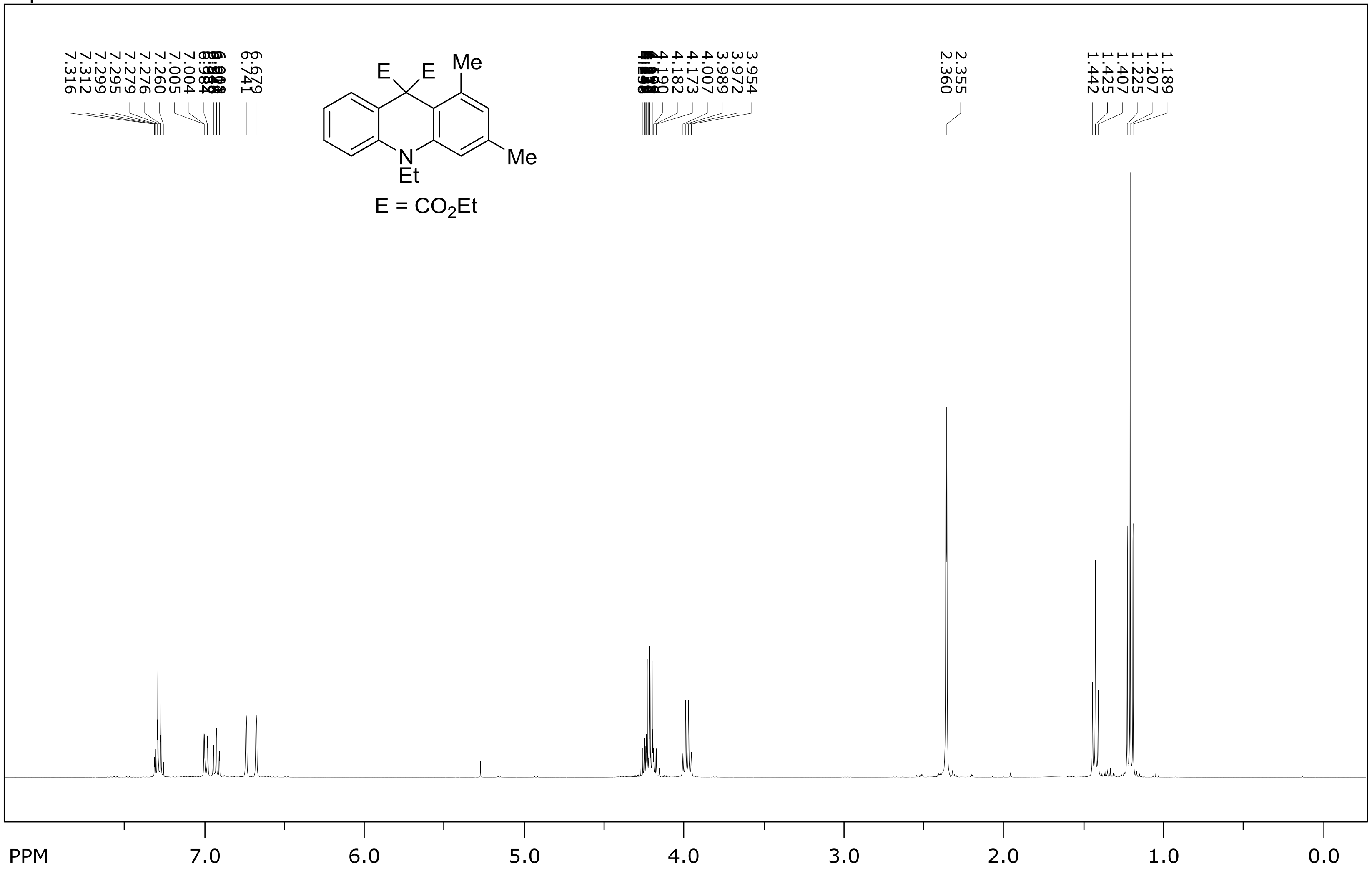

file: ...2-853-1\n7331teh_Proton_ft-1-1.jdf expt: undefined
transmitter freq.: 399.782198 MHz
time domain size: 32768 points
width: 7503.00 Hz = 18.7677 ppm = 0.228973 Hz/pt
number of scans: 8

freq. of 0 ppm: 399.780194 MHz
processed size: 16384 complex points
LB: 0.000 GF: 0.0000

SpinWorks 4: T Hurst TEH 312-853-1

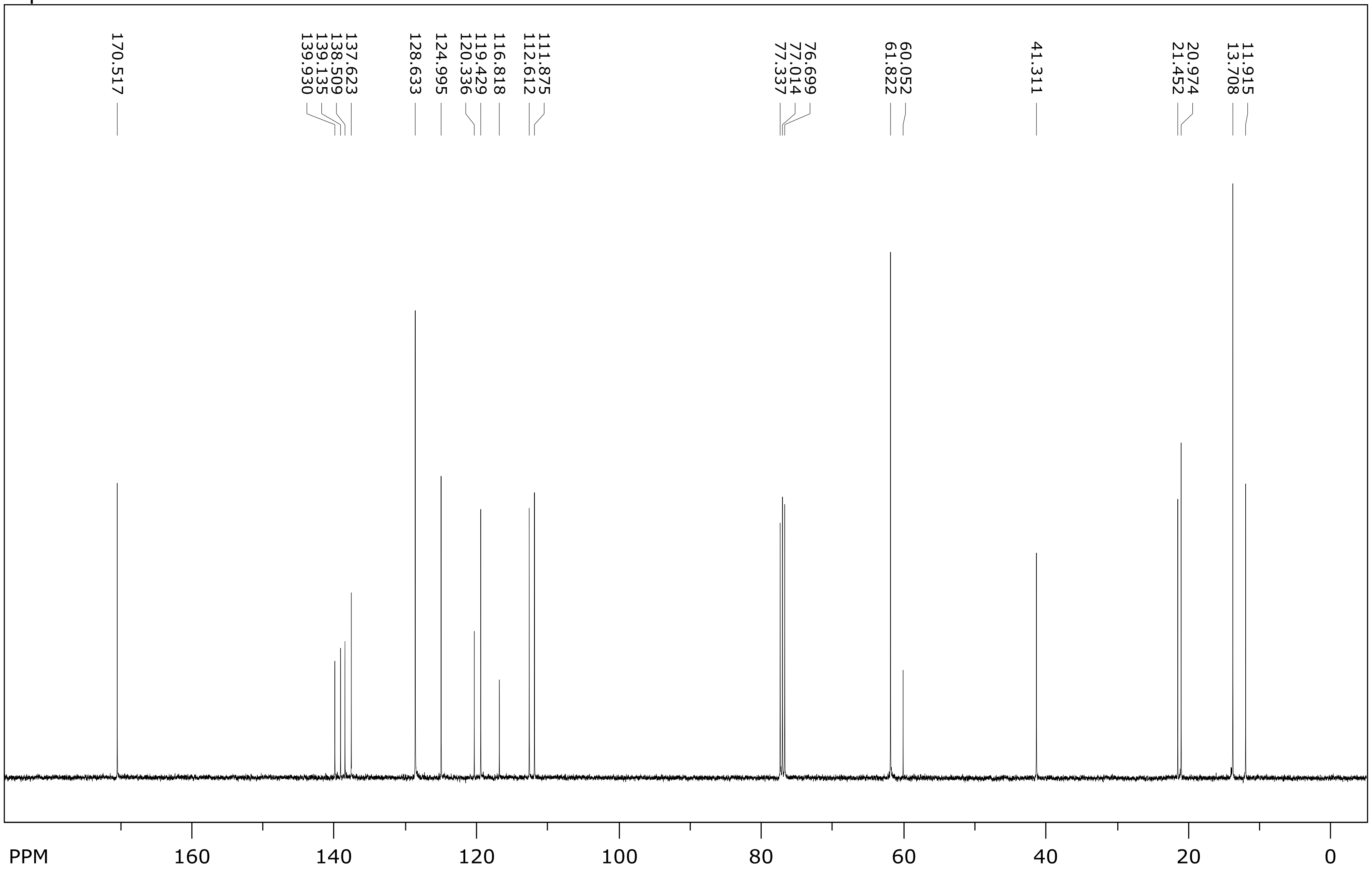

file: ...2-853-1\n7331teh_Carbon_ft-1-1.jdf expt: undefined
transmitter freq.: 100.525303 MHz
time domain size: 65536 points
width: 31407.04 Hz = 312.4292 ppm = 0.479233 Hz/pt
number of scans: 256

freq. of 0 ppm: 100.515273 MHz
processed size: 32768 complex points
LB: 0.000 GF: 0.0000

teh312

York - Chemistry - Mass Spectrometry Service Report

Analysis Information

Acquisition Date 21/07/2015 14:36:24

Analysis Filename	rjkt53686th_P1-E-4_01_59915.d
Method	400p_meoh1260_2c1s.m
Submission Name	rjkt53686th
Instrument	micrOTOF
ESI	Positive

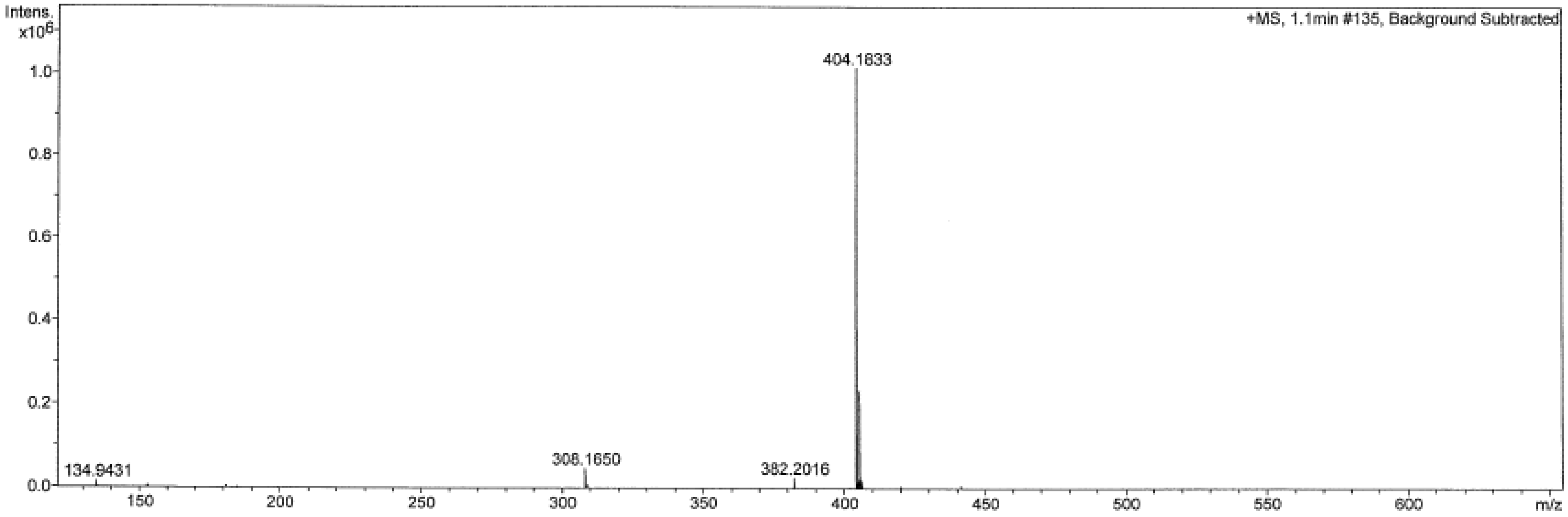

Meas. m/z	#	Formula	m/z	err [ppm]	err [mDa]	mSigma	Mean err [ppm]
382.2016	1	C 23 H 28 N O 4	382.2013	-0.9	-0.3	53.5	-0.6
404.1833	1	C 23 H 27 N Na O 4	404.1832	-0.2	-0.1	17.4	1.0

PerkinElmer Spectrum Version 10.03.07
01 July 2015 17:56

Analyst Administrator
Date 01 July 2015 17:56

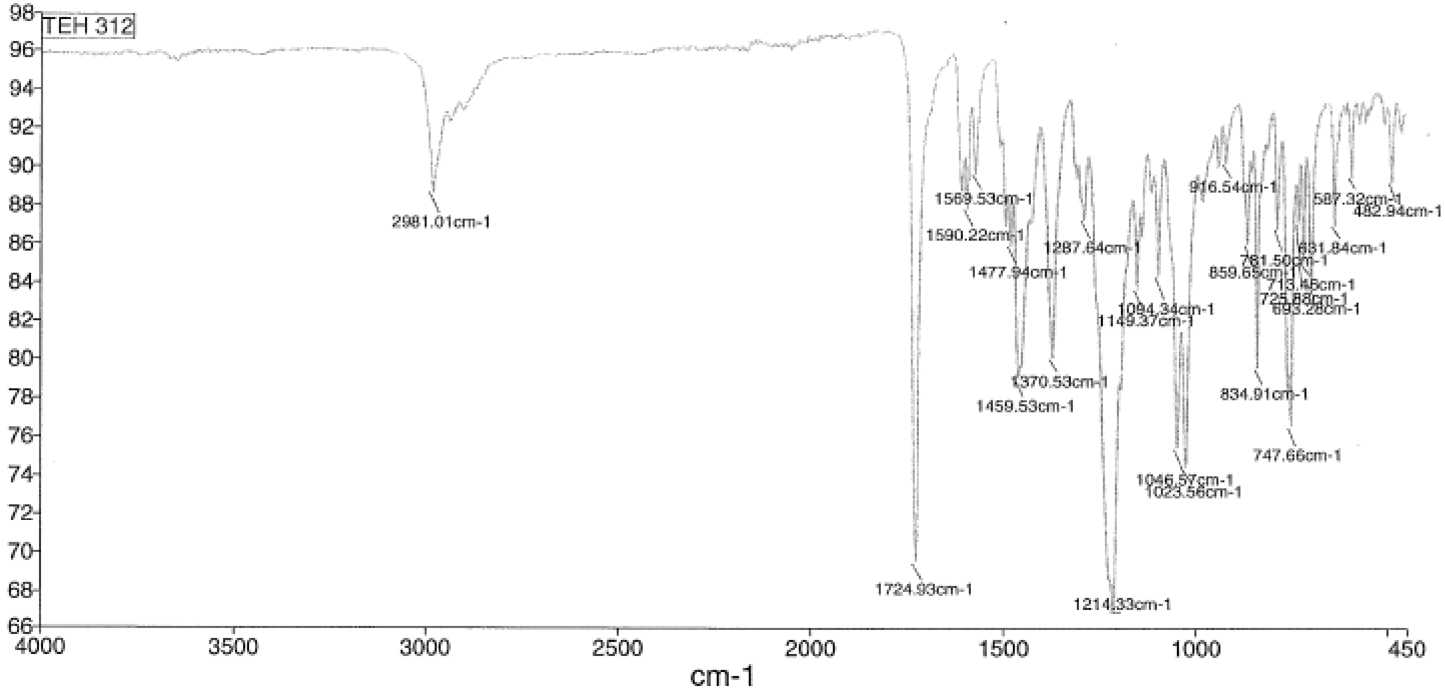

RJKT_01 07 2015_ 57 RJKT_01 07 2015_057

9,9-Diethyl 6-chloro-10-ethyl-2-methoxy-9,10-dihydroacridine-9,9-dicarboxylate 16j

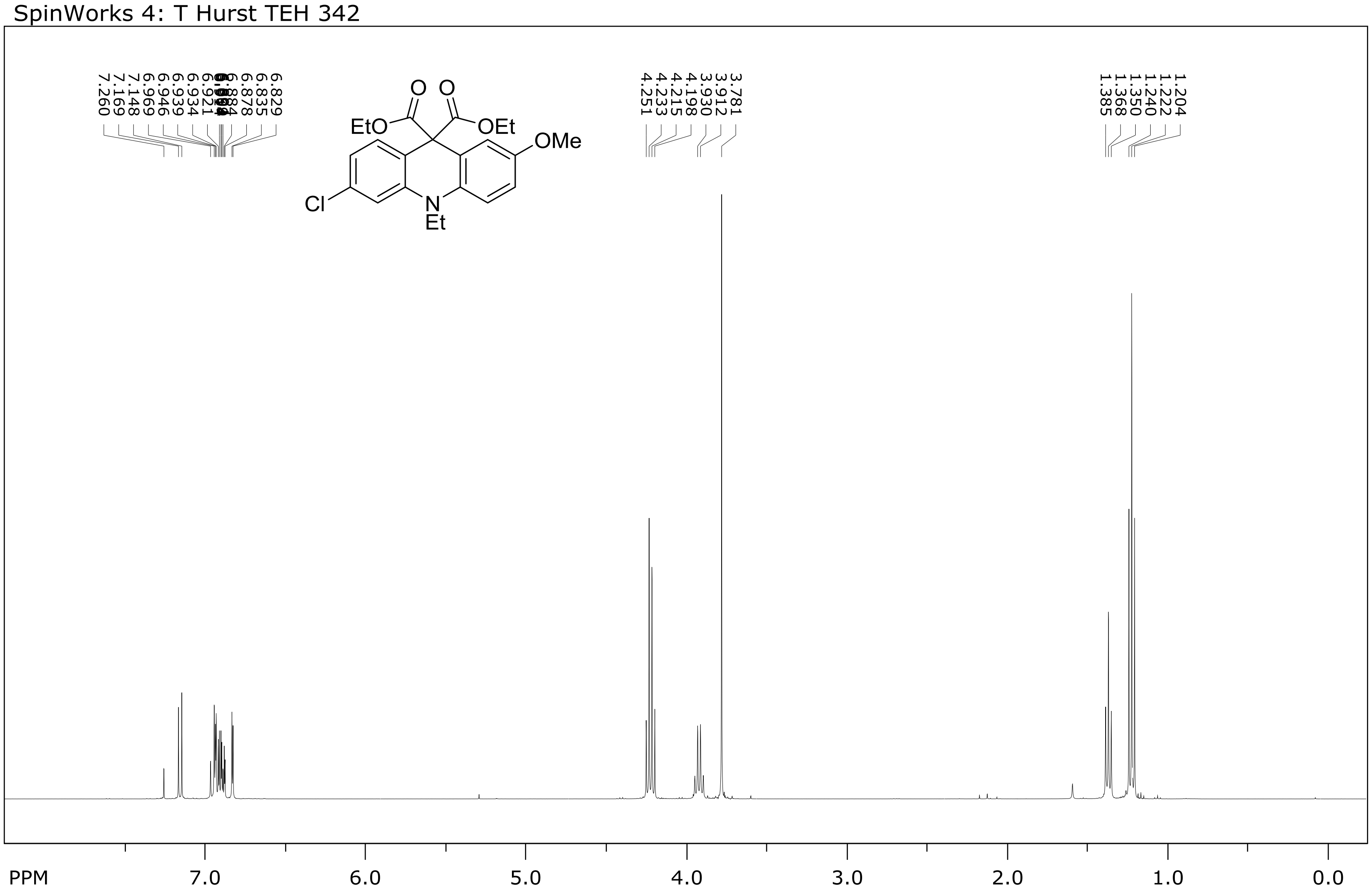

file: ...TEH 342\p6331teh_Proton_ft-1-1.jdf expt: undefined
transmitter freq.: 399.782198 MHz
time domain size: 32768 points
width: 7503.00 Hz = 18.7677 ppm = 0.228973 Hz/pt
number of scans: 8

freq. of 0 ppm: 399.780194 MHz
processed size: 16384 complex points
LB: 0.000 GF: 0.0000

SpinWorks 4: T Hurst TEH 342

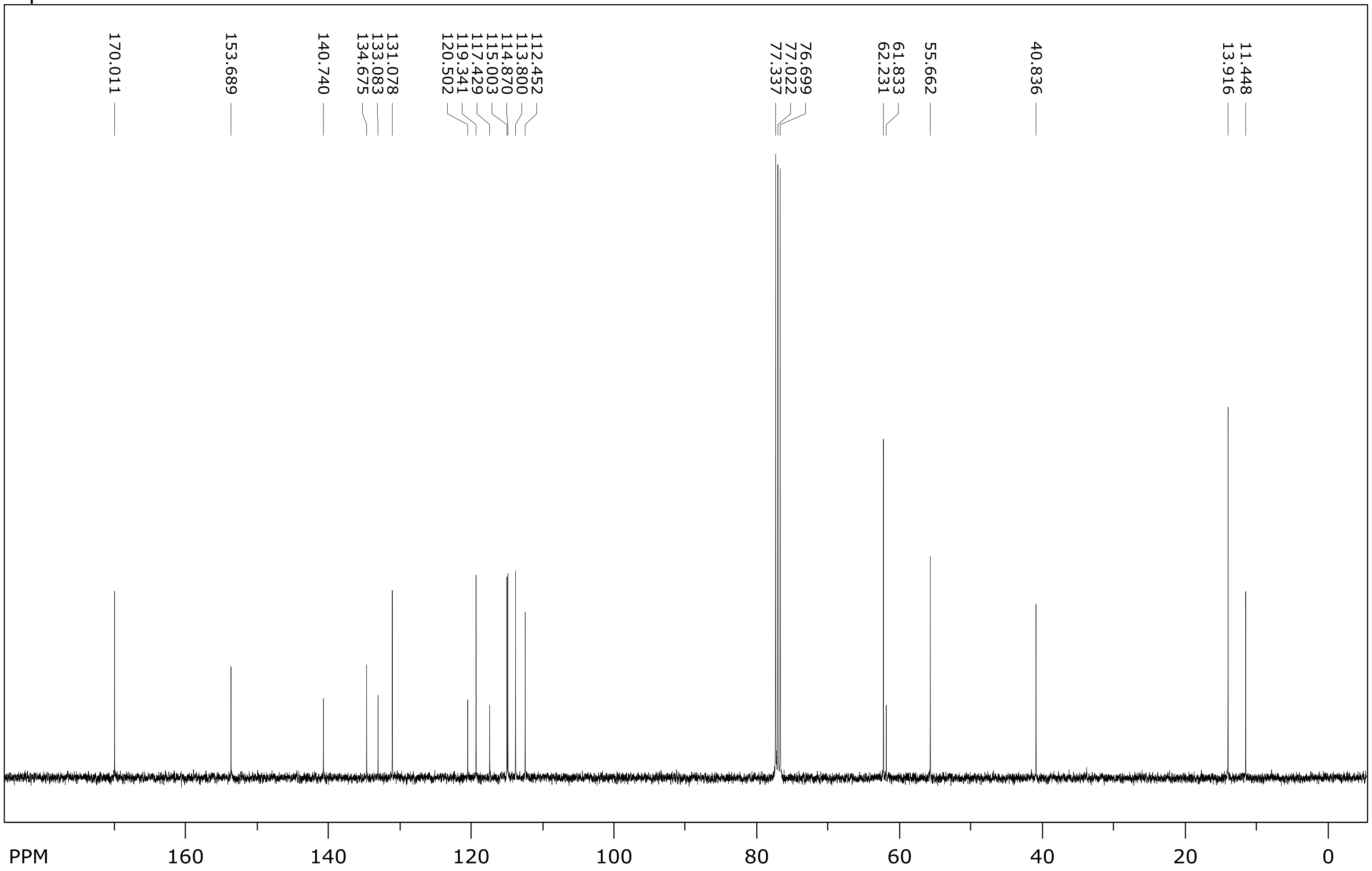

file: ...TEH 342\p6331teh_Carbon_ft-1-1.jdf expt: undefined
transmitter freq.: 100.525303 MHz
time domain size: 65536 points
width: 31407.04 Hz = 312.4292 ppm = 0.479233 Hz/pt
number of scans: 256

freq. of 0 ppm: 100.515261 MHz
processed size: 32768 complex points
LB: 0.000 GF: 0.0000

teh342

York - Chemistry - Mass Spectrometry Service Report

Analysis Information

Acquisition Date 21/07/2015 14:39:20

Analysis Filename	rjkt53687th_P1-E-5_01_59916.d
Method	600p_meoh1260_2c1s.m
Submission Name	rjkt53687th
Instrument	micrOTOF
ESI	Positive

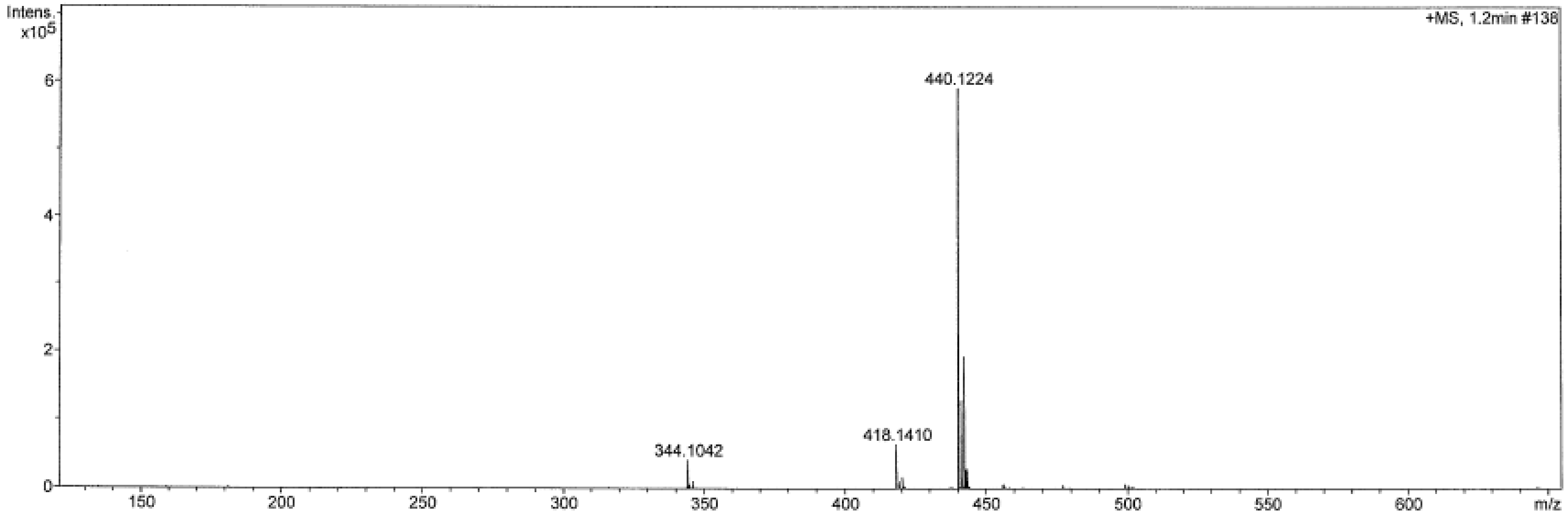

Meas. m/z	#	Formula	m/z	err [ppm]	err [mDa]	mSigma	Mean err [ppm]
418.1410	1	C 22 H 25 Cl N O 5	418.1416	1.4	0.6	62.5	1.3
440.1224	1	C 22 H 24 Cl N Na O 5	440.1235	2.5	1.1	22.9	2.5

PerkinElmer Spectrum Version 10.03.07
01 July 2015 17:58

Analyst Administrator
Date 01 July 2015 17:58

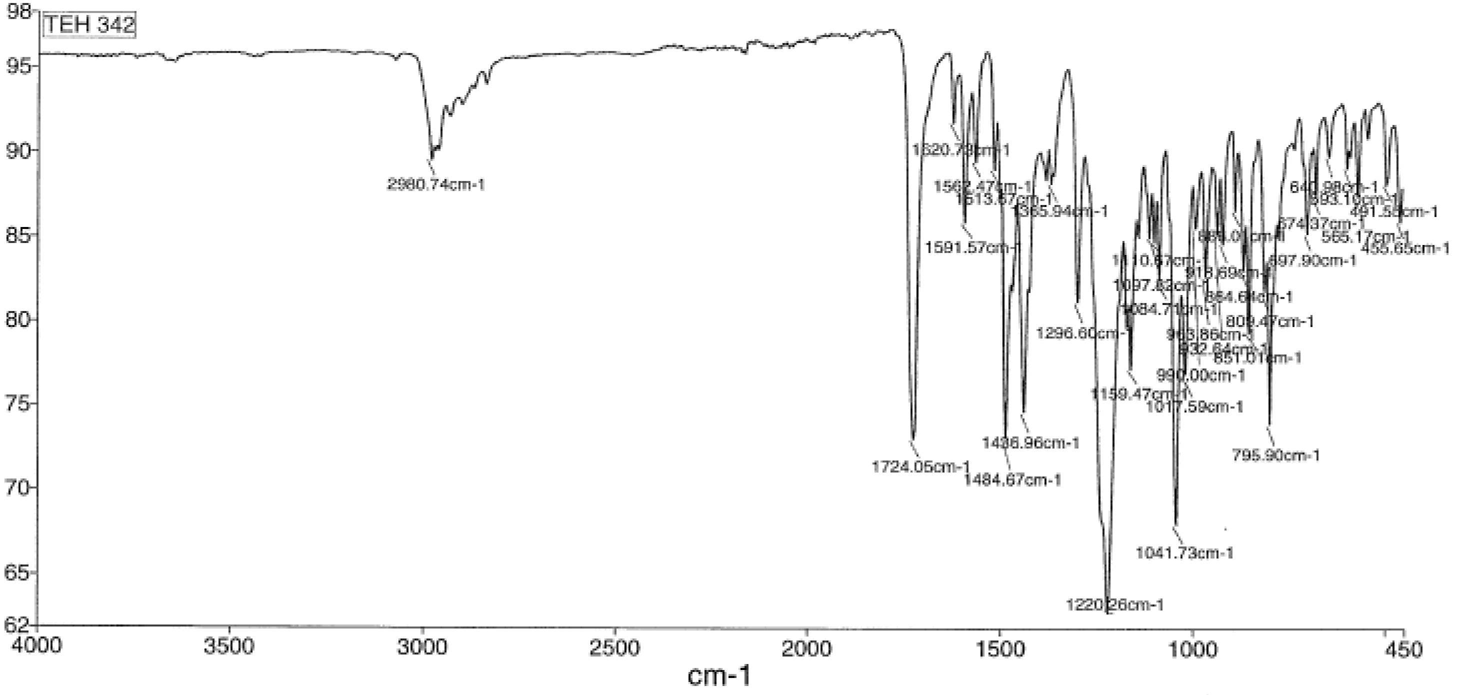

RJKT_01 07 2015_ 58 RJKT_01 07 2015_058

10,10-Diethyl 8-*tert*-butyl-5-methyl-5*H*,10*H*-benzo[*b*]1,5-naphthyridine-10,10-dicarboxylate 16k

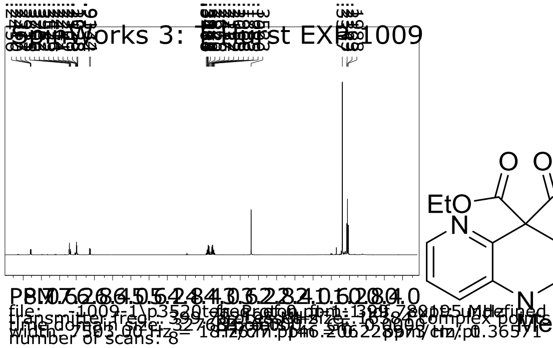

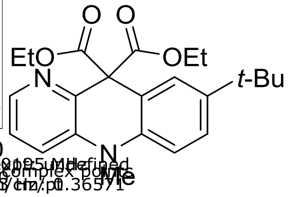

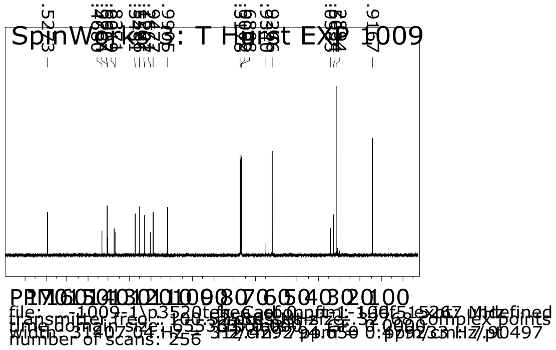
SpinWorks 3: T Hurst EXP 1009
number of scans: 256

teh368

York - Chemistry - Mass Spectrometry Service Report

Analysis Information Acquisition Date 14/10/2015 14:10:28

Analysis Filename	rjkt55053th_P1-E-8_01_61515.d
Method	600p_meoh1260_2c1s.m
Submission Name	rjkt55053th
Instrument	micrOTOF
ESI	Positive

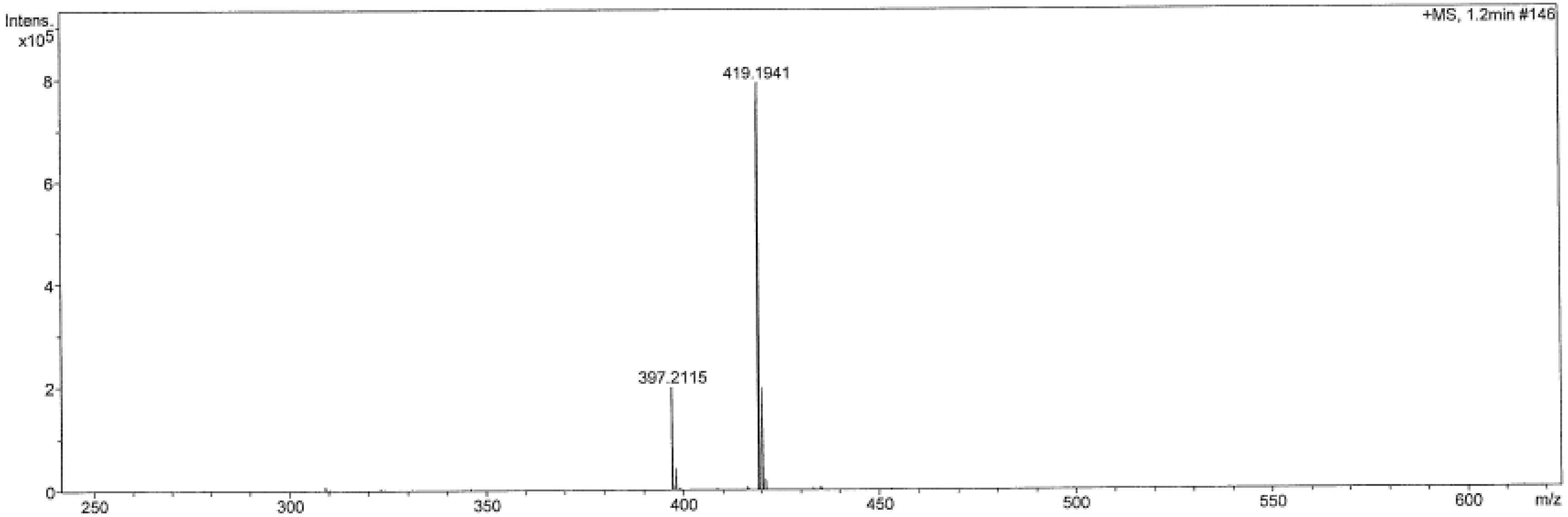

Meas. m/z	#	Formula	m/z	err [ppm]	err [mDa]	mSigma	Mean err [ppm]
397.2115	1	C 23 H 29 N 2 O 4	397.2122	1.7	0.7	30.8	2.2
419.1941	1	C 23 H 28 N 2 Na O 4	419.1941	0.1	0.1	8.7	1.0

PerkinElmer Spectrum Version 10.03.07
14 October 2015 21:21

Analyst PEService
Date 14 October 2015 21:21

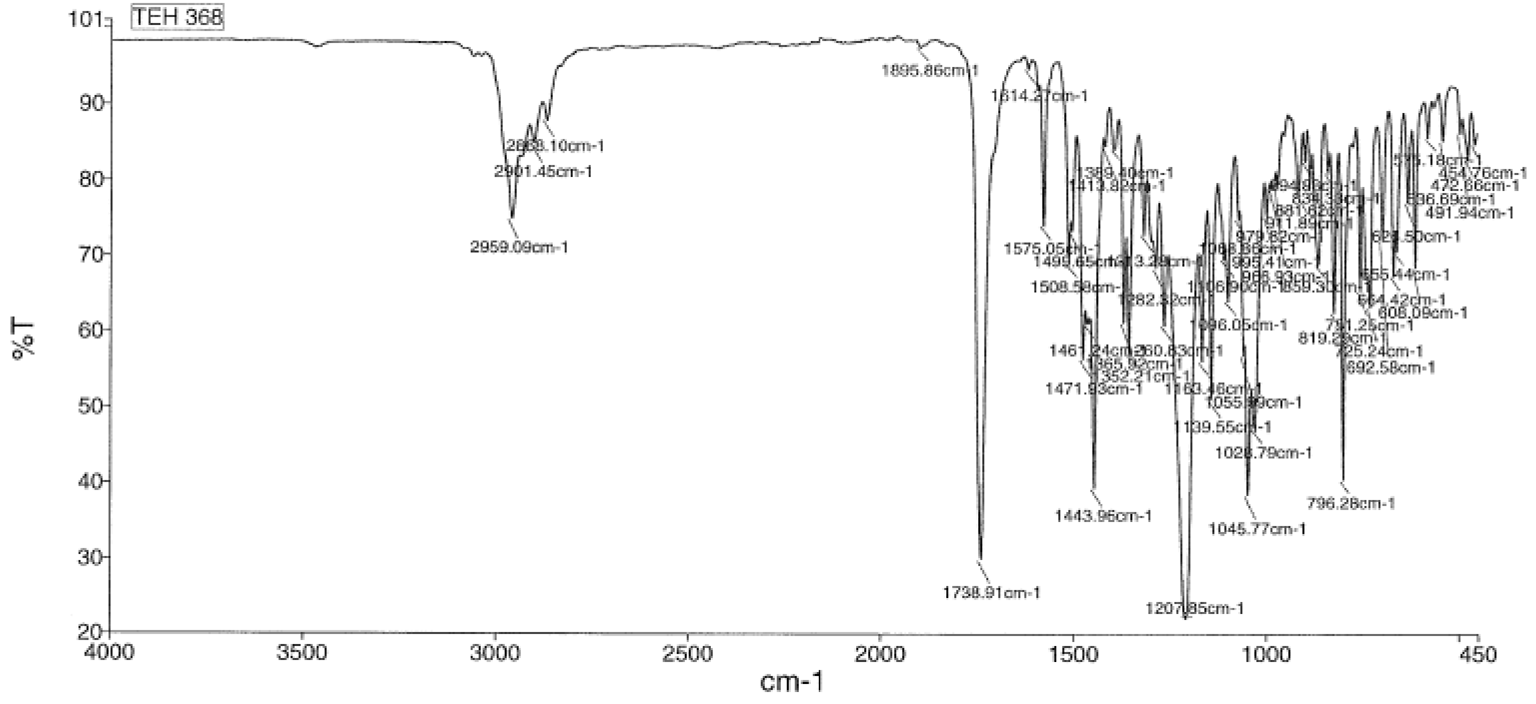

PEService 32 Sample 032 By PEService Date Wednesday, October 14 2015

9,9-Diethyl 9*H*-xanthene-9,9-dicarboxylate 16l

SpinWorks 4: T Hurst EXP 906A DIPEA

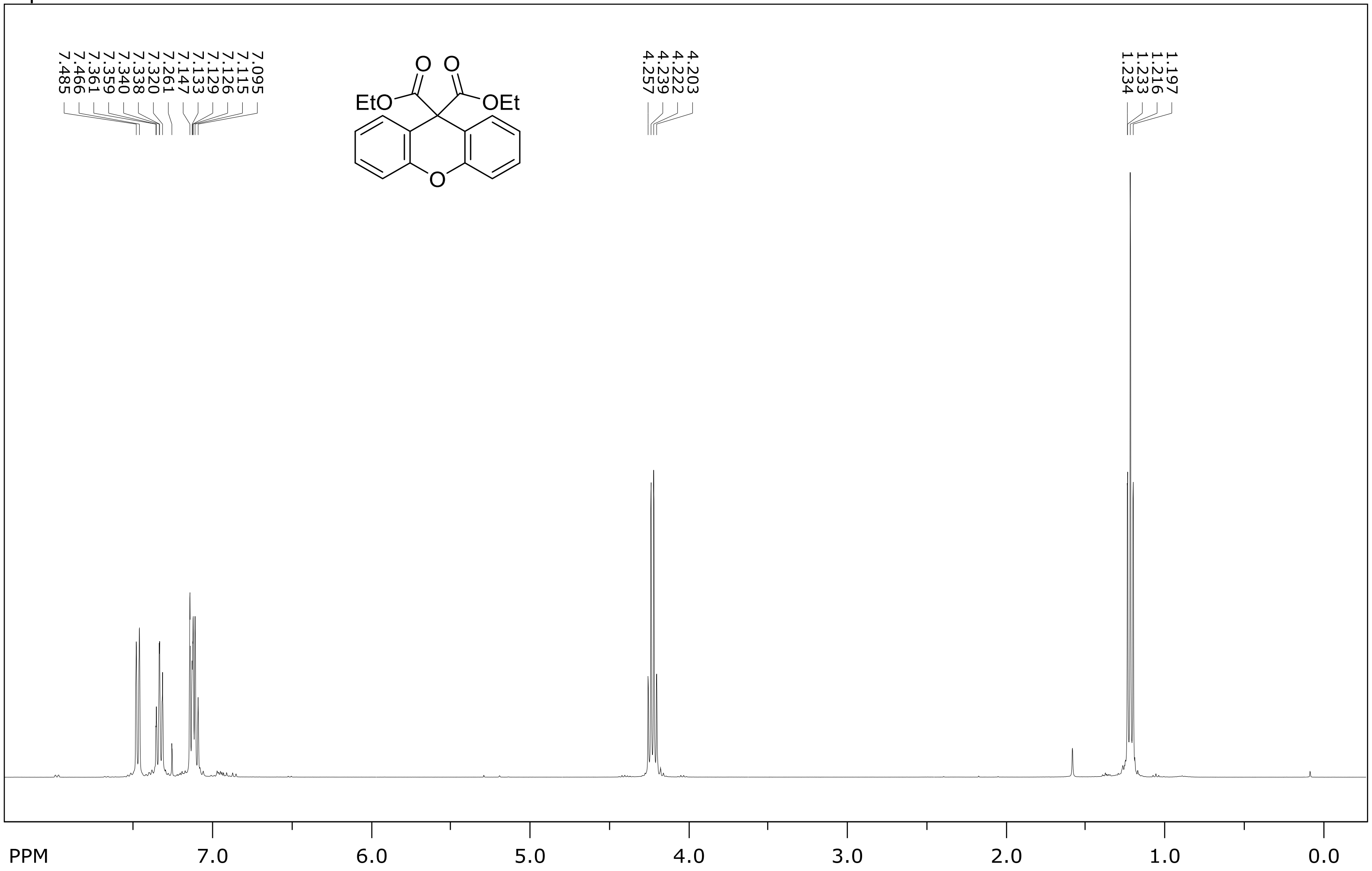

file: ...6-906-1\n9610teh_Proton_ft-1-1.jdf expt: undefined
transmitter freq.: 399.782198 MHz
time domain size: 32768 points
width: 7503.00 Hz = 18.7677 ppm = 0.228973 Hz/pt
number of scans: 8

freq. of 0 ppm: 399.780194 MHz
processed size: 16384 complex points
LB: 0.000 GF: 0.0000

SpinWorks 4: T Hurst EXP 906A DIPEA

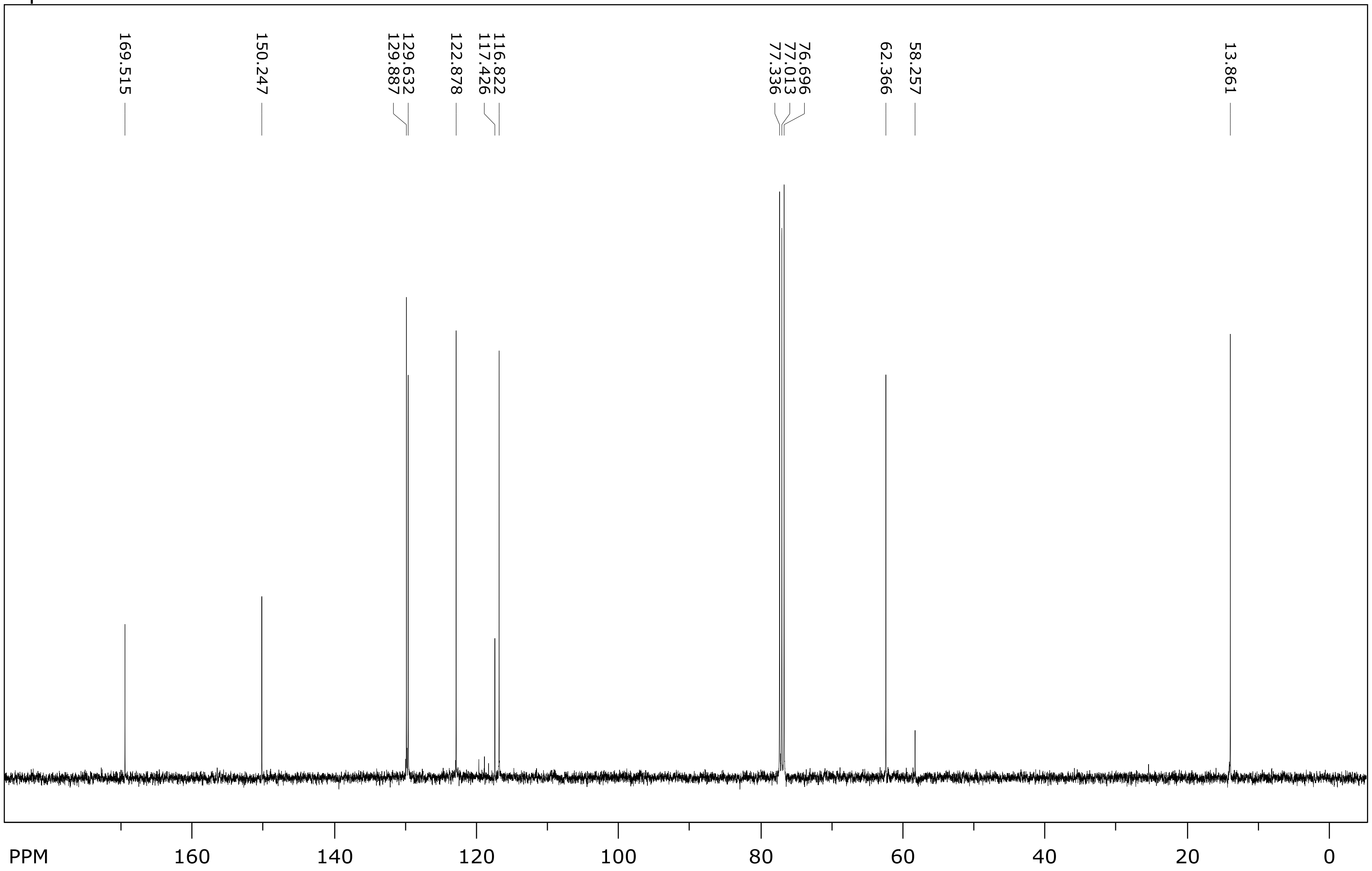

file: ...6-906-1\n9610teh_Carbon_ft-1-1.jdf expt: undefined
transmitter freq.: 100.525303 MHz
time domain size: 65536 points
width: 31407.04 Hz = 312.4292 ppm = 0.479233 Hz/pt
number of scans: 256

freq. of 0 ppm: 100.515261 MHz
processed size: 32768 complex points
LB: 0.000 GF: 0.0000

teh326

York - Chemistry - Mass Spectrometry Service Report

Analysis Information

Acquisition Date 10/08/2015 16:30:18

Analysis Filename	rjkt54046th_P1-B-3_01_60279.d
Method	400p_meoh1260_2c1s.m
Submission Name	rjkt54046th
Instrument	micrOTOF
ESI	Positive

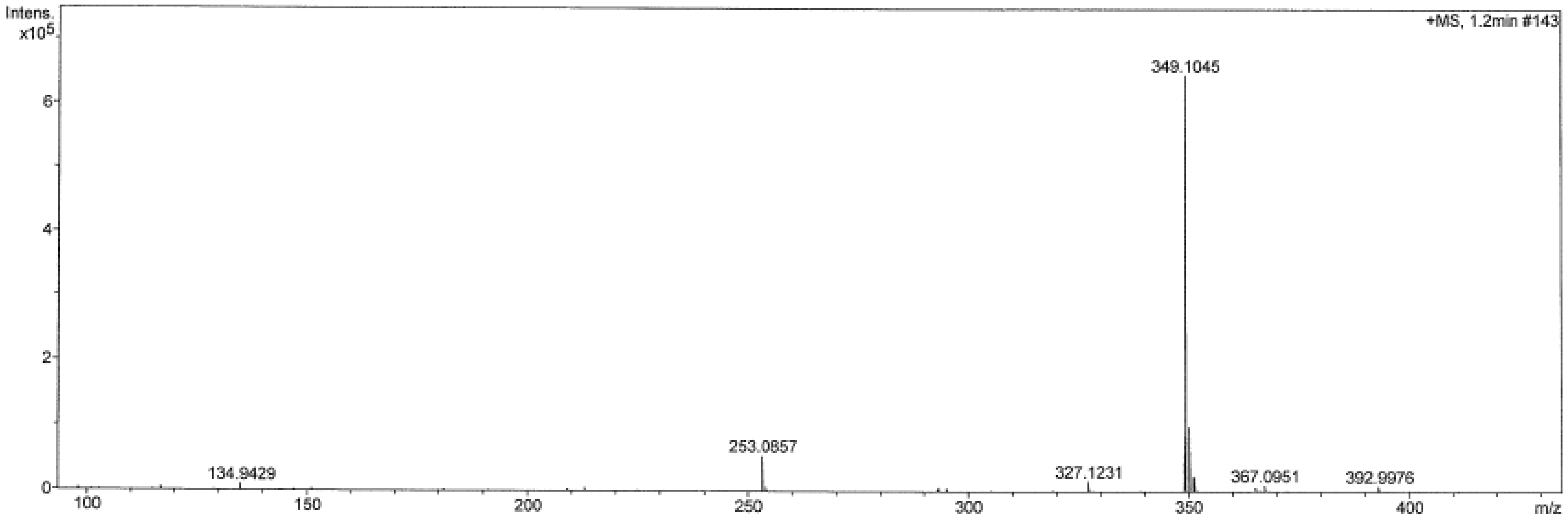

Meas. m/z	#	Formula	m/z	err [ppm]	err [mDa]	mSigma	Mean err [ppm]
327.1231	1	C 19 H 19 O 5	327.1227	-1.1	-0.4	34.9	-1.7
349.1045	1	C 19 H 18 Na O 5	349.1046	0.5	0.2	30.6	0.1

PerkinElmer Spectrum Version 10.03.07
10 August 2015 16:43

Analyst Administrator
Date 10 August 2015 16:43

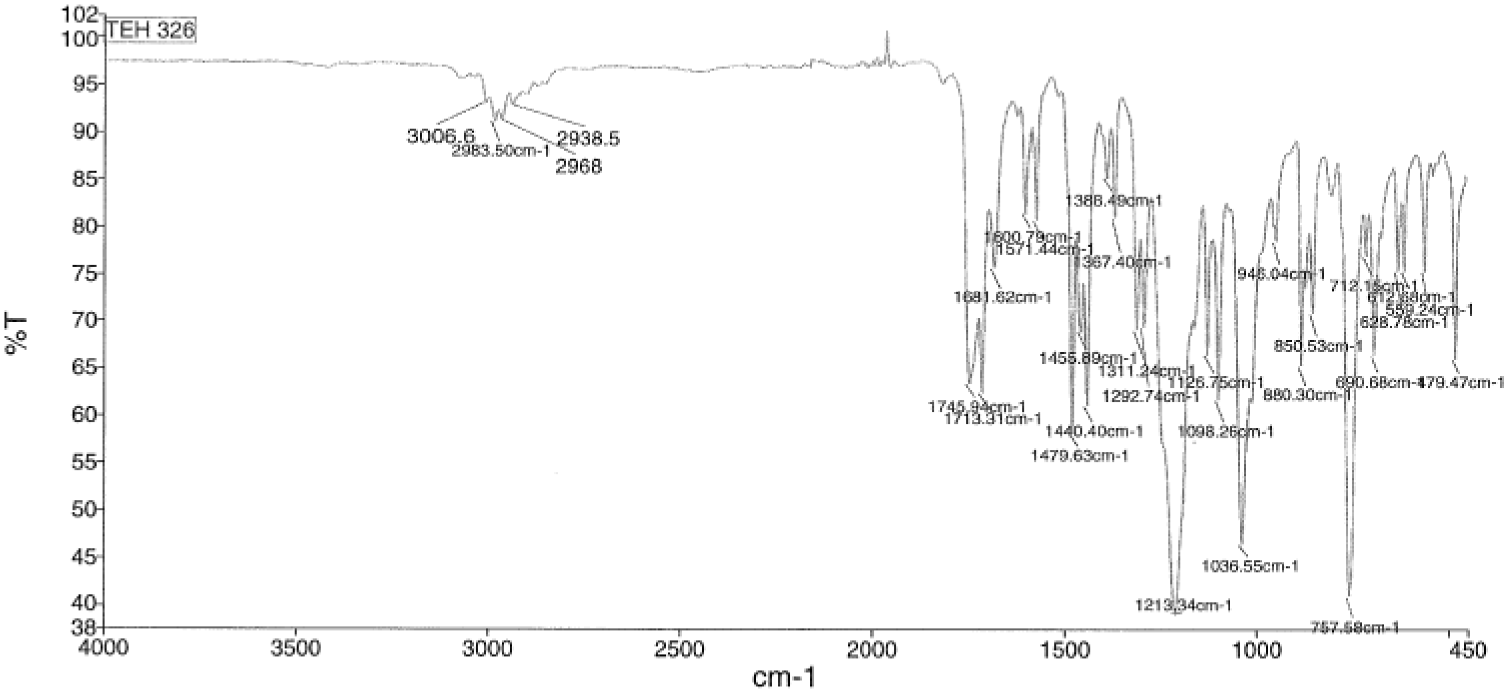

9,9-Diethyl 9*H*-thioxanthene-9,9-dicarboxylate 16m

SpinWorks 4: T Hurst TEH 346-931-1

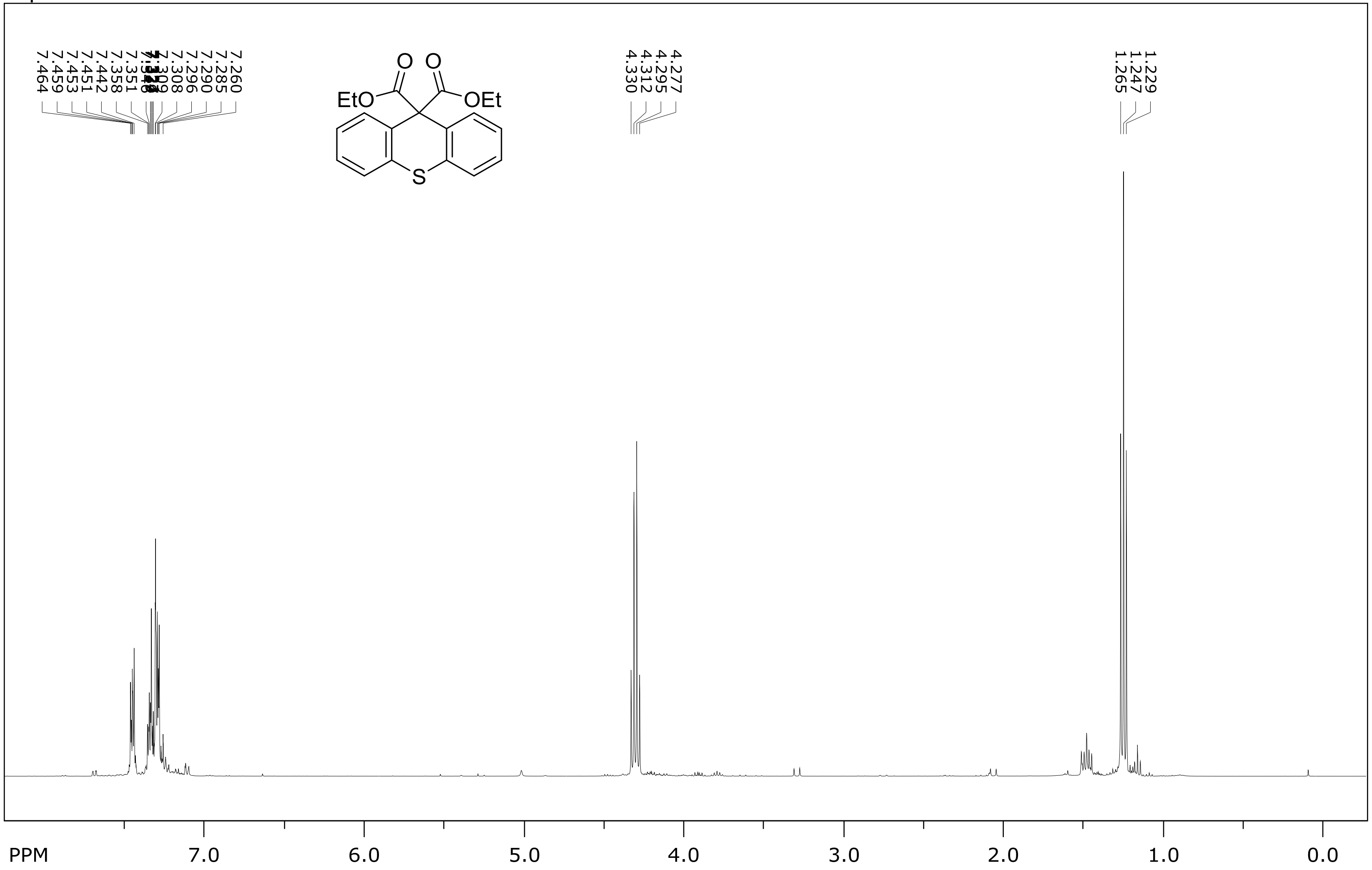

file: ...6-931-1\p0421teh_Proton_ft-1-1.jdf   expt: undefined
transmitter freq.: 399.782198 MHz
time domain size: 32768 points
width: 7503.00 Hz = 18.7677 ppm = 0.228973 Hz/pt
number of scans: 8

freq. of 0 ppm: 399.780195 MHz
processed size: 16384 complex points
LB: 0.000   GF: 0.0000

SpinWorks 4: T Hurst TEH 346-931-1

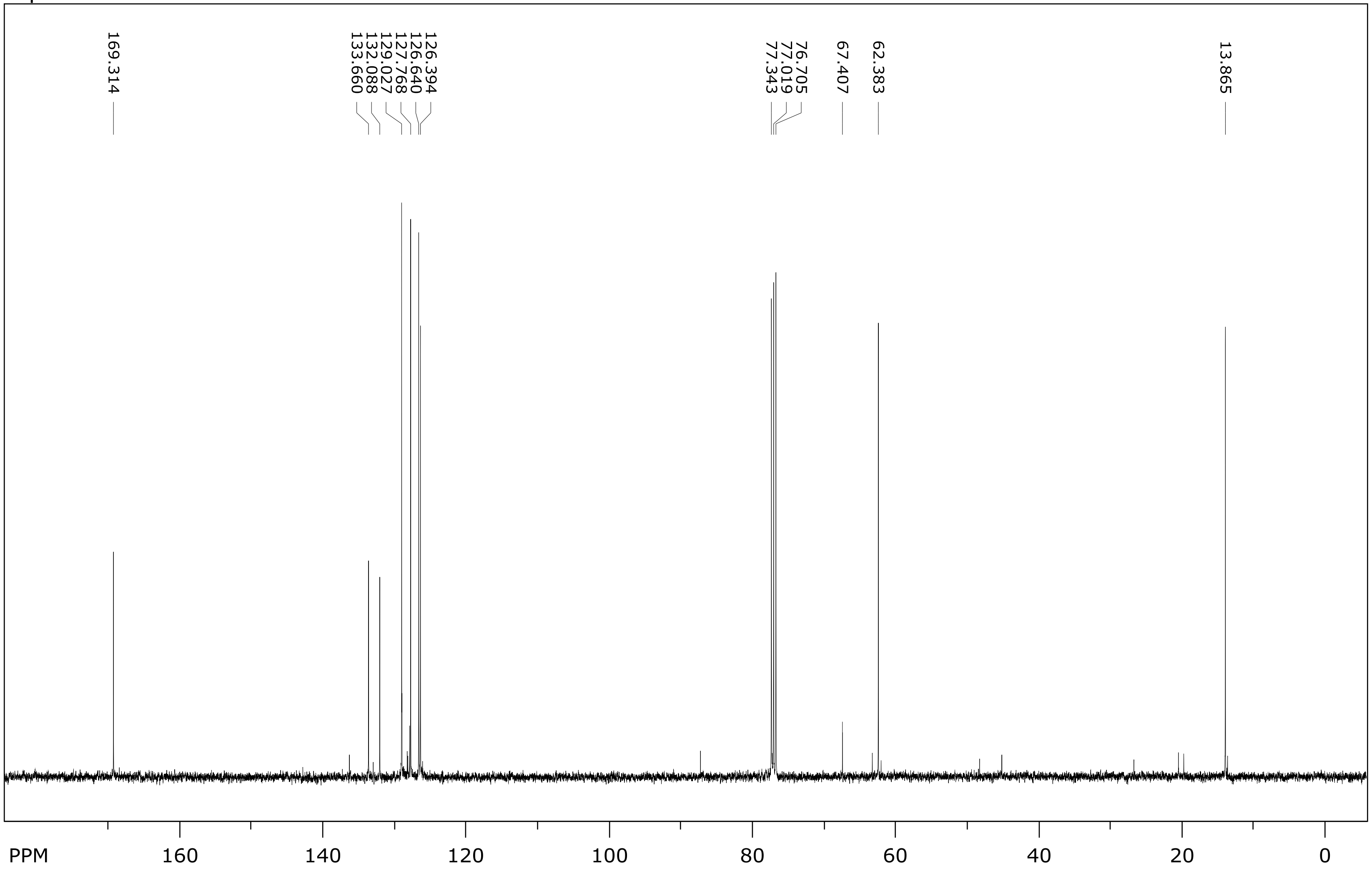

file: ...6-931-1\p0421teh_Carbon_ft-1-1.jdf expt: undefined
transmitter freq.: 100.525303 MHz
time domain size: 65536 points
width: 31407.04 Hz = 312.4292 ppm = 0.479233 Hz/pt
number of scans: 256

freq. of 0 ppm: 100.515264 MHz
processed size: 32768 complex points
LB: 0.000 GF: 0.0000

teh346

York - Chemistry - Mass Spectrometry Service Report

Analysis Information

Acquisition Date 10/08/2015 16:34:24

Analysis Filename	rjkt54047th_P1-B-4_01_60280.d
Method	400p_meoh1260_2c1s.m
Submission Name	rjkt54047th
Instrument	micrOTOF
ESI	Positive

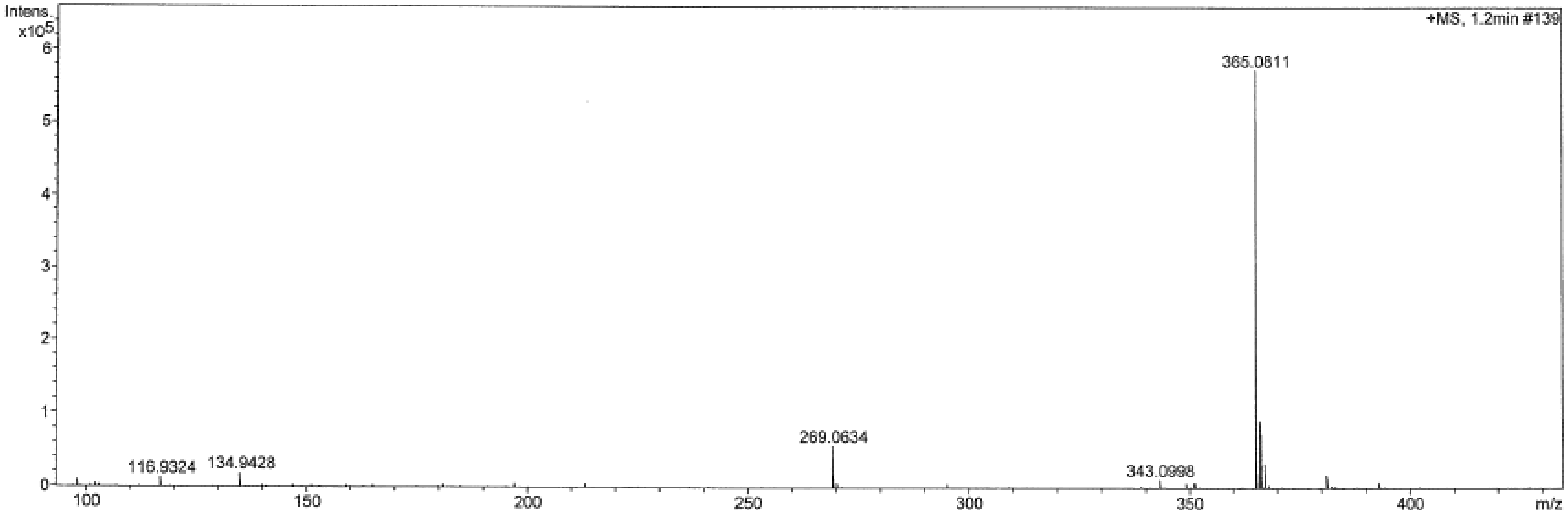

Meas. m/z	#	Formula	m/z	err [ppm]	err [mDa]	mSigma	Mean err [ppm]
343.0998	1	C 19 H 19 O 4 S	343.0999	0.2	0.1	44.9	1.5
365.0811	1	C 19 H 18 Na O 4 S	365.0818	2.0	0.7	28.5	0.9

PerkinElmer Spectrum Version 10.03.07
10 August 2015 16:45

Analyst Administrator
Date 10 August 2015 16:45

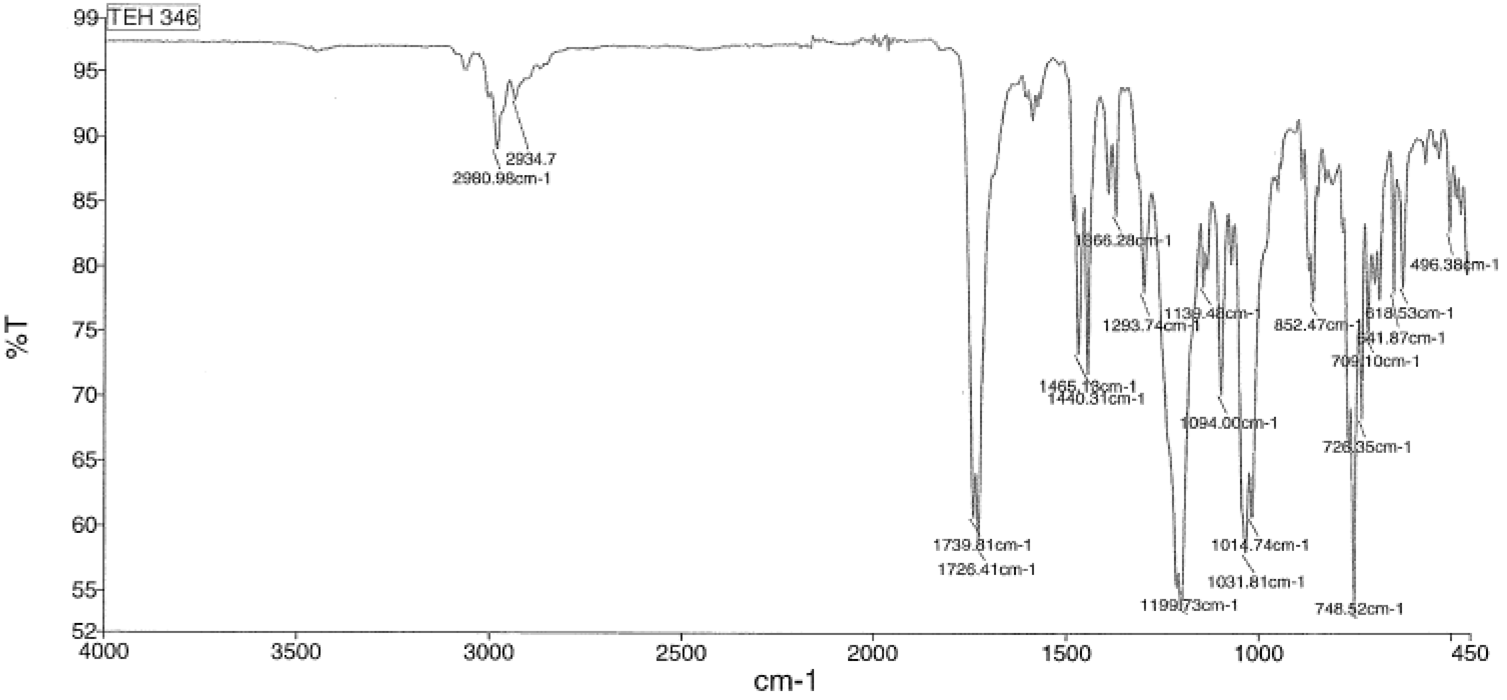

10-Ethyl-9,10-dihydroacridine-9-carboxylic acid 17

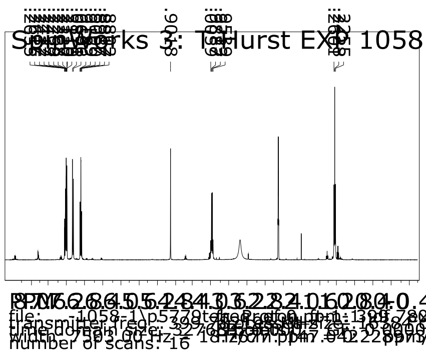

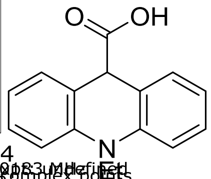

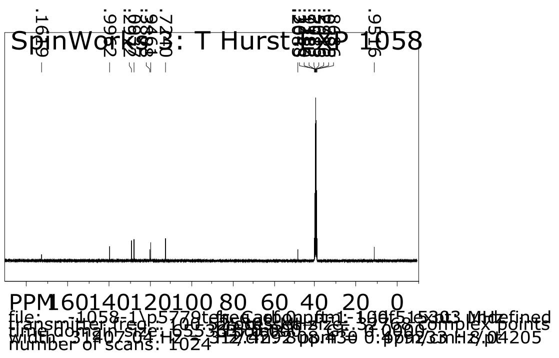

PPM 160 140 120 100 80 60 40 20 0

teh395

York - Chemistry - Mass Spectrometry Service Report

Analysis Information

Acquisition Date 15/10/2015 11:25:35

Analysis Filename	rjkt55066th_P1-B-4_01_61541.d
Method	400p_meoh1260_2c1s.m
Submission Name	rjkt55066th
Instrument	micrOTOF
ESI	Positive

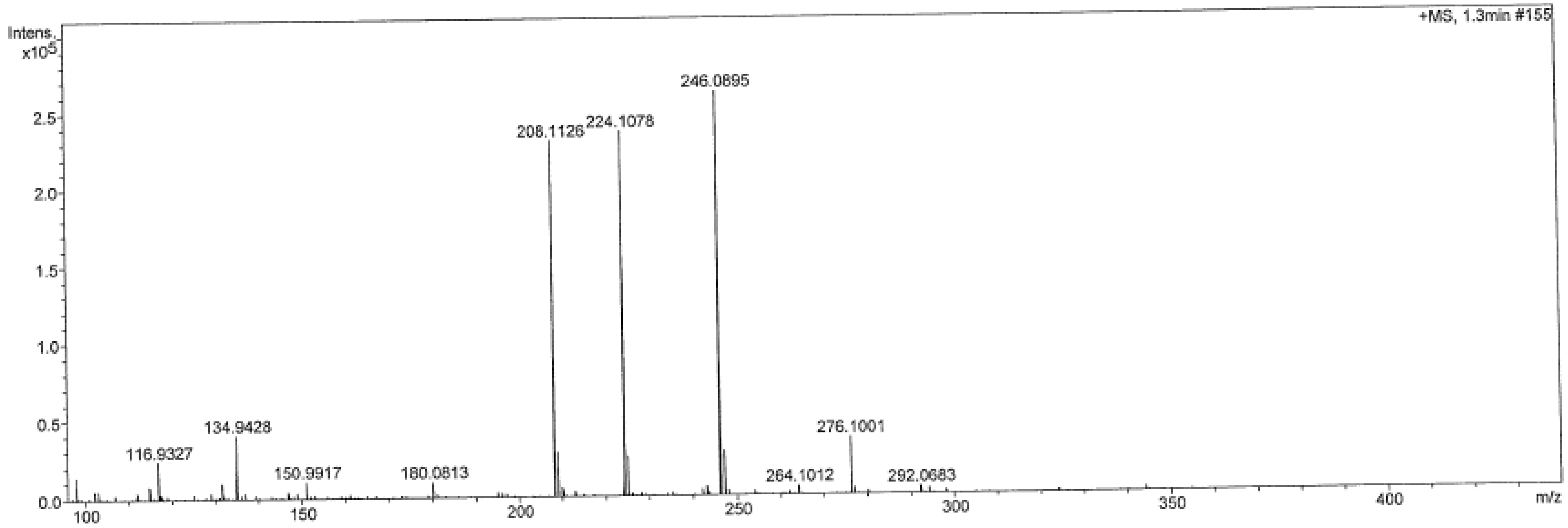

Meas. m/z	#	Formula	m/z	err [ppm]	err [mDa]	mSigma	Mean err [ppm]
276.1001	1	C 16 H 15 N Na O 2	276.0995	-2.3	-0.6	37.1	-2.2

PerkinElmer Spectrum Version 10.03.07
14 October 2015 21:25

Analyst PEService
Date 14 October 2015 21:25

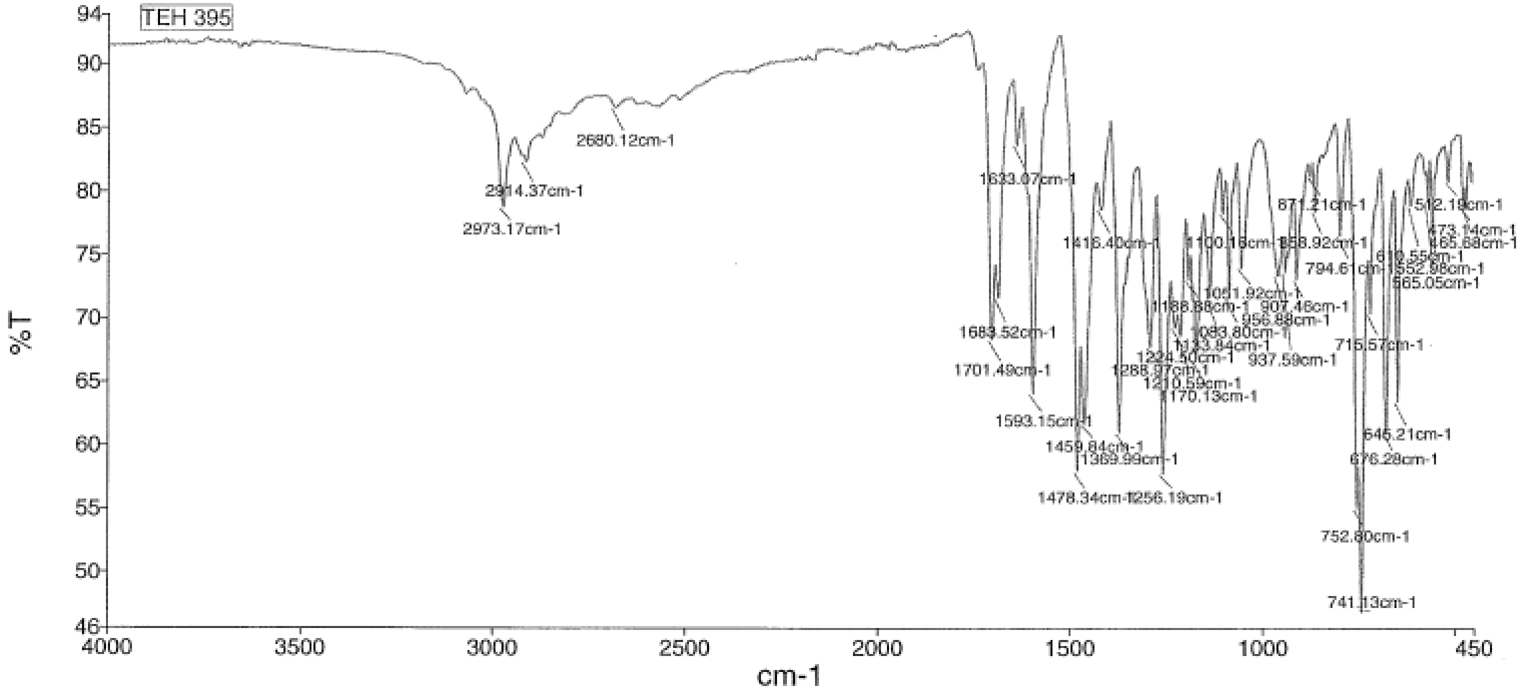

PEService 33 Sample 033 By PEService Date Wednesday, October 14 2015

10-Ethyl-9,9-di(hydroxymethyl)-9,10-dihydroacridine 18

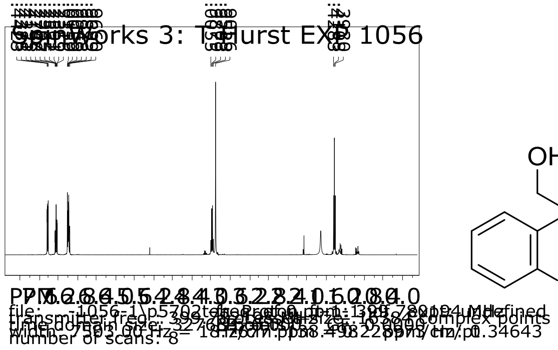

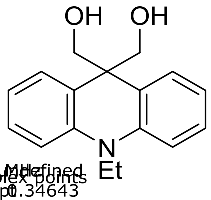

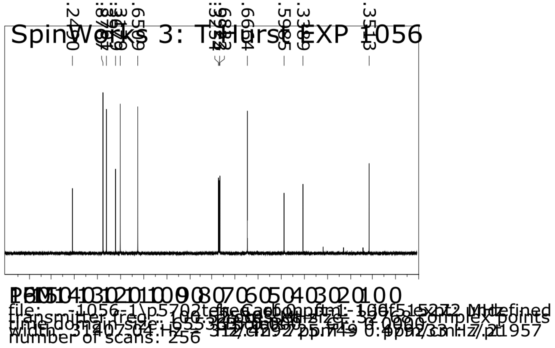
SpinWorks 3: T. Hurst EXP 1056
number of scans: 256

teh399

York - Chemistry - Mass Spectrometry Service Report

Analysis Information

Acquisition Date 15/10/2015 11:28:30

Analysis Filename	rjkt55067th_P1-B-5_01_61542.d
Method	400p_meoh1260_2c1s.m
Submission Name	rjkt55067th
Instrument	micrOTOF
ESI	Positive

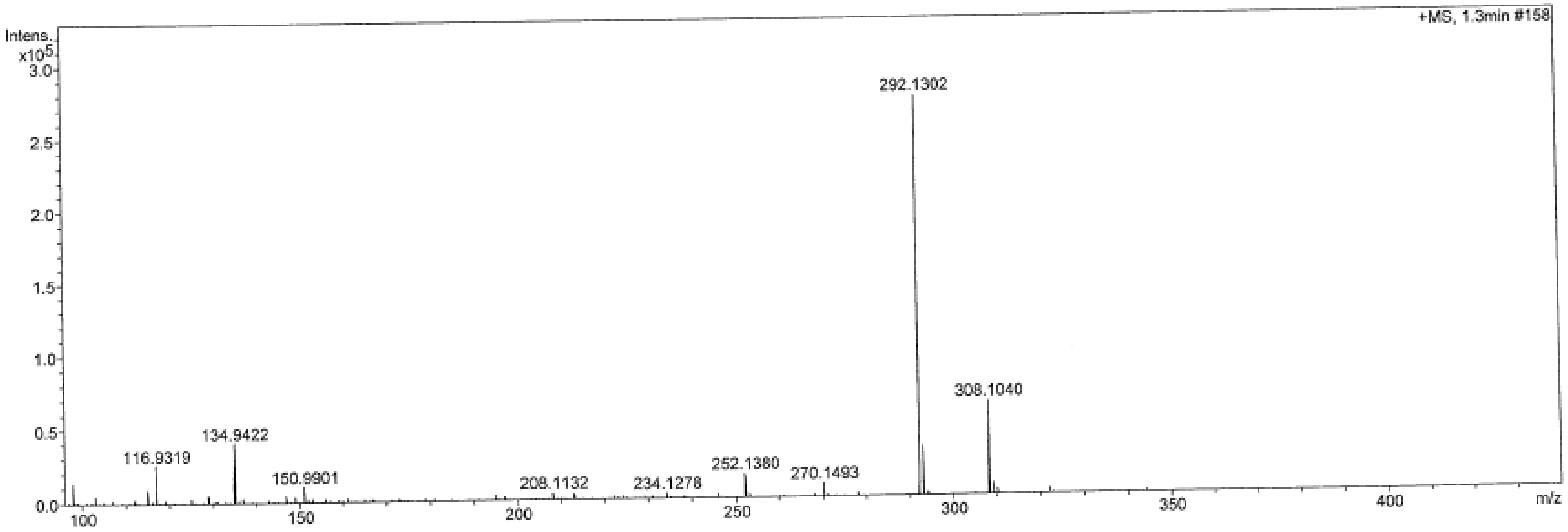

Meas. m/z	#	Formula	m/z	err [ppm]	err [mDa]	mSigma	Mean err [ppm]
270.1493	1	C 17 H 20 N O 2	270.1489	-1.8	-0.5	15.5	-0.2
292.1302	1	C 17 H 19 N Na O 2	292.1308	2.2	0.6	39.4	1.9

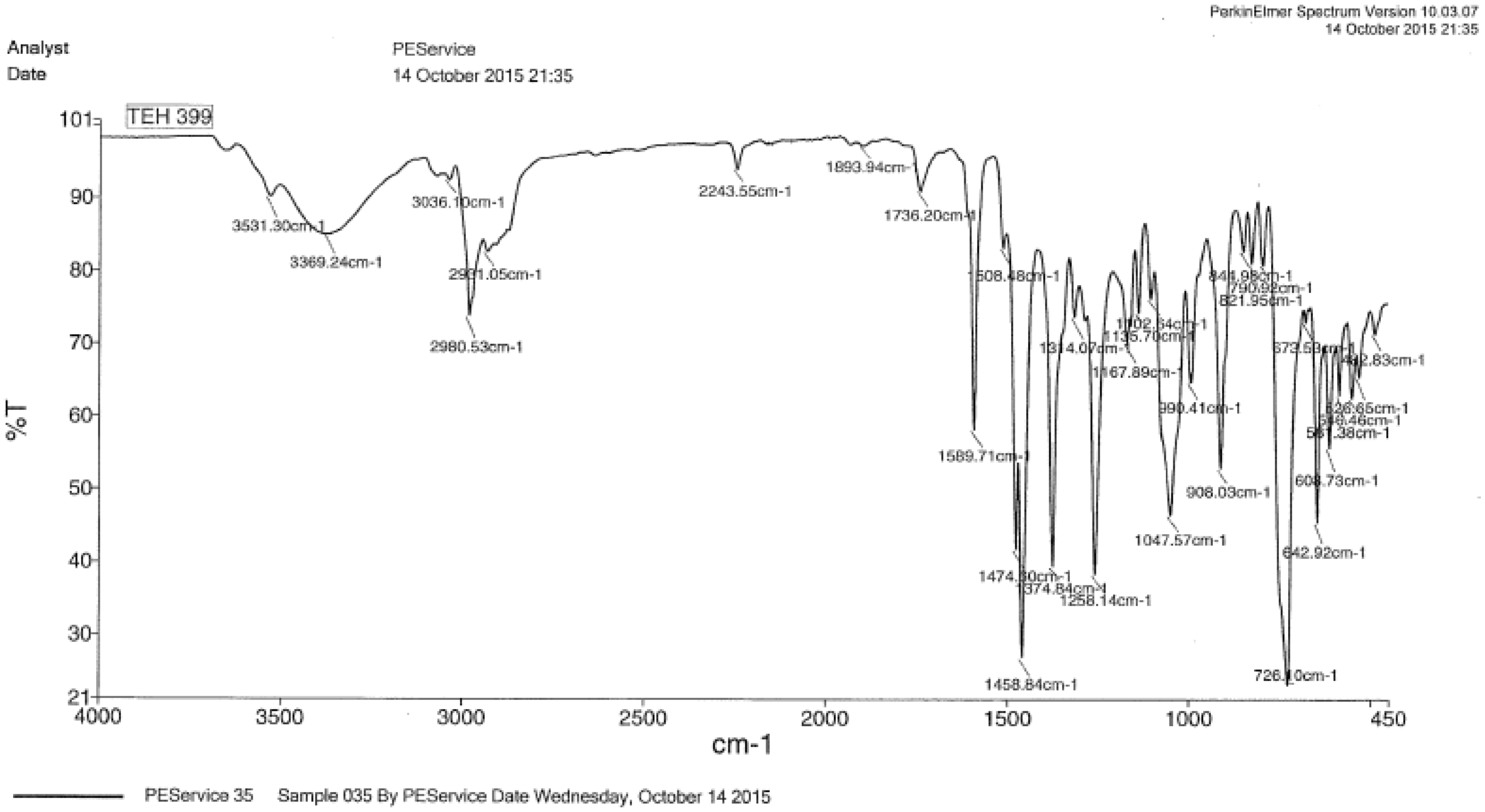
PerkinElmer Spectrum Version 10.03.07
14 October 2015 21:35
Analyst
Date
PEService
14 October 2015 21:35
TEH 399
%T
cm-1
3531.30cm-1
3369.24cm-1
3036.10cm-1
2980.53cm-1
2243.55cm-1
1893.94cm-1
1736.20cm-1
1589.71cm-1
1474.30cm-1
1458.84cm-1
1374.84cm-1
1258.14cm-1
1167.89cm-1
1047.57cm-1
908.03cm-1
790.92cm-1
821.95cm-1
726.10cm-1
642.92cm-1
608.73cm-1
526.65cm-1
561.38cm-1
PEService 35 Sample 035 By PEService Date Wednesday, October 14 2015

Ethyl 10-ethyl-9-(prop-2-en-1-yl)-9,10-dihydroacridine-9-carboxylate 19

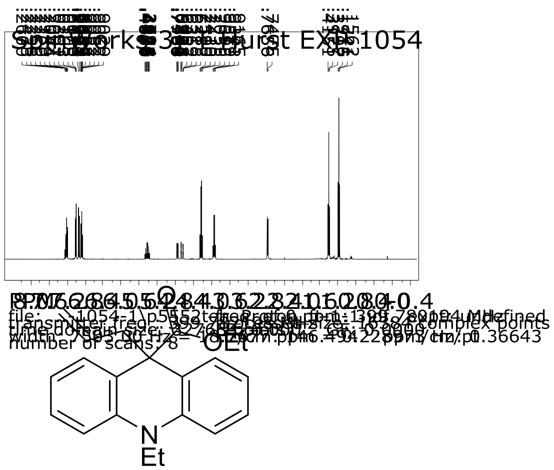

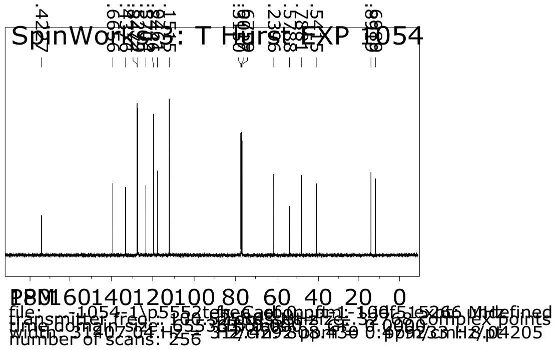
SpinWorks 3: T Hurst-EXP 1054
PPM
number of scans: 256

teh390

York - Chemistry - Mass Spectrometry Service Report

Analysis Information

Acquisition Date 15/10/2015 11:22:39

Analysis Filename	rjkt55065th_P1-B-3_01_61540.d
Method	400p_meoh1260_2c1s.m
Submission Name	rjkt55065th
Instrument	micrOTOF
ESI	Positive

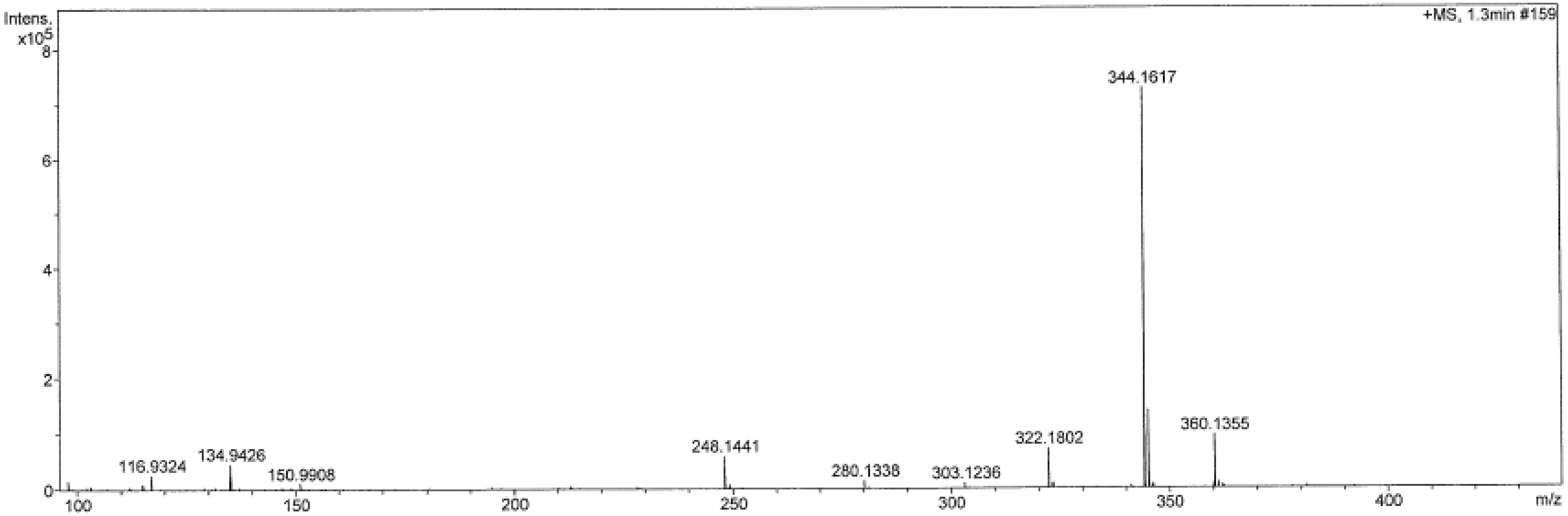

Meas. m/z	#	Formula	m/z	err [ppm]	err [mDa]	mSigma	Mean err [ppm]
322.1802	1	C 21 H 24 N O 2	322.1802	-0.2	-0.1	53.4	-0.6
344.1617	1	C 21 H 23 N Na O 2	344.1621	1.2	0.4	24.6	0.9

Analyst PEService
Date 14 October 2015 21:38

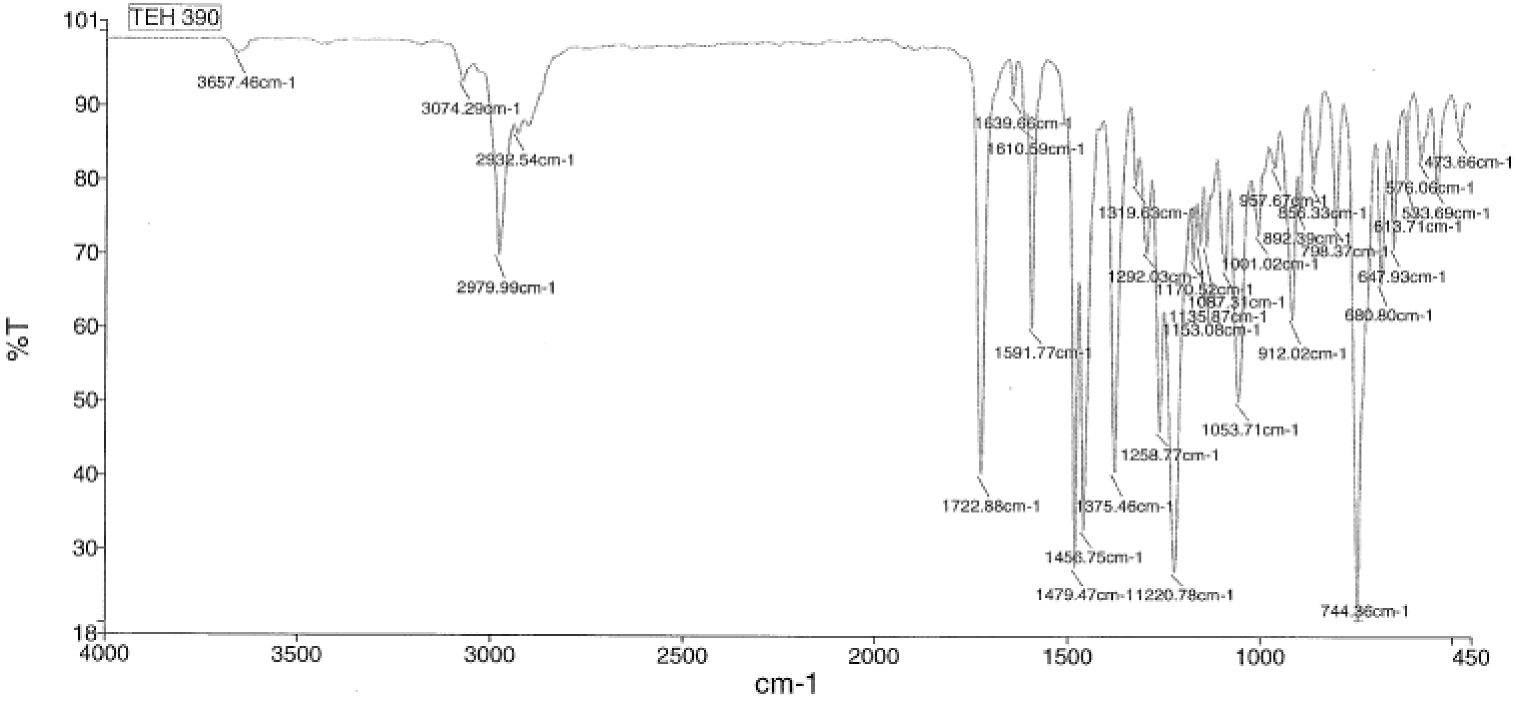

PEService 36 Sample 036 By PEService Date Wednesday, October 14 2015

9,9-Diethyl 9,10-dihydroacridine-9,9-dicarboxylate 20

SpinWorks 4: T Hurst EXP 1074 F53-61

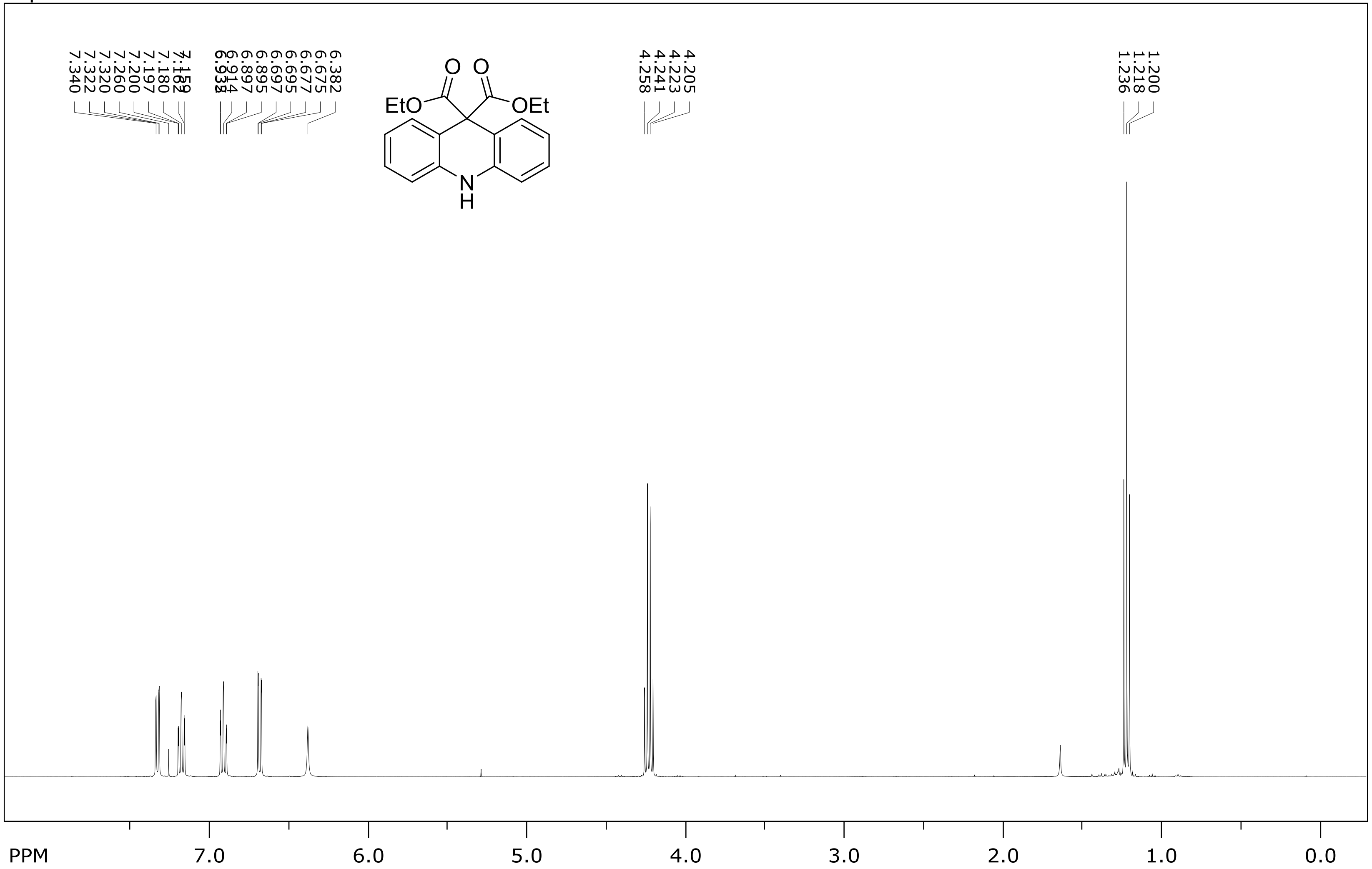

file: ...-1074-1\p6370teh_Proton_ft-1-1.jdf expt: undefined
transmitter freq.: 399.782198 MHz
time domain size: 32768 points
width: 7503.00 Hz = 18.7677 ppm = 0.228973 Hz/pt
number of scans: 8

freq. of 0 ppm: 399.780194 MHz
processed size: 16384 complex points
LB: 0.000 GF: 0.0000

SpinWorks 4: T Hurst EXP 1074 F53-61

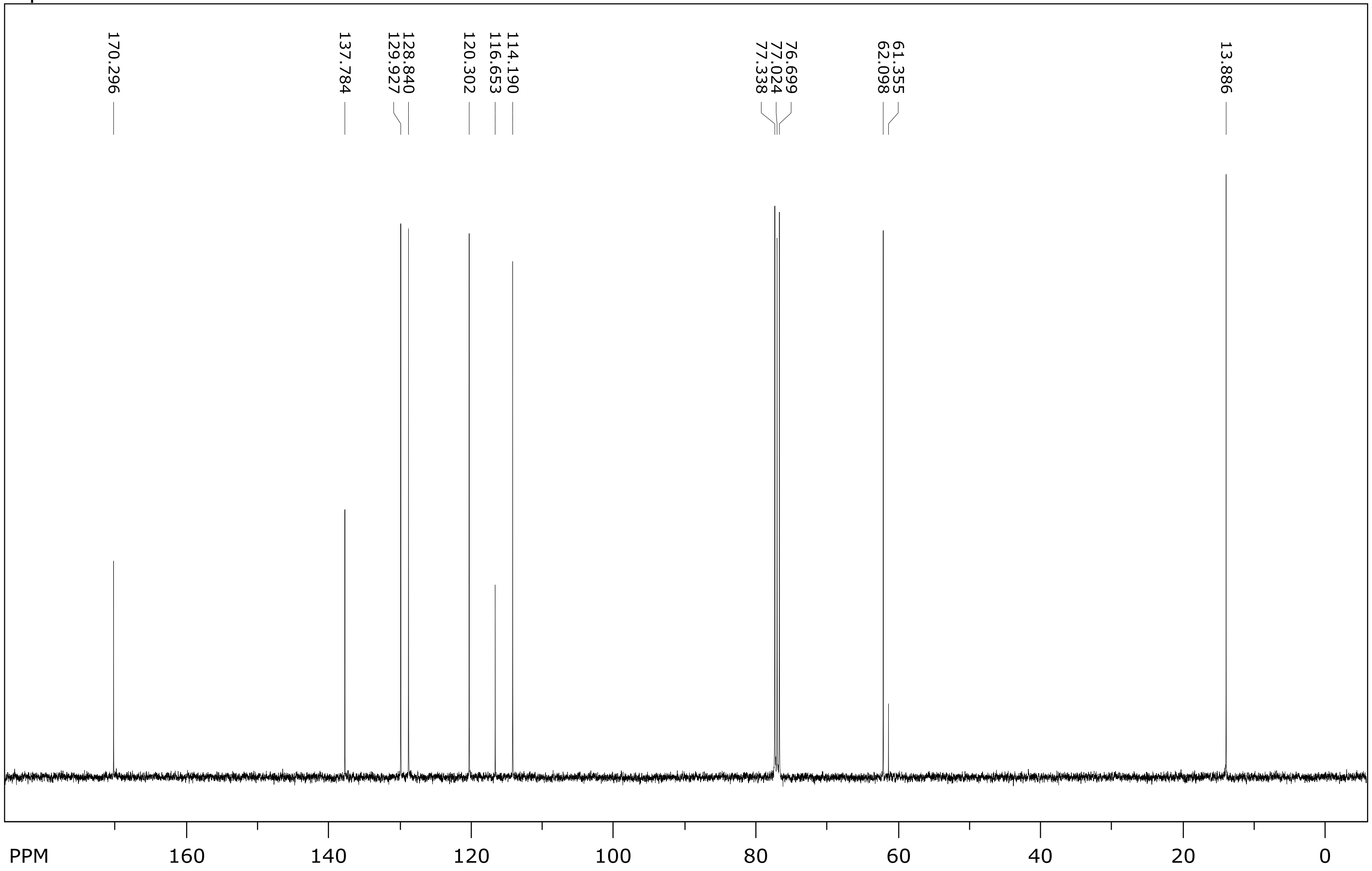

file: ...-1074-1\p6370teh_Carbon_ft-1-1.jdf expt: undefined
transmitter freq.: 100.525303 MHz
time domain size: 65536 points
width: 31407.04 Hz = 312.4292 ppm = 0.479233 Hz/pt
number of scans: 256

freq. of 0 ppm: 100.515263 MHz
processed size: 32768 complex points
LB: 0.000 GF: 0.0000

teh404

York - Chemistry - Mass Spectrometry Service Report

Analysis Information

Acquisition Date 22/10/2015 15:15:00

Analysis Filename	rjkt55200th_P1-B-4_01_61690.d
Method	400p_meoh1260_2c1s.m
Submission Name	rjkt55200th
Instrument	micrOTOF
ESI	Positive

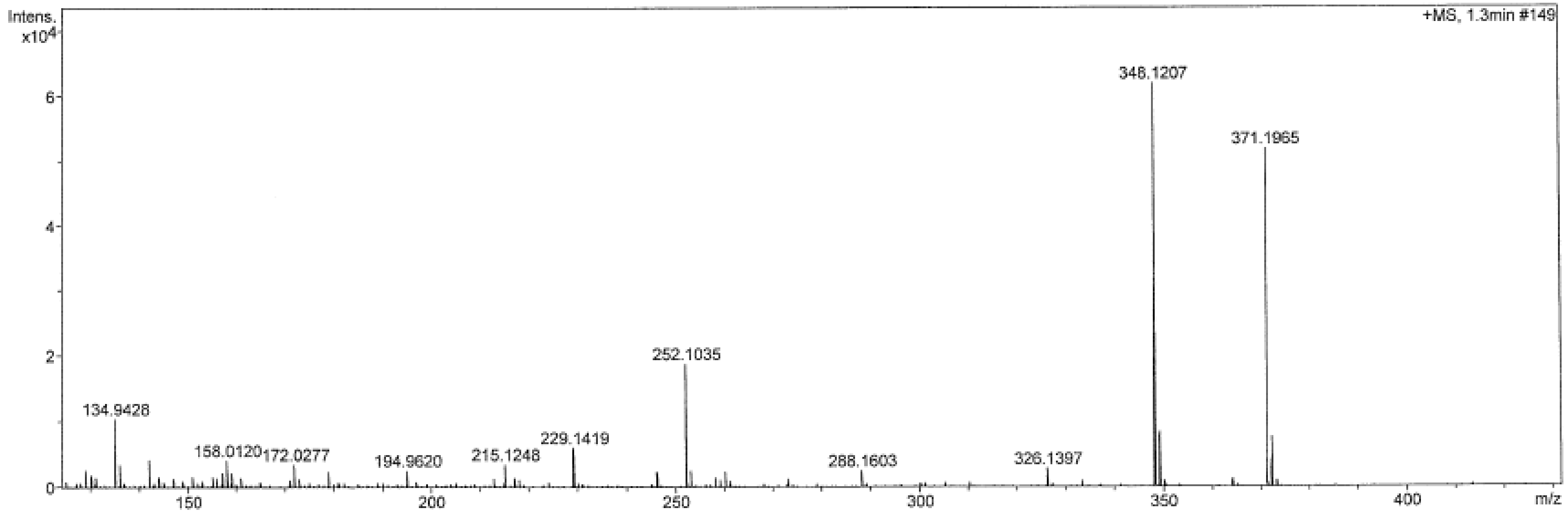

Meas. m/z	#	Formula	m/z	err [ppm]	err [mDa]	mSigma	Mean err [ppm]
348.1207	1	C 19 H 19 N Na O 4	348.1206	-0.2	-0.1	46.4	-0.6

PerkinElmer Spectrum Version 10.03.07
27 October 2015 13:35

Analyst PEService
Date 27 October 2015 13:35

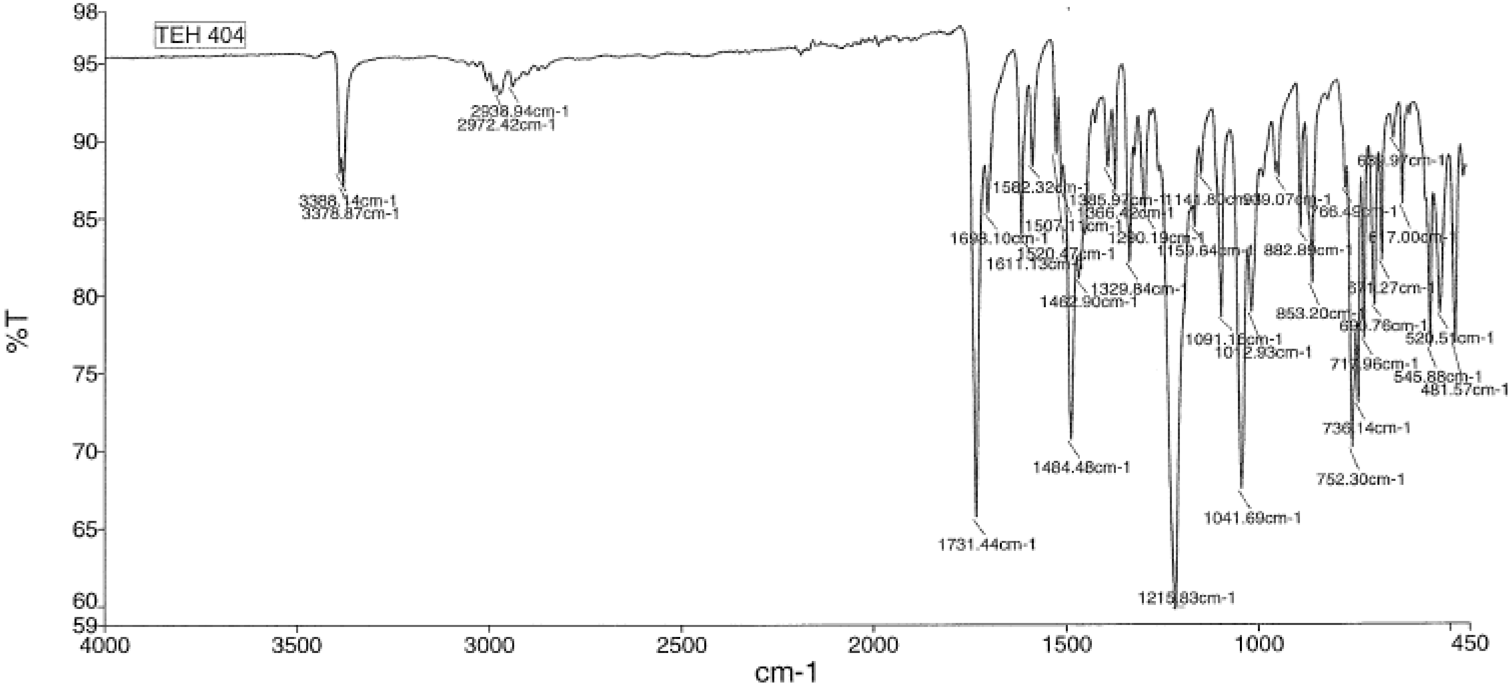

PEService 141 Sample 141 By PEService Date Tuesday, October 27 2015

2-Bromo-*N*-phenylaniline S6

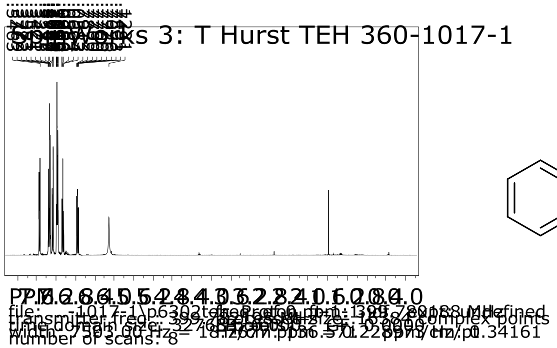

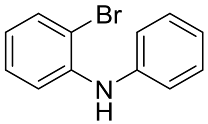

SpinWorks 3: Hurst TEH 360-1017-1

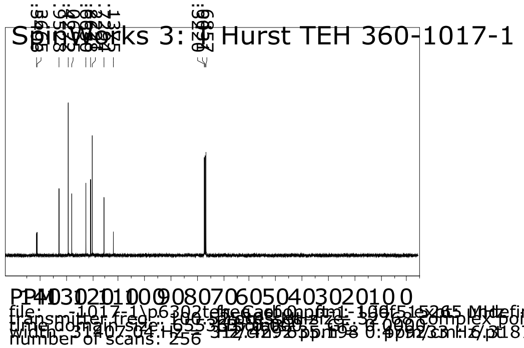

[illegible]
[illegible]
[illegible]
[illegible]
number of scans: 256

2-Bromo-5-methyl-*N*-phenylaniline S7

SpinWorks 4: T Hurst TEH 403-1073-1

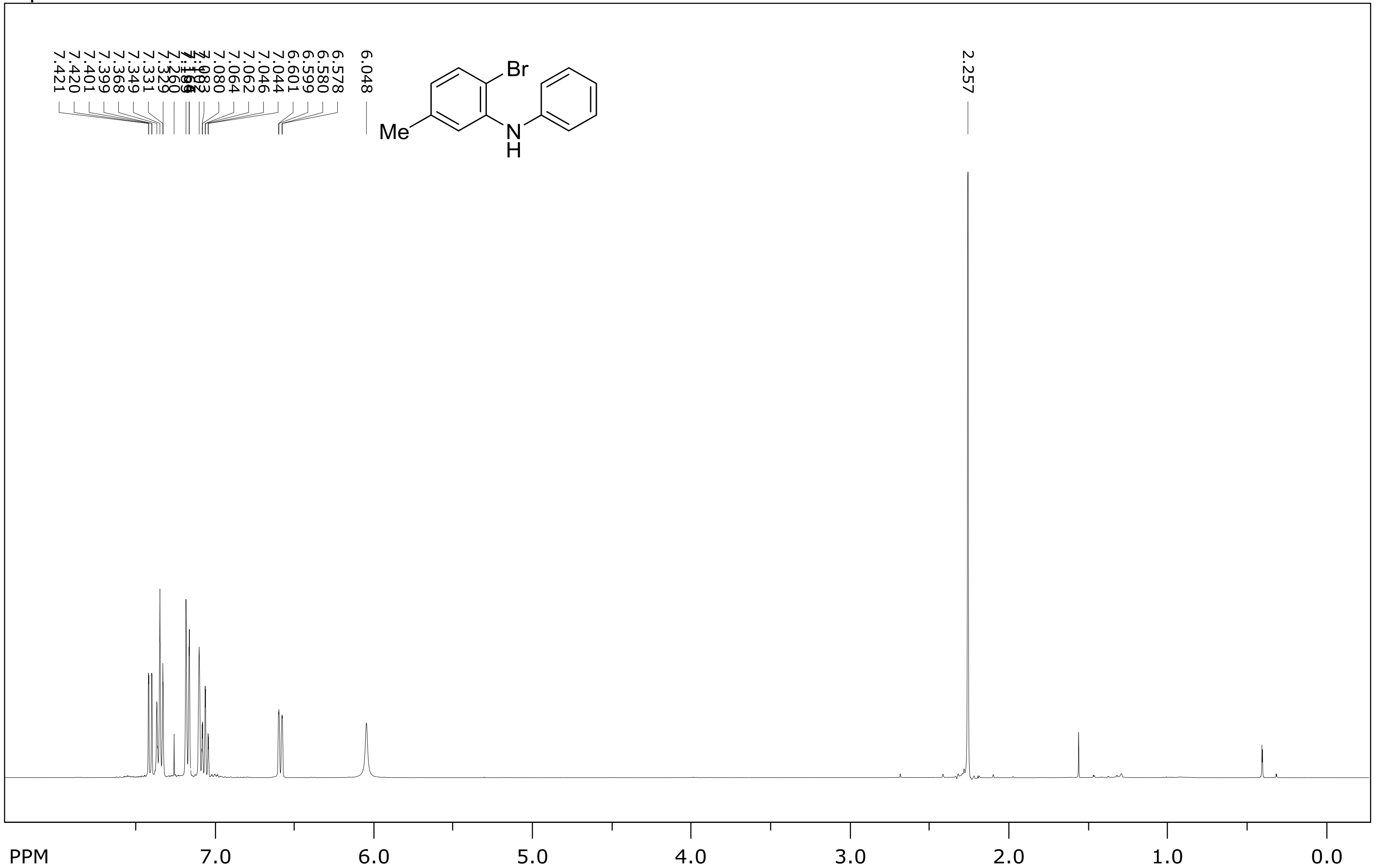

file: ...-1073-1\p6304teh_Proton_ft-1-1.jdf expt: undefined
transmitter freq.: 399.782198 MHz
time domain size: 32768 points
width: 7503.00 Hz = 18.7677 ppm = 0.228973 Hz/pt
number of scans: 8

freq. of 0 ppm: 399.780194 MHz
processed size: 16384 complex points
LB: 0.000 GF: 0.0000

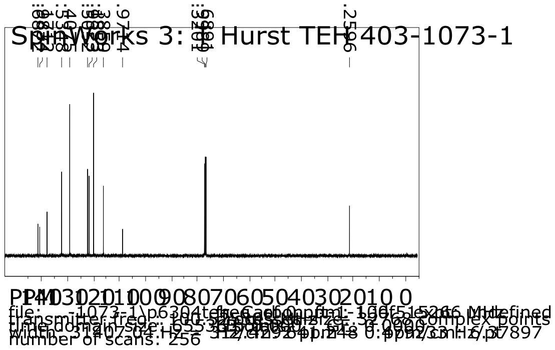
number of scans: 256

teh403

York - Chemistry - Mass Spectrometry Service Report

Analysis Information

Acquisition Date 22/10/2015 15:12:04

Analysis Filename	rjkt55199th_P1-B-3_01_61689.d
Method	400p_meoh1260_2c1s.m
Submission Name	rjkt55199th
Instrument	micrOTOF
ESI	Positive

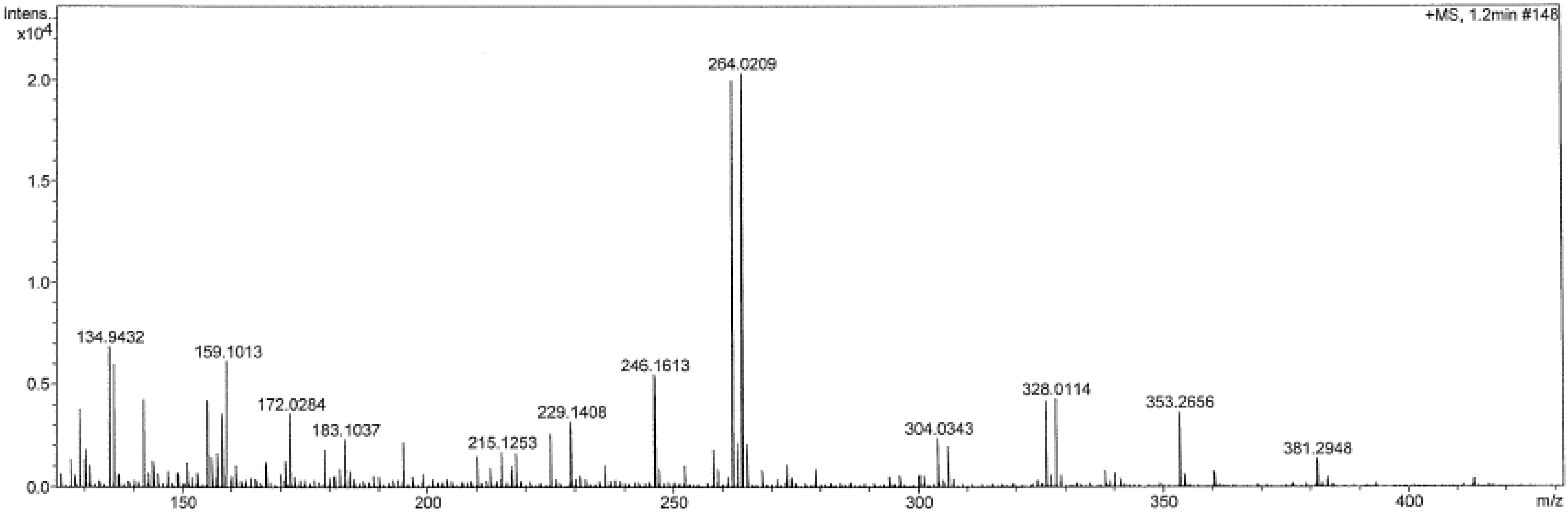

Meas. m/z	#	Formula	m/z	err [ppm]	err [mDa]	mSigma	Mean err [ppm]
262.0228	1	C 13 H 13 Br N	262.0226	-0.7	-0.2	26.9	-1.0

PerkinElmer Spectrum Version 10.03.07
27 October 2015 13:33

Analyst PEService
Date 27 October 2015 13:33

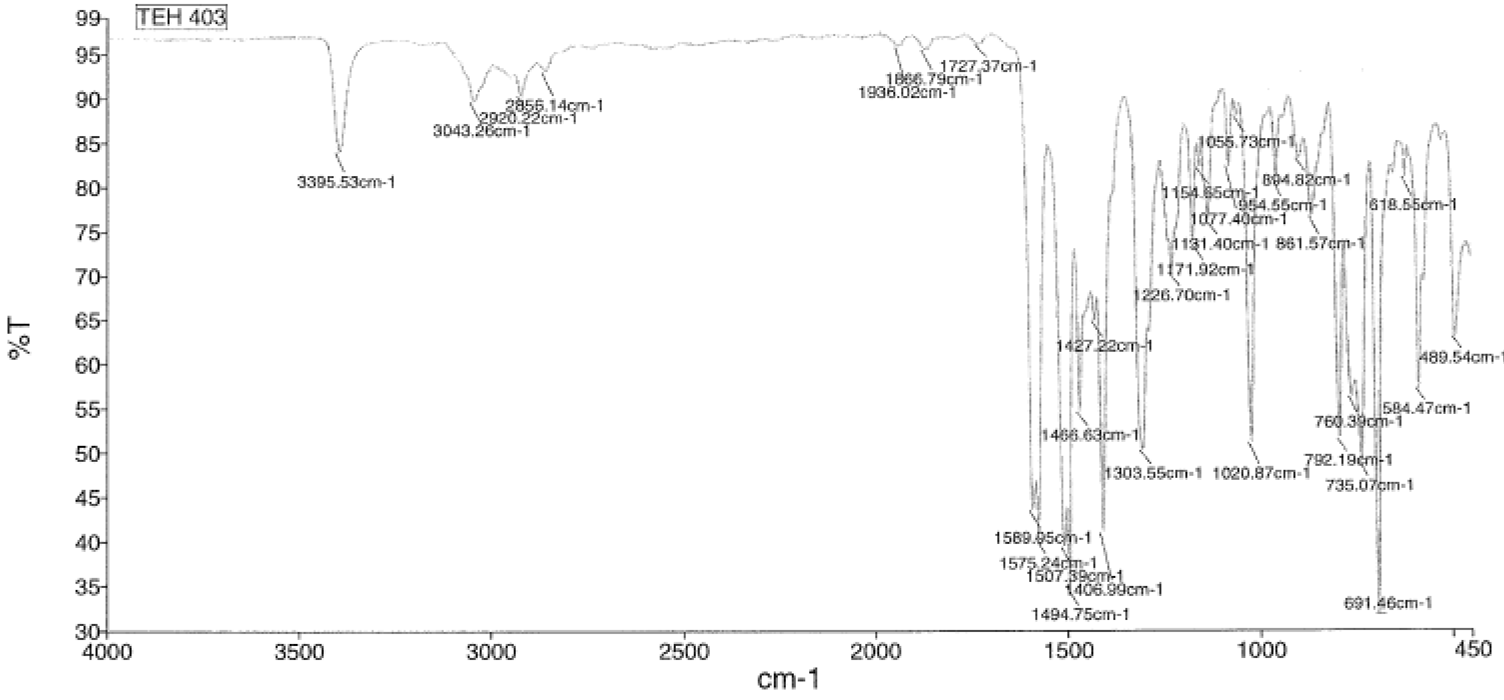

PEService 140 Sample 140 By PEService Date Tuesday, October 27 2015

***N*-(2-Bromo-4-fluorophenyl)-2*H*-1,3-benzodioxol-5-amine S8**

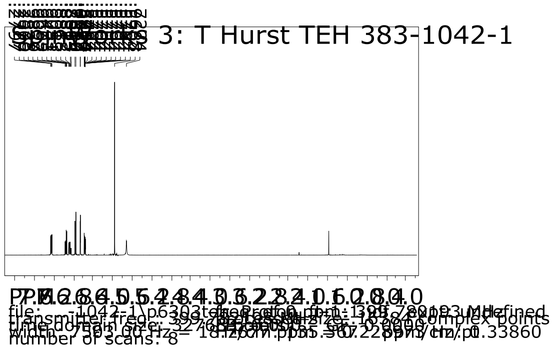

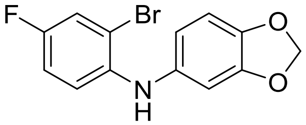

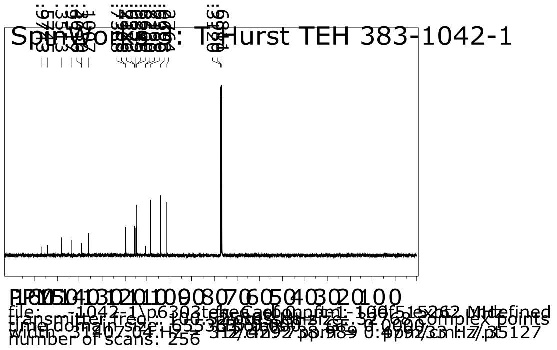
Hurst TEH 383-1042-1
number of scans: 256

teh383

York - Chemistry - Mass Spectrometry Service Report

Analysis Information

Acquisition Date 20/10/2015 11:49:35

Analysis Filename	rjkt55141th_P1-B-2_01_61620.d
Method	400p_meoh1260_2c1s.m
Submission Name	rjkt55141th
Instrument	micrOTOF
ESI	Positive

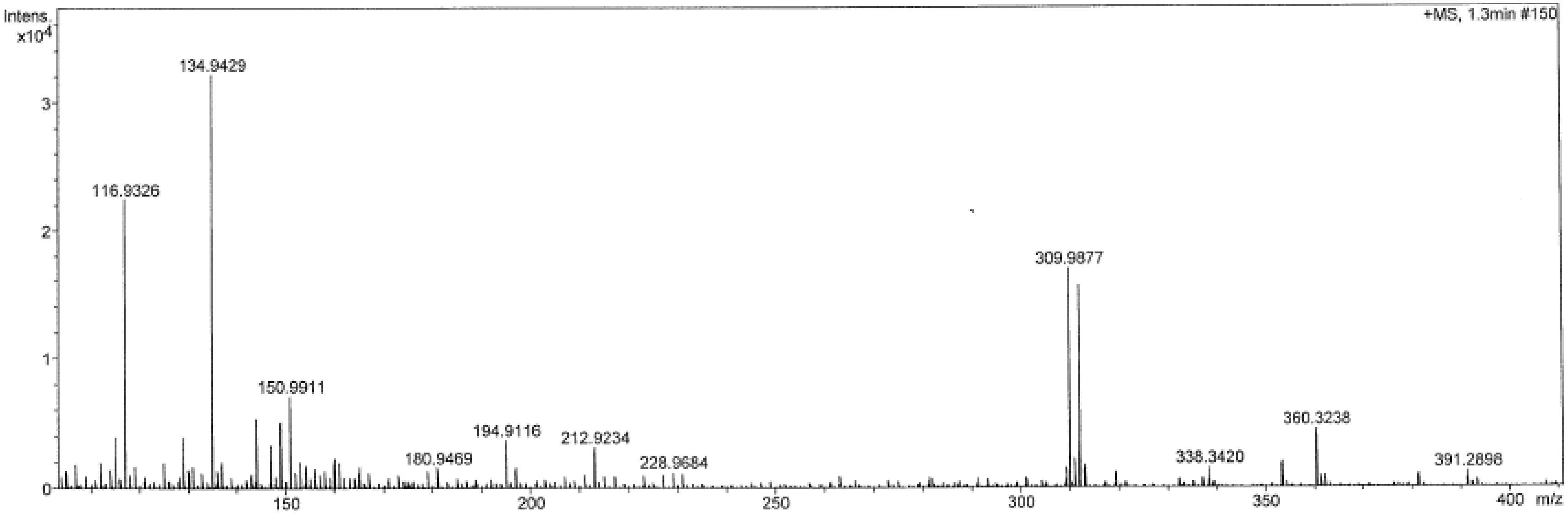

Meas. m/z	#	Formula	m/z	err [ppm]	err [mDa]	mSigma	Mean err [ppm]
309.9877	1	C 13 H 10 Br F N O 2	309.9873	-1.3	-0.4	34.6	-3.1

PerkinElmer Spectrum Version 10.03.07
14 October 2015 21:52

Analyst PEService
Date 14 October 2015 21:52

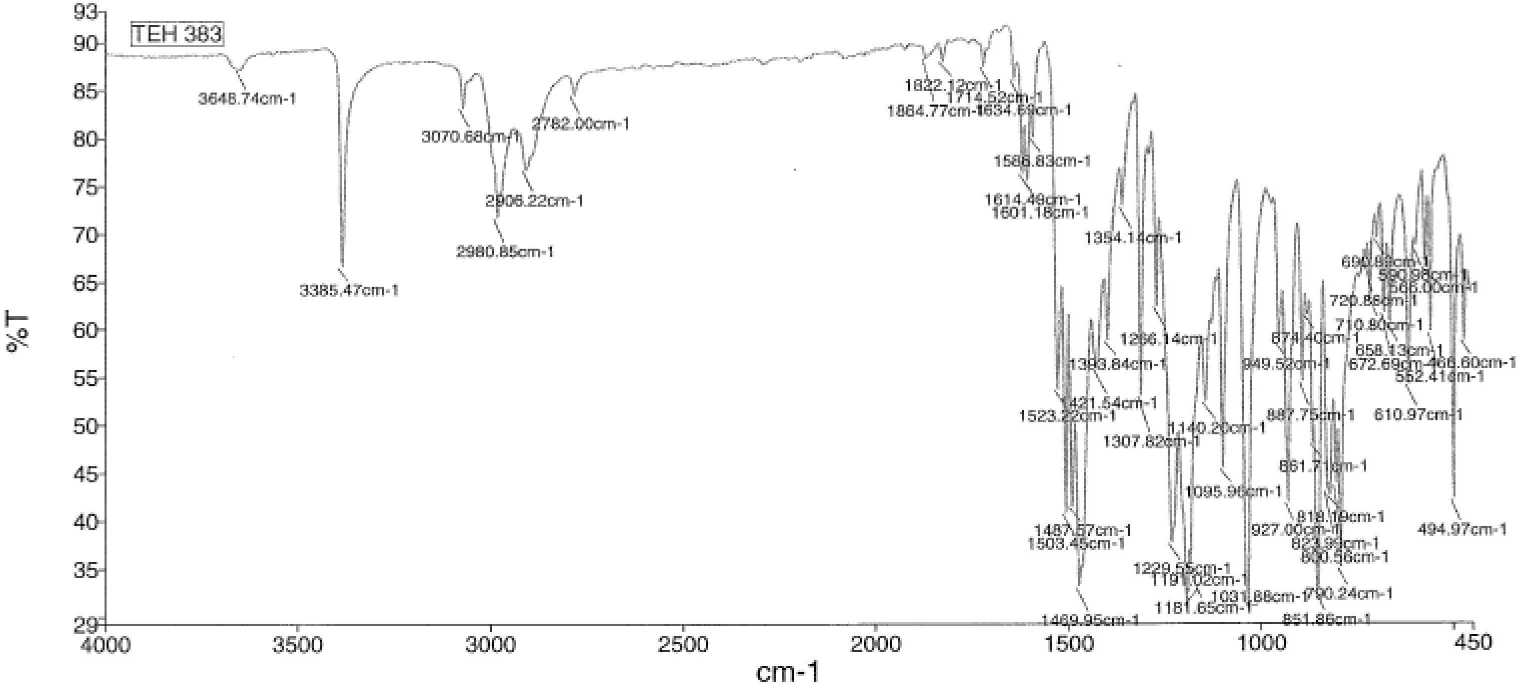

PEService 44 Sample 044 By PEService Date Wednesday, October 14 2015

2-Bromo-*N*-methyl-*N*-phenylaniline 21b

SpinWorks 4: T Hurst TEH 365-997-1

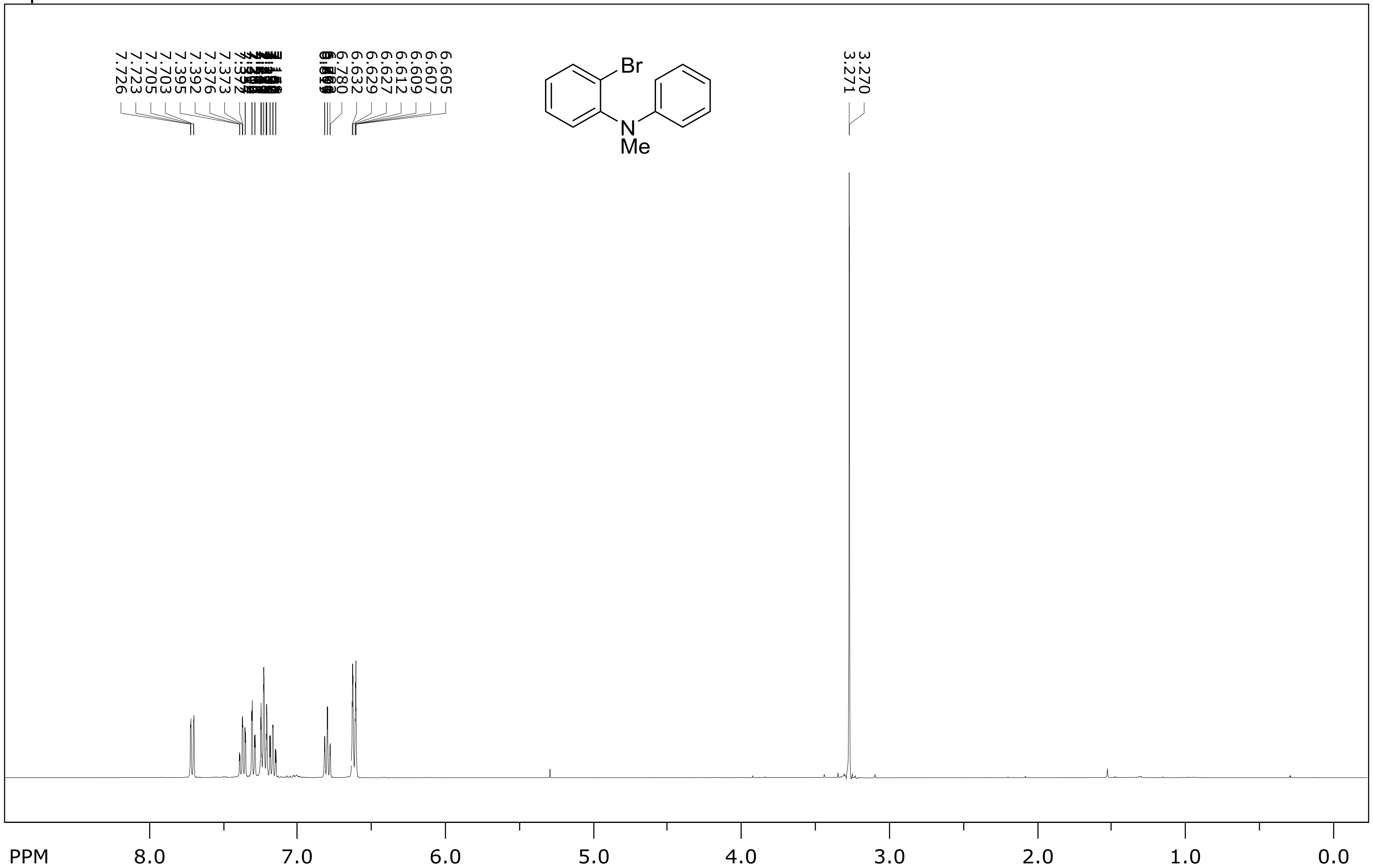

file: ...5-997-1\p2976teh_Proton_ft-1-1.jdf expt: undefined
transmitter freq.: 399.782198 MHz
time domain size: 32768 points
width: 7503.00 Hz = 18.7677 ppm = 0.228973 Hz/pt
number of scans: 8

freq. of 0 ppm: 399.780199 MHz
processed size: 16384 complex points
LB: 0.000 GF: 0.0000

SpinWorks 4: T Hurst TEH 365-997-1

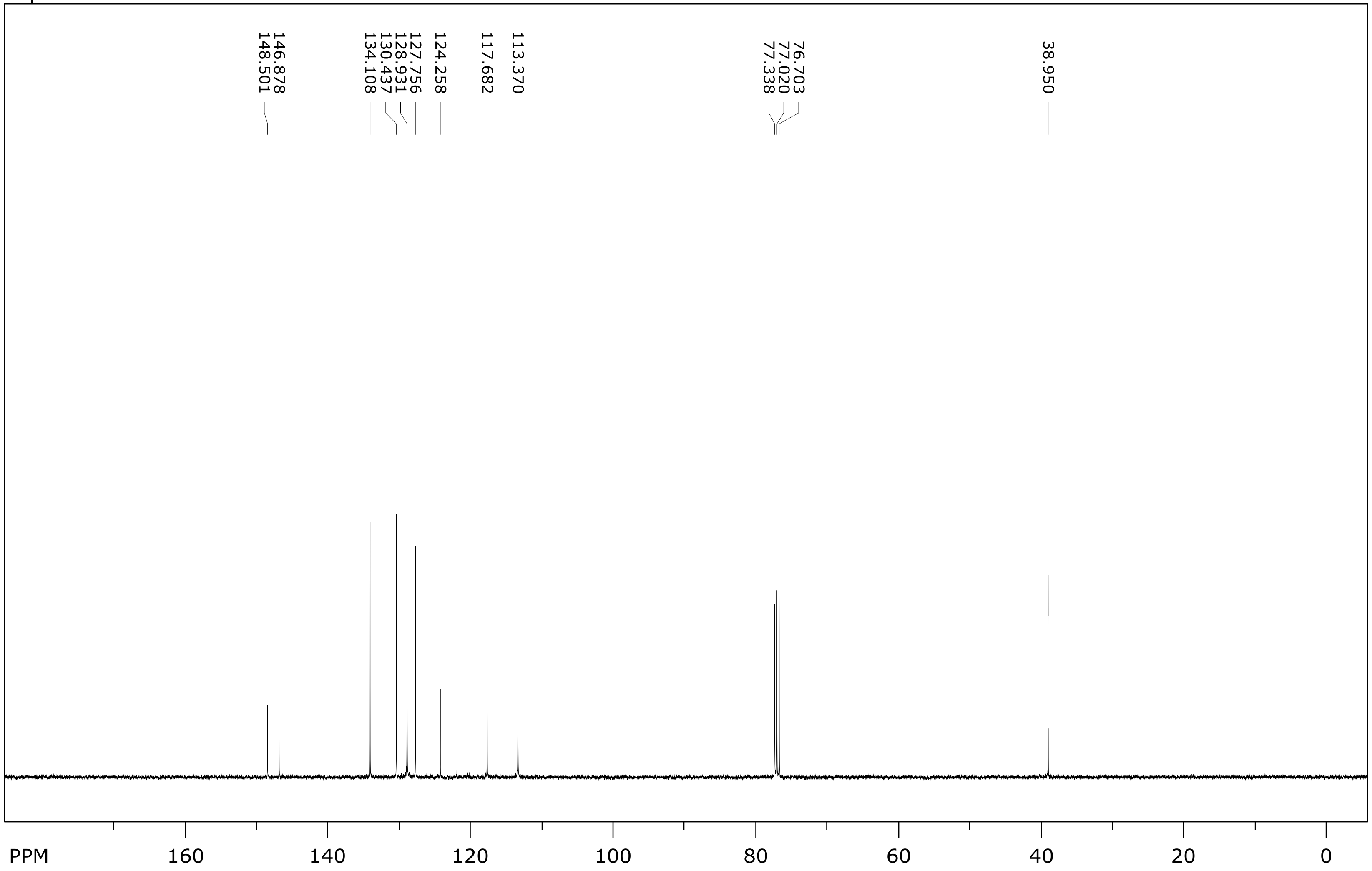

file: ...5-997-1\p2976teh_Carbon_ft-1-1.jdf   expt: undefined
transmitter freq.: 100.525303 MHz
time domain size: 65536 points
width: 31407.04 Hz = 312.4292 ppm = 0.479233 Hz/pt
number of scans: 256

freq. of 0 ppm: 100.515268 MHz
processed size: 32768 complex points
LB: 0.000   GF: 0.0000

2-Bromo-*N*,5-dimethyl-*N*-phenylaniline 21c

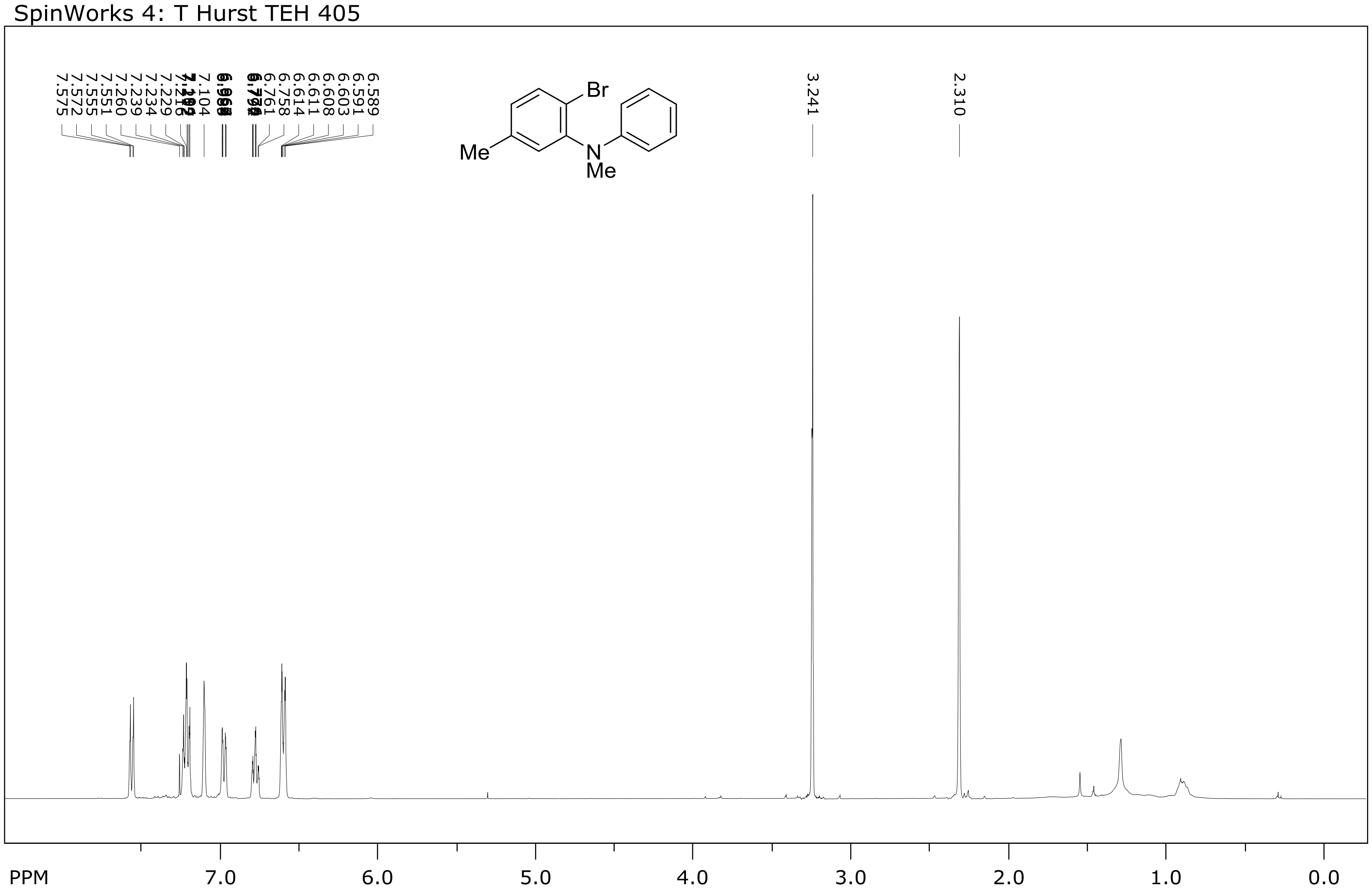

file: ...-1075-1\p6730teh_Proton_ft-1-1.jdf   expt: undefined
transmitter freq.: 399.782198 MHz
time domain size: 32768 points
width: 7503.00 Hz = 18.7677 ppm = 0.228973 Hz/pt
number of scans: 8

freq. of 0 ppm: 399.780194 MHz
processed size: 16384 complex points
LB: 0.000   GF: 0.0000

SpinWorks 4: T Hurst TEH 405

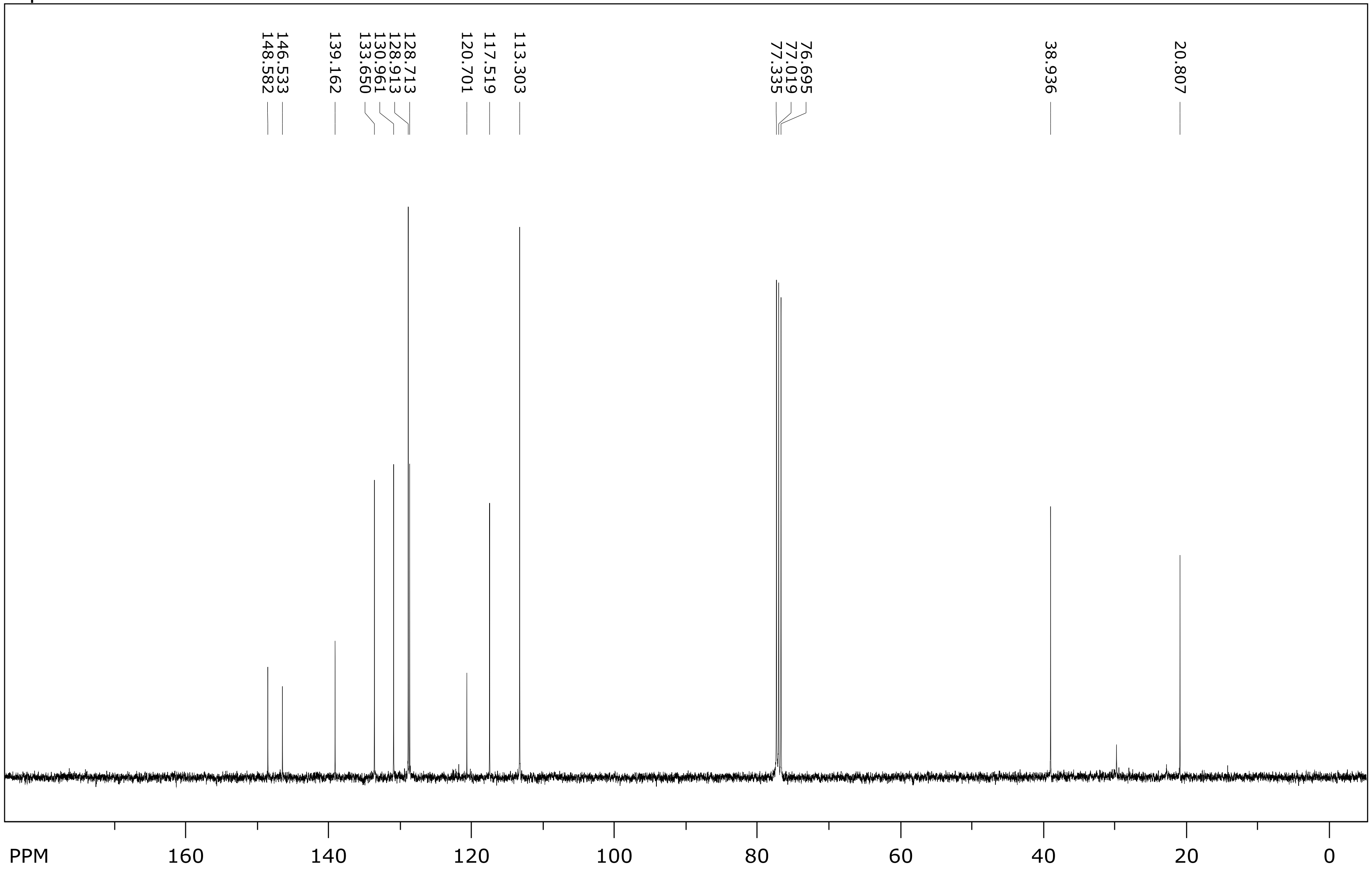

file: ...-1075-1\p6730teh_Carbon_ft-1-1.jdf   expt: undefined
transmitter freq.: 100.525303 MHz
time domain size: 65536 points
width: 31407.04 Hz = 312.4292 ppm = 0.479233 Hz/pt
number of scans: 256

freq. of 0 ppm: 100.515263 MHz
processed size: 32768 complex points
LB: 0.000   GF: 0.0000

teh405

York - Chemistry - Mass Spectrometry Service Report

Analysis Information

Acquisition Date 22/10/2015 15:17:58

Analysis Filename	rjkt55201th_P1-B-5_01_61691.d
Method	400p_meoh1260_2c1s.m
Submission Name	rjkt55201th
Instrument	micrOTOF
ESI	Positive

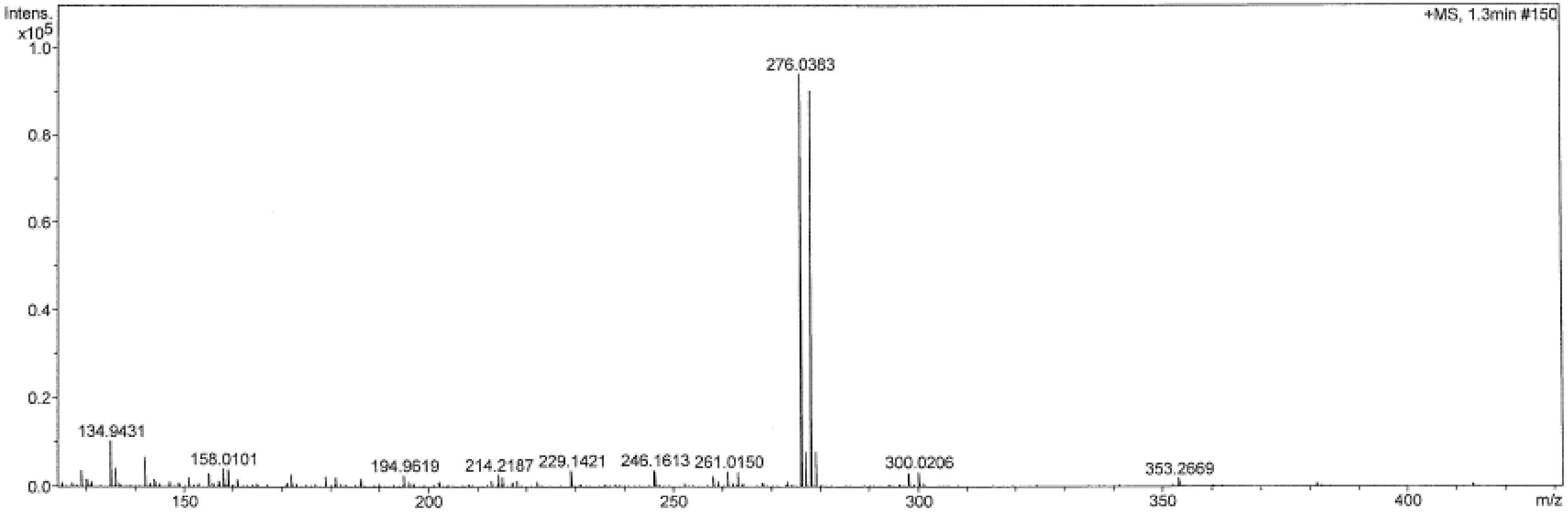

Meas. m/z	#	Formula	m/z	err [ppm]	err [mDa]	mSigma	Mean err [ppm]
276.0383	1	C 14 H 15 Br N	276.0382	-0.0	-0.0	45.4	-1.4

PerkinElmer Spectrum Version 10.03.07
27 October 2015 13:39

Analyst PEService
Date 27 October 2015 13:39

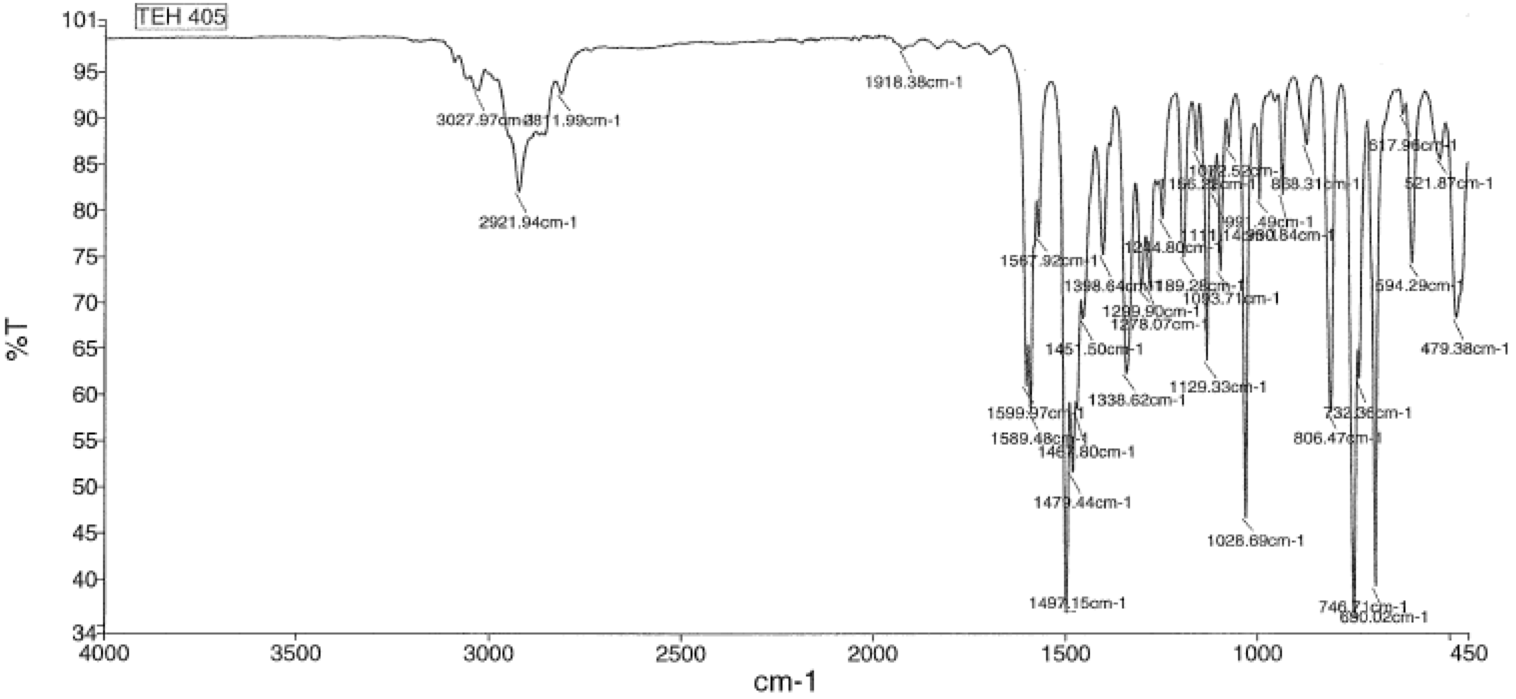

PEService 143 Sample 143 By PEService Date Tuesday, October 27 2015

_N_-(2-Bromo-4-fluorophenyl)-_N_-methyl-2_H_-1,3-benzodioxol-5-amine 21d

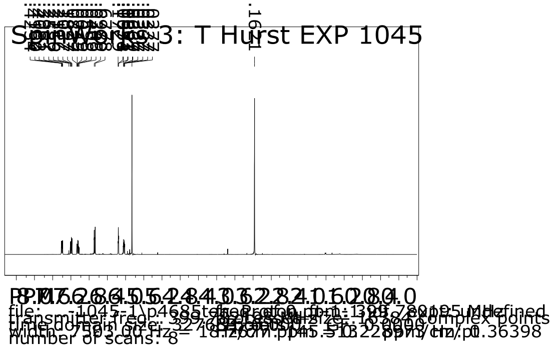

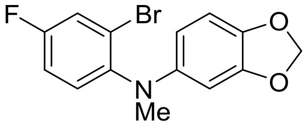

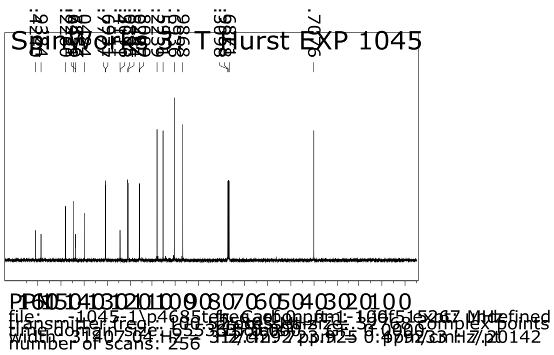
number of scans: 256

teh386

York - Chemistry - Mass Spectrometry Service Report

Analysis Information Acquisition Date 20/10/2015 11:52:33

Analysis Filename	rjkt55142th_P1-B-3_01_61621.d
Method	400p_meoh1260_2c1s.m
Submission Name	rjkt55142th
Instrument	micrOTOF
ESI	Positive

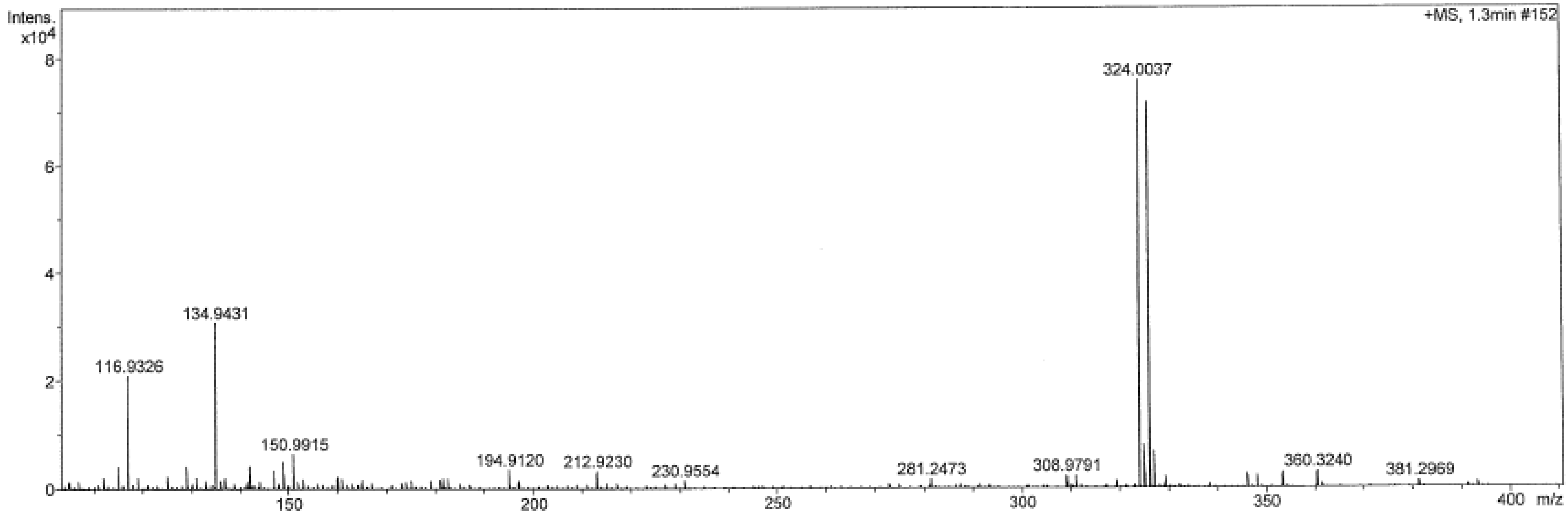

Meas. m/z	#	Formula	m/z	err [ppm]	err [mDa]	mSigma	Mean err [ppm]
324.0037	1	C 14 H 12 Br F N O 2	324.0030	-2.3	-0.7	41.6	-2.0

PerkinElmer Spectrum Version 10.03.07
14 October 2015 21:55

Analyst PEService
Date 14 October 2015 21:55

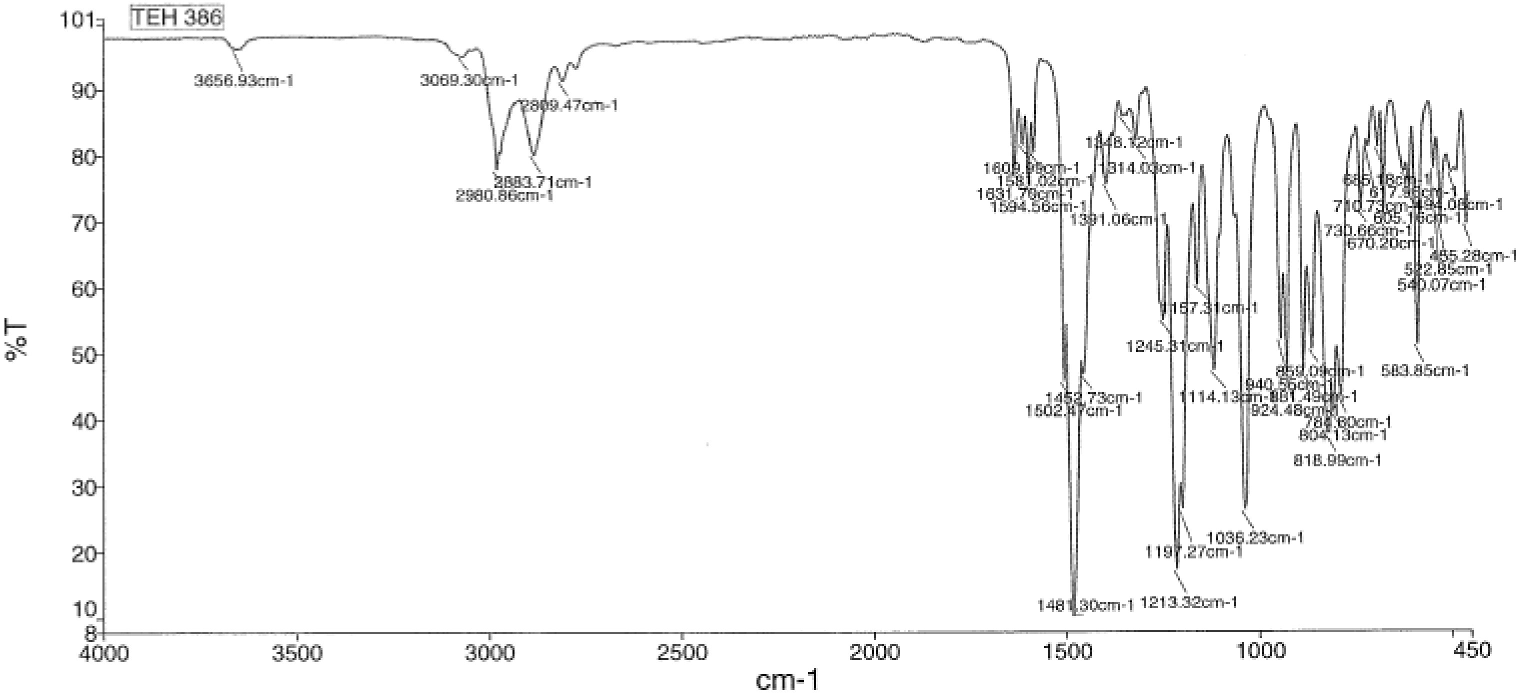

PEService 45 Sample 045 By PEService Date Wednesday, October 14 2015

1,2-Diethyl 2-(2-[ethyl(phenyl)amino]phenyl)propanedioate 15n

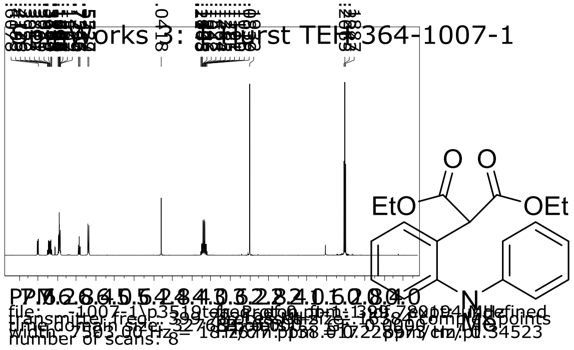

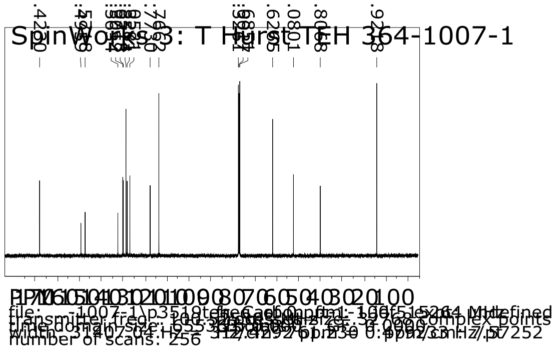

teh364

York - Chemistry - Mass Spectrometry Service Report

Analysis Information

Acquisition Date 20/10/2015 11:55:29

Analysis Filename	rjkt55143th_P1-B-4_01_61622.d
Method	400p_meoh1260_2c1s.m
Submission Name	rjkt55143th
Instrument	micrOTOF
ESI	Positive

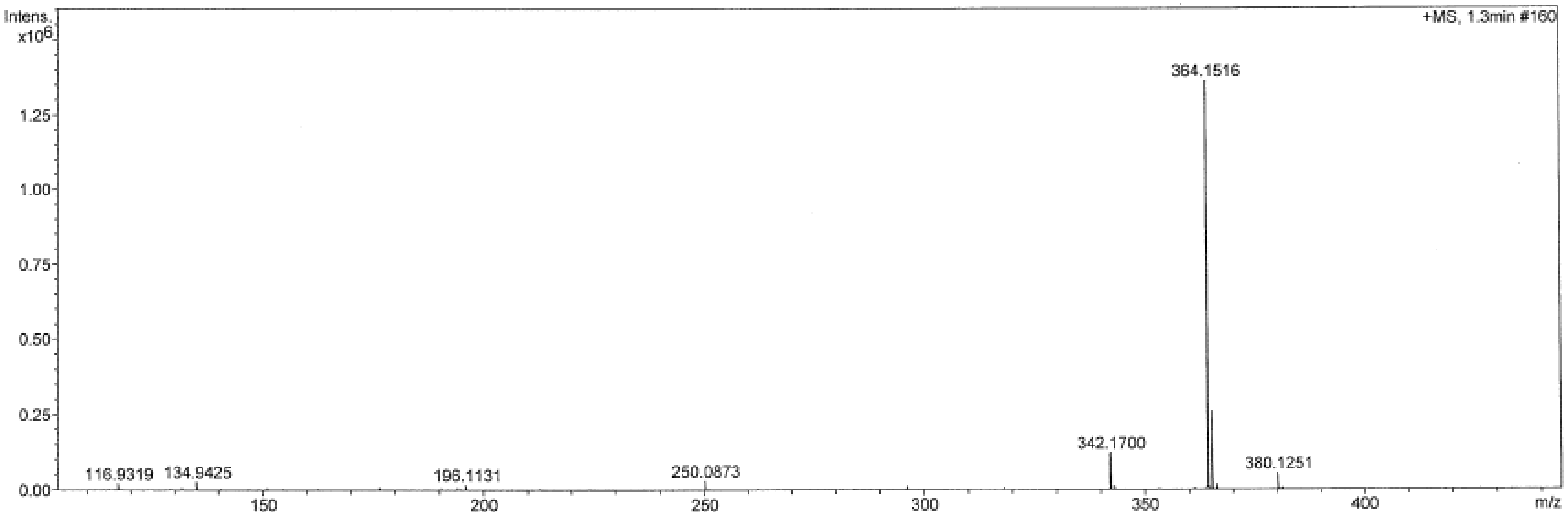

Meas. m/z	#	Formula	m/z	err [ppm]	err [mDa]	mSigma	Mean err [ppm]
342.1700	1	C 20 H 24 N O 4	342.1700	0.1	0.0	53.2	0.3
364.1516	1	C 20 H 23 N Na O 4	364.1519	1.0	0.3	19.4	1.6

PerkinElmer Spectrum Version 10.03.07
14 October 2015 21:41

Analyst PEService
Date 14 October 2015 21:41

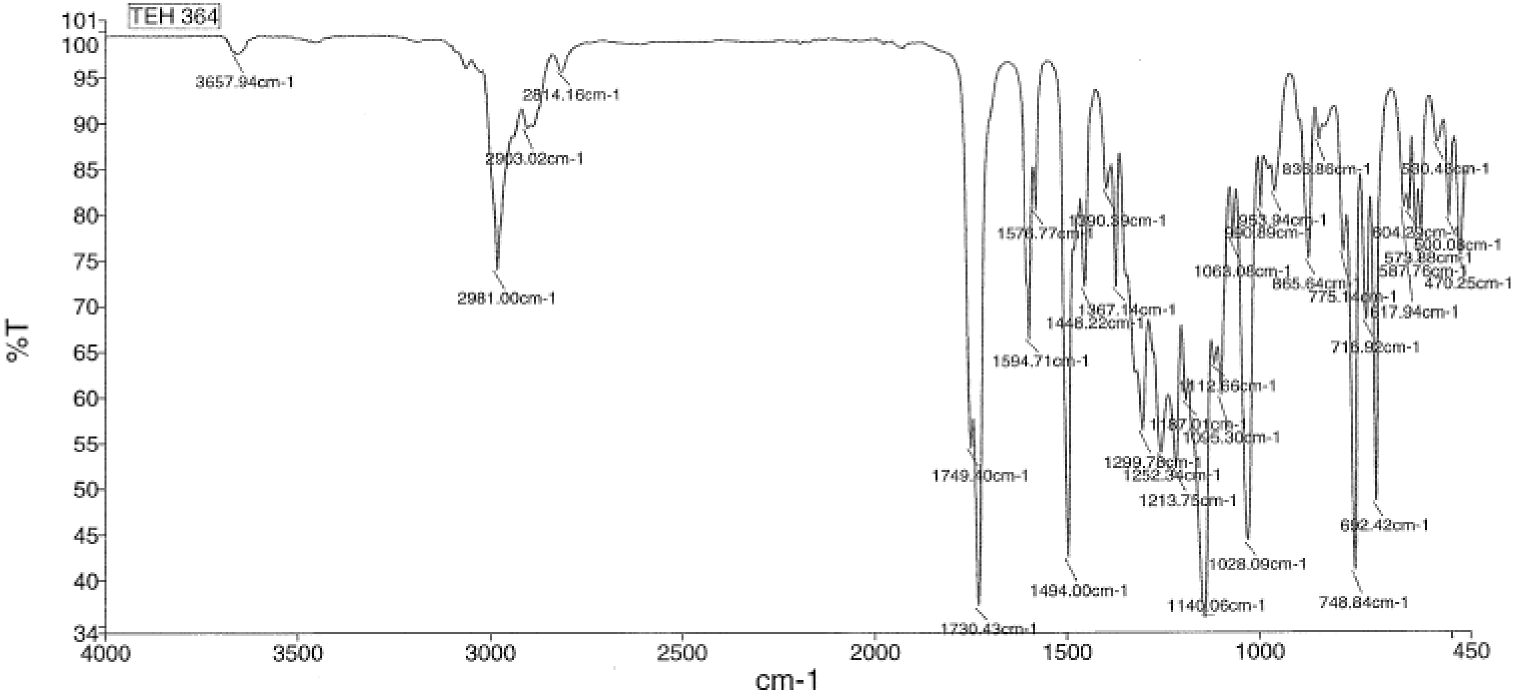

PEService 38 Sample 038 By PEService Date Wednesday, October 14 2015

9,9-Diethyl 10-methyl-9,10-dihydroacridine-9,9-dicarboxylate 16n

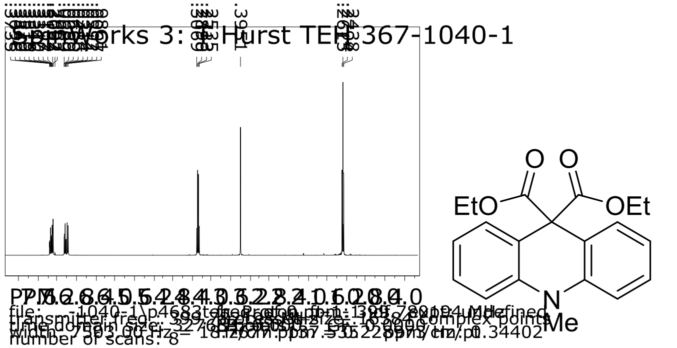

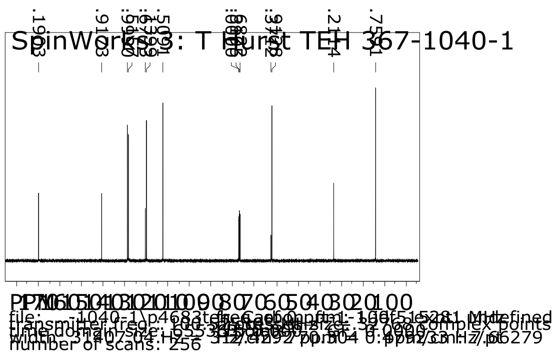

teh367

York - Chemistry - Mass Spectrometry Service Report

Analysis Information

Acquisition Date 20/10/2015 11:58:25

Analysis Filename	rjkt55144th_P1-B-5_01_61623.d
Method	400p_meoh1260_2c1s.m
Submission Name	rjkt55144th
Instrument	micrOTOF
ESI	Positive

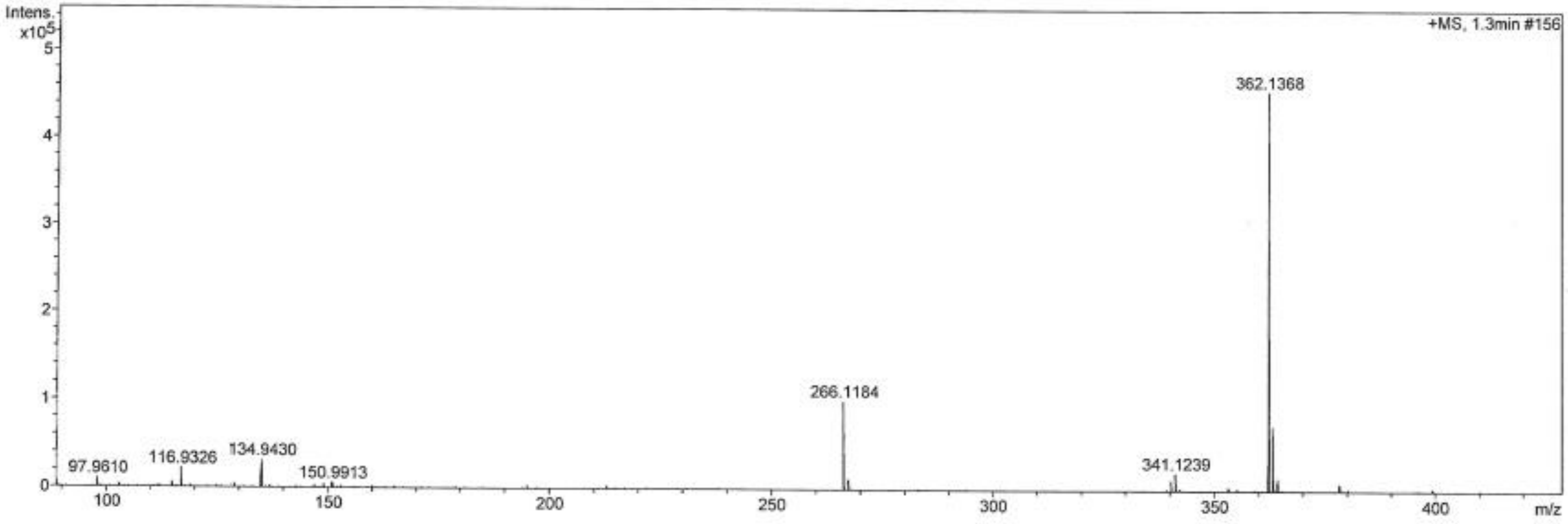

Meas. m/z	#	Formula	m/z	err [ppm]	err [mDa]	mSigma	Mean err [ppm]
340.1553	1	C 20 H 22 N O 4	340.1543	-2.8	-1.0	164.1	11.2
362.1368	1	C 20 H 21 N Na O 4	362.1363	-1.4	-0.5	35.5	-1.8

overlapping isotope pattern

PerkinElmer Spectrum Version 10.03.07
14 October 2015 21:48

Analyst PEService
Date 14 October 2015 21:48

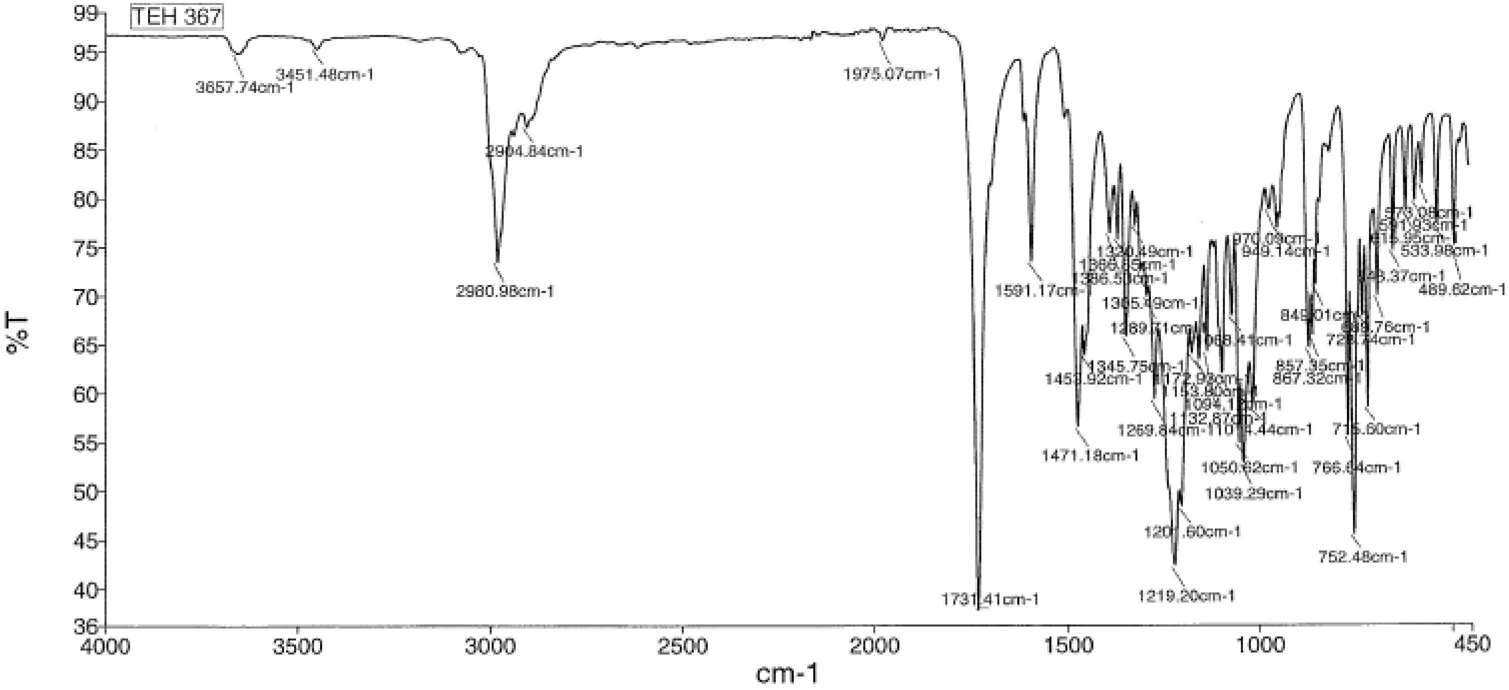

PEService 42 Sample 042 By PEService Date Wednesday, October 14 2015

9,9-Diethyl 3,10-dimethyl-9,10-dihydroacridine-9,9-dicarboxylate 16o

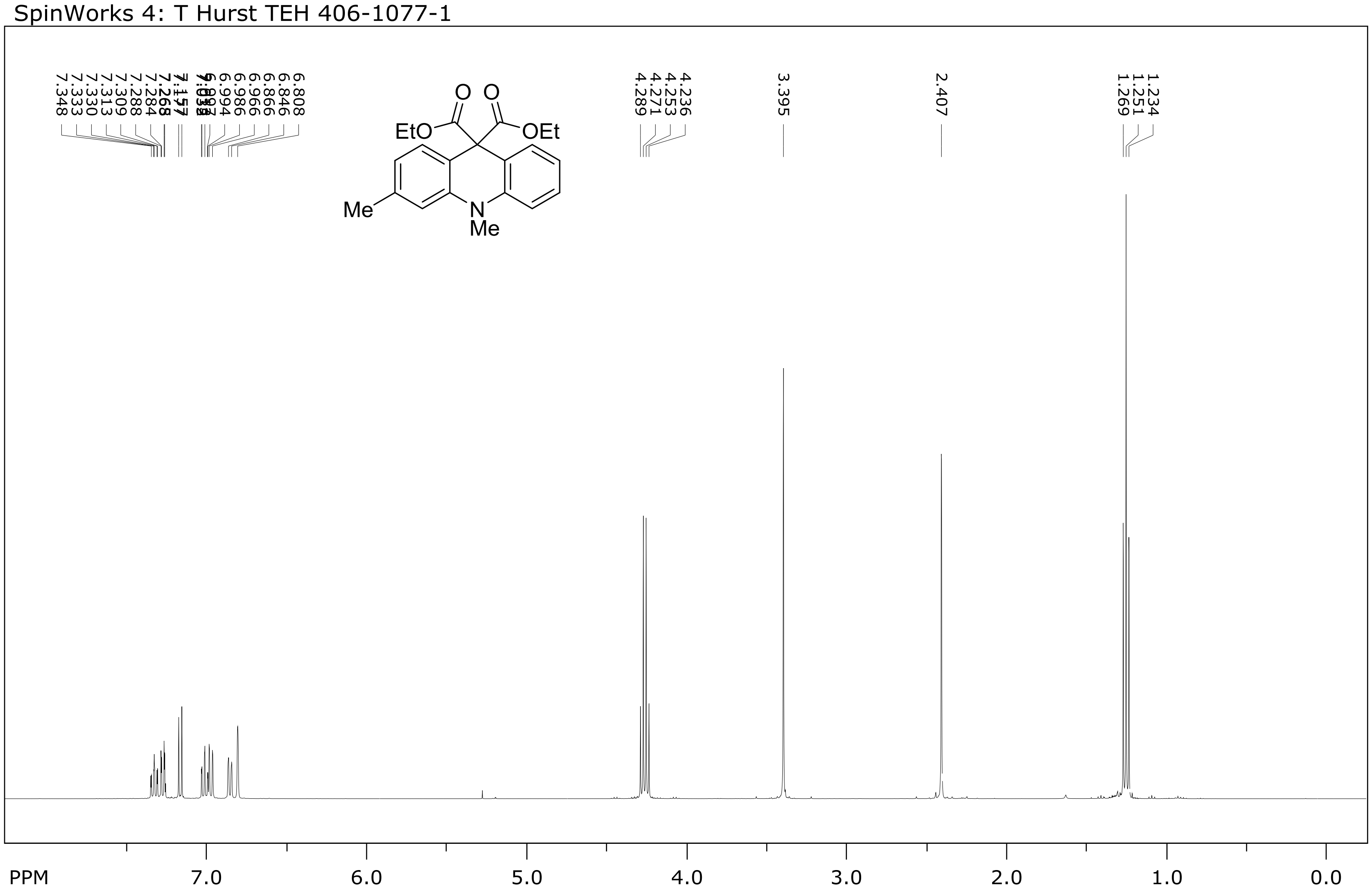

file: ...-1077-1\p6333teh_Proton_ft-1-1.jdf expt: undefined
transmitter freq.: 399.782198 MHz
time domain size: 32768 points
width: 7503.00 Hz = 18.7677 ppm = 0.228973 Hz/pt
number of scans: 8

freq. of 0 ppm: 399.780194 MHz
processed size: 16384 complex points
LB: 0.000 GF: 0.0000

SpinWorks 4: T Hurst TEH 406-1077-1

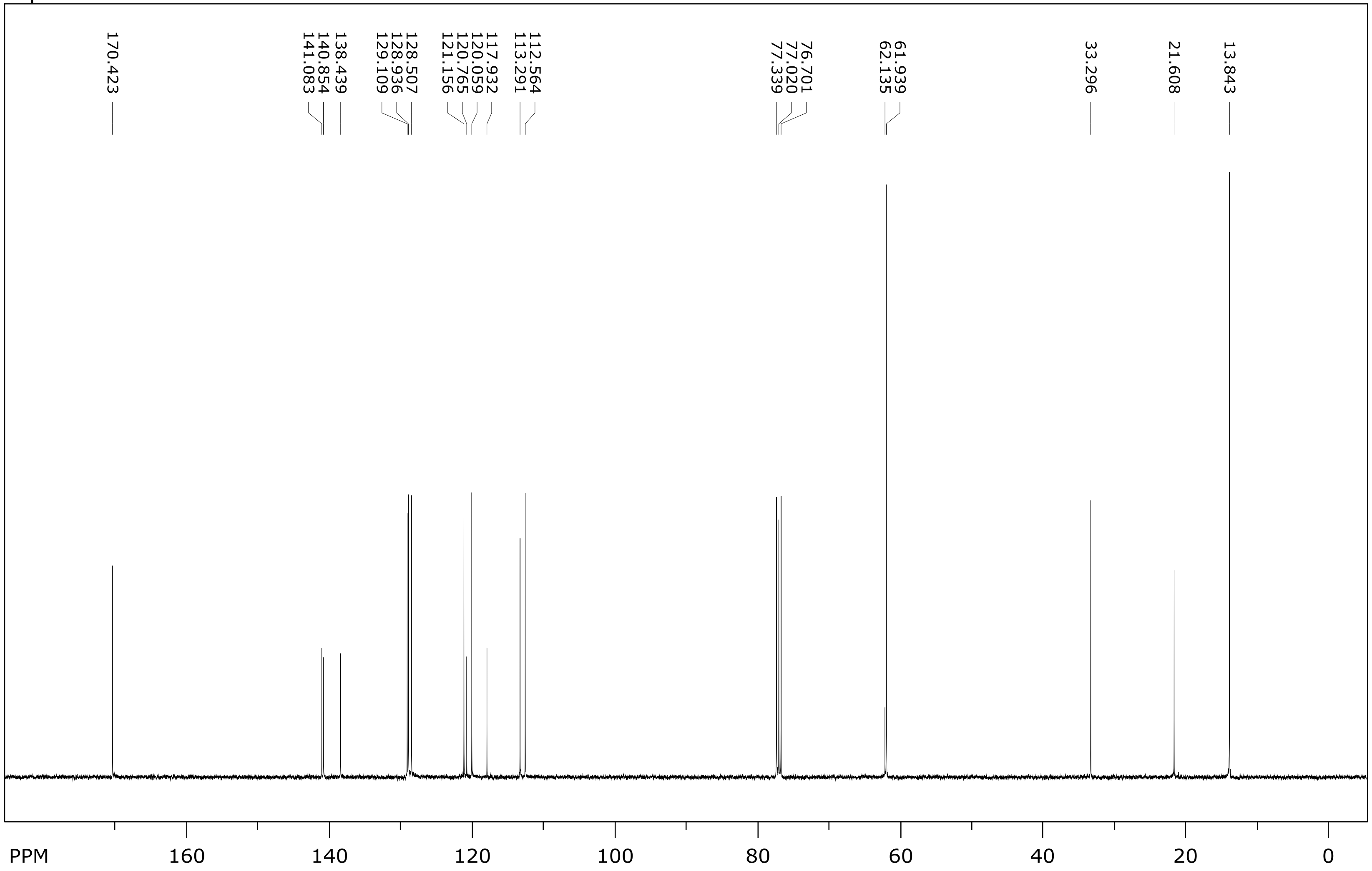

file: ...-1077-1\p6333teh_Carbon_ft-1-1.jdf expt: undefined
transmitter freq.: 100.525303 MHz
time domain size: 65536 points
width: 31407.04 Hz = 312.4292 ppm = 0.479233 Hz/pt
number of scans: 256

freq. of 0 ppm: 100.515272 MHz
processed size: 32768 complex points
LB: 0.000 GF: 0.0000

teh406

York - Chemistry - Mass Spectrometry Service Report

Analysis Information

Acquisition Date 27/10/2015 11:18:17

Analysis Filename	rjkt55313th_P1-F-2_01_61800.d
Method	400p_meoh1260_2c1s.m
Submission Name	rjkt55313th
Instrument	micrOTOF
ESI	Positive

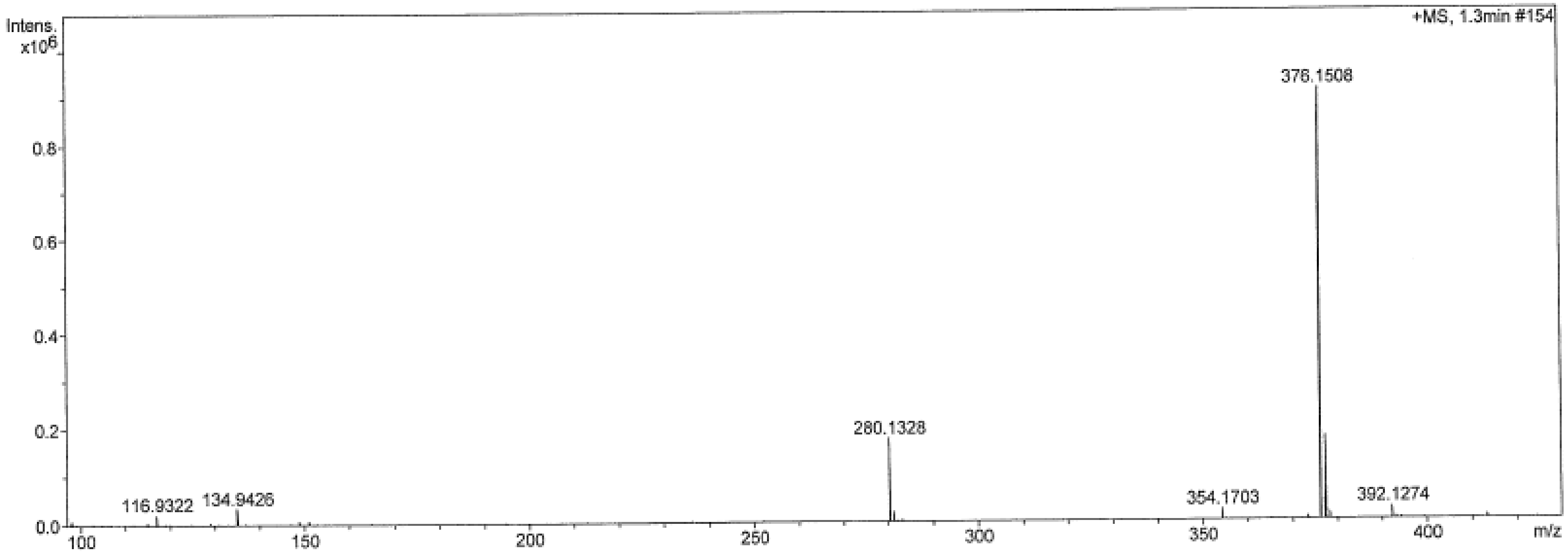

Meas. m/z	#	Formula	m/z	err [ppm]	err [mDa]	mSigma	Mean err [ppm]
354.1703	1	C 21 H 24 N O 4	354.1700	-0.8	-0.3	49.6	-0.4
376.1508	1	C 21 H 23 N Na O 4	376.1519	2.9	1.1	25.6	3.9

PerkinElmer Spectrum Version 10.03.07
27 October 2015 13:41

Analyst PEService
Date 27 October 2015 13:41

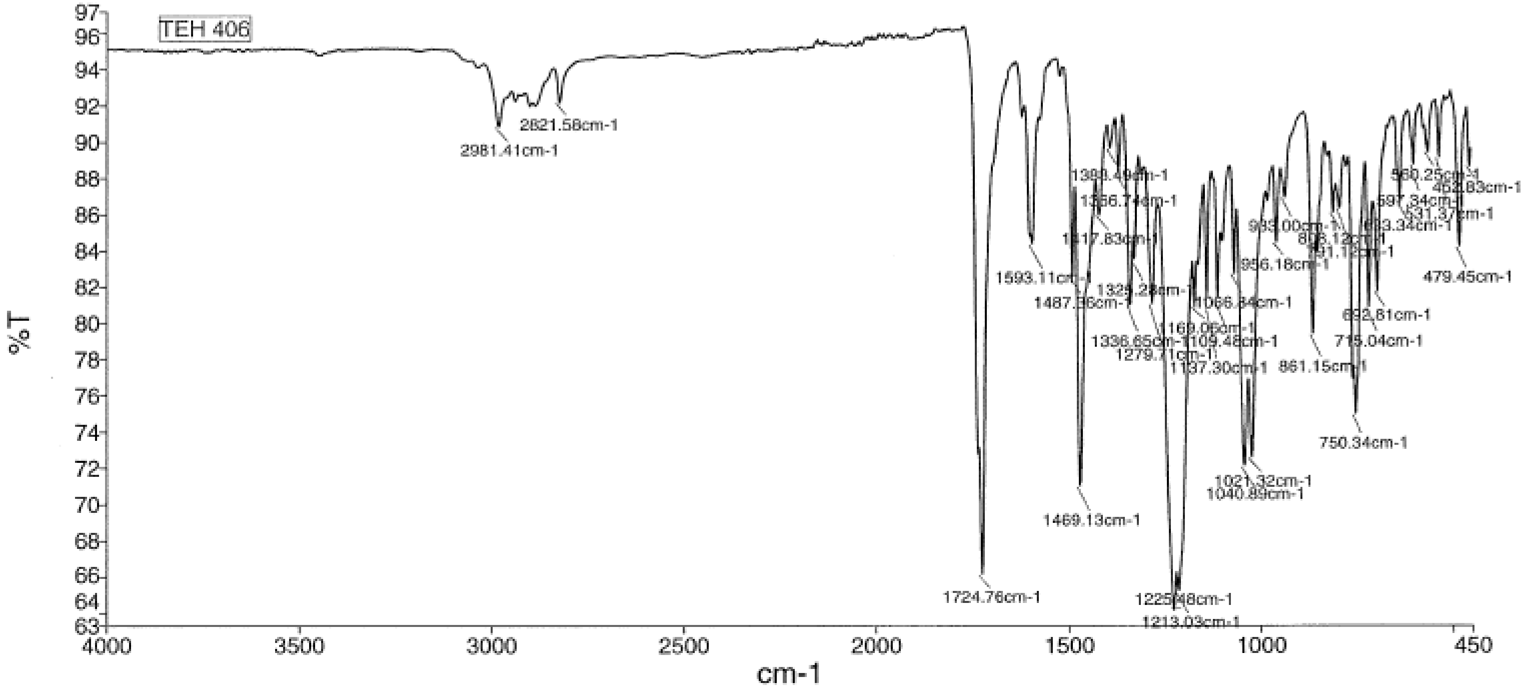

PEService 144 Sample 144 By PEService Date Tuesday, October 27 2015

10,10-Diethyl 8-fluoro-2*H*,5*H*,10*H*-[1,3]dioxolo[4,5-*b*]acridine-10,10-dicarboxylate 16p

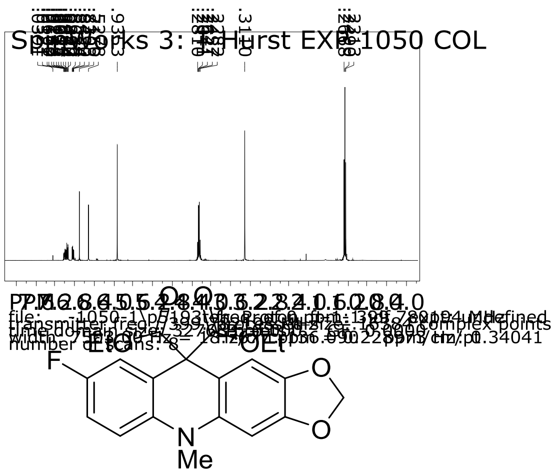

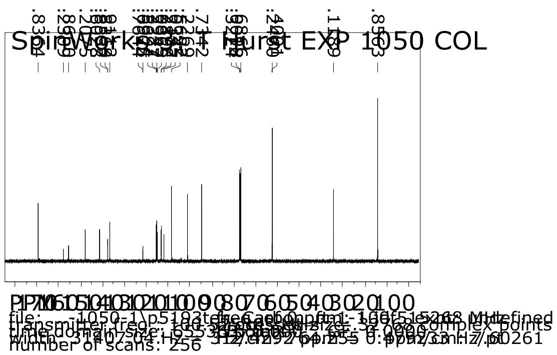
number of scans: 256

teh389

York - Chemistry - Mass Spectrometry Service Report

Analysis Information

Acquisition Date 29/09/2015 14:32:35

Analysis Filename	rjkt54815th_P1-F-3_01_61205.d
Method	600p_meoh1260_2c1s.m
Submission Name	rjkt54815th
Instrument	micrOTOF
ESI	Positive

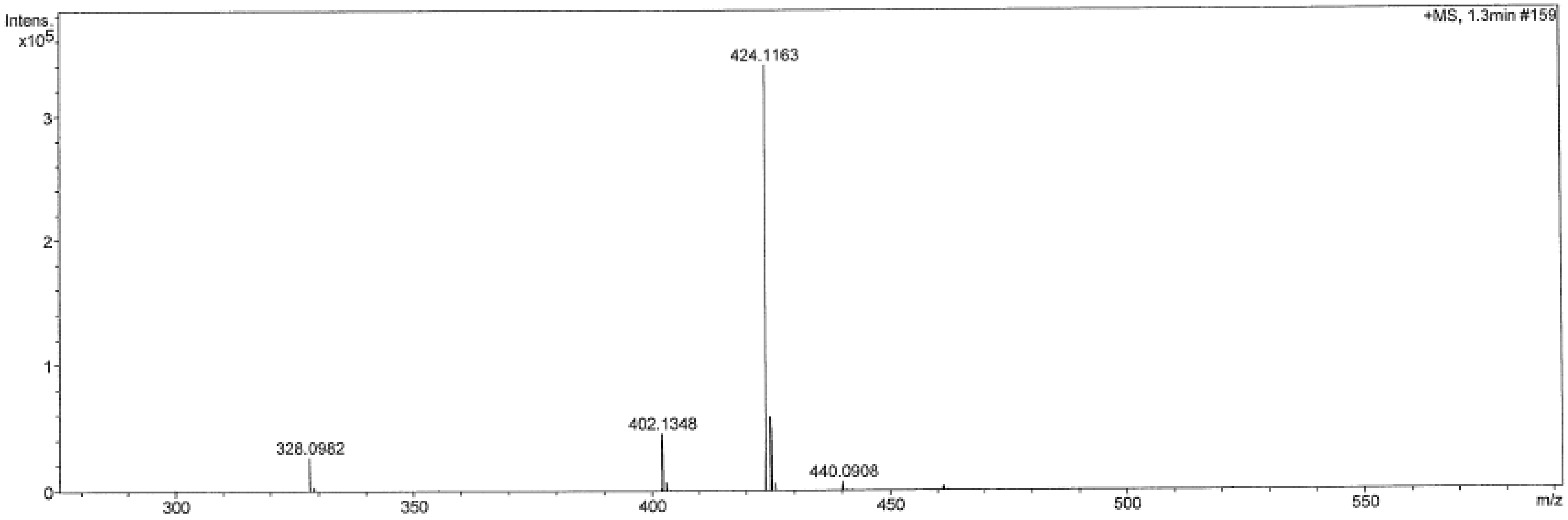

Meas. m/z	#	Formula	m/z	err [ppm]	err [mDa]	mSigma	Mean err [ppm]
402.1348	1	C 21 H 21 F N O 6	402.1347	-0.2	-0.1	49.6	-0.4
424.1163	1	C 21 H 20 F N Na O 6	424.1167	0.8	0.4	37.0	1.0

PerkinElmer Spectrum Version 10.03.07
14 October 2015 21:58

Analyst PEService
Date 14 October 2015 21:58

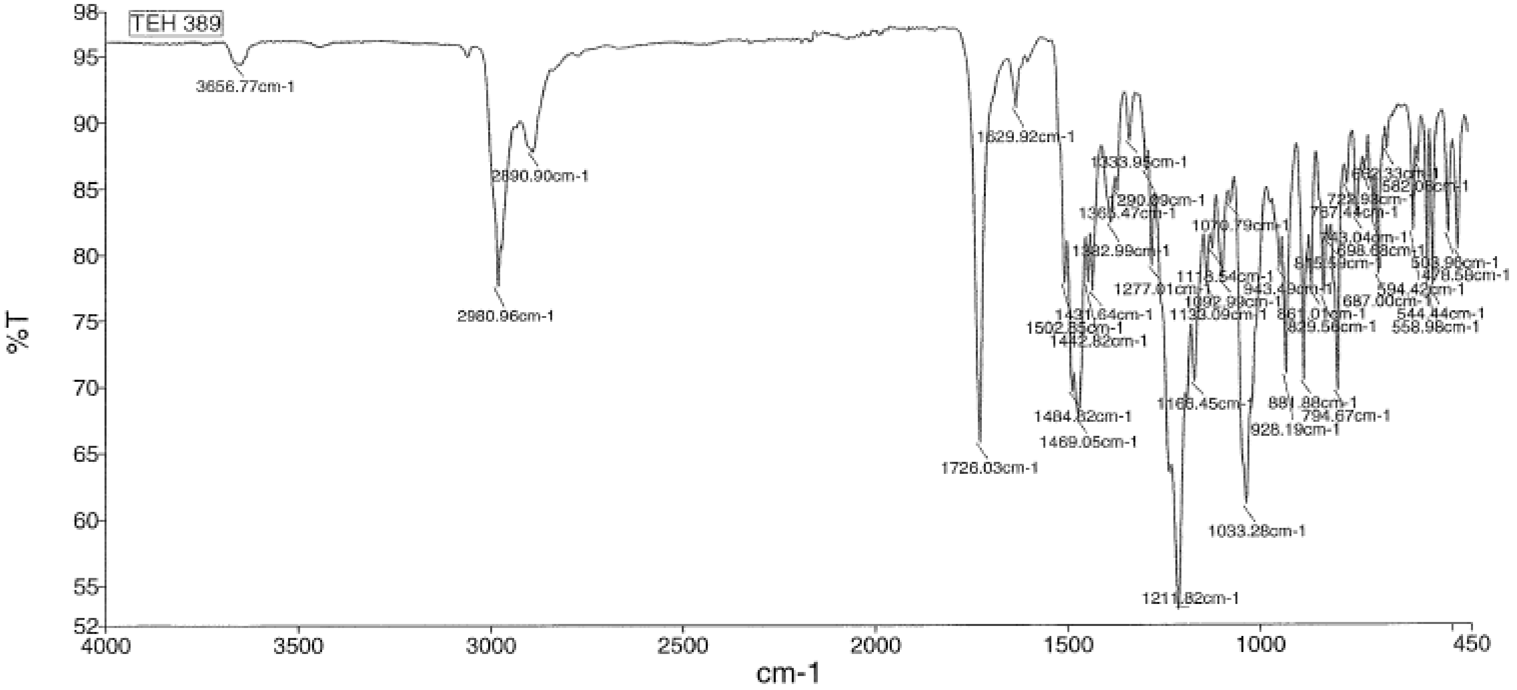

PEService 46 Sample 046 By PEService Date Wednesday, October 14 2015
